# Dynamic assembly of DNA-ceria nanocomplex in living cells generates artificial peroxisome

**DOI:** 10.1038/s41467-022-35472-2

**Published:** 2022-12-14

**Authors:** Chi Yao, Yuwei Xu, Jianpu Tang, Pin Hu, Hedong Qi, Dayong Yang

**Affiliations:** 1 Frontiers Science Center for Synthetic Biology, Key Laboratory of Systems Bioengineering (MOE), Institute of Biomolecular and Biomedical Engineering, School of Chemical Engineering and Technology, Tianjin University, Tianjin, 300350 P. R. China; 2 Zhejiang Institute of Tianjin University, Ningbo, Zhejiang 315200 P. R. China

**Keywords:** DNA nanotechnology, Drug delivery, Peroxisomes, Nanobiotechnology

## Abstract

Intracellular accumulation of reactive oxygen species (ROS) leads to oxidative stress, which is closely associated with many diseases. Introducing artificial organelles to ROS-imbalanced cells is a promising solution, but this route requires nanoscale particles for efficient cell uptake and micro-scale particles for long-term cell retention, which meets a dilemma. Herein, we report a deoxyribonucleic acid (DNA)-ceria nanocomplex-based dynamic assembly system to realize the intracellular in-situ construction of artificial peroxisomes (AP). The DNA-ceria nanocomplex is synthesized from branched DNA with i-motif structure that responds to the acidic lysosomal environment, triggering transformation from the nanoscale into bulk-scale AP. The initial nanoscale of the nanocomplex facilitates cellular uptake, and the bulk-scale of AP supports cellular retention. AP exhibits enzyme-like catalysis activities, serving as ROS eliminator, scavenging ROS by decomposing H_2_O_2_ into O_2_ and H_2_O. In living cells, AP efficiently regulates intracellular ROS level and resists GSH consumption, preventing cells from redox dyshomeostasis. With the protection of AP, cytoskeleton integrity, mitochondrial membrane potential, calcium concentration and ATPase activity are maintained under oxidative stress, and thus the energy of cell migration is preserved. As a result, AP inhibits cell apoptosis, reducing cell mortality through ROS elimination.

## Abstract (web summary)

Artificial organelles can potentially be used support cellular functions, but there is a trade-off between cellular uptake and cellular retention. Here, the authors report the dynamic assembly of DNA-ceria-based artificial peroxisomes in cells, and show they can be used to reduce intracellular ROS.

## Introduction

Reactive oxygen species (ROS) are vital signaling molecules in cellular metabolism that are generated from numerous enzymatic reactions as by-products^1,2^. Generally, the intracellular content of ROS maintains a dynamic balance between production and scavenging^2^. The excessive production or deficient scavenging of ROS leads to oxidative stress, which is closely associated with the pathogenesis and progression of many diseases^3^, including cancers^4^, cardiovascular diseases^5^, and neurodegenerative diseases^6^. The intracellular balance of ROS is maintained by various organelles, including cytoplasm, endoplasmic reticulum, mitochondria, and peroxisome^7^. Peroxisome is a multifunctional organelle that virtually exists in all eukaryotic cells, which contains redox enzymes for the elimination of ROS^8,9^. In pathological regions, the over-generated ROS or the impaired scavenging ability of disordered organelles will lead to cellular damage^10^. Under oxidative stress of ROS, the structural integrity and organelle functions of cells are damaged, and consequently the cell proliferation, differentiation and metabolism are influenced^10^. The introduction of artificial organelles, such as the analogs or substitutes of peroxisome to ROS-imbalanced cells, is an promising route to deal with this ROS damage^11^.

Artificial organelles, which can be regarded as active nanoreactors in cells, are designed to mimic or over-take the function of specific natural organelles^12,13^ and control the cell fates^14,15^. Rationally designed artificial organelles require proper size for cell-uptake, but relatively large structures for long-time cellular retention^16–18^. To overcome this dilemma, dynamic systems are desired to achieve intracellular controlled assembly^19,20^ and perform the function of organelle^21^. As a natural biomacromolecule, deoxyribonucleic acid (DNA) exhibits precise programmability and thus has been employed as a building-block to realize controlled assembly/disassembly^22,23^. In particular, functional DNA sequences can respond to specific intracellular stimuli such as enzymes^24^, ions^25^, and pH^26^, facilitating topological transformation and self-assembly in living cells. Recently, we developed a DNA-based pH-responsive dynamic system that successfully achieved topological transformation from nanoparticles to organelle-like hydrogel architectures specifically mediated by lysosomal acidic condition inside living cells^27^. Moreover, DNA molecule can be easily hybridized with additional components to introduce more functional modules, such as ceria as redox enzyme mimetics, enabling DNA-based materials with organelle-like activities^28^.

Herein, we design a DNA-ceria nanocomplex (DCNC) to achieve intracellular dynamic assembly, and thus realize the in situ construction of artificial peroxisomes, which performs one function of peroxisomes to efficiently scavenge intracellular high-level ROS. Branched-DNA building units are rationally designed with complementary sticky ends to construct DNA assembler (DAS). Ceria interacts with the phosphate backbone of DNA^29^ and assembles with the DAS to form DCNC with proper nanoscale size for efficient cellular uptake. Upon the acidic lysosomal condition^30^, i-motif sequences in branched-DNA can drive the nanoscale DCNC to assemble into stable microscale architecture, termed artificial peroxisome (AP), for improved cellular retention. In a typical AP, ceria exhibits catalyst activity, mimicking the active centers of superoxide dismutase (SOD), catalase (CAT), and peroxidase (POD) in natural peroxisomes; DNA provides the materials system for assembly, and then serves as the matrix of peroxisomes. The dynamic assembly of DCNC plays a key role in improving the function of artificial organelles. In living cells under high ROS conditions, this dynamic material system can efficiently eliminate ROS, thus protecting cells from ROS damage and realizes cell protection.

## Results and discussion

### Design, synthesis, and characterization of DCNC

**Fig. 1:**
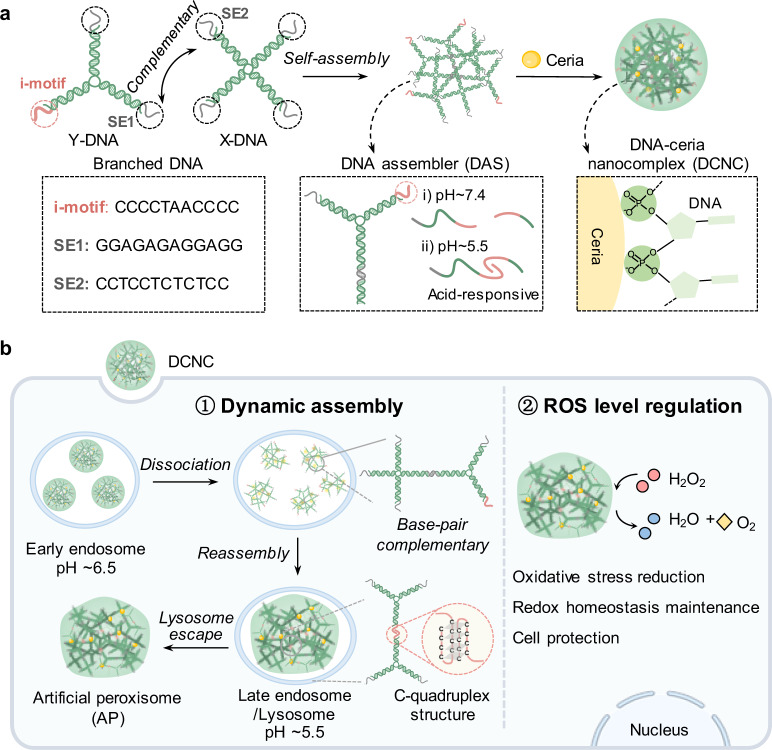
Construction of artificial peroxisome via dynamic assembly of DCNC in a living cell. **a** Molecular design and self-assembly of DCNC. SE, sticky end. **b** Intracellular lysosomal acidic condition-induced assembly of DCNC into artificial peroxisome. ROS reactive oxygen species.

The synthesis of DCNC included three steps (Fig. 1a): (i) the synthesis of X- and Y-shaped branched DNA (X-DNA and Y-DNA). X-DNA contained four sticky ends (SEs), and Y-DNA contained two SEs as well as one i-motif sequence; (ii) the formation of DAS via complementary base pairing of sticky ends (SE1 and SE2) between X-DNA and Y-DNA; (iii) the formation of DCNC based on electrostatic interaction and coordination between ceria and the phosphate acid skeleton of DAS. Ceria has been reported to be the mimetics of enzymes due to its unique redox properties^31^. In particular, ceria-based nanoparticles can serve as ROS eliminator to scavenge excessive intracellular ROS^32^; by decomposing H_2_O_2_ into O_2_ and H_2_O, ceria-based materials can provide cellular protection and alleviate cell damage^33^. In our design, after being internalized by cells, nanoscale DCNCs first partially dissociated in early endosomal slight acidic condition (pH ~6.5), and then reassembled into microscale aggregates through crosslinking of i-motif sequence in late-endosomal/lysosomal acidic condition (pH ~5.5). The aggregates transferred into cytoplasm via lysosomal escape, becoming long-term retained APs, and served as intracellular ROS regulator to achieve the reduction of oxidative stress, the maintenance of redox homeostasis, and the protection of cells (Fig. 1b).

**Fig. 2:**
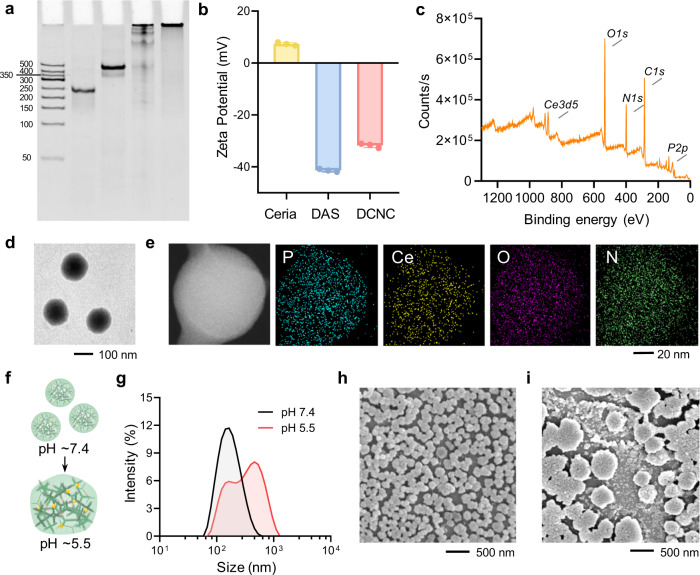
The synthesis and characterization of DCNC. **a** 12% polyacrylamide gel electrophoresis (PAGE) for verifying the synthesis of Y-DNA, X-DNA, DAS, and DCNC. **b** Zeta potential measurement of ceria, DAS, and DCNC. Bars represent mean ± SD (*n* = 3 independent samples). **c** X-ray photoelectron spectroscopy (XPS) analysis of DCNC. **d** Representative transmission electron microscopy (TEM) images of DCNC. **e** TEM element mapping images of DCNC. **f** Schematic illustration of acid-triggered assembly of DCNC. **g** Dynamic light scattering (DLS) analysis of DCNC at pH 7.4 and pH 5.5, respectively. **h**–**i** Representative scanning electron microscopy images of DCNC at pH 7.4 (**h**) and pH 5.5 (**i**).

X-DNA and Y-DNA as building units were rationally designed and synthesized (Supplementary Table 1). Polyacrylamide gel electrophoresis (12% PAGE) was performed to verify the synthesis of DAS and DCNC (Fig. 2a). Lane 1, 2, 3, and 4 represented Y-DNA (Supplementary Fig. 1a), X-DNA (Supplementary Fig. 1b), DAS, and DCNC, respectively. In lane 3, an intense band with smear was observed, validating the formation of DAS; in lane 4, a band was observed in the loading well, confirming that the DCNC was assembled from copies of Y-DNA and X-DNA. Ceria was synthesized by the chemical precipitation method^34^. According to the high-resolution transmission electron microscopy (HRTEM) images, ceria showed spherical morphology and monodispersed size of ~5 nm, with a lattice spacing of 0.2 nm (Supplementary Fig. 2). The size of DAS was measured to be ~43.8 nm by dynamic light scattering (DLS) analysis (Supplementary Fig. 3a). Driven by electrostatic interaction and coordination, ceria (+7.32 mV) easily integrated with DAS (−41.37 mV) to form DCNC (−31.77 mV) (Fig. 2b, c). Transmission electron microscopy (TEM) image suggested that DCNC was spherical, with a mono-distributed size of ~100 nm (Fig. 2d). Elemental mapping images showed that Ce element from ceria and P/N elements from DNA were homogeneously distributed in the nanocomplex (Fig. 2e). When pH changed from 7.4 to 5.5, DCNC assembled into aggregates (Fig. 2f); DLS analysis and scanning electron microscopy (SEM) confirmed that the average size of nanocomplex increased from nanoscale into microscale (Fig. 2g–i). From circular dichroism (CD) spectra analysis, the characteristic peak at 272 nm red-shifted to 276 nm due to the formation of i-motif structure (Supplementary Fig. 4). In contrast, the CD spectra analysis of D_ni_CNC (without i-motif sequence) did not show significant shift at different pH (Supplementary Fig. 5), and the DLS analysis confirmed that the average size of nanocomplex did not increase (Supplementary Fig. 3b), indicating no acid-responsive assembly mediated by the formation of i-motif structure. According to the calibration curve of absorbance *vs*. concentration of ceria, the loading capacity of DCNC towards ceria at 100 μM was calculated to 88.1% (Supplementary Fig. 6 and 7), and there was no significant difference between the loading capacity of D_ni_CNC and DCNC (Supplementary Fig. 8).

To explore the assembly behaviors at different pH conditions, DCNCs were incubated in pH 7.4, 6.5, and 5.5 buffer for 4 h, respectively. The result of agarose gel electrophoresis showed that the smear band of pH 6.5 was more obvious than that of pH 7.4 and 5.5, which was probably due to the weakened interactions between DAS and ceria, as well as the inefficient formation of i-motif structure at pH 6.5 (Supplementary Fig. 9a). These results also supported the proposed mechanism that the DCNC firstly dissociated in early endosome and then reassembled in late endosome/lysosome (Fig. 1b). Then, D_ni_CNC and DCNC were incubated in pH 5.5 solution for 4 h, respectively. A smear band in 2% agarose gel electrophoresis image showed the disassembly of D_ni_CNC, while DCNC still remained in the loading well (Supplementary Fig. 9b). It was inferred that in acid environment DNA was protonated, and thus the electrostatic interaction of DNA-ceria was weakened, leading to the dissociation of D_ni_CNC and DCNC. In DCNC, the dissociated DNA immediately reassembled into larger-sized aggregates through crosslinking of i-motif sequence; in D_ni_CNC, the dissociated DNA would not form aggregates due to the lack of i-motif sequences. After pH being tuned from 5.5 to 7.4, the band of D_ni_CNC appeared in the loading well, indicating that the assembly of D_ni_CNC recovered in neutral buffer (Supplementary Fig. 9c); the aggregates formed by DCNC still retained in the loading well, which was further confirmed by TEM (Supplementary Fig. 10). It was supposed that during the reassembly progress, DNA further assembled by inter-strand entanglement and ceria-DNA coordination, which maintained the aggregation state^27^. Notably, limited by the resolution of agarose gel electrophoresis, the nano-structured D_ni_CNC and DCNC (~100 nm), and the micro-sized aggregates both remained in the loading wells.

### Verification of the catalysis activity of DCNC

**Fig. 3:**
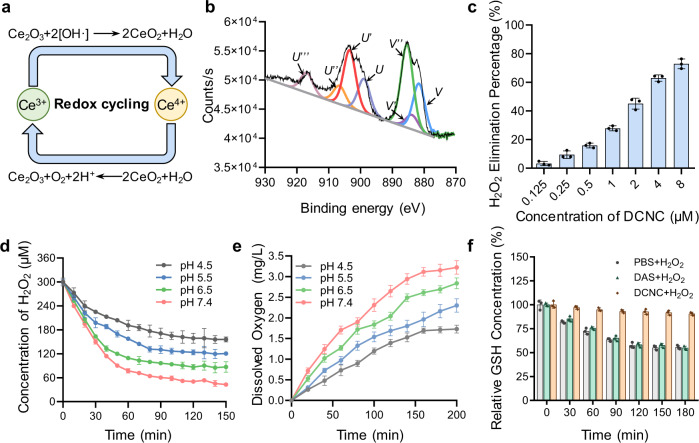
Evaluation of the catalytic activity of DCNC. **a** Schematic illustration of reaction mechanism of H_2_O_2_ decomposition by DCNC. **b** XPS analysis of the chemical valence of cerium in DCNC. **c** Elimination of H_2_O_2_ by varied concentrations of DCNC. **d** Decomposition of H_2_O_2_ at varied pH by the catalysis of DCNC in aqueous solution. **e** Production of O_2_ by the catalysis of DCNC in aqueous solution. **f** Glutathione (GSH) consumption in the present of H_2_O_2_, and treated with PBS, DAS, and DCNC, respectively. Bars represent mean ± SD (*n* = 3 independent samples).

The elimination effect of DCNC towards ROS was then explored, during which DCNC catalyzed ROS into nontoxic H_2_O and O_2_ (Fig. 3a). The performance of the catalytic activity of DCNC depends on the Ce^3+^/Ce^4+^ ratio^35^. X-ray photoelectron spectroscopy (XPS) of cerium in DCNC showed that the chemical valence of cerium in DCNC was a mixed state of trivalence and tetravalence, and the Ce^3+^/Ce^4+^ ratio was 1/9. The relatively high level of cerium in +4 oxidation state endowed the DCNC with enzyme-like activity (Fig. 3b).

The enzyme-like catalysis activities of DCNC, including SOD-, CAT-, and POD-like activity were evaluated. The SOD-like activity of DCNC was determined via nitroblue tetrazolium (NBT) assay by monitoring the decomposition of superoxide anion (O_2_^·−^). O_2_^·−^ was produced by the system of riboflavin, methionine, and NBT under UV irradiation, and O_2_^·−^ level was evaluated by measuring the absorbance at 560 nm. The results showed that O_2_^·−^ level decreased significantly with the increase of DCNC concentration, presenting SOD-like activity of DCNC (Supplementary Fig. 11). CAT-like activity was detected via ammonium molybdate (AMT) spectrophotometric method, in which H_2_O_2_ interacted with AMT to form stable yellow complex, whose absorbance at 405 nm was proportional to the concentration of H_2_O_2_ (Supplementary Fig. 12). POD-like activity was evaluated via colorimetric assay using 2, 2’-Azinobis-(3-ethylbenzthiazoline-6-sulfonate) (ABTS) as substrate. In the presence of H_2_O_2_ and DCNC, ABTS was oxidized into green-colored ABTS^·+^_,_ with the characteristic absorption peaks around 405 nm (Supplementary Fig. 13). As shown in Fig. 3c, with the increase of DCNC concentration, H_2_O_2_ elimination percentage improved significantly. In 300 μM H_2_O_2_ solution, 72.8% of H_2_O_2_ was eliminated by 8 μM DCNC at pH 7.4 in 60 min.

The CAT-like activity of DCNC at varied pH was further evaluated by monitoring H_2_O_2_ decomposition and O_2_ production. DCNC was confirmed to decompose H_2_O_2_ at both acidic and neutral conditions; the decomposition percentage reached 85.8% at pH 7.4 in 150 min (Fig. 3d), and there was no significant difference on the catalytic activity between D_ni_CNC and DCNC (Supplementary Fig. 14). The O_2_ production at varied time points were then detected by using dissolved oxygen meter. When pH was changed from 7.4 to 4.5, the generation efficiency of O_2_ decreased from 3.22 to 1.73 mg/L, indicating that DCNC possessed the strongest CAT-like activity at pH 7.4 (Fig. 3e). These results indicated that the endogenous ROS can be efficiently transformed to O_2_ by DCNC at physiological condition. Due to the CAT-like activity of decomposing H_2_O_2_, DCNC was potential to serve as antioxidant to prevent glutathione (GSH) from being oxidized into oxidized glutathione (GSSH) by H_2_O_2_. PBS buffer, DAS, and DCNC were added to the mixture of GSH and H_2_O_2_, respectively, and then GSH consumption were monitored at varied time points (Fig. 3f). PBS group showed 44.4% GSH consumption by H_2_O_2_, while GSH concentration in DCNC group maintained 90.6%, verifying that DCNC was an efficient protector against GSH oxidization under high H_2_O_2_ concentration.

### Intracellular dynamic assembly of DCNC

**Fig. 4:**
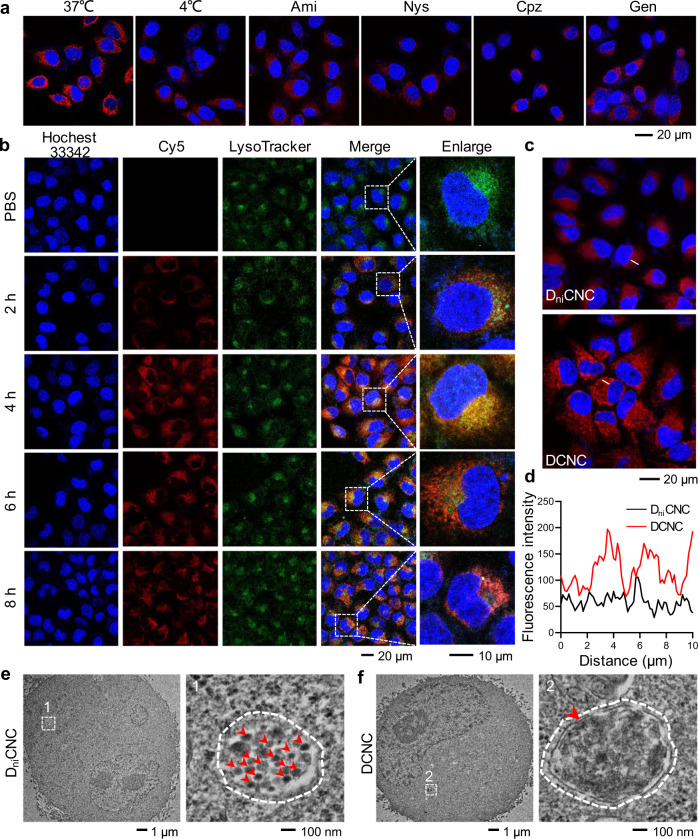
Lysosomal acidic condition-induced assembly of DCNC. **a** Representative confocal microscopy images of MCF-7 cells treated with different endocytosis inhibitors and then incubated with 1 μM DCNC for 6 h. Ami amiloride, Nys nystatin, Cpz chlorpromazine, Gen genistein. **b** Representative confocal microscopy images of MCF-7 cells after incubation with DCNC for varied time durations. Cell nuclei were stained with Hoechst 33342 (blue); late endosome/lysosomes were stained with LysoTracker (green). **c** Representative confocal microscopy images of MCF-7 cells that were incubated with D_ni_CNC and DCNC for 48 h, respectively. **d** The line-scanning plots for fluorescence intensity quantitative analysis. **e**, **f** Representative TEM images and partial enlargement of MCF-7 cells that were treated with D_ni_CNC (**e**) and DCNC (**f**), respectively, for 6 h. The white dotted line indicated lysosomes; the red arrows indicated nanocomplex (**e**) and aggregates (**f**).

The stability of DCNC was tested by incubating DCNCs with 10% FBS for varied time durations. According to the results of PAGE, no obvious degradation was observed during 60-h incubation (Supplementary Fig. 15). These results showed the strong interactions between DNA and ceria, and indicated that ceria particles stably stayed with DNA and would not leak out from the complex. In addition, the acid-responsive assembly of DCNC mediated by the formation of i-motif structure still occurred in cell culture medium (Supplementary Fig. 3c). To investigate the elimination effects of DCNC towards ROS, the cellular uptake behavior of Cy5-labeled DCNC by MCF-7 cells was then evaluated. Uptake efficiency was analyzed by flow cytometry (FCM) at varied time points. The fluorescence signal intensity of Cy5 increased with time in 12 h, indicating that DCNC was efficiently internalized (Supplementary Fig. 16). The cellular uptake pathway of DCNC was studied by treating MCF-7 cells with specific endocytic inhibitors^36^. Compared with the control group, the cellular uptake of DCNC was significantly inhibited by low temperature, amiloride (micropinocytosis inhibitor), nystatin/genistein (caveolae-mediated endocytosis inhibitor), and chlorpromazine (clathrin-mediated endocytosis inhibitor), indicating that the cellular uptake was an ATP-dependent process, which relied on micropinocytosis, caveolae-mediated endocytosis, and clathrin-mediated endocytosis (Fig. 4a, Supplementary Fig. 17). This internalization pathway indicated that DCNC was internalized to early endosomes (pH ~6.5), and then translocated to the late endosome/lysosome (pH ~5.5). The colocalization between Cy5-labeled DCNC and lysosomes was tracked by confocal laser scanning microscopy (CLSM). DCNC entered cells, and the fluorescence signals of Cy5 and LysoTracker were not overlapped at 2-h incubation (Fig. 4b). After 4-h incubation, the intracellular fluorescence intensity of Cy5 increased, co-localized with the fluorescence of LysoTracker, indicating that DCNC had translocated into late endosome/lysosomes. In late endosomal/lysosomal acidic environment, DCNC tended to dissociate and reassemble into larger aggregates through acid-responsive i-motif structure. At the time point of 6 h, less fluorescence signal of DCNC coincided with that of late endosome/lysosomes, suggesting that the aggregates began to escape from lysosomes. In the magnified images, the red fluorescence presented as clusters, reflecting that DCNC remained aggregation after escaping from lysosomes. At the time point of 8 h, most of the aggregates had translocated into cytoplasm and became APs.

To perform the role of artificial organelles, the APs were required to present long-time retention in complex biological and remained the function of cellular metabolism regulation. Thus, the intracellular retention of APs was studied. Fluorophore-labeled DCNC was incubated with MCF-7 cells for 12 h to adequately enter cells, and then the culture medium was changed into fresh medium for incubation. According to the CLSM images and corresponding quantitative analysis, the intracellular fluorescence intensity of DCNC was significantly higher than that of D_ni_CNC (Fig. 4c, d). In addition, the escape kinetics of DCNC and D_ni_CNC was monitored by measuring the fluorescence intensity of the cell culture supernatant. The results showed that the intensity in DCNC group was significantly lower than that in D_ni_CNC group during 60-h incubation (Supplementary Fig. 18). These results indicated that the assembly of DCNC from monomers to aggregates improved intracellular retention time. The process of dynamic assembly was further observed at the subcellular level. D_ni_CNC and DCNC were incubated with MCF-7 cells for 12 h, respectively, in which half D_ni_CNC and DCNC were Cy5-labeled and the other half were black hole quencher (BHQ)-labeled. Upon the assembly behavior, Cy5 fluorophores were supposed to be quenched by BHQ, leading to the decrease in fluorescence intensity, and this process was verified via the analysis of fluorescence intensity by CLSM. Compare to D_ni_CNC group, the fluorescence intensity of DCNC group significantly decreased, indicating that the assembly depended on the formation of i-motif structures (Supplementary Fig. 19). The assembly of DCNC in lysosomes was further observed by TEM. In D_ni_CNC group, the magnified image of a lysosome showed that spherical nanoparticles with the size of ~100 nm distributed in the lysosome (Fig. 4e, Supplementary Fig. 20a), indicating that D_ni_CNCs did not form aggregates due to the lack of i-motif sequences. In DCNC group, the images showed amorphous aggregates in the lysosome (Fig. 4f, Supplementary Fig. 20b), confirming the assembly of DCNC in lysosomes.

The biocompatibility of DCNC was further evaluated via cell counting kit-8 (CCK-8) assay. DCNC showed good biocompatibility over a wide range of concentrations from 0.25 to 4 μM, and even promoted the proliferation of MCF-7 cells (Supplementary Fig. 21). The concentration of 1 μM was chosen for the following experiments. The biocompatibility of other groups (D_ni_AS, DAS, and D_ni_CNC) at varying concentrations was also tested. The intracellular catalytic activities of ROS elimination were then measured through the change of intracellular ROS level. The ROS level was evaluated by the fluorescence intensity of a ROS probe, 2’−7’dichlorofluorescin diacetate (DCFH-DA), which was a fluorogenic dye that interacted with ROS and showed green fluorescence. After incubating DCNC with MCF-7 cells for varied time, fluorescence microscopy images showed that the green fluorescence intensity began to decrease at 4-h incubation, indicating that DCNC effectively reduced the intracellular ROS level (Supplementary Fig. 22a and 22b). On the other hand, the ability of DCNC to produce O_2_ in living cells was investigated. Tris (4, 7-diphenyl-1, 10-phenanthroline) ruthenium (II) dichloride ([Ru(dpp)_3_]^2+^Cl_2_) was used as the quenchable indicator for O_2_ generation. The fluorescence images showed that the red fluorescence intensity decreased obviously in 4 h (Supplementary Fig. 22c and 22d). These results demonstrated the intracellular catalytic activity of DCNC to decompose ROS into O_2_.

### Intracellular effects of artificial peroxisome

**Fig. 5:**
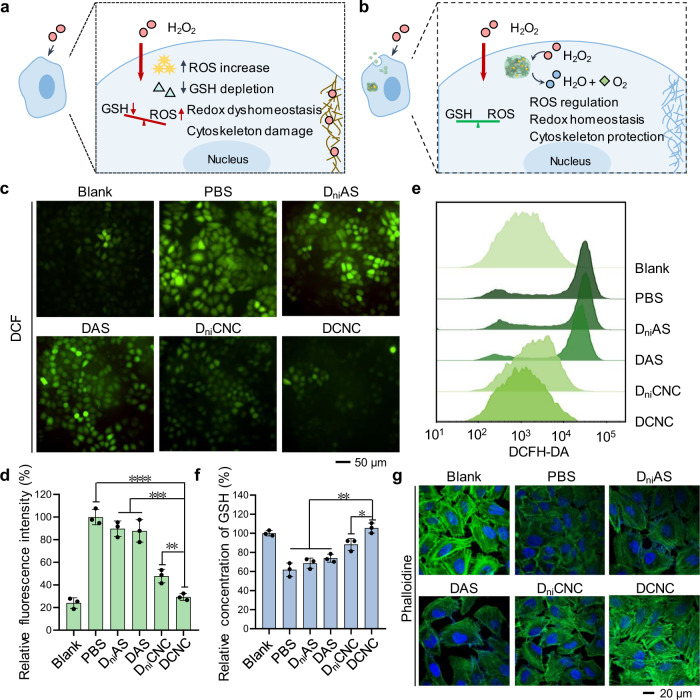
ROS regulation effects of DCNC in living cells. **a** Schematic illustration of cell effects upon the treatment of high-concentration H_2_O_2_. **b** Schematic illustration cell effects upon the treatment of DCNC and then high-concentration H_2_O_2_. **c** Representative fluorescence microscopy images showing ROS levels measured with DCF fluorescent probe after incubation with different materials and treatment with H_2_O_2_. **d** Mean fluorescence intensity of DCF analyzed by image J software. Bars represent mean ± SD (*n* = 3 independent samples). (**P < 0.01; ***P < 0.001; ****P < 0.0001, calculated by two-tailed unpaired *t* test). **e** Flow cytometry of ROS levels in the MCF-7 cells treated with a DCFH-DA fluorescent probe after incubation with different materials and H_2_O_2_. **f** Relative concentration of intracellular GSH in the MCF-7 cells treated with different materials and H_2_O_2_. Bars represent mean ± SD (*n* = 3 independent samples). (*P < 0.05; **P < 0.01, calculated by two-tailed unpaired *t* test). **g** Representative confocal microscopy images of F-actin stained with FITC-phalloidine after incubation with different materials and treatment with H_2_O_2_.

Intracellular high ROS concentration can interfere cell metabolism and induce oxidative stress, causing cell damage and even apoptosis^37^. To verify that DCNC was able to regulate the intracellular ROS level and protect cells, H_2_O_2_ was introduced to simulate the oxidative stress condition (Fig. 5a, b). MCF-7 cells were incubated with a series of materials (PBS, D_ni_AS, DAS, D_ni_CNC, and DCNC) for 12 h to ensure the sufficient internalization of materials. Then the medium was replaced with fresh one and cultured for another 24 h to complete intracellular assembly process and the formation of AP. The cells were treated with high-concentration H_2_O_2_ (1 mM) and cultured at normal conditions to observe the subsequent biological effect. The intracellular ROS level was measured with ROS probe. After being treated with high-concentration H_2_O_2_, the green fluorescence intensity in PBS group was 4.17 times of that in blank group, confirming the rise of intracellular ROS level (Fig. 5c, d). D_ni_AS and DAS groups showed 3.74- and 3.66-times fluorescence intensity of blank group, indicating that DNA as only component was not able to effectively eliminate ROS. In contrast, the fluorescence intensity of D_ni_CNC and DCNC groups decreased to 1.99 and 1.22 times of blank group; remarkably, DCNC group showed significant elimination of ROS via the formation of AP. The fluorescence intensity in cells was then analyzed by FCM, which was consistent with the results of fluorescence microscopy, that DCNC effectively regulated intracellular ROS level (Fig. 5e). The ROS elimination was further monitored with time-variation. MCF-7 cells were treated with high-concentration H_2_O_2_ (1 mM) and incubated with DCNC. According to the results of fluorescence microscopy, intracellular ROS level gradually decreased with DCNC incubation in 48 h (Supplementary Fig. 23). These results demonstrated that the intracellularly-constructed AP via the dynamic assembly of DCNC possessed strong ability of ROS elimination, and maintained redox homeostasis via the regulation of intracellular ROS level.

GSH is a predominant intracellular antioxidant, and also acts as cofactors of many detoxifying enzymes^38^. In the redox reaction between GSH and H_2_O_2_, GSH donates electrons to H_2_O_2_ and catabolize H_2_O_2_ into H_2_O and O_2_. The excessive consumption of intracellular GSH leads to increased oxidative stress, reduced supply of oxygen and nutrients to the cells, and eventually cell apoptosis. The intracellular GSH contents of cells incubated with varied materials and then treated with H_2_O_2_ were measured. The concentration of GSH in PBS group reduced 38.1% compared to that of blank group, confirming that ROS increase caused GSH depletion, and led to the dyshomeostasis of cells (Fig. 5f). The GSH concentration in D_ni_AS and DAS groups reduced 31.3% and 26.1% respectively, indicating that pure DNA materials could not decompose excessive H_2_O_2_. Remarkably, the GSH level in DCNC group recovered to equal to that in PBS group, indicating the resistance of GSH consumption by AP through ROS elimination. To validate the preservation effects of DCNC toward cells at molecular level, western blot on the expression of Bcl-2 protein was performed. Under oxidative stress, the expression level of Bcl-2 would be inhibited by the high-concentration ROS. According to the results, H_2_O_2_ significantly decreased the expression level of Bcl-2, while the Bcl-2 expression maintained at the normal level after DCNC treatment, indicating the preservation effects of AP to cells through ROS elimination (Supplementary Fig. 24).

Cytoskeleton is a network to maintain cellular morphology and important cell functions, which is susceptible to oxidative stress. Evidences have supported that the perturbation of cytoskeletal proteins is the initial step of oxidant-induced cell damage^39^. To investigate the influence of oxidative stress to cytoskeleton, MCF-7 cells were incubated with varied materials and treated with H_2_O_2_, and β-actin were stained with FITC-labeled phalloidin. As shown in CLSM images, the fluorescence intensity of cytoskeleton in PBS group was notably decreased, and the cells exhibited shrunken morphology (Fig. 5g). This phenomenon was due to the production of ·OH in the process of H_2_O_2_ metabolism, inducing the remodeling of actin cytoskeleton and reducing the formation of stress fibers. The cytoskeleton in DCNC-treated group showed clear actin filaments in CLSM images, suggesting that with the protection of AP, cells maintained the structural integrity under oxidative stress.

### Preservation effects of DCNC towards cells

**Fig. 6:**
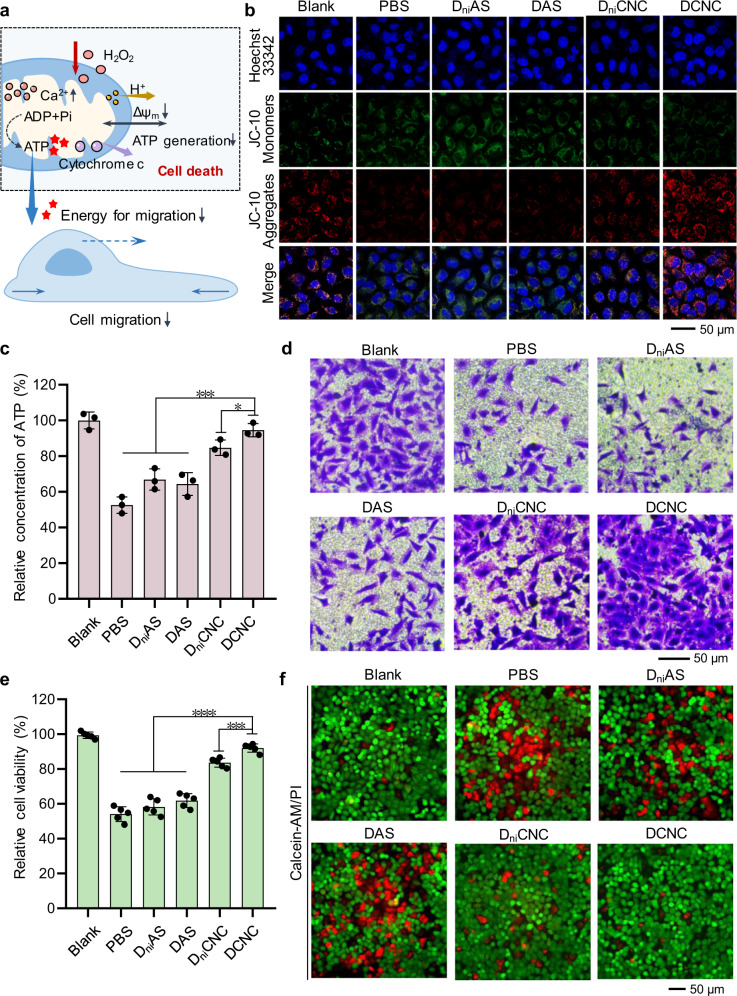
Preservation effects of DCNC towards cells. **a** Schematic illustration of mitochondria damage caused by high-concentration H_2_O_2_. **b** Representative fluorescence microscopy images showing the JC-10-staining MCF-7 cells after incubation with DCNC for different time and then treated with H_2_O_2_. **c** Relative concentration of intracellular ATP. Bars represent mean ± SD (*n* = 3 independent samples). (*P < 0.05; ***P < 0.001, calculated by two-tailed unpaired *t* test). **d** Representative microscopy images of MCF-7 cells treated with a series of materials in a Transwell cell invasion device. **e** Cell viability of MCF-7 cells after incubation with different materials and treatment with H_2_O_2_. Bars represent mean ± SD (*n* = 5 independent samples). (***P < 0.001; ****P < 0.0001, calculated by two-tailed unpaired *t* test). **f** Representative fluorescence microscopy images showing living/dead cell assay of the MCF-7 cells treated with H_2_O_2_ and different materials. Living and dead MCF-7 cells were stained with calcein-acetoxymethylester (calcein-AM, green) and propidium iodide (PI, red), respectively.

The accumulation of H_2_O_2_-induced oxidative stress would cause oxidative damage to mitochondria, leading to the decrease in mitochondrial membrane potential and the rise of calcium concentration (Fig. 6a). Mitochondrial membrane potential (∆ψ_M_) is one of the key parameters of mitochondrial function. JC-10 mitochondrial membrane potential assay was used to test the change of mitochondrial membrane potential upon the treatment of a series of materials and H_2_O_2_. Green fluorescence was from JC-10 monomers and the red was from JC-10 aggregates; the decreased red intensity represented the drop of mitochondrial membrane potential. According to Fig. 6b, red fluorescence intensity in PBS group was significantly lower than that in blank group, reflecting the oxidative damage to mitochondria. The mitochondrial membrane potential in DCNC group maintained normal level, indicating that DCNC assembled into AP, effectively eliminating ROS and protecting cells from mitochondrial damage. The change of mitochondrial membrane potential of cells protected by DCNC was further monitored in 48 h. According to the CLSM images, mitochondrial membrane potential increased regularly after the addition of DCNC, indicating the resistance toward oxidative stress by AP (Supplementary Fig. 25).

H_2_O_2_-induced oxidative would cause the increase of intracellular calcium (Ca^2+^) concentration, and thus Ca^2+^ concentration during H_2_O_2_ accumulation and elimination was detected. The presence of AP in DCNC group effectively regulated H_2_O_2_ level and maintained Ca^2+^ concentration (Supplementary Fig. 26). In a continued monitoring with time duration, where cells were damaged by high-concentration of ROS, DCNC eliminated the overdosed H_2_O_2_ and decreased calcium concentration in 48 h (Supplementary Fig. 27). In addition, H_2_O_2_ accumulation influenced ATPase activity and cause the decrease of ATP content in cells. Relative ATP content of PBS group decreased 46.4% compared to that of blank group, while the ATP level of DCNC group showed negligible change (Fig. 6c). Since mitochondria are the “power plant” of cell that produce energy, mitochondrial damage may influence the ATP-dependent cell migration. Transwell assay was used to detect the invasion ability of cells mediated by cell migration. The presence of H_2_O_2_ significantly weakened cell invasion; the level of cell migration and invasion in DCNC group was equal to that observed in blank group (Fig. 6d, Supplementary Fig. 28).

H_2_O_2_ can be transported through cell membrane and induce concentration-dependent cytotoxicity; high concentration can cause cell apoptosis and death. The cytotoxicity of varied H_2_O_2_ concentrations to MCF-7 cells was confirmed by CCK-8 assay (Supplementary Fig. 29). Then the protection effect of AP on cells was investigated. After incubation with a series of materials, MCF-7 cells were treated with 1 mM H_2_O_2_ (Fig. 6e). Compared to the blank group, the cell viability in PBS group decreased 45.9%. Groups of pure DNA materials D_ni_AS and DAS showed negligible protection toward mortality. In the presence of ceria, cell viability in D_ni_CNC group rose to 83.6%. Remarkably, cell viability in DCNC group remained 92.1%, due to the prolonged retention time of assembled AP in cells. To visually evaluate the protection on cells, living/dead cell analysis was then performed to test the viability of MCF-7 cells incubated with a series of materials and treated with H_2_O_2_. As shown in the fluorescence microscopy images, after treated with H_2_O_2_, PBS, D_ni_AS, and DAS groups contained 36.2%, 33.5% and 21.9% of PI-stained dead cells, respectively, reflecting the cytotoxicity of H_2_O_2_ and inefficient protection by pure DNA materials (Fig. 6f, Supplementary Fig. 30). In contrast, D_ni_CNC and DCNC groups contained 21.9 and 10.7% dead cells respectively, due to the decomposition of H_2_O_2_ and protective effect by the materials, especially by AP assembled from DCNC. Besides, apoptosis level of cardiomyocytes was determined by the FITC-annexinV/PI (FITC, fluorescein isothiocyanate; PI, propidium iodide) double-staining flow cytometry assay (Supplementary Fig. 31). DCNC showed the highest inhibition effect of cell apoptosis, demonstrating an effectively reduced cell mortality via inhibiting apoptosis.

In conclusion, we developed a DNA-ceria nanocomplex that responds to intracellular conditions, realizing the in-situ construction of artificial peroxisome via dynamic assembly. The nanocomplex was synthesized with the correct size for cellular uptake; where in response to late-endosomal/lysosomal acidic conditions, the nanocomplex is transformed into bulk-sized artificial peroxisomes to achieve enhanced retention. The artificial peroxisome performed one function of peroxisomes to efficiently scavenge the high-concentration ROS and prevent cells from oxidative stress. Compared with other strategies for constructing artificial organelles, our strategy achieved the in-situ dynamic assembly to form functional artificial peroxisomes in living cells.

This chemical material system can be further expanded, as discussed below. (1) The dynamic assembly of DCNC was triggered by the acidic condition in late endosome/lysosome, and the capability of DCNC on ROS scavenging was also proved effective in more cell lines, such as H9C2 cell and BEAS-2B cell (Supplementary Fig. 32). (2) For DNA material itself, many functional modules can be obtained through DNA sequence design, which can be integrated to DNA nanostructures to achieve more abundant chemical properties and biological functions, facilitating the intracellular dynamic assembly of artificial organelles; (3) For the hybridization of DNA with other materials, DNA molecules can be coupled with many other functional units including organic and inorganic materials, and thus the artificial organelles could be endowed with more biological activities.

In the current work, the feasibility of the intracellular in-situ construction of artificial peroxisome from DNA-ceria nano complex has been demonstrated, and the capability of this artificial peroxisome on ROS scavenging has been validated in vitro. For in vivo applications, challenges including the influence of complicated conditions, such as the acidic extracellular microenvironments and intracellular compartments, would need to be considered. (1) Since the aggregation of DCNC is irreversible, the delivery strategy of DCNC would be important to ensure assembly in the target region. For example, liposomes could be potential carriers for the efficient loading and delivery of DCNC. (2) In the design of DNA materials, targeting modules such as aptamers could be introduced to improve the specificity of materials to tumors or targeted organs. (3) Complicated conditions including acid, metal ions, and other biological stimuli could potentially provide additional possibilities for dynamic assembly and construction of artificial organelles. We could exploit these complicated biological conditions, as well as the differences of biological stimuli at cellular and subcellular levels, to achieve dynamic assembly in targeted sites.

## Methods

### Synthesis of ceria

The monodispersed ceria was synthesized via the alkaline-based precipitation method published by Perez et al. 10 mL of an aqueous solution of 1 M cerium nitrate and 0.1 M dextran T-10 were prepared and mixed by vortex. The solution was then added to 30 mL of 25% ammonium hydroxide dropwise, and the mixture was stirred at 25 °C for 24 h. The particles were centrifuged three times at 1500 × *g* for 10 min to remove debris and unattached dextran. Finally, the particles were suspended in PBS and stored at 4 °C.

### Synthesis of DAS

For the synthesis of Y-shaped and X-shaped DNA (Y-DNA and X-DNA, 20 μM), three (Y1-, Y2-, and Y3-DNA) or four components (X1-, X2-, X3-, and X4-DNA) were mixed together at the equal molar ratio in 1 × TAE/Mg^2+^ buffer (Tris–acetic–EDTA, 40 mM Tris base, 20 mM acetic acid, 2 mM EDTA, and 12.5 mM magnesium acetate, pH 8.0) and then added to the Eppendorf (EP) tube. The mixture was heated up to 95 °C for 1 h and slowly annealed to 10 °C. For the preparation of DAS, Y-DNA and X-DNA were mixed together at the molar ratio of 2:1 in TAE/Mg^2+^ buffer. The mixture was first incubated at 35 °C for 1 h with the vibration speed of 1000 rpm, and then heated to 95 °C as well as slowly annealed to 10 °C. The molar concentration of DAS is determined on the basis of the concentration of X-DNA.

### Synthesis of DNA-ceria nanocomplex (DCNC)

DCNC was synthesized by adding extra concentration of ceria in the DAS. 20 μM X-DNA was mixed with 20 μM Y-DNA at the molar ratio of 1:2 in TAE/Mg^2+^ buffer, forming into DAS. The obtained DAS was further mixed with 100 μM ceria suspension at the volume ratio of 3:17. After ultrasound for 10 min, the mixture was heated up to 95 °C for 1 h and slowly annealed to 10 °C, forming into DCNC. The concentration of DCNC was determined on the basis of the final concentration of X-DNA (1 μM).

The synthesized DCNC were centrifuged at 12,000 × *g* for 5 min and washed three times by 1 × TAE/Mg^2+^ buffer (pH 8.0) to achieve the purification step. Then the loading capacity of ceria was determined by the difference between the concentration of original ceria, and ceria remained in the supernatant after centrifugation.

The absorbance value at 300 nm (*A*_300_) of ceria at different ceria concentration (*C*_ceria_) was first determined.

$${A}_{{{{{\rm{300}}}}}}=0.0006443\ast {C}_{{{{{\rm{ceria}}}}}}-0.002103\quad{R}^{2}=0.9922$$

The loading capacity of DCNC towards ceria at different concentration were then tested, and the loading capacity of DCNC towards ceria at 100 μM reached 88.1%.

### Acidity-triggered formation of aggregates from DCNC

The synthesized DCNC were centrifuged at 12,000 × *g* for 5 min and then suspended in 1 × TAE/Mg^2+^ buffer (pH 5.5) to form aggregates.

### Polyacrylamide gel electrophoresis (PAGE) and agarose gel electrophoresis

The construction of Y-DNA, X-DNA, DAS, and DCNC was verified by using 12% PAGE. Each sample (X, Y-shaped DNA, DAS, and DCNC, 5 μL) was mixed with 6× loading buffer (1 μL) and analyzed by 12% PAGE gel at 120 V for 45 min in 1 × TBE buffer. The bands were stained with ethidium bromide (EB), visualized by UV illumination (302 nm), and photographed by the JS-680B gel imaging analysis system. The reassembly of DCNC at pH 5.5 was verified by 2% agarose gel electrophoresis.

### Circular dichroism (CD) spectra measurement

The samples were diluted to 0.5 μM. 200 μL samples were loaded into circular cuvette. The absorbance values of the samples were measured at wavelength ranging of 220~320 nm by a CD chiroptical spectrometer (J-810, Jasco, Japan) at room temperature.

### H_2_O_2_ decomposition assay

The H_2_O_2_ concentrations at series of time points were measured by using AMT spectrophotometric method. Different concentrations of DCNC (0.125, 0.25, 0.5, 1.0, 2.0, 4.0, and 8.0 µM) and 300 μM H_2_O_2_ were mixed in PBS (pH 7.4) at 37 °C or a total of 300 μM of H_2_O_2_ and DCNC (8 μM) were mixed in PBS (pH 4.5, 5.5, 6.5, and 7.4) at 37 °C, followed by measuring the absorbance at 400 nm (*A*_400_) using a microplate reader every 10 min until 150 min to evaluate the H_2_O_2_ concentration.

### Oxygen generation

In order to verify the capability of H_2_O_2_ decomposition and O_2_ production, PBS, DAS, and DCNC (8 μM) were mixed with 10 mL PBS containing 1 mM H_2_O_2_ at 37 °C. Catalyst capability at different (pH 4.5, 5.5, 6.5, and 7.4) were also conducted. The dissolved O_2_ concentration was real-time monitored by a dissolved oxygen meter (P903, YOKE instruments, China).

### GSH consumption

To confirm that the addition of materials could protect GSH from being oxidized into GSSH by H_2_O_2_, PBS, DAS (8 μM), and DCNC (8 μM) were incubated with 25 µL GSH (1 mM) and 25 µL H_2_O_2_ (1 mM) at 37 °C for different time points (range from 30 to 180 min). After the incubation, materials were separated by centrifugation, and the supernatant was collected for GSH measurement by GSH kit. 10 µL of supernatant was added into 100 µL of GSH kit reaction mixture, and measured by UV-vis to determine GSH concentration after 25 min’s reaction.

### TEM of cell samples

For TEM imaging, MCF-7 cells were first treated with DAS and DCNC for 6 h, respectively. Cells were washed three times with PBS and then collected for further fixation and ultrathin sections. TEM images of MCF-7 cells were observed using a transmission electron microscope (JEM-1400 Flash, Japan).

### Cell culture

MCF-7 cells (CVCL_0031), H9C2 cells (CVCL_A0TS), and BEAS-2B cells (CVCL_0168) were purchased FENGHUISHENGWU Technology Co., Ltd. The human breast carcinoma cell line MCF-7 cells were routinely cultured with Dulbecco’s modified eagle medium/high glucose (DMEM-H) (4.5 g/L glucose, 4.0 mM l-glutamine) supplemented with 10% (v/v) FBS, 100 U/mL penicillin, 100 mg/mL streptomycin and 50 mg/mL gentamycin sulfate under the condition of 5% CO_2_ at 37 °C.

### Cellular uptake test

MFC-7 cells were seeded into six-well cell culture dish and cultured overnight to allow the cells to attach onto the glass bottom. Afterwards, Cy5-labeled DCNC was added at different time points respectively to each well with the final concentration of 100 nM. Before the end of incubation, the cells were stained by LysoTracker Green DND-26 for 2 h, and fixed with 4% fixative solution (paraformaldehyde) for 20 min, and then washed by PBS for three times. The cells were stained with Hoechst 33342 (5 μg/mL) for another 15 min. Finally, the fluorescence was observed by confocal laser scanning microscope (CLSM 800 with Airyscan, Zeiss).

### Escape kinetics

D_ni_CNC and DCNC were stained by SYBR Green I for 1 h and then washed by TAE/Mg^2+^ buffer for two times. MCF-7 cells were incubated with different materials (PBS, D_ni_CNC, and DCNC) for 12 h, respectively. The culture medium was changed into fresh one and the cells were cultured continuously. The culture medium was collected after incubation for 12, 24, 36, 48, and 60 h, respectively. The fluorescence intensity of the collected media was measured by a microplate reader (BioTek, SYNERGY H1). Excitation: 490 nm; Emission: 530 nm.

### Intracellular O_2_ levels measurement

Intracellular O_2_ levels of MCF-7 was indicated with [Ru(dpp)_3_]^2+^Cl_2_ (tris (4, 7-diphenyl-1,10-phenanthroline) ruthenium (II) dichloride). DCNC were incubated with MCF-7 cells at different time points, respectively. The O_2_ indicator [Ru(dpp)_3_]^2+^Cl_2_ and Hoechst 33342 was added, with the final concentration of 10 μg/mL and 5 μg/mL, respectively. Then the intracellular fluorescence was observed by CLSM.

### Intracellular ROS imaging

MCF-7 cells were first incubated with different materials (PBS, D_ni_AS, DAS, D_ni_CNC, and DCNC) for 12 h, ensuring that materials can enter cells sufficiently. And then the culture medium was changed into fresh one and cultured for another 24 h to ensure the intracellular dynamic assembly process. MCF-7 cells were then incubated with high concentration H_2_O_2_ (1 mM) for 2 h and cultured at normal condition for another 12 h. After the culture, MCF-7 cells were incubated with DCFH-DA for 20 min and washed before fluorescence microscopy. Fluorescent imaging of DCFH-DA was performed with 488 nm excitation and 510~540 nm emission.

### Western blot

Protein concentrations of samples were determined by BCA protein quantification kit. The samples with 30 μg protein were separated by sodium dodecyl sulfate-polyacrylamide gel electrophoresis (12% sodium dodecyl-sulfate polyacrylamide gel electrophoresis) and transferred onto polyvinylidene fluoride membranes. The membranes were washed three times with Tris-buffered saline containing 0.1% Tween-20 (TBST), blocked by 5% skimmed milk for 1 h, washed again with TBST, and finally incubated with anti-Bcl-2 (Abcam, ab219608, diluted to 1:2000) and anti-β-actin (Cell Signaling Technology, #4970, diluted to 1:1000) at 4 °C overnight. After washing with TBST, the membranes were incubated with horseradish peroxidase-labeled secondary antibody (Yeasen, 33201ES60, 33101ES60, diluted to 1:5000) at 37 °C for 1 h. The membranes were washed three times with TBST. The protein bands were detected using enhanced chemiluminescence reagent (Yeasen, 36208ES60*) and observed by using a gel image system.

### Flow cytometry

In the experiment of cell uptake, MCF-7 cells were seeded and incubated overnight. 100 μL of Cy5-labeled DCNC were added into each well and incubated with cells for 2, 4, 6, 8, and 12 h, respectively. After incubation, the supernatant was discarded, and MCF-7 cells were digested by trypsin-EDTA solution and terminated for digestion by adding DMEM-H medium. The cell suspension was washed twice with PBS, filtered with 70-μm sieves, and collected into flow tubes in dark.

In the experiment of ROS levels, MCF-7 cells were seeded and incubated overnight. PBS, D_ni_AS, DAS, D_ni_CNC, and DCNC were incubated with cells for 12 h, respectively. The medium was replaced with fresh DMEM-H, and the cells were cultured for another 24 h. The cells were treated with high concentration H_2_O_2_ (1 mM) for 2 h and then cultured at normal conditions for another 12 h. Cells were then collected and stained with DCFH-DA. The cell suspension was washed twice with PBS, filtered with 70 μm sieves, and collected into flow tubes in dark.

In the experiment of cell apoptosis, MCF-7 cells were seeded and incubated overnight. PBS, D_ni_AS, DAS, D_ni_CNC, and DCNC were incubated with cells for 12 h, respectively. The medium was replaced with fresh DMEM-H, and the cells were cultured for another 24 h. The cells were treated with high concentration H_2_O_2_ (1 mM) for 2 h and then cultured at normal conditions for another 12 h. The cells were then collected and stained with ANNEXIN V-FITC/PI. The cell suspension was filtered with 70-μm sieves and collected into flow tubes in dark.

Gating process was shown in Supplementary Fig. 16a. All data of flow cytometry was processed by FlowJo VX10.

### Intracellular GSH level

MCF-7 cells were seeded into the six-well plate (2 × 10^5^ cells per well), and cultured overnight. MCF-7 cells were first incubated with different materials (PBS, D_ni_AS, DAS, D_ni_CNC, and DCNC) for 12 h, ensuring that materials can enter cells sufficiently. The culture medium was changed into fresh one and cultured for another 24 h. MCF-7 cells were then incubated with high concentration H_2_O_2_ (1 mM) for 2 h and cultured at normal conditions for another 12 h. After the culture, the cells were collected to extract lysis. The concentration of GSH in lysis were measured by GSH kit purchased from Beyotime.

### Mitochondrial membrane potential (MMP) analysis

MMP was analyzed by CLSM using MMP assay kit (JC-10 kit). MCF-7 cells were seeded into the six-well plate (2 × 10^5^ cells per well), and cultured overnight. MCF-7 cells were first incubated with different materials (PBS, D_ni_AS, DAS, D_ni_CNC, and DCNC) for 12 h, ensuring that materials can enter cells sufficiently. The culture medium was changed into fresh one and cultured for another 24 h. MCF-7 cells were then incubated with high concentration H_2_O_2_ (1 mM) for 2 h and cultured at normal conditions for another 12 h. After the culture, MCF-7 cells were stained with JC-10 kit, and then stained with Hoechst 33342 (5 μg/mL) for another 15 min. Finally, the intracellular fluorescence was observed by CLSM.

### Cytotoxicity assay

MCF-7 cells were seeded into 96-well culture plates and cultured at 37 °C for 24 h. Then, cells were incubated with different materials (PBS, D_ni_AS, DAS, D_ni_CNC, and DCNC) for 24 h, respectively. Each well was washed with PBS, followed by the addition of 90 µL fresh medium and 10 µL CCK-8, and the plates were incubated for 4 h. The absorbance intensity of each well was measured in a microplate reader at 450 nm wavelength.

### F-actin staining

MCF-7 cells were seeded into the 6-well plate and cultured overnight. MCF-7 cells were first incubated with different materials (PBS, D_ni_AS, DAS, D_ni_CNC, and DCNC) for 12 h. Then the culture medium was changed into fresh one and cultured for another 24 h. MCF-7 cells were then incubated with high concentration H_2_O_2_ (1 mM) for 2 h and cultured at normal condition for another 12 h. After the culture, cells were washed three times with PBS, followed by fixation and permeabilization. F-actin was stained with FITC-labeled phalloidin. Finally, the intracellular fluorescence was observed by CLSM.

### Transwell migration assays

Transwell migration assays were performed at 37 °C using 24-well Transwell inserts (Corning). After incubation with PBS, D_ni_AS, DAS, D_ni_CNC, and DCNC for 12 h and then treated with 1 mM H_2_O_2_ for 2 h, MCF-7 cells (3 × 10^4^) suspended in 200 μL of serum-free medium were seeded into the upper chamber. 800 mL of migration inducer (DMEM-H with 10% FBS) was placed in the lower chamber. Cells were cultured for another 24 h. After the culture, the cells were stained by 1% crystal violet and observed by fluorescence microscope.

### Cell apoptosis assay

MCF-7 cells were first incubated with different materials (PBS, D_ni_AS, DAS, D_ni_CNC, and DCNC) for 12 h. Then the culture medium was changed into fresh one and cultured for another 24 h. MCF-7 cells were then incubated with high concentration H_2_O_2_ (1 mM) for 2 h and cultured for another 12 h. The cells were stained with Annexin V-FITC and PI. 1 mL of pre-cooled PBS was added and gently vibrated to suspend the cells. The suspension was then centrifuged at 156 × *g*, 4 °C for 5 min, and the supernatant was discarded. The washing procedure was repeated twice. The cells were resuspended in 200 μL binding buffer. 10 μL of Annexin V-FITC solution and 10 μL of PI solution were then added to cell suspension and incubated at room temperature for 15 min. Finally, 300 μL binding buffer was added and the samples were analyzed by flow cytometry.

### Calcein-AM/PI double-staining assay

Calcein-AM/PI staining reagents were applied to stain living cells as green fluorescence (*λ*_ex_ = 490 nm, *λ*_em_ = 515 nm) and dead cells as red fluorescence (*λ*_ex_ = 535 nm, *λ*_em_ = 617 nm). MCF-7 cells were first incubated with different materials (PBS, D_ni_AS, DAS, D_ni_CNC, and DCNC) for 12 h. Then the culture medium was changed into fresh one, and cultured for another 24 h. MCF-7 cells were then incubated with high concentration H_2_O_2_ (1 mM) for 2 h and cultured at normal condition for another 12 h. 5 μL of 4 mM calcein-AM solution and 5 μL of 16 mM PI solution were added to 10 mL PBS to dilute into 2 μM calcein-AM and 8 μM PI. After 30 min of incubation, staining solutions were removed and washed by PBS twice. The samples were then observed by fluorescence microscopy.

### Statistics and reproducibility

All data were reported as mean ± standard deviation (SD) from at least three independent runs. The two-tailed unpaired *t* test was used to assess the two-group differences. In all cases, a *P* value <0.05 was considered to be statistically significant. Analyses were performed using GraphPad Prism 8.

### Figure treatment

Microsoft PowerPoint 2019 was used to organize figures. ImageJ Fiji v1.52 v was used to process microscopy images.

### Reporting summary

Further information on research design is available in the Nature Portfolio Reporting Summary linked to this article.

## Supplementary information

Supplementary Information

Reporting Summary

## Data Availability

All data supporting this manuscript are contained within the main text and supplementary figures. Source data are provided with this paper.

## Source data

Source data

## References

[1] D’Autréaux B, Toledano MB (2007). ROS as signalling molecules: mechanisms that generate specificity in ROS homeostasis. Nat. Rev. Mol. Cell Biol..

[2] Yang B, Chen Y, Shi J (2019). Reactive oxygen species (ROS)-based nanomedicine. Chem. Rev..

[3] Trachootham D, Alexandre J, Huang P (2009). Targeting cancer cells by ROS-mediated mechanisms: a radical therapeutic approach?. Nat. Rev. Drug Discov..

[4] Liou G-Y, Storz P (2010). Reactive oxygen species in cancer. Free Radic. Res..

[5] Fraisl P, Aragonés J, Carmeliet P (2009). Inhibition of oxygen sensors as a therapeutic strategy for ischaemic and inflammatory disease. Nat. Rev. Drug Discov..

[6] Barnham KJ, Masters CL, Bush AI (2004). Neurodegenerative diseases and oxidative stress. Nat. Rev. Drug Discov..

[7] Sies H, Jones DP (2020). Reactive oxygen species (ROS) as pleiotropic physiological signalling agents. Nat. Rev. Mol. Cell Biol..

[8] Lodhi J, Semenkovich F (2014). Peroxisomes: a nexus for lipid metabolism and cellular signaling. Cell Metab..

[9] Sugiura A, Mattie S, Prudent J, McBride HM (2017). Newly born peroxisomes are a hybrid of mitochondrial and ER-derived pre-peroxisomes. Nature.

[10] Gorrini C, Harris IS, Mak TW (2013). Modulation of oxidative stress as an anticancer strategy. Nat. Rev. Drug Discov..

[11] Qin X (2021). Peroxisome inspired hybrid enzyme nanogels for chemodynamic and photodynamic therapy. Nat. Commun..

[12] Godoy-Gallardo M, York-Duran MJ, Hosta-Rigau L (2018). Recent progress in micro/nanoreactors toward the creation of artificial organelles. Adv. Healthc. Mater..

[13] Chen Y (2020). Artificial organelles based on cross-linked Zwitterionic vesicles. Nano Lett..

[14] Yang ZM, Xu KM, Guo ZF, Guo ZH, Xu B (2007). Intracellular enzymatic formation of nanofibers results in hydrogelation and regulated cell death. Adv. Mater..

[15] Einfalt T (2018). Biomimetic artificial organelles with in vitro and in vivo activity triggered by reduction in microenvironment. Nat. Commun..

[16] Tanner P, Balasubramanian V, Palivan CG (2013). Aiding nature’s organelles: artificial peroxisomes play their role. Nano Lett..

[17] Zhang D (2015). In situ formation of nanofibers from purpurin18-peptide conjugates and the assembly induced retention effect in tumor sites. Adv. Mater..

[18] Dahiya UR (2019). Role of cellular retention and intracellular state in controlling gene delivery efficiency of multiple nonviral carriers. ACS Omega.

[19] Liang G, Ren H, Rao J (2010). A biocompatible condensation reaction for controlled assembly of nanostructures in living cells. Nat. Chem..

[20] Gao J, Zhan J, Yang Z (2020). Enzyme-instructed self-assembly (EISA) and hydrogelation of peptides. Adv. Mater..

[21] Schoonen L, van Hest JCM (2016). Compartmentalization approaches in soft matter science: from nanoreactor development to organelle mimics. Adv. Mater..

[22] Yang D (2014). DNA materials: bridging nanotechnology and biotechnology. Acc. Chem. Res..

[23] Whitfield CJ (2021). Functional DNA–polymer conjugates. Chem. Rev..

[24] Xing Y (2011). Self-assembled DNA hydrogels with designable thermal and enzymatic responsiveness. Adv. Mater..

[25] Wu Y, Wang D, Willner I, Tian Y, Jiang L (2018). Smart DNA hydrogel integrated nanochannels with high ion flux and adjustable selective ionic transport. Angew. Chem. Int. Ed..

[26] Fu W (2019). Rational design of pH-responsive DNA motifs with general sequence compatibility. Angew. Chem. Int. Ed..

[27] Guo X (2020). Construction of organelle-like architecture by dynamic DNA assembly in living cells. Angew. Chem. Int. Ed..

[28] Yao C, Xu Y, Hu P, Ou J, Yang D (2021). Gene-like precise construction of functional DNA materials. Acc. Mater. Res..

[29] Pautler R (2013). Attaching DNA to nanoceria: regulating oxidase activity and fluorescence quenching. ACS Appl. Mater. Inter..

[30] Wang J (2022). Intracellular condensates of oligopeptide for targeting lysosome and addressing multiple drug resistance of cancer. Adv. Mater..

[31] Xu C (2013). Nanoceria-triggered synergetic drug release based on CeO_2_-capped mesoporous silica host–guest interactions and switchable enzymatic activity and cellular effects of CeO_2_. Adv. Healthc. Mater..

[32] Kwon HJ (2018). Ceria nanoparticle systems for selective scavenging of mitochondrial, intracellular, and extracellular reactive oxygen species in parkinson’s disease. Angew. Chem. Int. Ed..

[33] Weng Q (2021). Catalytic activity tunable ceria nanoparticles prevent chemotherapy-induced acute kidney injury without interference with chemotherapeutics. Nat. Commun..

[34] Perez JM, Asati A, Nath S, Kaittanis C (2008). Synthesis of biocompatible dextran-coated nanoceria with pH-dependent antioxidant properties. Small.

[35] Yao C (2018). Near-infrared upconversion mesoporous cerium oxide hollow biophotocatalyst for concurrent pH-/H_2_O_2_-responsive O_2_-evolving synergetic cancer therapy. Adv. Mater..

[36] Rennick JJ, Johnston APR, Parton RG (2021). Key principles and methods for studying the endocytosis of biological and nanoparticle therapeutics. Nat. Nanotechnol..

[37] Winterbourn CC (2008). Reconciling the chemistry and biology of reactive oxygen species. Nat. Chem. Biol..

[38] Mochizuki A (2018). Balanced regulation of redox status of intracellular thioredoxin revealed by in-cell NMR. J. Am. Chem. Soc..

[39] Zhu D (2005). Hydrogen peroxide alters membrane and cytoskeleton properties and increases intercellular connections in astrocytes. J. Cell Sci..

