# Supplementary material for: Dynamic assembly of DNA-ceria nanocomplex in living cells generates artificial peroxisome
Source: Nat Commun. 2022 Dec 14;13:7739. doi: 10.1038/s41467-022-35472-2 (PMC9751304; doi:10.1038/s41467-022-35472-2)
Supplement: Supplementary file 1 — Supplementary Information [file 41467_2022_35472_MOESM1_ESM.pdf]

Supplementary Information

Dynamic assembly of DNA-ceria nanocomplex in living cells generates artificial peroxisome

Chi Yao[1,2,#,*], Yuwei Xu[1,#], Jianpu Tang[1,#], Pin Hu[1], Hedong Qi[1], Dayong Yang[1,2,*]

[1] Frontiers Science Center for Synthetic Biology, Key Laboratory of Systems Bioengineering (MOE), Institute of Biomolecular and Biomedical Engineering, School of Chemical Engineering and Technology, Tianjin University, Tianjin, 300350, P.R. China.

[2] Zhejiang Institute of Tianjin University, Ningbo, Zhejiang, 315200, P.R. China.

*Corresponding authors: dayong.yang@tju.edu.cn; chi.yao@tju.edu.cn

#These authors contributed equally to this work.

Keywords: DNA nanotechnology, artificial organelle, i-motif DNA, reactive oxygen species

Contents:

Supplementary Figures 1-32

Supplementary Table 1

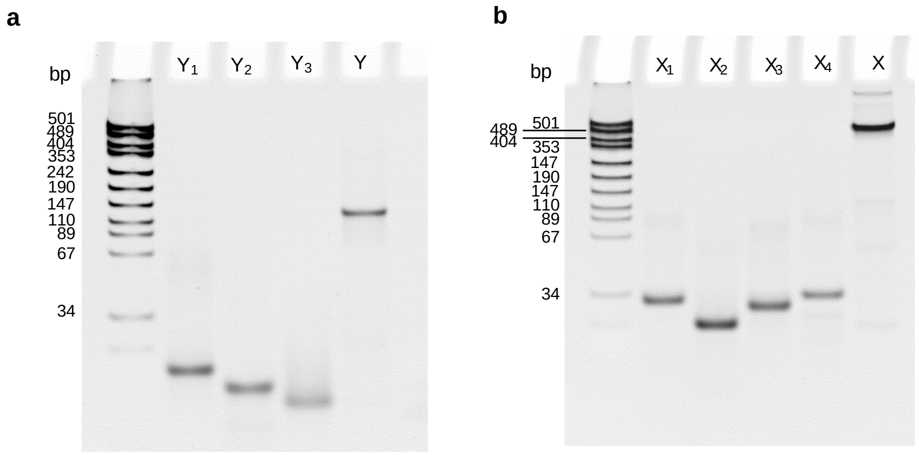

Supplementary Fig. 1. Polyacrylamide gel electrophoresis (12%) to verify the synthesis of Y-shaped DNA (**a**) and X-shaped DNA (**b**).

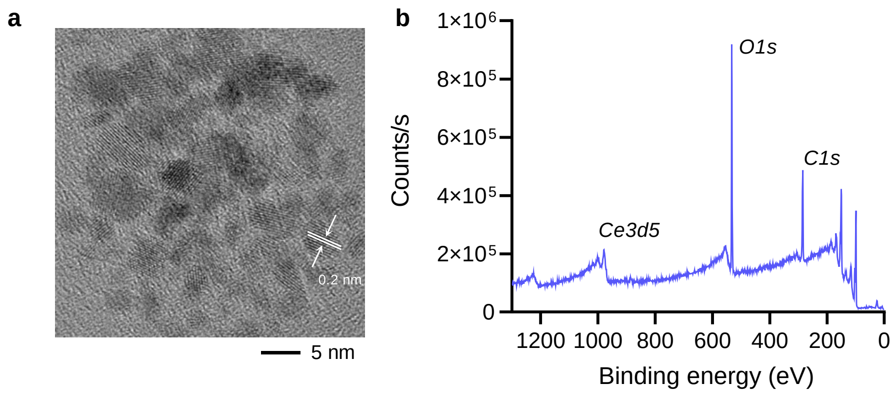

Supplementary Fig. 2. Characterization of ceria. a, Representative transmission electron microscopy image of ceria. **b,** X-ray photoelectron spectroscopy analysis of ceria.

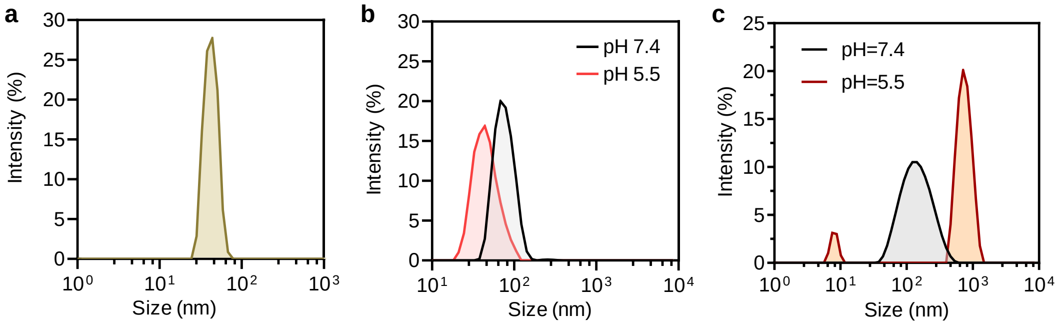

Supplementary Fig. 3. Dynamic light scattering (DLS) analysis. a, DLS analysis of DAS at pH 7.4. **b,** DLS analysis of D_{ni}CNC at pH 7.4 and pH 5.5, respectively. **c,** DLS analysis of DCNC in cell culture media at pH 7.4 and pH 5.5, respectively.

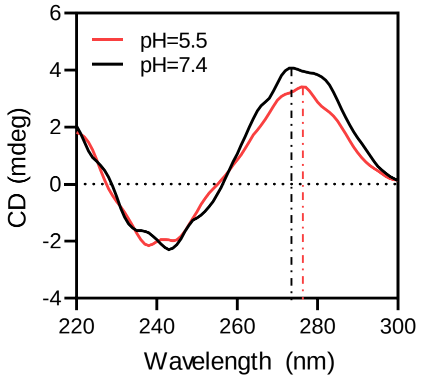

Supplementary Fig. 4. Circular dichroism (CD) spectra of DCNC at pH 7.4 and pH 5.5, respectively.

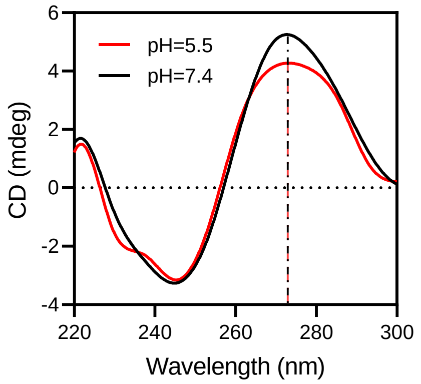

Supplementary Fig. 5. CD spectra of D_{ni}CNC at pH 7.4 and pH 5.5, respectively.

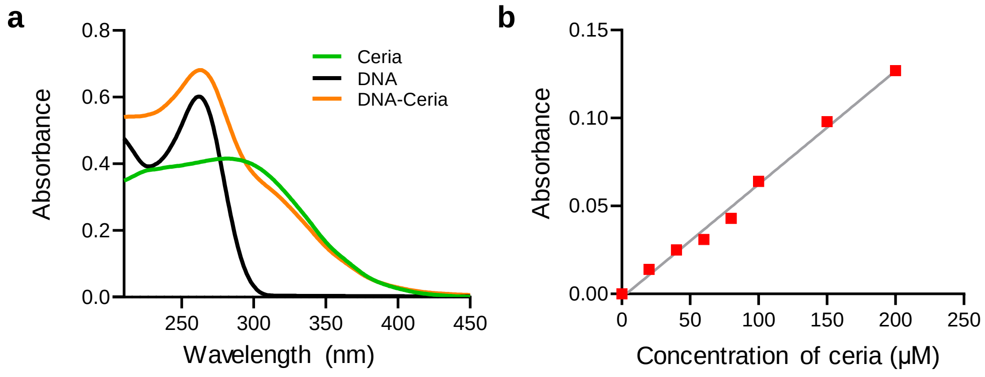

Supplementary Fig. 6. Calibration curve of absorbance *vs.* concentration of ceria. a, Absorbance spectra of ceria, DNA and DNA-ceria. **b,** The calibration curve of absorbance *vs.* concentration of ceria.

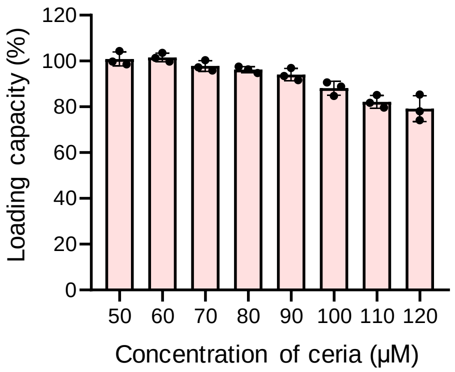

Supplementary Fig. 7. Loading capacity of DCNC toward ceria at different concentration of ceria. Bars represent mean ± SD (n=3 independent samples).

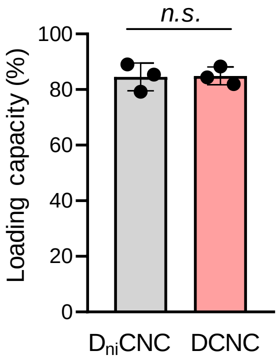

Supplementary Fig. 8. Loading capacity of D_{ni}CNC and DCNC towards ceria at 100 μM. Bars represent mean ± SD (n=3 independent samples). (*n.s.*, no significance, P>0.05, calculated by two-tailed unpaired t test).

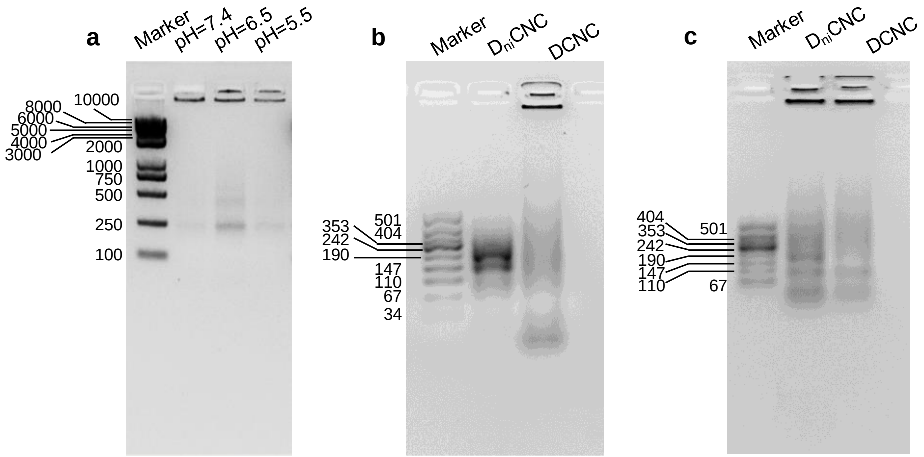

Supplementary Fig. 9. Agarose gel electrophoresis for verifying the dissociation and reassembly of materials. a, DCNCs were incubated in pH 7.4, 6.5 and 5.5 buffer for 4 h, respectively. **b,** D_{ni}CNC and DCNC were separately incubated in pH 5.5 buffer for 4 h. **c,** D_{ni}CNC and DCNC were separately incubated in pH 5.5 buffer and then incubated in pH 7.4 buffer.

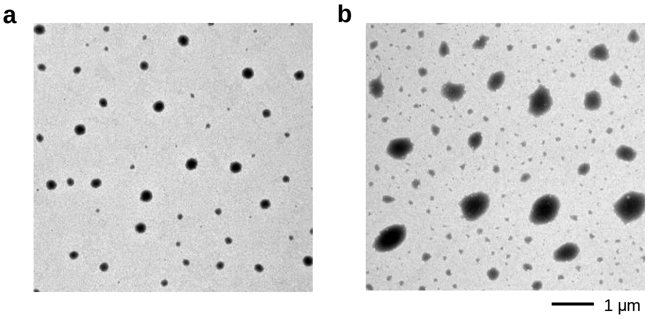

Supplementary Fig. 10. Representative transmission electron microscopy images of the DCNC at pH 7.4 (**a**) and the DCNC incubated from pH 5.5 to pH 7.4 (**b**).

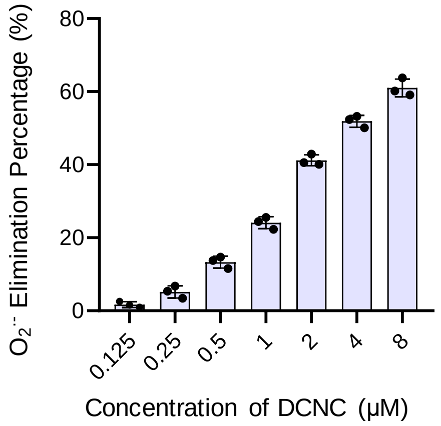

Supplementary Fig. 11. $O_2^{\cdot-}$ elimination percentages under the different concentrations of DCNC at pH 7.4. Bars represent mean ± SD (n=3 independent samples).

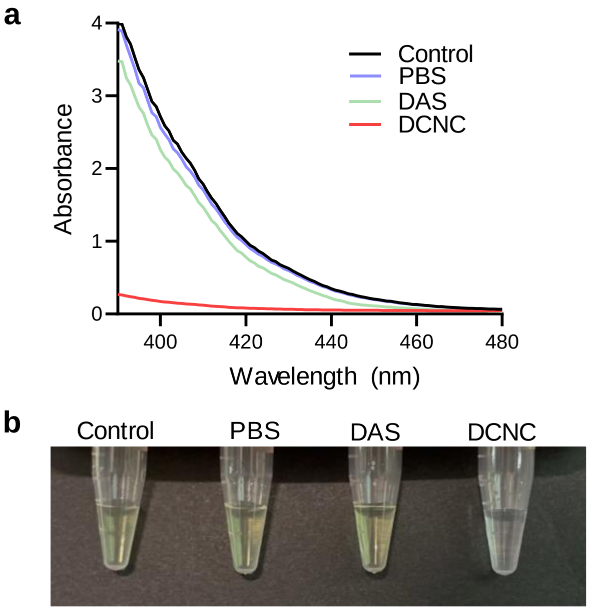

Supplementary Fig. 12. Test of catalase-like catalytic ability. a, Absorbance spectra of ammonium molybdate (AMT) after incubation with water (control), PBS, DAS and DCNC for 30 min, respectively. **b,** Photographs of AMT after incubation with water (control), PBS, DAS and DCNC for 30 min, respectively.

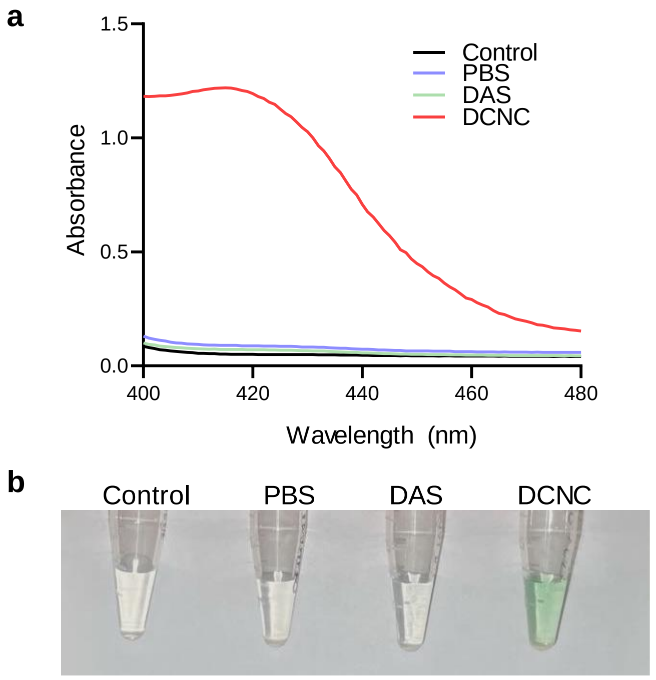

Supplementary Fig. 13. Test of peroxidase-like catalytic ability. a, Absorption spectra of ABTS after incubation with PBS, DAS and DCNC for 30 min, respectively. **b,** Photographs of ABTS after incubation with water (control), PBS, DAS and DCNC for 30 min, respectively.

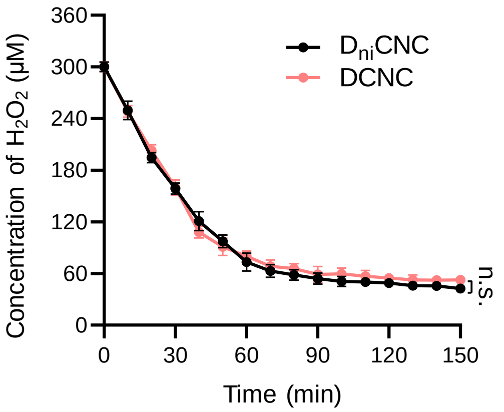

Supplementary Fig. 14. Decomposition of H_2O_2 at different time points catalyzed by D_{ni}CNC and DCNC, respectively. Bars represent mean ± SD (n=3). (*n.s.*, no significance, P>0.05, calculated by two-tailed unpaired t test).

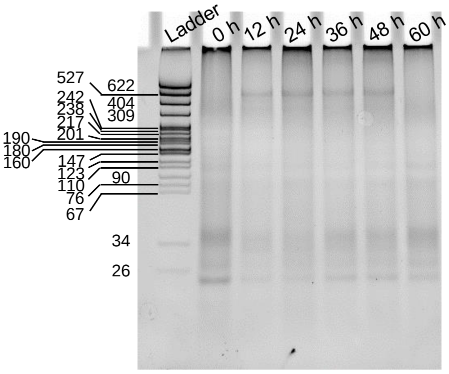

Supplementary Fig. 15. Polyacrylamide gel electrophoresis to analyze the stability of DCNC after incubation with 10% FBS for different time durations.

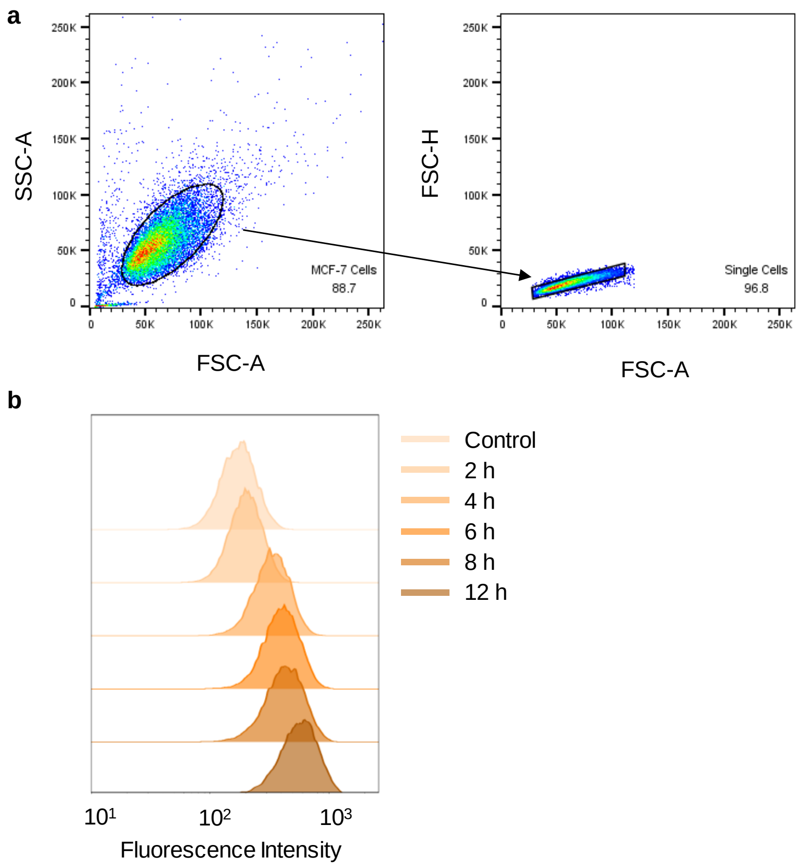

Supplementary Fig. 16. Flow cytometry to analyze the uptake of DCNC by MCF-7 cells with time. a, Front/side/fluorescence scatter plots showing gating process. **b,** Fluorescence intensity of MCF-7 cells incubated with Cy5-labelled DCNC for different time durations.

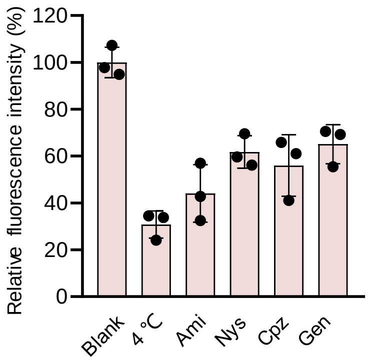

Supplementary Fig. 17. Quantitative statistics of the red fluorescence intensity of DCNC in the MCF-7 cells with different treatments, respectively. Ami, amiloride; Nys, nystatin; Cpz, chlorpromazine; Gen, genistein. Bars represent mean ± SD (n=3 independent samples).

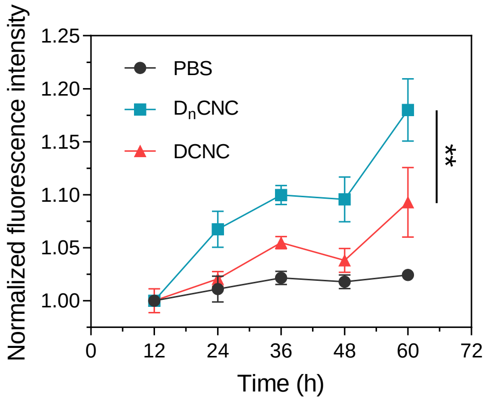

Supplementary Fig. 18. Escape kinetics of D_{ni}CNC and DCNC from MCF-7 cells, respectively. Bars represent mean ± SD (n=3 independent samples). (**, P<0.01, calculated by two-tailed unpaired t test).

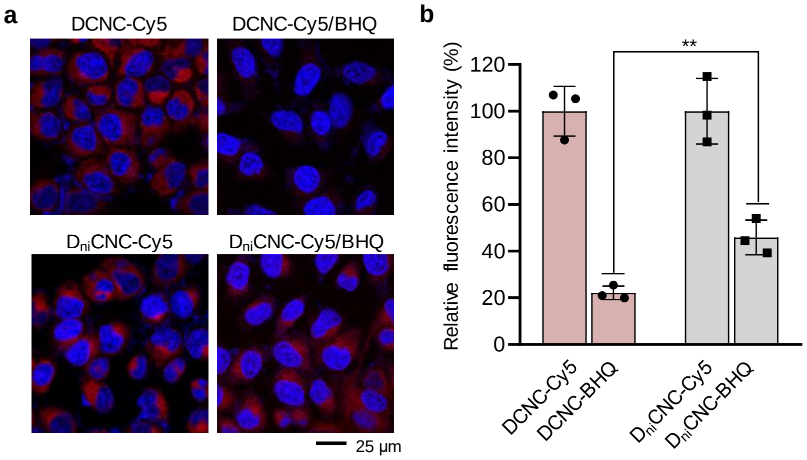

Supplementary Fig. 19. Aggregative processes of DCNC in MCF-7 cells verified by FRET. a, Representative fluorescence images of MCF-7 cells after incubation with DCNC and D_{ni}CNC for 12 h, respectively. DCNC-Cy5: adding 100% Cy5-labelled DCNC; DCNC-Cy5/BHQ: adding 50% Cy5-labelled DCNC and 50% BHQ-labelled DCNC; D_{ni}CNC-Cy5: adding 100% Cy5-labelled D_{ni}CNC; D_{ni}CNC-Cy5/BHQ: adding 50% Cy5-labelled D_{ni}CNC and 50% BHQ-labelled D_{ni}CNC. **b,** Quantitative statistics of the red fluorescence intensity in **a**. Bars represent mean ± SD (n=3 independent samples). (**, P<0.01, calculated by two-tailed unpaired t test). BHQ, black hole quencher.

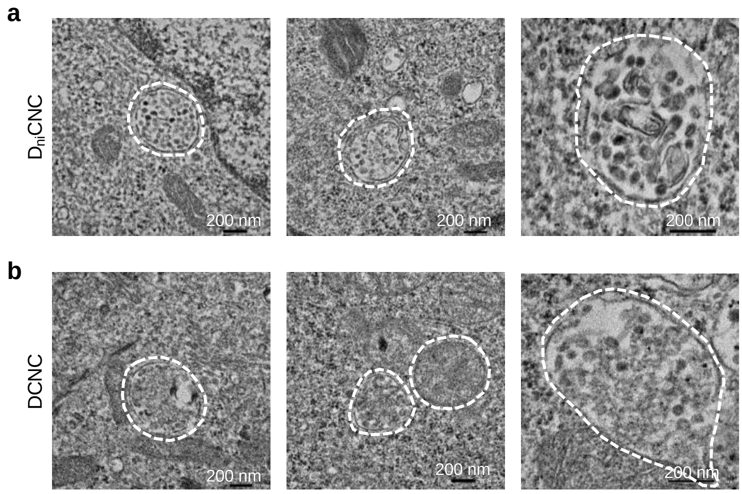

Supplementary Fig. 20. TEM images of MCF-7 cells that were treated with D_{ni}CNC (**a**) and DCNC (**b**) for 6 h, respectively. The white circles indicated lysosomes.

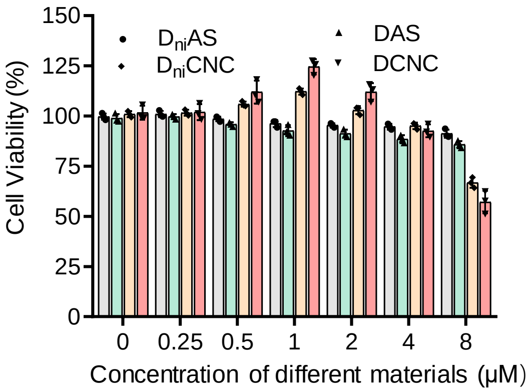

Supplementary Fig. 21. Cell viability of MCF-7 cells after incubation with different concentrations of materials for 24 h, respectively. Bars represent mean ± SD (n=3 independent samples).

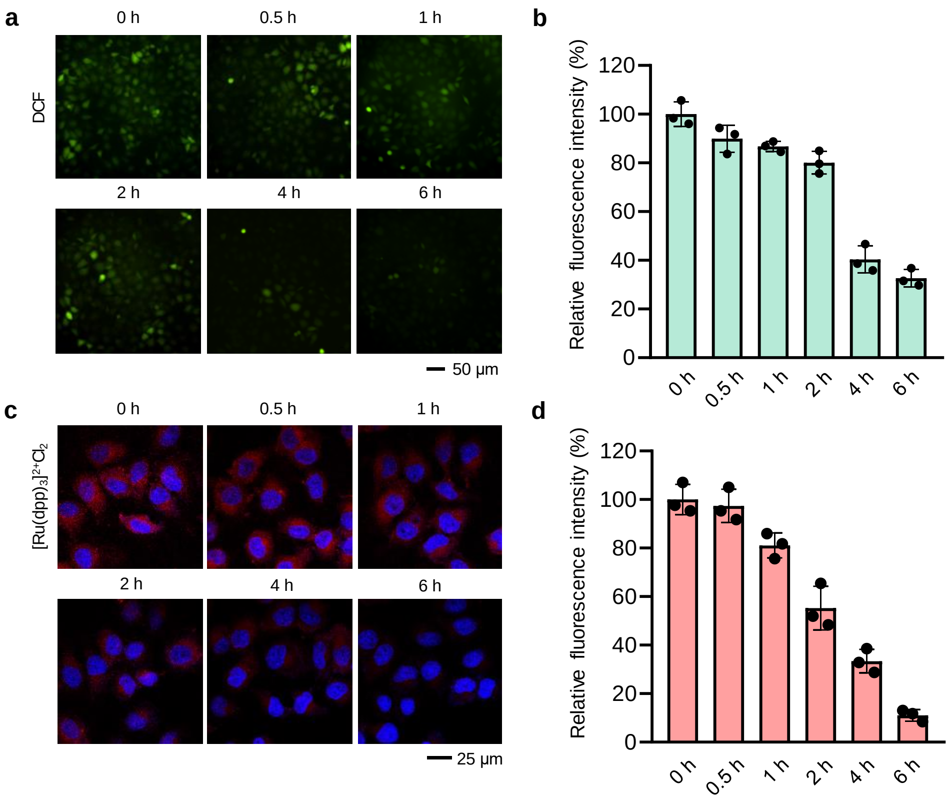

Supplementary Fig. 22. Intracellular effects of DCNC. a, Representative fluorescence microscopy images of intracellular ROS level in MCF-7 cells incubated with DCNC for varied time durations. **b,** Quantitative statistics of fluorescence intensity in **a**. Bars represent mean ± SD (n=3 independent samples). **c,** Representative confocal microscopy images of intracellular O_2 generation in MCF-7 cells detected by $[Ru(dpp)_3]^{2+}Cl_2$. **d,** Quantitative statistics of the red fluorescence intensity in **c**. Bars represent mean ± SD (n=3 independent samples).

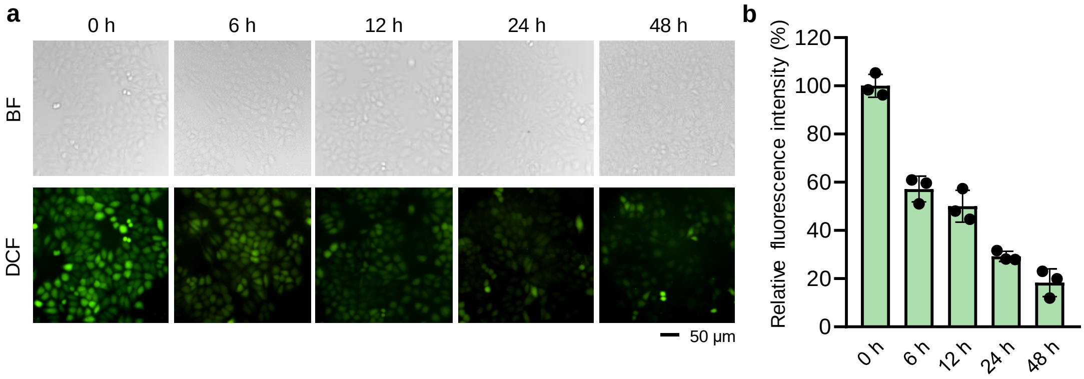

Supplementary Fig. 23. Intracellular ROS levels measured by DCFH-DA fluorescent probe. **a,** Representative fluorescence microscopy images of MCF-7 cells treated with 1 mM H_2O_2 and then incubated with DCNC for different time durations. **b,** Quantitative statistics of fluorescence intensity in **a**. Bars represent mean ± SD (n=3 independent samples).

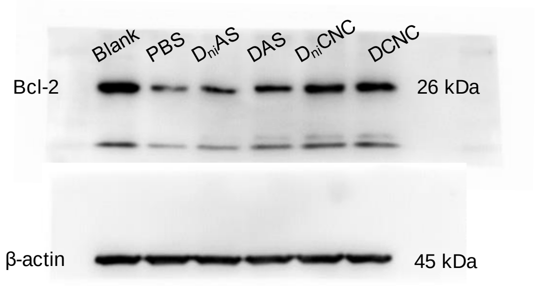

Supplementary Fig. 24. Expression level of Bcl-2 analyzed by western blot. β-actin was set as the control.

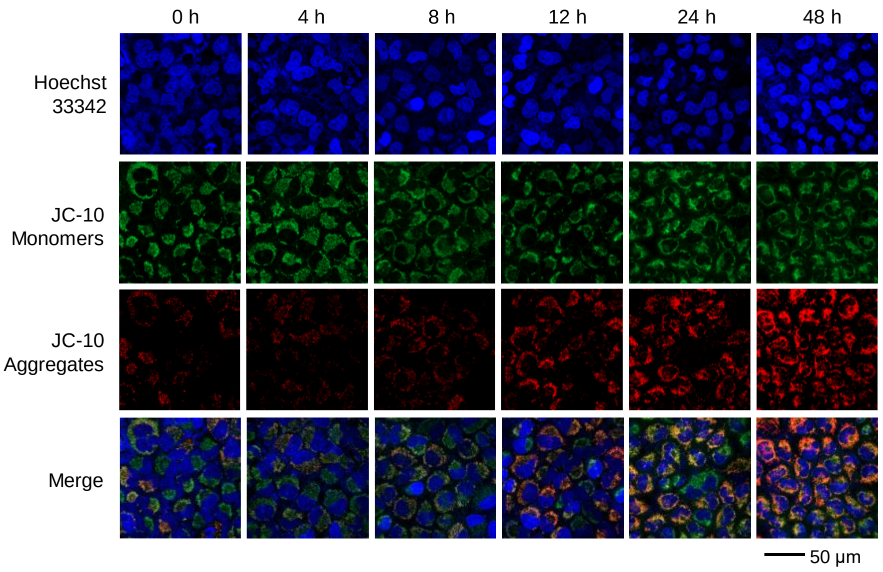

Supplementary Fig. 25. Representative confocal microscopy images of JC-10 staining in the MCF-7 cells treated with 1 mM H_2O_2 and then incubated with DCNC for different time durations.

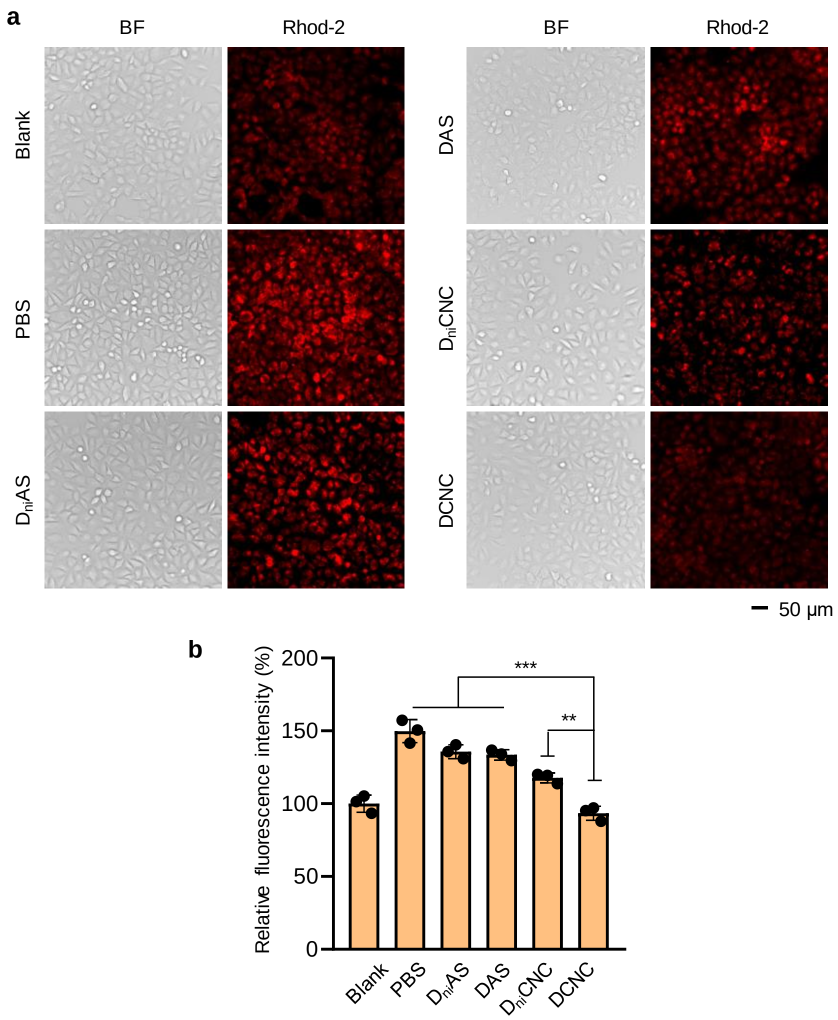

Supplementary Fig. 26. Intracellular Ca^{2+} level in the MCF-7 cells incubated with different materials. a, Representative fluorescence microscopy images of MCF-7 cells incubated with different materials, respectively, and then stained by Rhod-2. **b,** Quantitative statistics of fluorescence intensity in **a**. Bars represent mean ± SD (n=3 independent samples). (**, P<0.01; ***, P<0.001, calculated by two-tailed unpaired t test).

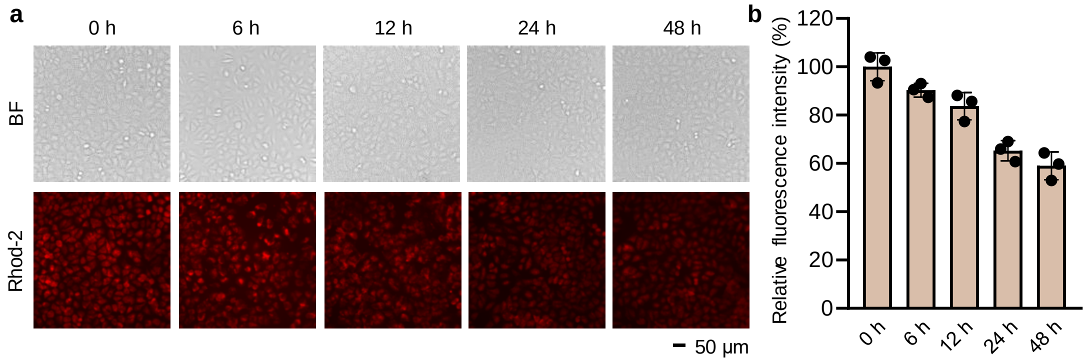

Supplementary Fig. 27. Intracellular Ca^{2+} level in the MCF-7 cells incubated with DCNC for different time durations. a, Representative fluorescence microscopy images of MCF-7 cells incubated with DCNC for different time durations and then stained by Rhod-2. **b,** Quantitative statistics of fluorescence intensity in **a**. Bars represent mean ± SD (n=3 independent samples).

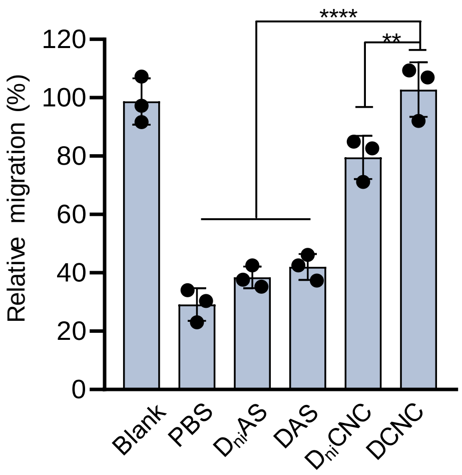

Supplementary Fig. 28. Quantitative statistics of the migrating MCF-7 cells treated with different materials in a Transwell cell invasion device. Bars represent mean ± SD (n=3 independent samples). (**, P<0.01; ****, P<0.0001, calculated by two-tailed unpaired t test).

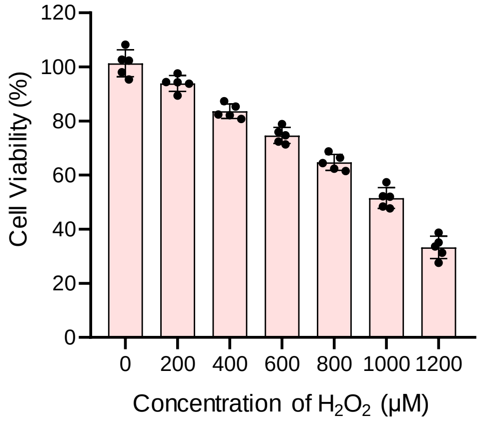

Supplementary Fig. 29. Cell viability of MCF-7 cells after incubation with different concentrations of H_2O_2 for 2 h. Bars represent mean ± SD (n=3 independent samples).

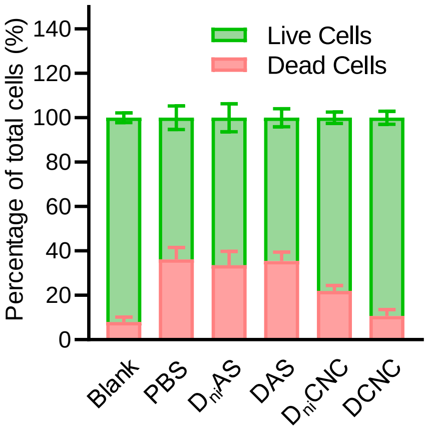

Supplementary Fig. 30. Statistical analysis of the live and dead MCF-7 cells after incubation with different materials, respectively. Bars represent mean ± SD (n=3 independent samples).

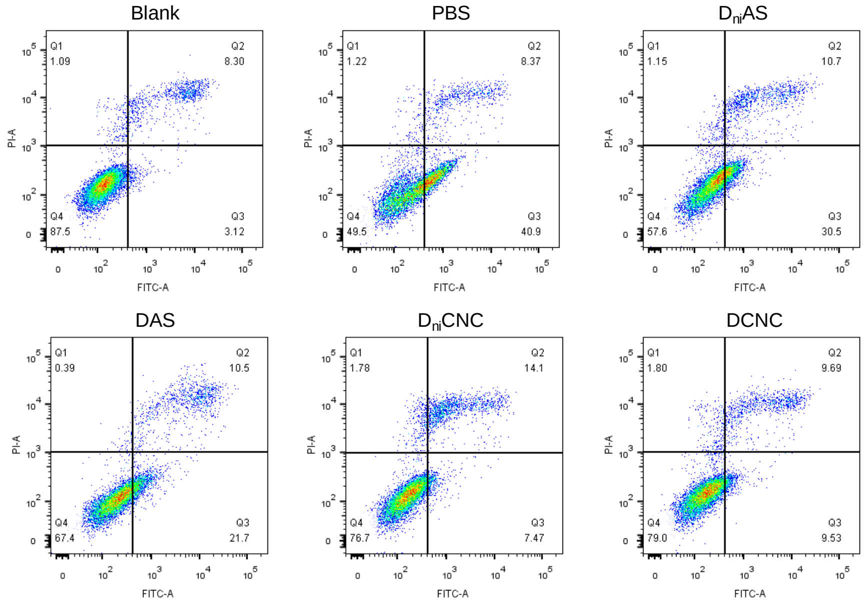

Supplementary Fig. 31. Flow cytometry analysis of cell apoptosis. MCF-7 cells were stained with Annexin V-FITC/PI after incubation with different materials.

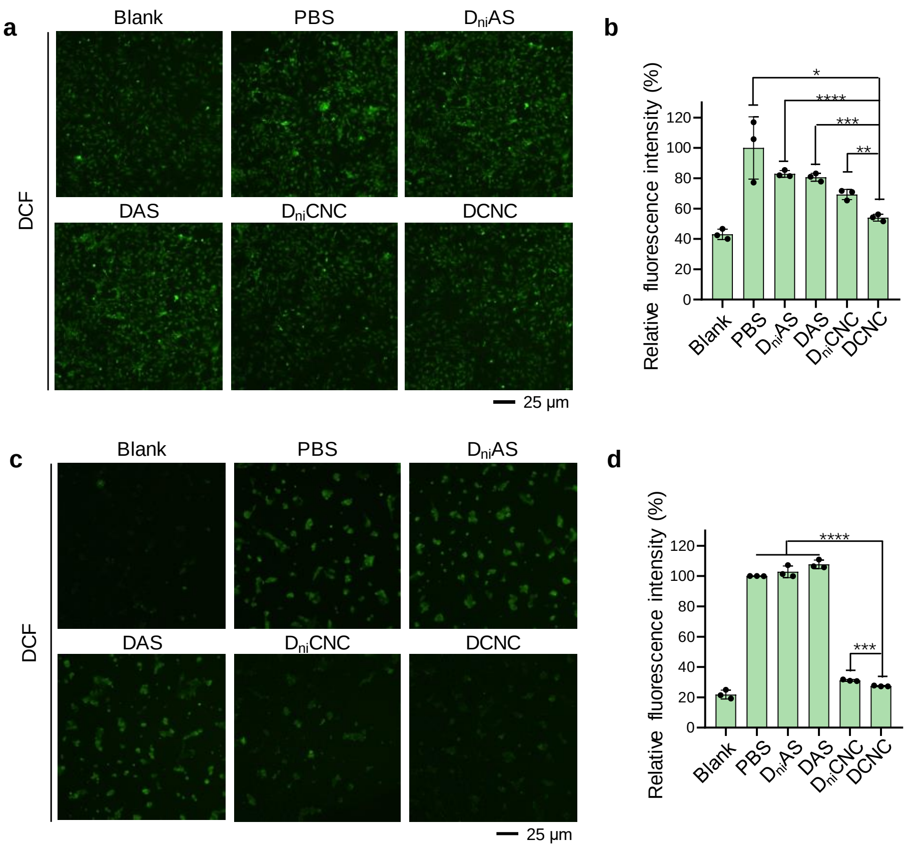

Supplementary Fig. 32. ROS regulation effects of DCNC in H9C2 cells and BEAS-2B cells. **a,** Representative fluorescence microscopy images showing the ROS levels in H9C2 cells measured with DCFH-DA fluorescent probe after incubation with different materials (PBS, D_{ni}AS, DAS, D_{ni}CNC and DCNC, respectively) and treatment of H_2O_2. The green signal was from DCF. **b,** Mean fluorescence intensity of DCF in (**a**). Bars represent mean ± SD (n=3 independent samples). (*, P<0.05; **, P<0.01; ***, P<0.001; ****, P<0.0001, calculated by two-tailed unpaired t test). **c,** Representative fluorescence microscopy images showing the ROS levels in BEAS-2B cells measured with DCFH-DA fluorescent probe after incubation with different materials (PBS, D_{ni}AS, DAS, D_{ni}CNC and DCNC, respectively) and treatment of H_2O_2. **d,** Mean fluorescence intensity of DCF in (**c**). Bars represent mean ± SD (n=3 independent samples). (***, P<0.001; ****, P<0.0001, calculated by two-tailed unpaired t test).

Supplementary Table 1. Oligonucleotide sequences used in this study.

Name	Sequence (5' to 3')	Notes
X-1	5'-CCTCCTCTCTCC GCGTGGATAGTAGACTCGCACAATTGCATC-3'	Synthesis of X-shaped DNA
X-2	5'-CCTCCTCTCTCC GATGCAATTGTGCGACAGGTACATTTGCTC-3'	
X-3	5'-CCTCCTCTCTCC GAGCAAATGTACCTGCAGCAGGAGTCAGTG-3'	
X-4	5'-CCTCCTCTCTCC CACTGACTCCTGCTGGTCTACTATCCACGC-3'	
$Y_{i\text{-motif}}$-1	5'-GGAGAGAGGAGG CTGGTTCACGACTCCATGACCGACGAAG-3'	Synthesis of Y_i-shaped DNA
$Y_{i\text{-motif}}$-2	5'-GGAGAGAGGAGG CTTCGTCGGTCATGTGGATCCTGACTCG-3'	
$Y_{i\text{-motif}}$-3	5'-CCCCTAACCCC CGAGTCAGGATCCAGAGTCGTGAACCAG-3'	
Y_{ni}-1	5'-GGAGAGAGGAGG CTGGTTCACGACTCCATGACCGACGAAG-3'	Synthesis of Y_{ni}-shaped DNA
Y_{ni}-2	5'-GGAGAGAGGAGG CTTCGTCGGTCATGTGGATCCTGACTCG-3'	
Y_{ni}-3	5'-AAAAAAAAAAA CGAGTCAGGATCCAGAGTCGTGAACCAG-3'	

Green letters: Sticky ends of X-shaped DNA, complementary to the sticky ends of Y-shaped DNA (Blue letters); Blue letters: Sticky ends of Y-shaped DNA, complementary to the sticky ends of X-shaped DNA (Green letters); Red letters: i-motif sequence of Y-shaped DNA.
