# Supplementary material for: Dynamic assembly of DNA-ceria nanocomplex in living cells generates artificial peroxisome
Source: Nat Commun. 2022 Dec 14;13:7739. doi: 10.1038/s41467-022-35472-2 (PMC9751304; doi:10.1038/s41467-022-35472-2)
Supplement: Supplementary file 2 — Reporting Summary [file 41467_2022_35472_MOESM2_ESM.pdf]

nature portfolio

Corresponding author(s): Dayong Yang

Last updated by author(s): Dec 4, 2022

Reporting Summary

Nature Portfolio wishes to improve the reproducibility of the work that we publish. This form provides structure for consistency and transparency in reporting. For further information on Nature Portfolio policies, see our Editorial Policies and the Editorial Policy Checklist.

Statistics

For all statistical analyses, confirm that the following items are present in the figure legend, table legend, main text, or Methods section.

n/a	Confirmed	
☐	☒	The exact sample size (*n*) for each experimental group/condition, given as a discrete number and unit of measurement
☐	☒	A statement on whether measurements were taken from distinct samples or whether the same sample was measured repeatedly
☐	☒	The statistical test(s) used AND whether they are one- or two-sided<br>*Only common tests should be described solely by name; describe more complex techniques in the Methods section.*
☐	☒	A description of all covariates tested
☐	☒	A description of any assumptions or corrections, such as tests of normality and adjustment for multiple comparisons
☐	☒	A full description of the statistical parameters including central tendency (e.g. means) or other basic estimates (e.g. regression coefficient) AND variation (e.g. standard deviation) or associated estimates of uncertainty (e.g. confidence intervals)
☐	☒	For null hypothesis testing, the test statistic (e.g. *F*, *t*, *r*) with confidence intervals, effect sizes, degrees of freedom and *P* value noted<br>*Give P values as exact values whenever suitable.*
☒	☐	For Bayesian analysis, information on the choice of priors and Markov chain Monte Carlo settings
☒	☐	For hierarchical and complex designs, identification of the appropriate level for tests and full reporting of outcomes
☐	☒	Estimates of effect sizes (e.g. Cohen's *d*, Pearson's *r*), indicating how they were calculated

Our web collection on statistics for biologists contains articles on many of the points above.

Software and code

Policy information about availability of computer code

Data collection	Microsoft PowerPoint 2019, Adobe Illustrator 2021
Data analysis	ImageJ Fiji v1.52v, FlowJo VX10, GraphPad Prism 8, Microsoft Excel 2019

For manuscripts utilizing custom algorithms or software that are central to the research but not yet described in published literature, software must be made available to editors and reviewers. We strongly encourage code deposition in a community repository (e.g. GitHub). See the Nature Portfolio guidelines for submitting code & software for further information.

Data

Policy information about availability of data

All manuscripts must include a data availability statement. This statement should provide the following information, where applicable:

- Accession codes, unique identifiers, or web links for publicly available datasets
- A description of any restrictions on data availability
- For clinical datasets or third party data, please ensure that the statement adheres to our policy

All data supporting this manuscript are contained within the main text and supplementary figures. Source data are provided with this paper (including data presented in the main text and in the Supplementary Information).

Field-specific reporting

Please select the one below that is the best fit for your research. If you are not sure, read the appropriate sections before making your selection.

- [x] Life sciences
- [ ] Behavioural & social sciences
- [ ] Ecological, evolutionary & environmental sciences

For a reference copy of the document with all sections, see nature.com/documents/nr-reporting-summary-flat.pdf

Life sciences study design

All studies must disclose on these points even when the disclosure is negative.

Sample size	For in vitro experiments, three independent samples were included in each technical replicate. We did not perform power analysis prior performing these experiments. We chose these sample sizes based on a pilot experiment using three/five samples in each group to compare the effect. The experiments demonstrated that three/five samples were sufficient to detect a significant difference (p<0.05) between the treatment group and control group.
Data exclusions	No data was excluded from the analysis.
Replication	All in vitro cellular experiments were replicated independently for at least 3 times. All attempts at replication were successful.
Randomization	Samples were allocated to plate locations randomly to minimise the impact of edge-effects on results.
Blinding	The person who executed the in vitro experiments was blinded to another person who carried out measurement.

Reporting for specific materials, systems and methods

We require information from authors about some types of materials, experimental systems and methods used in many studies. Here, indicate whether each material, system or method listed is relevant to your study. If you are not sure if a list item applies to your research, read the appropriate section before selecting a response.

Materials & experimental systems

n/a	Involved in the study	
☐	☒	Antibodies
☐	☒	Eukaryotic cell lines
☒	☐	Palaeontology and archaeology
☒	☐	Animals and other organisms
☒	☐	Human research participants
☒	☐	Clinical data
☒	☐	Dual use research of concern

Methods

n/a	Involved in the study	
☒	☐	ChIP-seq
☐	☒	Flow cytometry
☒	☐	MRI-based neuroimaging

Antibodies

Antibodies used	All antibodies used in the figures are listed in the Methods with supplier name and catalog number.<br>Primary antibodies: Rabbit monoclonal [EPR17509] to Bcl-2 (Abcam, ab219608), dilution 1:2000; β-Actin (13E5) Rabbit mAb (Cell Signaling Technology, #4970), dilution 1:1000.
Validation	According to the manufacturer, there have been more than 200 citations for Rabbit monoclonal [EPR17509] to Bcl-2 (Abcam, ab219608), and more than 2000 citations for β-Actin (13E5) Rabbit mAb (Cell Signaling Technology, #4970). Western blot is carried out for the validation of both rabbit monoclonal [EPR17509] to Bcl-2 and β-Actin (13E5) Rabbit mAb.

Eukaryotic cell lines

Policy information about cell lines

Cell line source(s)	The cell lines MCF-7, H9C2 and BEAS-2B used in this work was purchased from the Shanghai Institute of Biochemistry and Cell Biology, CAS, China.
Authentication	Authentication of the cell lines by the vendors was confirmed vis STR prior to their purchase and use.
Mycoplasma contamination	The cell lines were tested with Universal Mycoplasma Detection Kit (ATCC #30-1012K) and confirmed free of mycoplasma contamination.

Commonly misidentified lines (See ICLAC register)	No commonly misidentified cell lines were used in the study.

Flow Cytometry

Plots

Confirm that:

- [x] The axis labels state the marker and fluorochrome used (e.g. CD4-FITC).
- [x] The axis scales are clearly visible. Include numbers along axes only for bottom left plot of group (a 'group' is an analysis of identical markers).
- [ ] All plots are contour plots with outliers or pseudocolor plots.
- [ ] A numerical value for number of cells or percentage (with statistics) is provided.

Methodology

Sample preparation	For the cell uptake measured by flow cytometry, MCF-7 cells were first seeded and incubated overnight. 100 μL of Cy5-labeled DCNC were added into each well and incubated with cells for indicated times. The incubation time was set as 2, 4, 6, 8 and 12 h. After incubation, the supernatant was discarded, and MCF-7 cells were digested by trypsin-EDTA solution and terminated for digestion by adding DMEM-H medium. Then, the cell suspension was washed twice with PBS. Next, MCF-7 cells were filtered with 70 μm sieves and collected into flow tubes in dark.<br>For the ROS levels measured by flow cytometry, MCF-7 cells were first seeded and incubated overnight. PBS, DniAS, DAS, DniCNC, DCNC were incubated with cells for 12 h. Then the medium was replaced with fresh DMEM-H and cultured for another 24 h. The cells were treated with high concentration H2O2 (1 mM) for 2 h and then cultured at normal conditions for another 12 h. Cells were then collected and stained with DCFH-DA. Then, the cell suspension was washed twice with PBS. Next, MCF-7 cells were filtered with 70 μm sieves and collected into flow tubes in dark.<br>For the cell apoptosis measured by flow cytometry, MCF-7 cells were first seeded and incubated overnight. PBS, DniAS, DAS, DniCNC, DCNC were incubated with cells for 12 h. Then the medium was replaced with fresh DMEM-H and cultured for another 24 h. The cells were treated with high concentration H2O2 (1 mM) for 2 h and then cultured at normal conditions for another 12 h. Cells were then collected and stained with ANNEXIN V-FITC/PI. Next, MCF-7 cells were filtered with 70 μm sieves and collected into flow tubes in dark.
Instrument	BD Flow Cytometer (FACSAria III)
Software	BD FACSAria III Software
Cell population abundance	Population abundance was determined by collecting a fixed (20000) cell number for each sample.
Gating strategy	The gating strategy has been described in Supplementary information.

- [x] Tick this box to confirm that a figure exemplifying the gating strategy is provided in the Supplementary Information.
