# Supplementary material for: Dynamic assembly of DNA-ceria nanocomplex in living cells generates artificial peroxisome
Source: Nat Commun. 2022 Dec 14;13:7739. doi: 10.1038/s41467-022-35472-2 (PMC9751304; doi:10.1038/s41467-022-35472-2)
Supplement: Supplementary file 3 — Source data [file 41467_2022_35472_MOESM3_ESM.zip › Blot and gel images.pptx]

## Slide 1
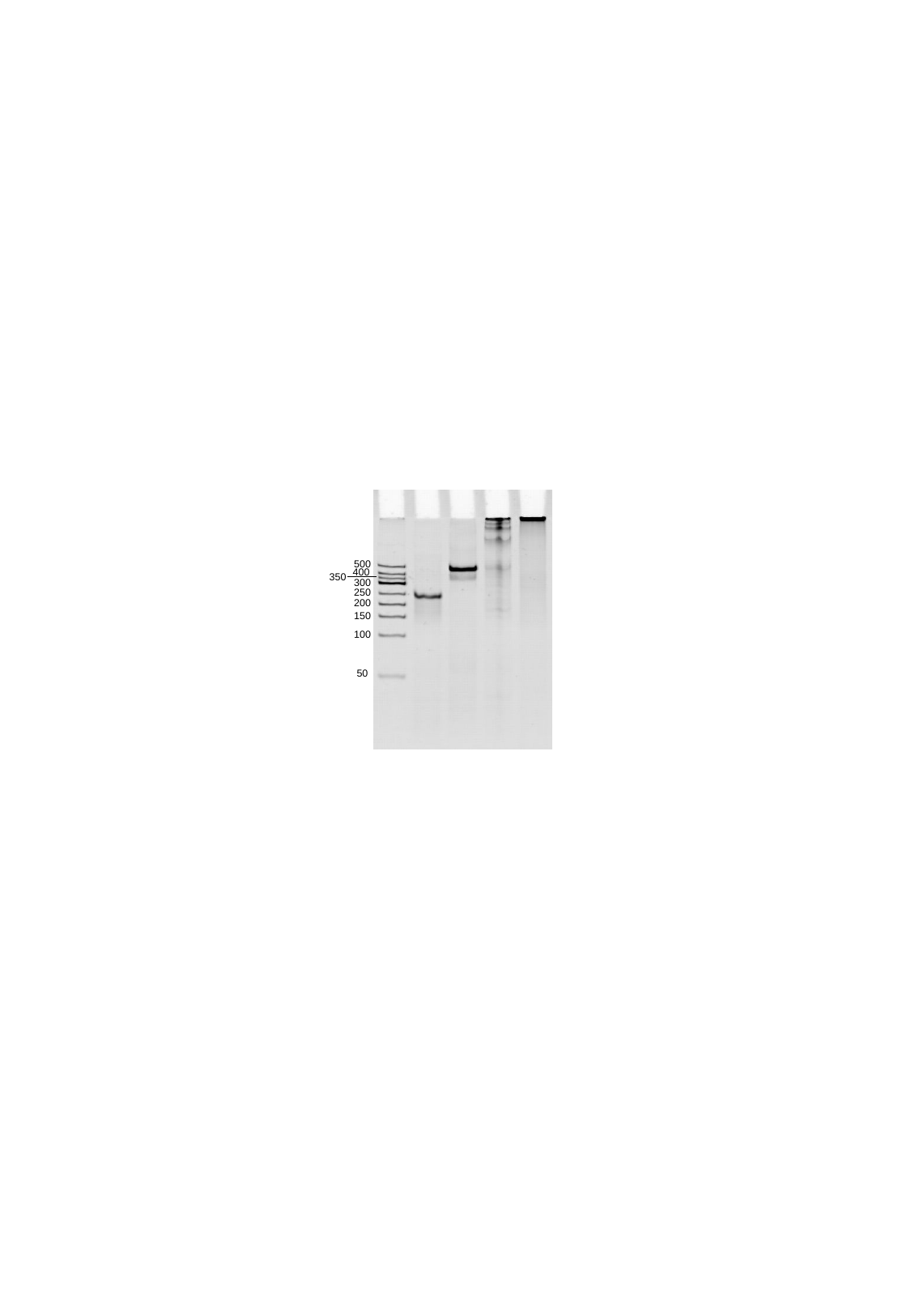

500
400
350
300
250
200
150
100
50

## Slide 2
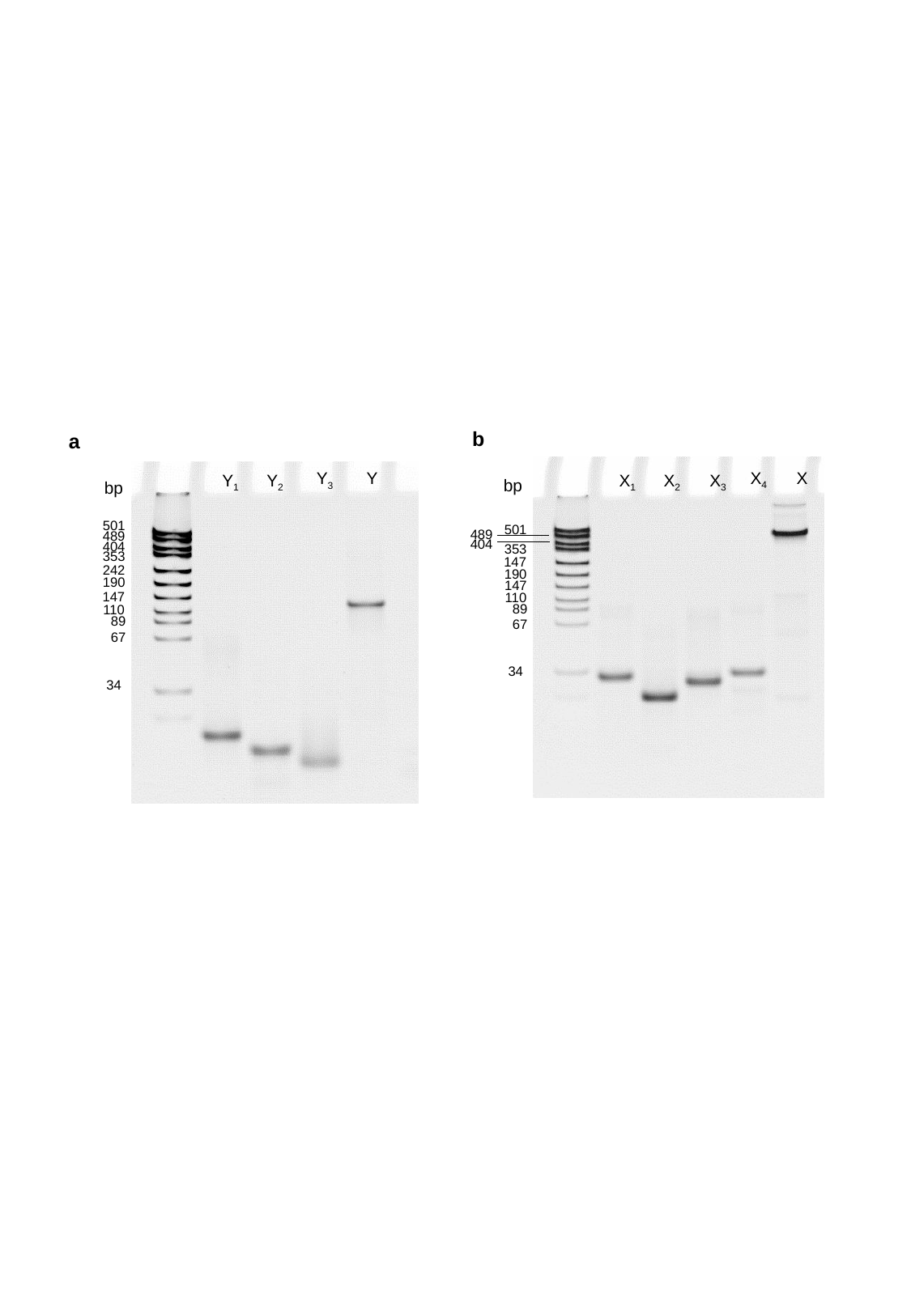

b
a
X4
X
X1
X2
X3
bp
501
489
404
353
147
190
147
110
89
67
34
Y
Y3
Y1
Y2
bp
501
489
404
353
242
190
147
110
89
67
34

## Slide 3
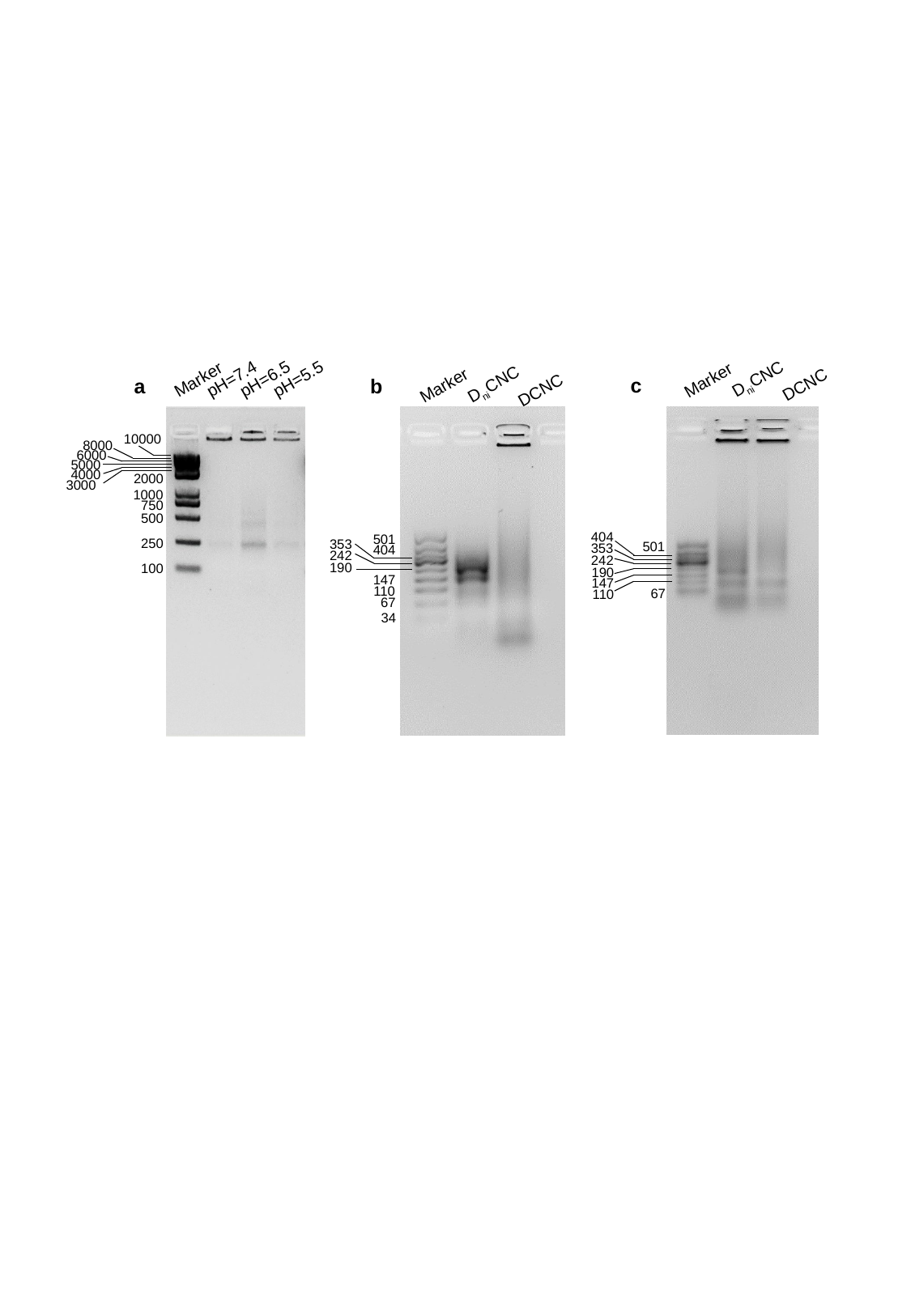

DniCNC
pH=7.4
pH=6.5
pH=5.5
Marker
Marker
c
DniCNC
Marker
DCNC
a
b
DCNC
10000
8000
6000
5000
4000
2000
3000
1000
750
500
404
501
250
353
501
353
404
242
242
190
100
190
147
147
110
67
110
67
34

## Slide 4
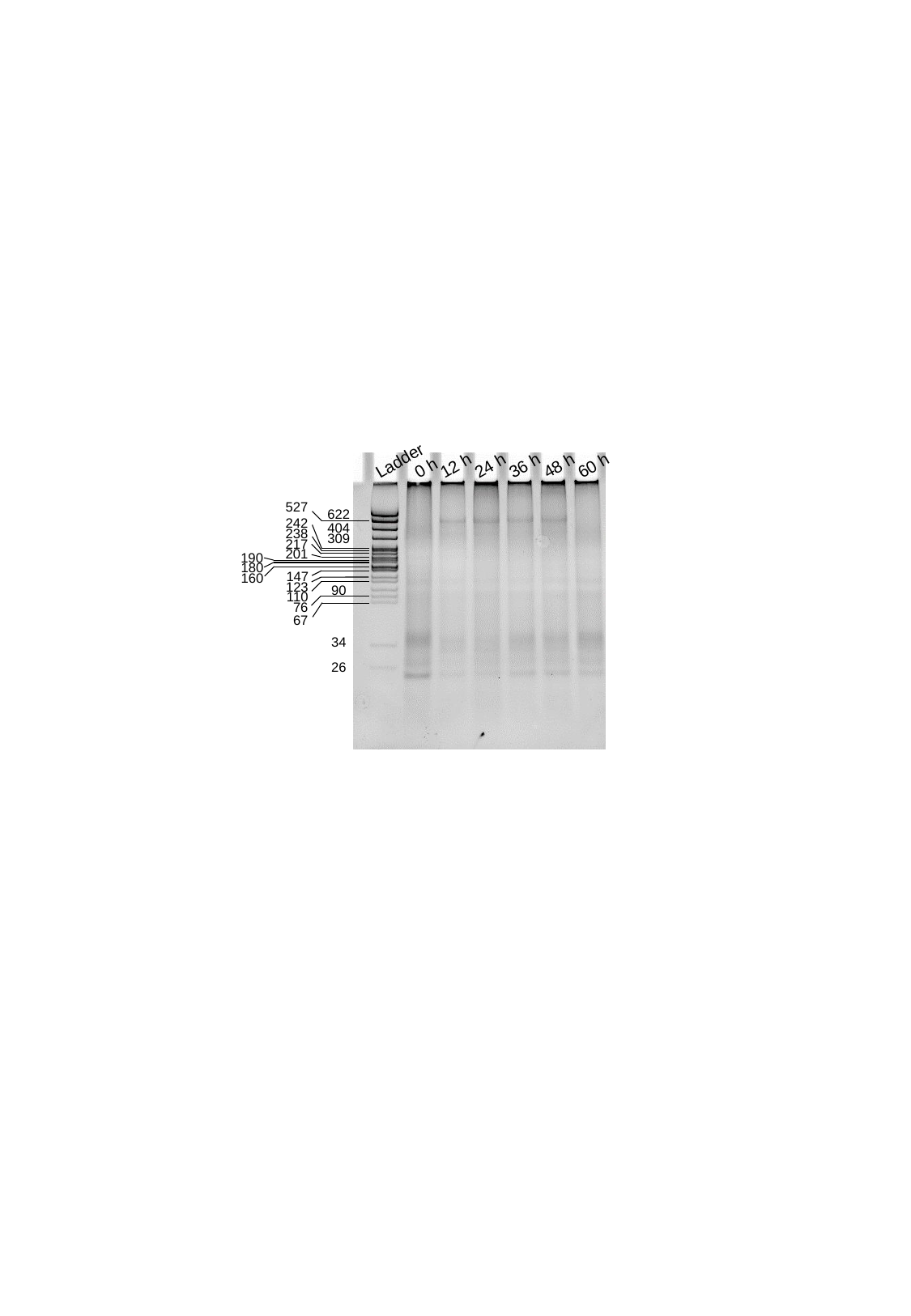

Ladder
12 h
24 h
36 h
48 h
60 h
0 h
527
622
242
404
238
309
217
201
190
180
147
160
123
90
110
76
67
34
26

## Slide 5
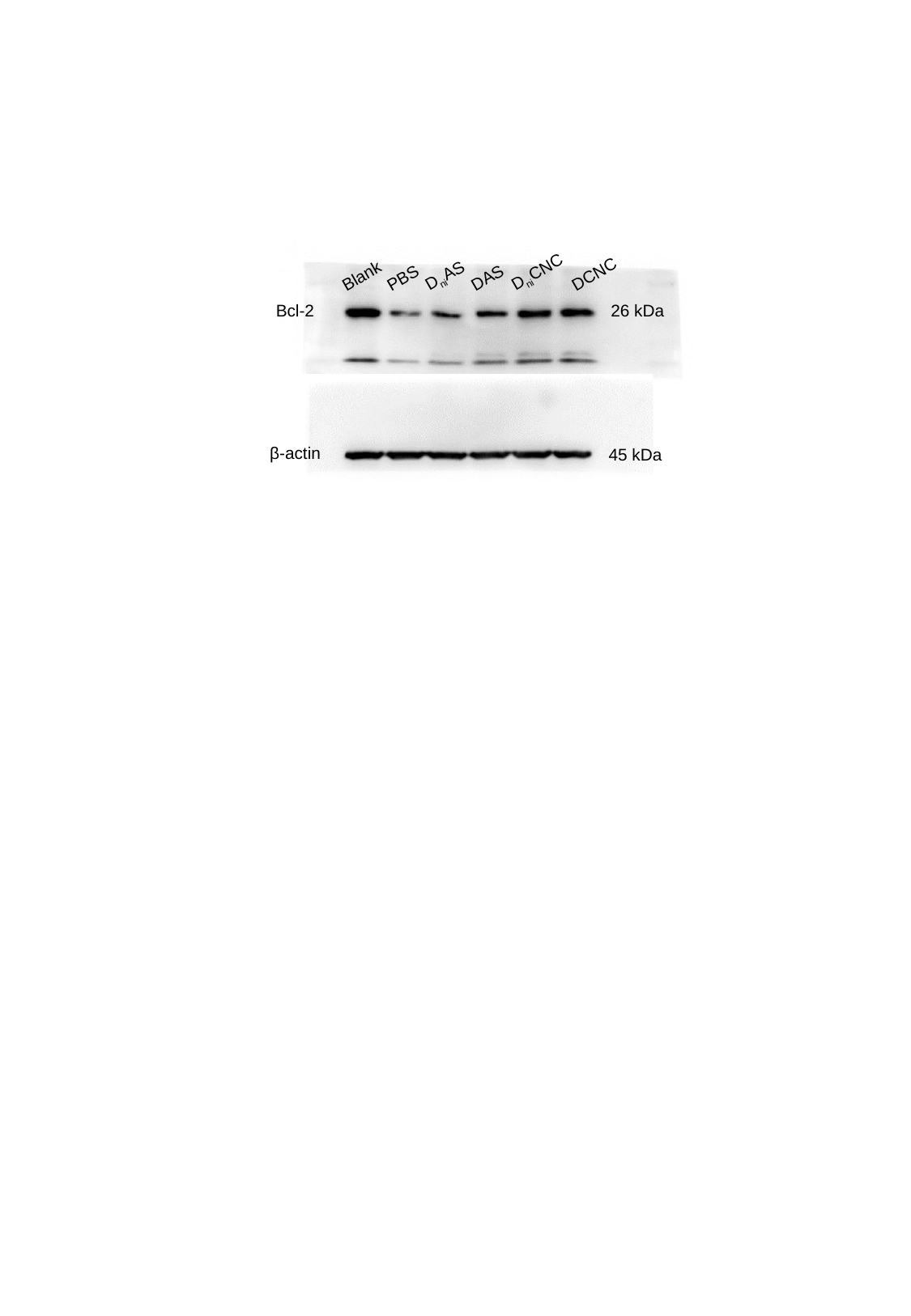

DniCNC
DCNC
DniAS
Blank
DAS
PBS
Bcl-2
26 kDa
β-actin
45 kDa
