# Supplementary figures and images for: Dynamic assembly of DNA-ceria nanocomplex in living cells generates artificial peroxisome (part 2 of 2)
Source: Nat Commun. 2022 Dec 14;13:7739. doi: 10.1038/s41467-022-35472-2 (PMC9751304; doi:10.1038/s41467-022-35472-2)

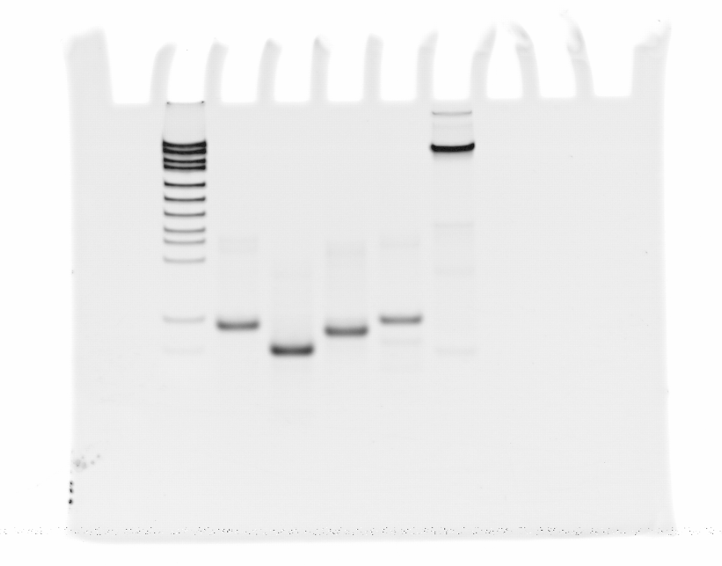

Supplement: Supplementary file 3 — Source data [file 41467_2022_35472_MOESM3_ESM.zip › Fig S1/Fig S1b.tif]

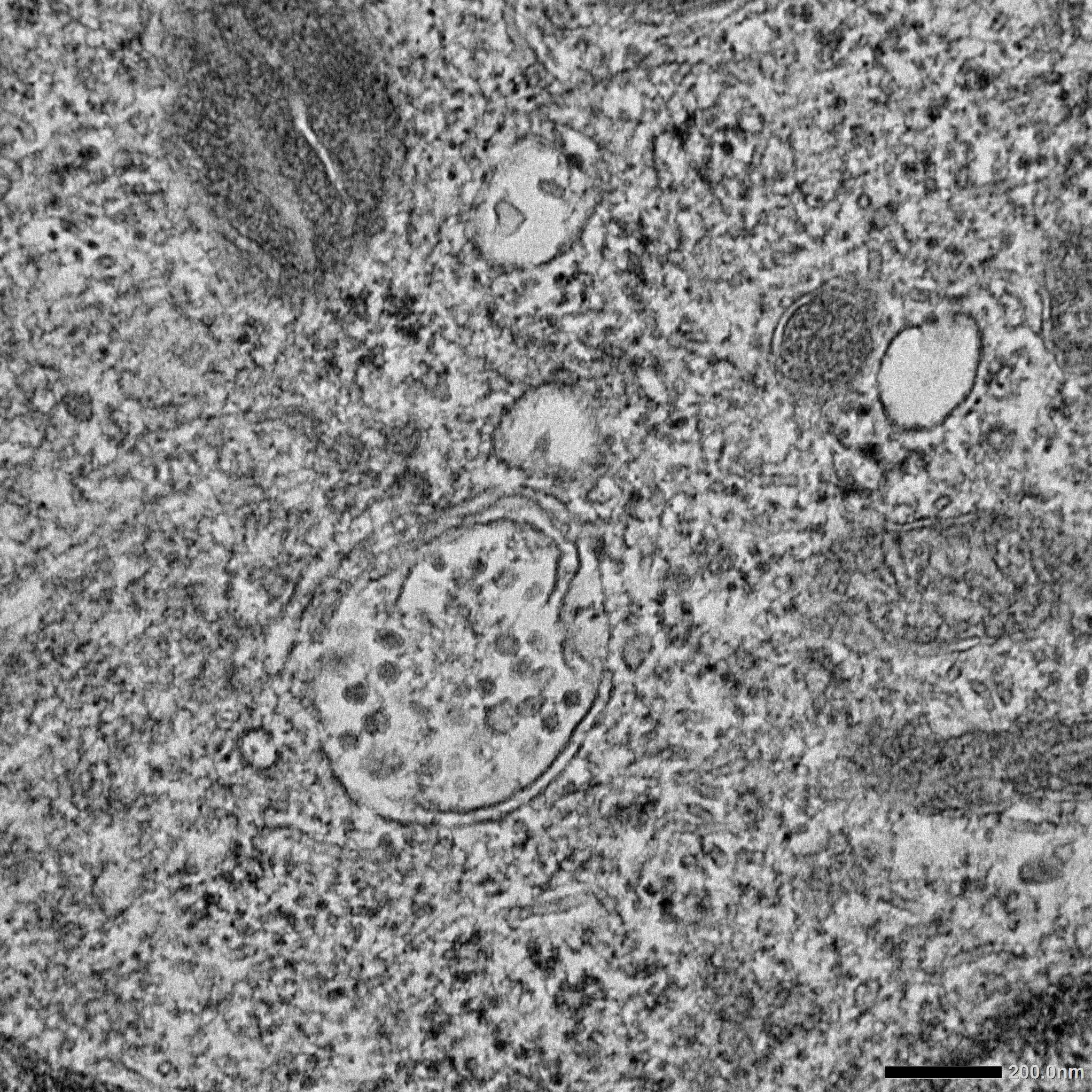

Supplement: Supplementary file 3 — Source data [file 41467_2022_35472_MOESM3_ESM.zip › Fig S20/JEM-1400 Flash_1_SA-MAG_X20k_014.jpg]

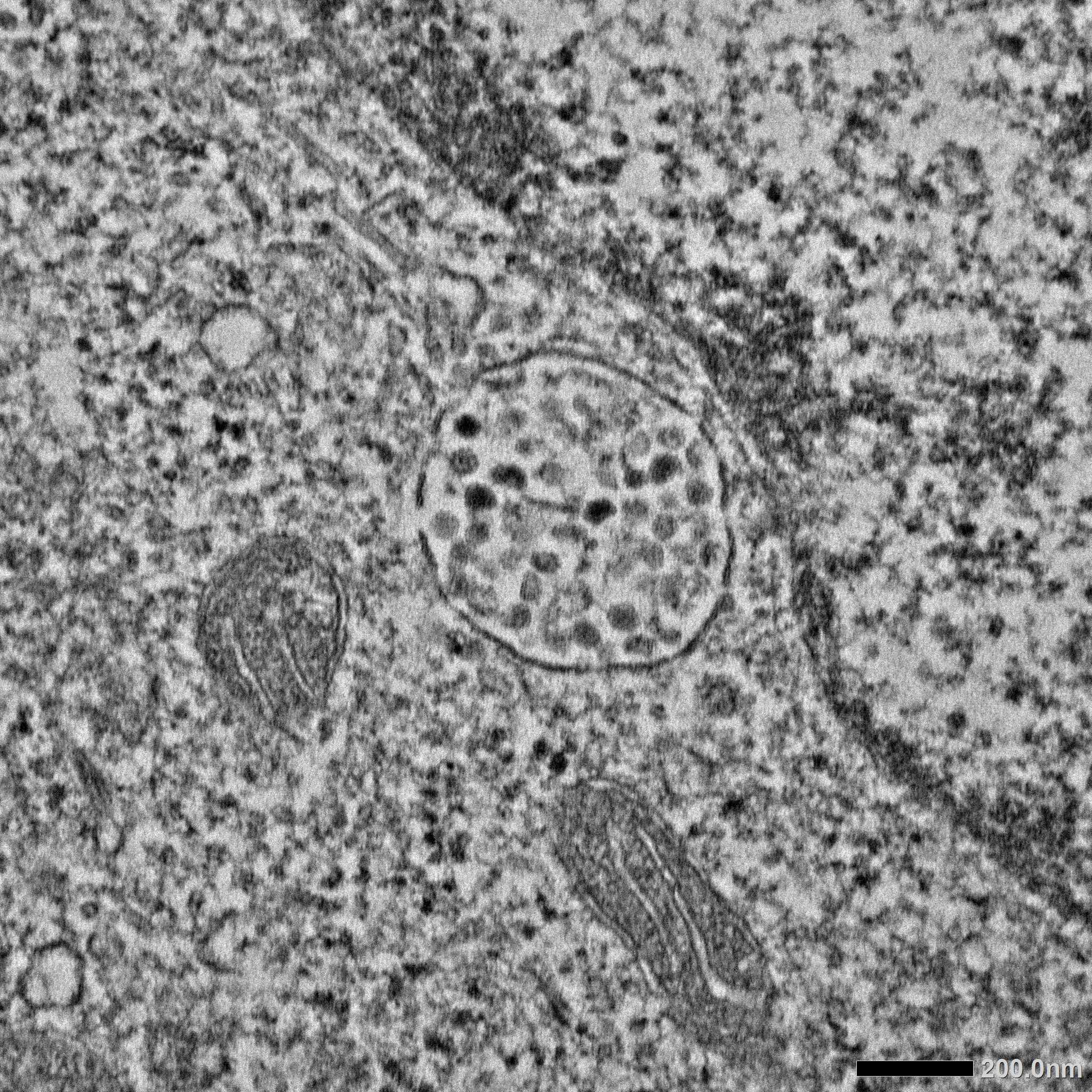

Supplement: Supplementary file 3 — Source data [file 41467_2022_35472_MOESM3_ESM.zip › Fig S20/JEM-1400 Flash_1_SA-MAG_X20k_058.jpg]

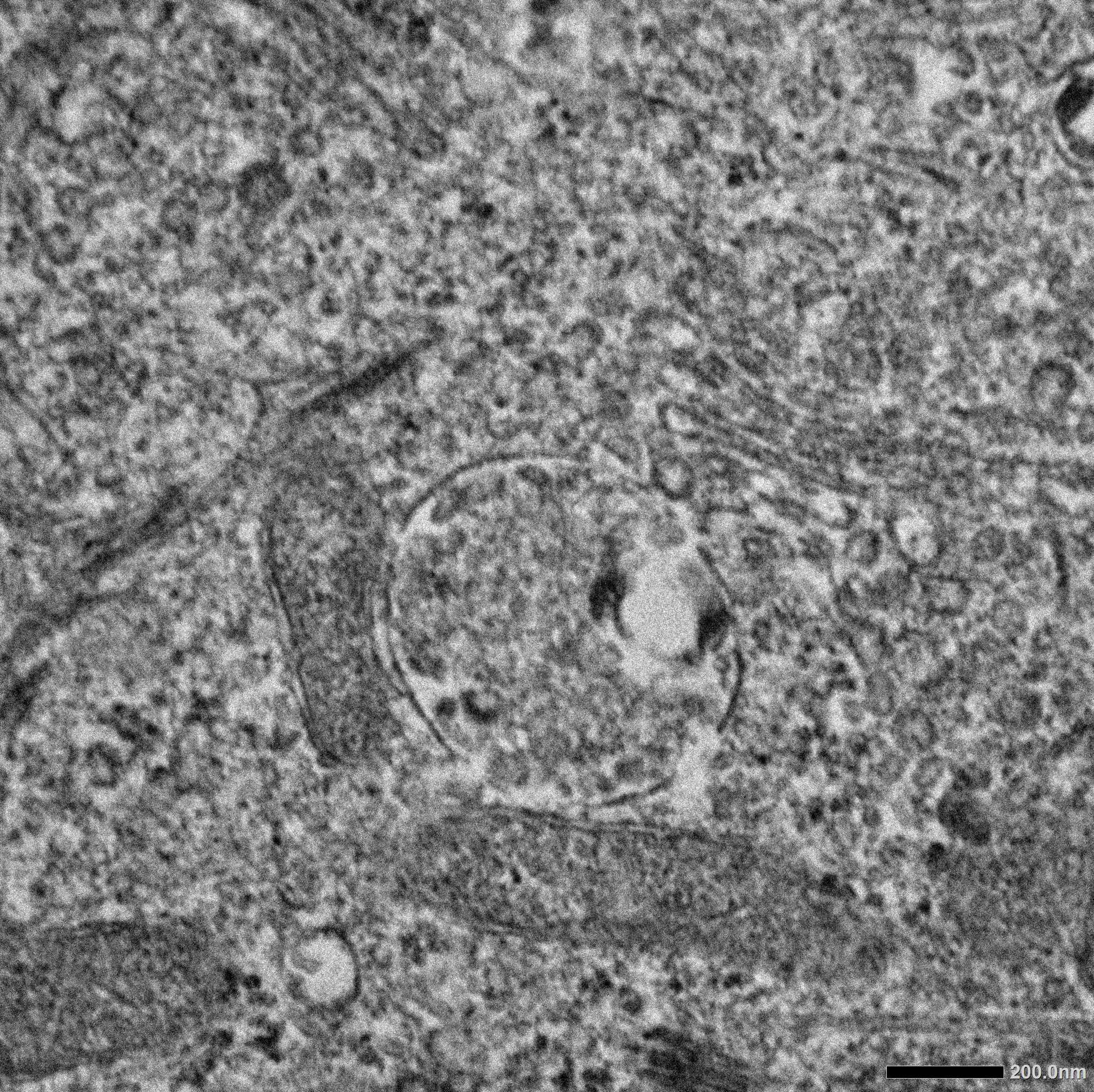

Supplement: Supplementary file 3 — Source data [file 41467_2022_35472_MOESM3_ESM.zip › Fig S20/JEM-1400 Flash_2_SA-MAG_X20k_066.jpg]

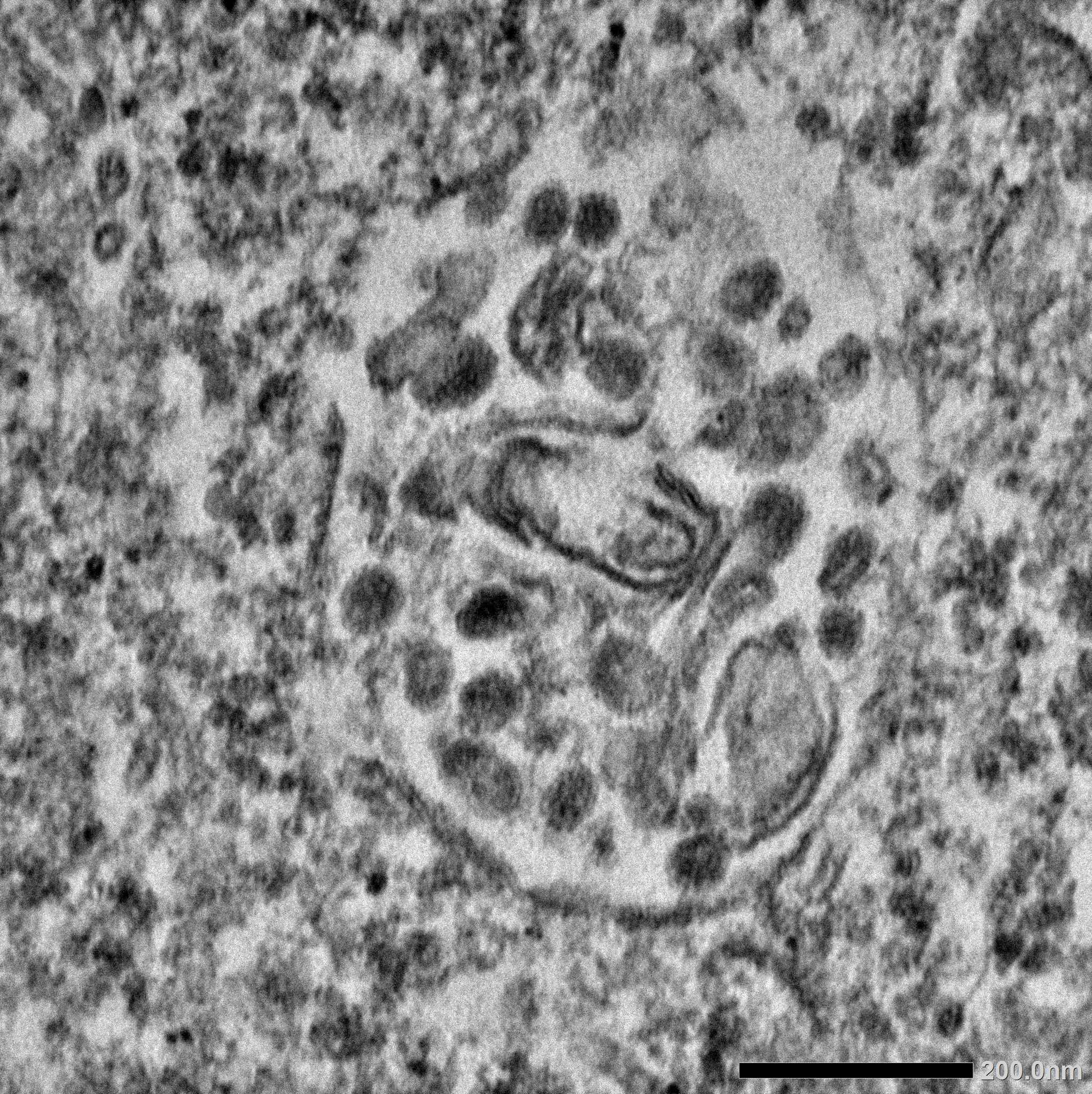

Supplement: Supplementary file 3 — Source data [file 41467_2022_35472_MOESM3_ESM.zip › Fig S20/JEM-1400 Flash_2_SA-MAG_X40k_082.jpg]

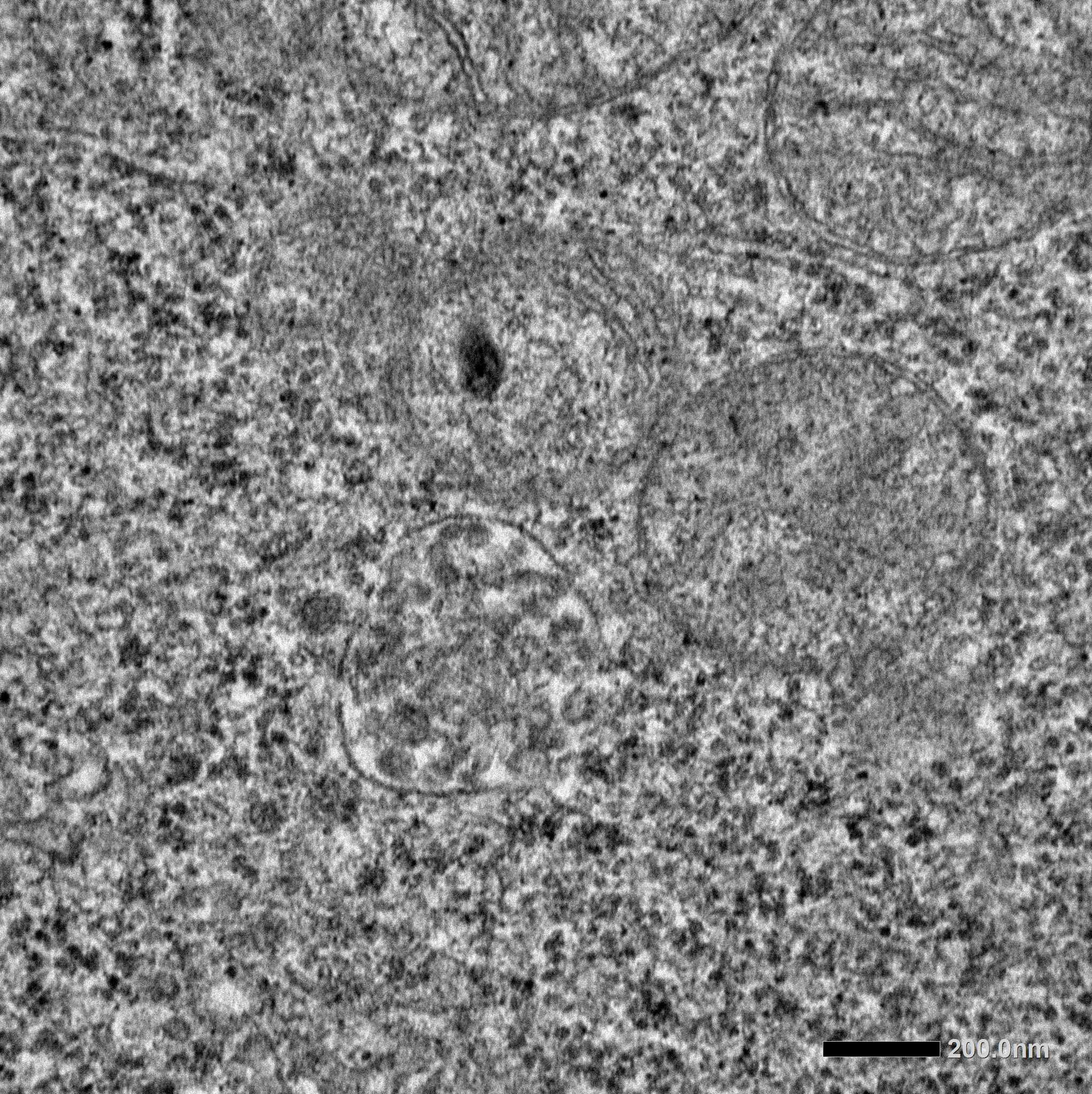

Supplement: Supplementary file 3 — Source data [file 41467_2022_35472_MOESM3_ESM.zip › Fig S20/JEM-1400 Flash_3_SA-MAG_X20k_008.jpg]

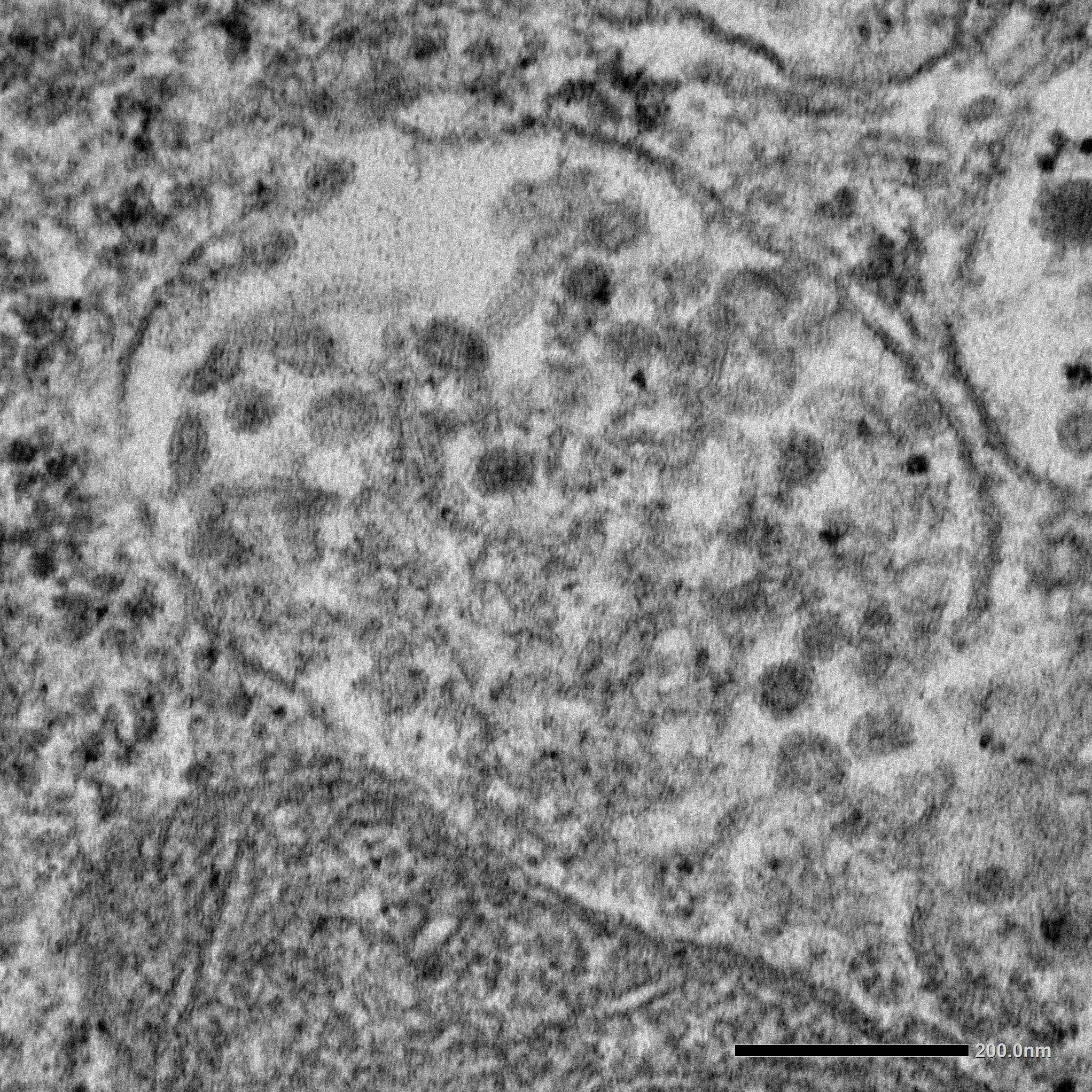

Supplement: Supplementary file 3 — Source data [file 41467_2022_35472_MOESM3_ESM.zip › Fig S20/JEM-1400 Flash_3_SA-MAG_X40k_031.jpg]

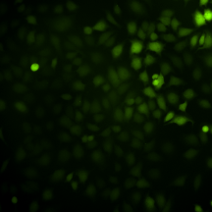

Supplement: Supplementary file 3 — Source data [file 41467_2022_35472_MOESM3_ESM.zip › Fig S22/a 0.5h.png]

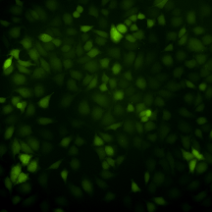

Supplement: Supplementary file 3 — Source data [file 41467_2022_35472_MOESM3_ESM.zip › Fig S22/a 0h.png]

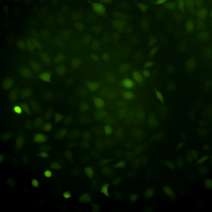

Supplement: Supplementary file 3 — Source data [file 41467_2022_35472_MOESM3_ESM.zip › Fig S22/a 1h.png]

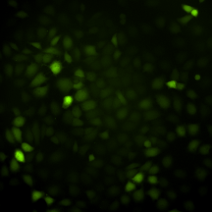

Supplement: Supplementary file 3 — Source data [file 41467_2022_35472_MOESM3_ESM.zip › Fig S22/a 2h.png]

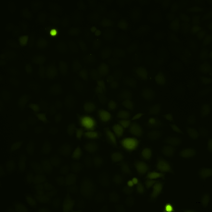

Supplement: Supplementary file 3 — Source data [file 41467_2022_35472_MOESM3_ESM.zip › Fig S22/a 4h.png]

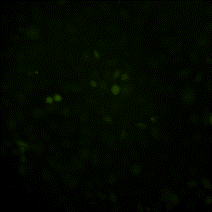

Supplement: Supplementary file 3 — Source data [file 41467_2022_35472_MOESM3_ESM.zip › Fig S22/a 6h.png]

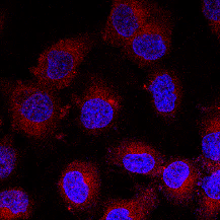

Supplement: Supplementary file 3 — Source data [file 41467_2022_35472_MOESM3_ESM.zip › Fig S22/b 0.5h.png]

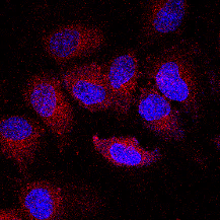

Supplement: Supplementary file 3 — Source data [file 41467_2022_35472_MOESM3_ESM.zip › Fig S22/b 0h.png]

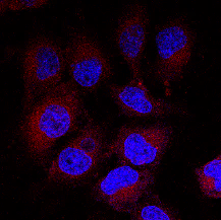

Supplement: Supplementary file 3 — Source data [file 41467_2022_35472_MOESM3_ESM.zip › Fig S22/b 1h.png]

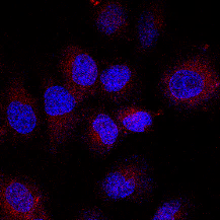

Supplement: Supplementary file 3 — Source data [file 41467_2022_35472_MOESM3_ESM.zip › Fig S22/b 2h.png]

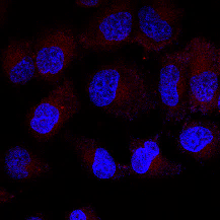

Supplement: Supplementary file 3 — Source data [file 41467_2022_35472_MOESM3_ESM.zip › Fig S22/b 4h.png]

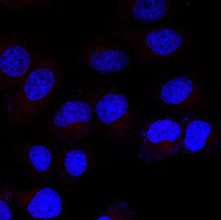

Supplement: Supplementary file 3 — Source data [file 41467_2022_35472_MOESM3_ESM.zip › Fig S22/b 6h.png]

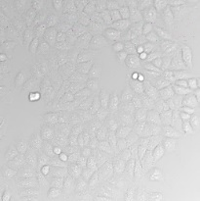

Supplement: Supplementary file 3 — Source data [file 41467_2022_35472_MOESM3_ESM.zip › Fig S23/BF 0h.png]

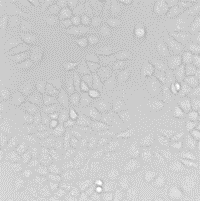

Supplement: Supplementary file 3 — Source data [file 41467_2022_35472_MOESM3_ESM.zip › Fig S23/BF 12h.png]

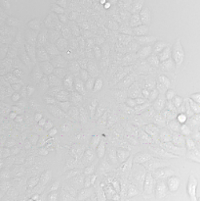

Supplement: Supplementary file 3 — Source data [file 41467_2022_35472_MOESM3_ESM.zip › Fig S23/BF 24h.png]

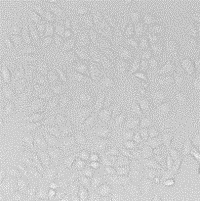

Supplement: Supplementary file 3 — Source data [file 41467_2022_35472_MOESM3_ESM.zip › Fig S23/BF 48h.png]

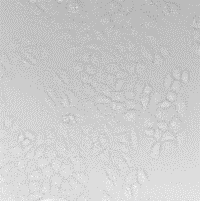

Supplement: Supplementary file 3 — Source data [file 41467_2022_35472_MOESM3_ESM.zip › Fig S23/BF 6h.png]

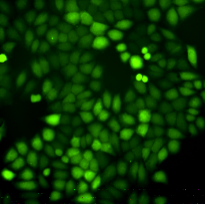

Supplement: Supplementary file 3 — Source data [file 41467_2022_35472_MOESM3_ESM.zip › Fig S23/DCF 0h.png]

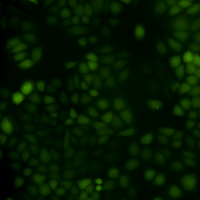

Supplement: Supplementary file 3 — Source data [file 41467_2022_35472_MOESM3_ESM.zip › Fig S23/DCF 12h.png]

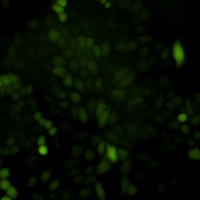

Supplement: Supplementary file 3 — Source data [file 41467_2022_35472_MOESM3_ESM.zip › Fig S23/DCF 24h.png]

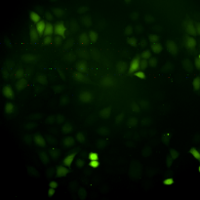

Supplement: Supplementary file 3 — Source data [file 41467_2022_35472_MOESM3_ESM.zip › Fig S23/DCF 48h.png]

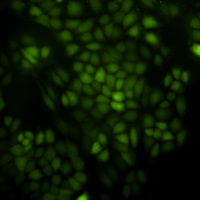

Supplement: Supplementary file 3 — Source data [file 41467_2022_35472_MOESM3_ESM.zip › Fig S23/DCF 6h.png]

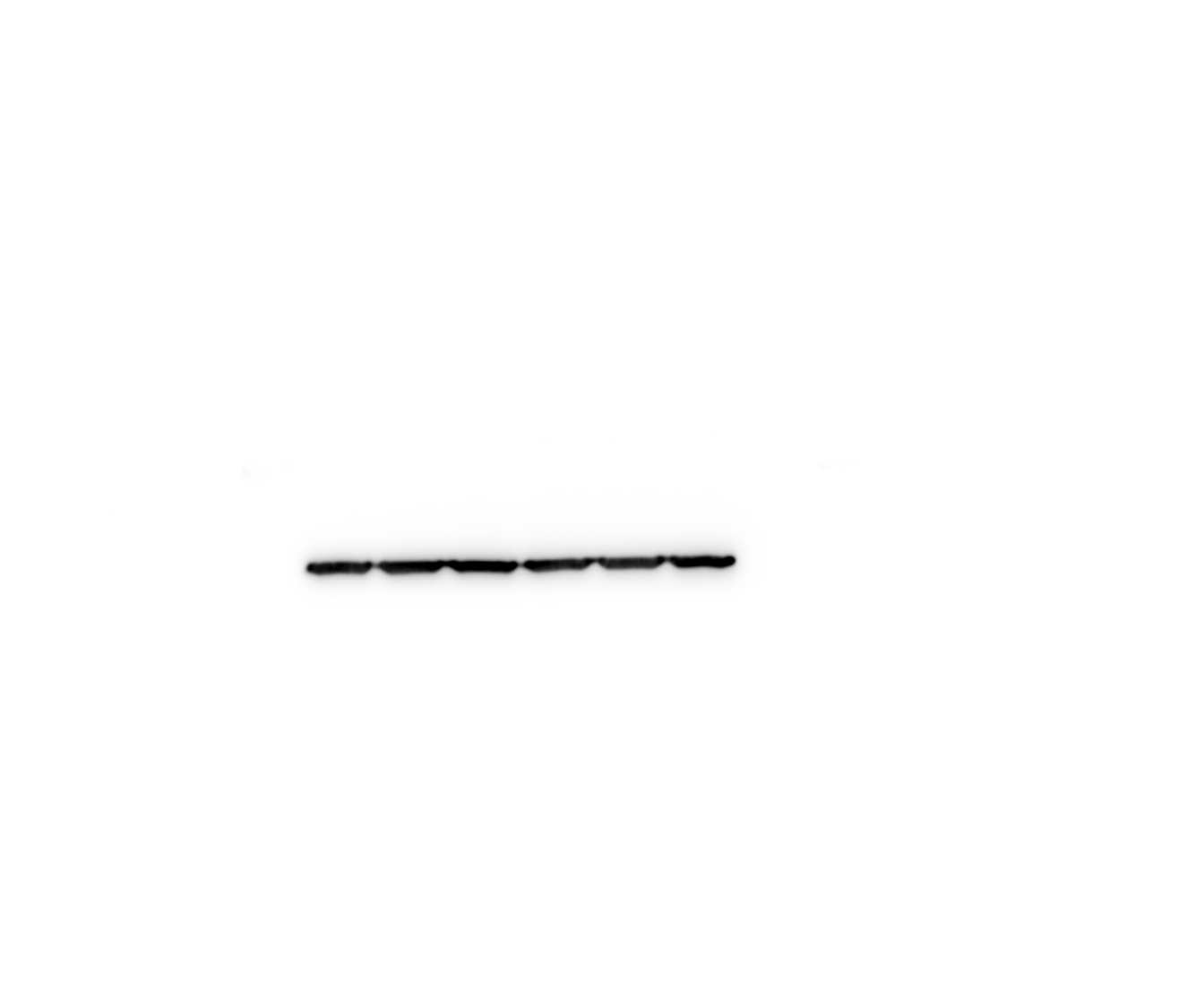

Supplement: Supplementary file 3 — Source data [file 41467_2022_35472_MOESM3_ESM.zip › Fig S24/actin.Tif]

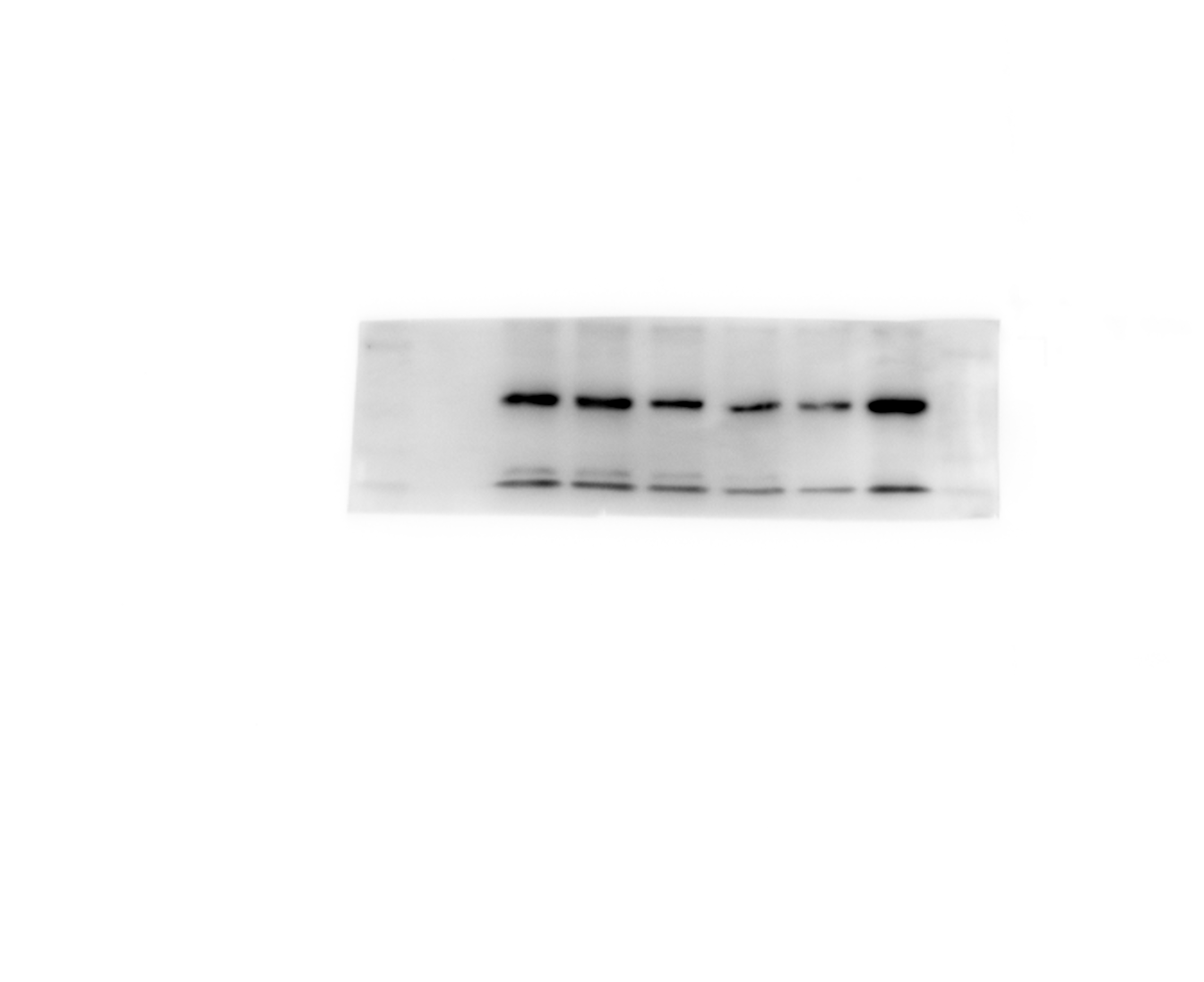

Supplement: Supplementary file 3 — Source data [file 41467_2022_35472_MOESM3_ESM.zip › Fig S24/bcl.Tif]

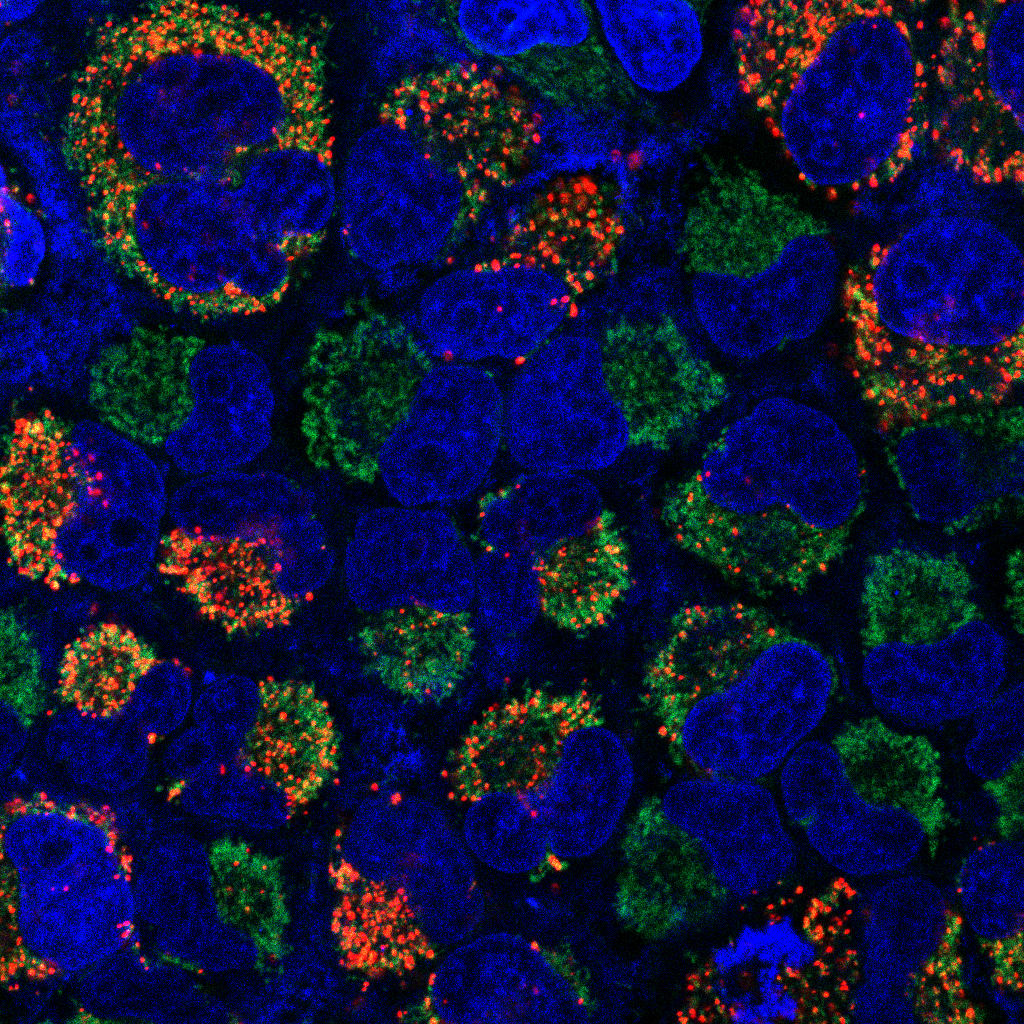

Supplement: Supplementary file 3 — Source data [file 41467_2022_35472_MOESM3_ESM.zip › Fig S25/0h/0h_c1-3.tif]

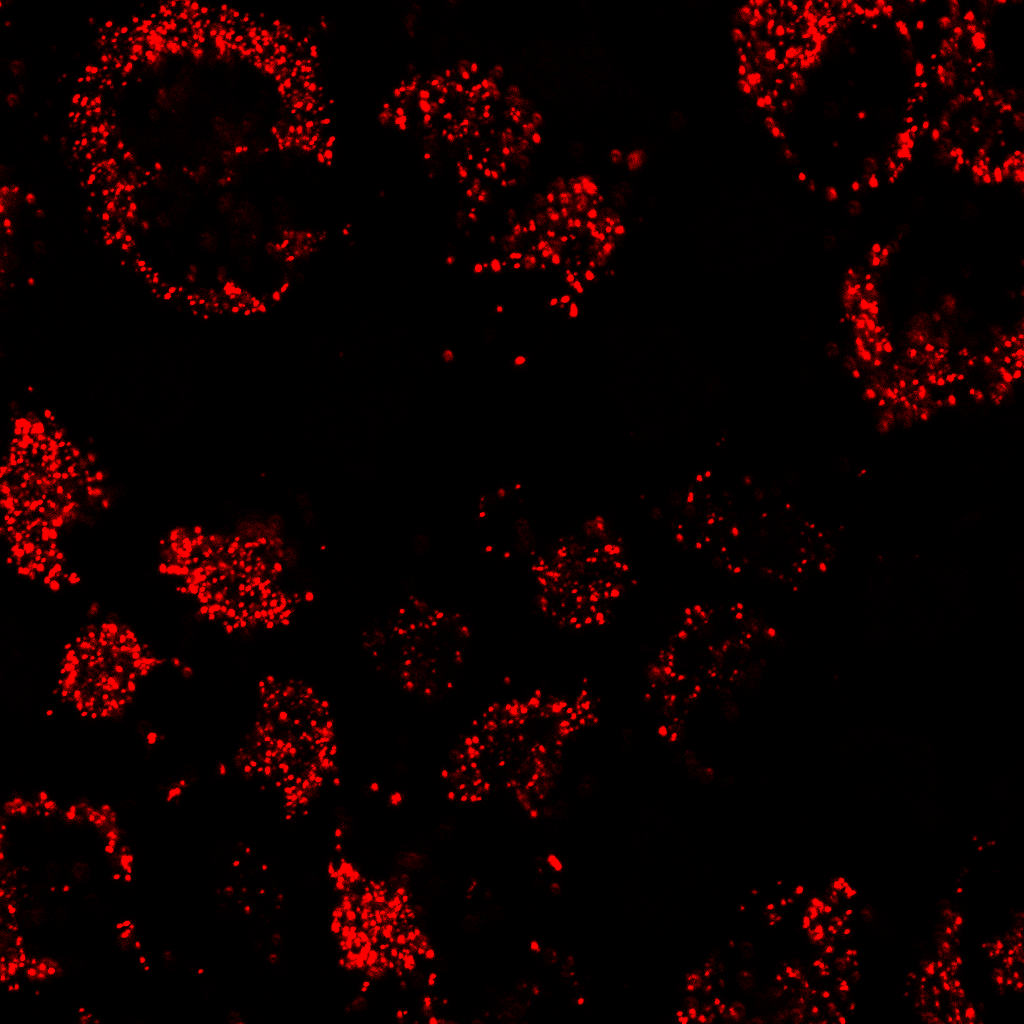

Supplement: Supplementary file 3 — Source data [file 41467_2022_35472_MOESM3_ESM.zip › Fig S25/0h/0h_c1.tif]

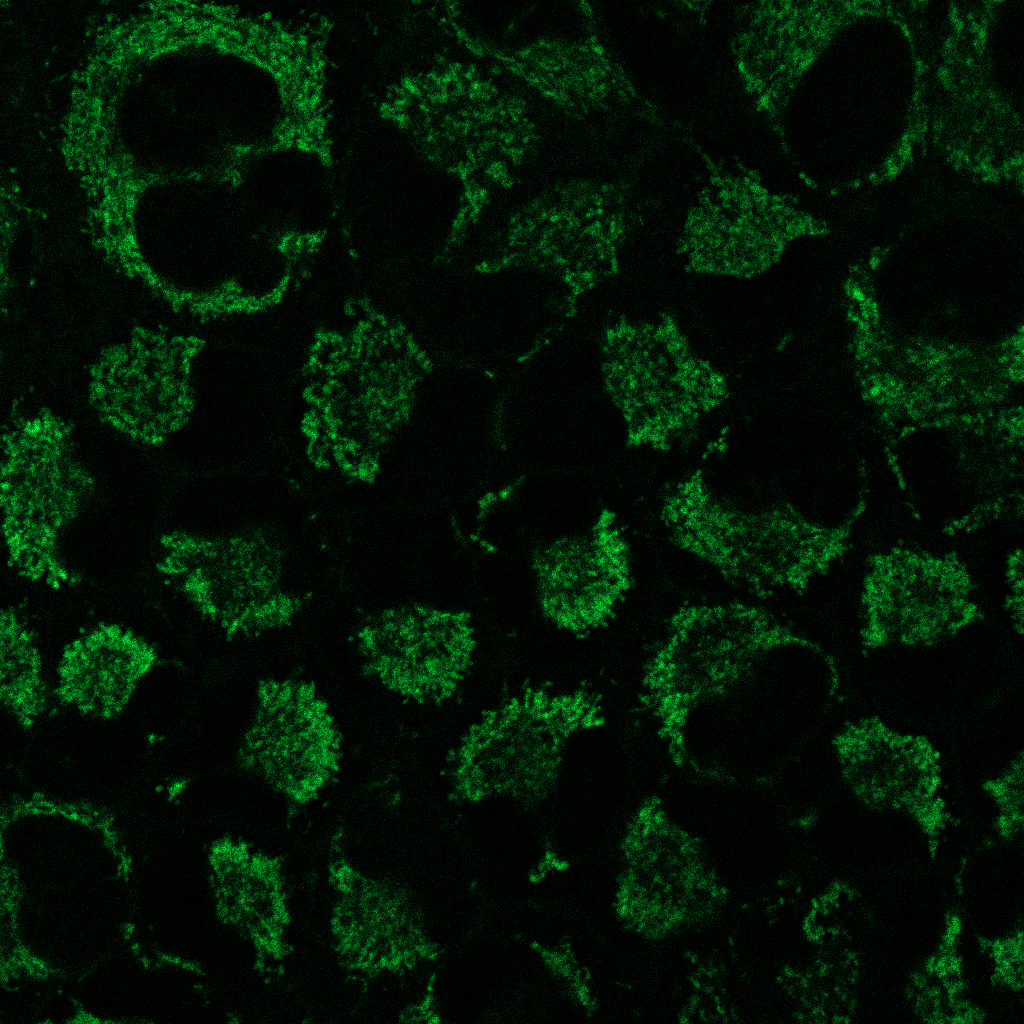

Supplement: Supplementary file 3 — Source data [file 41467_2022_35472_MOESM3_ESM.zip › Fig S25/0h/0h_c2.tif]

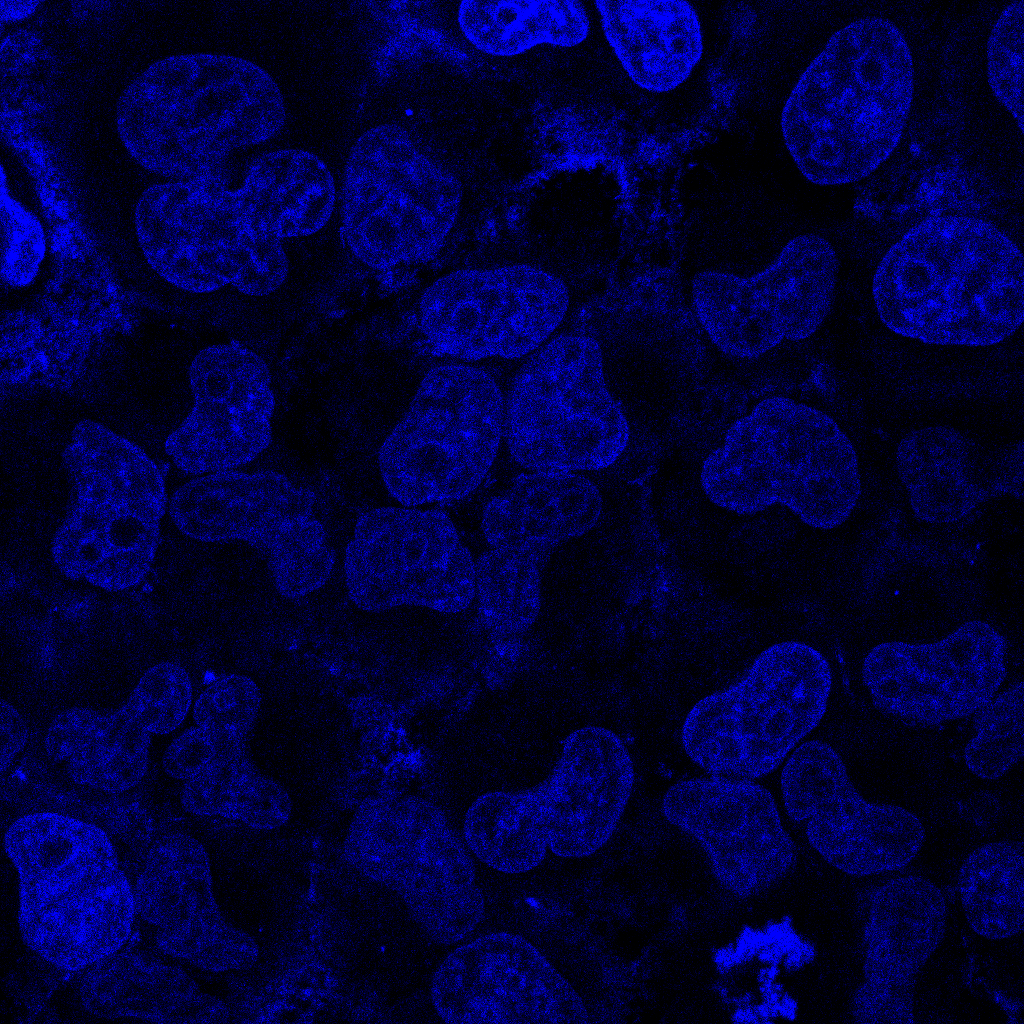

Supplement: Supplementary file 3 — Source data [file 41467_2022_35472_MOESM3_ESM.zip › Fig S25/0h/0h_c3.tif]

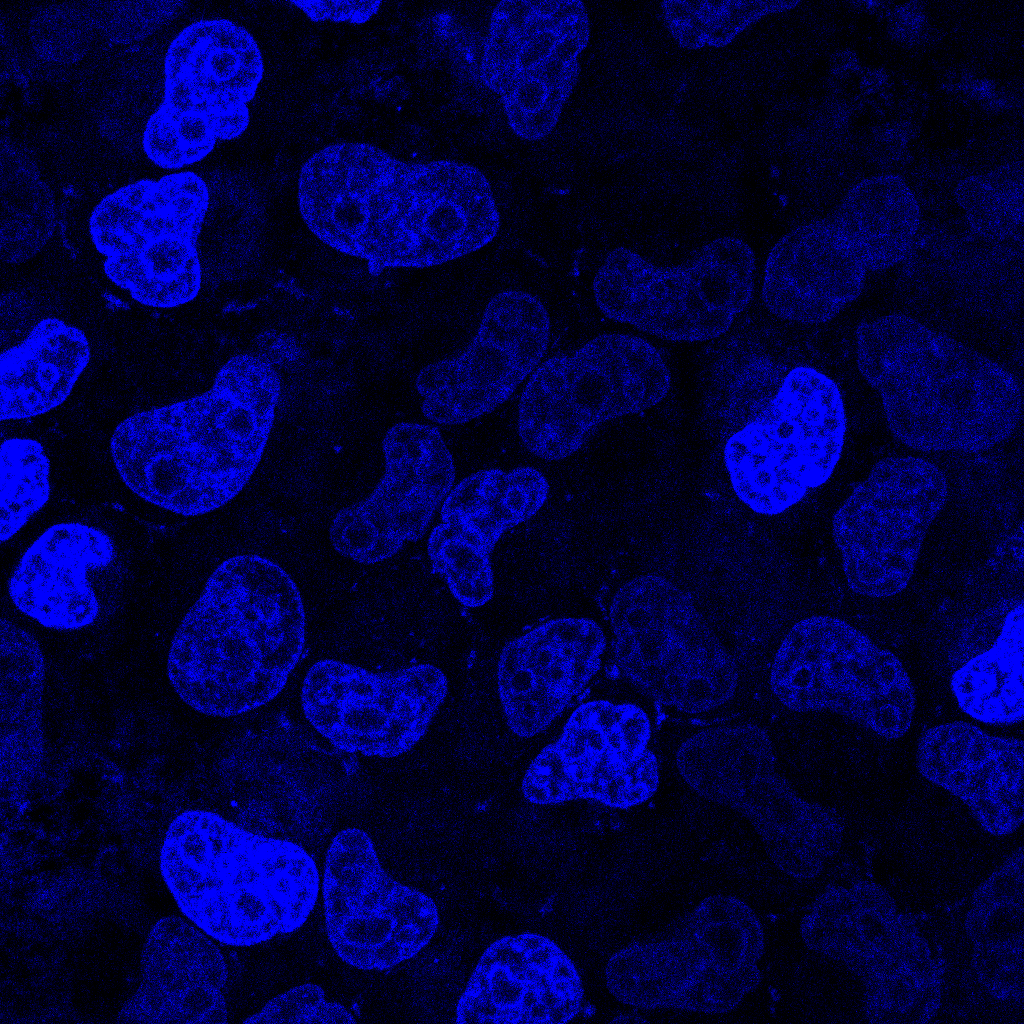

Supplement: Supplementary file 3 — Source data [file 41467_2022_35472_MOESM3_ESM.zip › Fig S25/12h/12h_c3.tif]

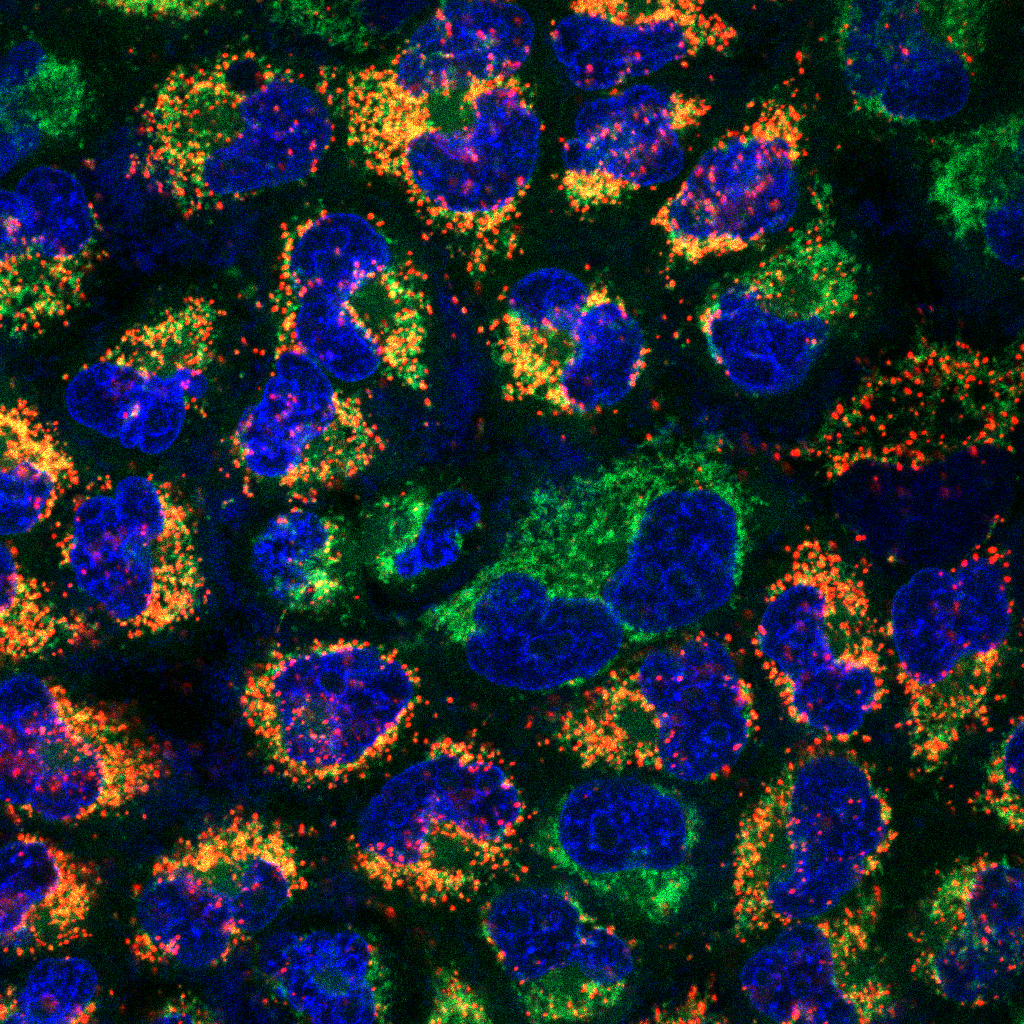

Supplement: Supplementary file 3 — Source data [file 41467_2022_35472_MOESM3_ESM.zip › Fig S25/24h/24h_c1-3.tif]

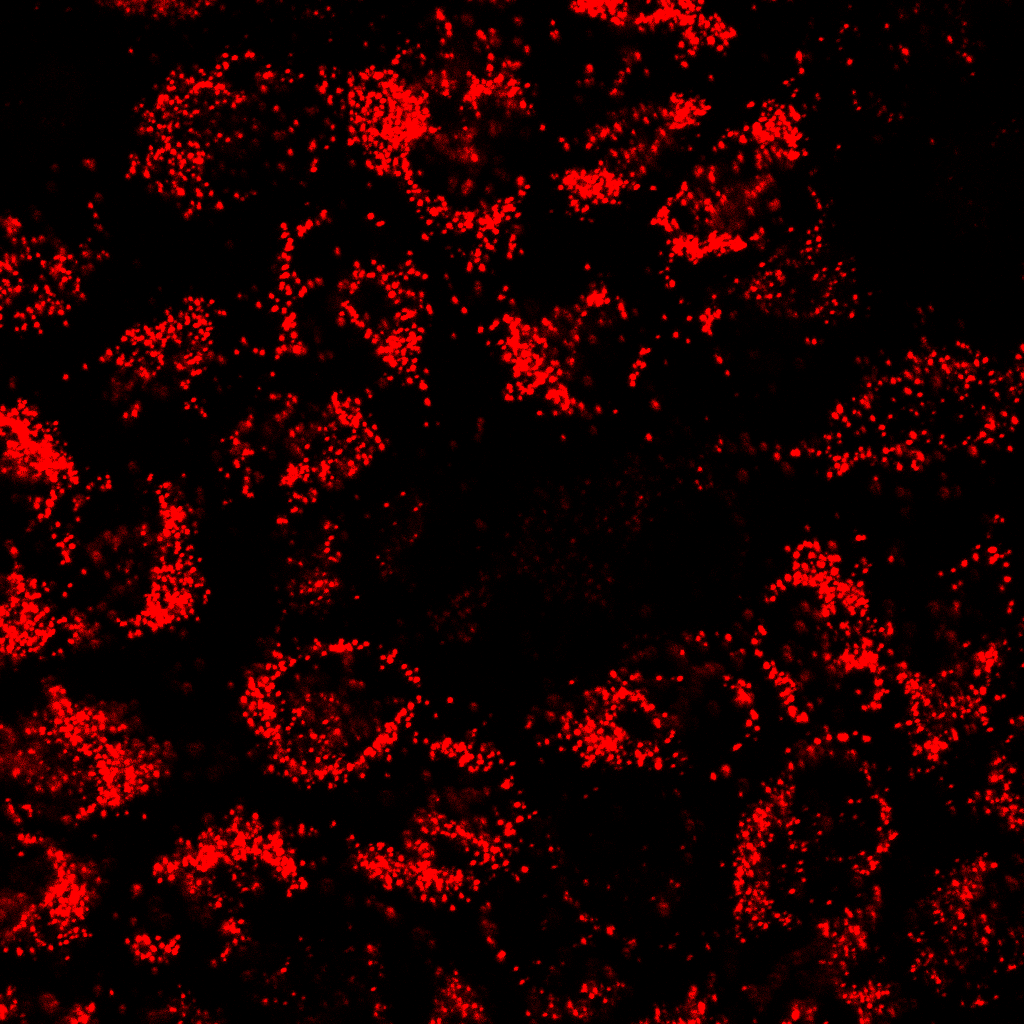

Supplement: Supplementary file 3 — Source data [file 41467_2022_35472_MOESM3_ESM.zip › Fig S25/24h/24h_c1.tif]

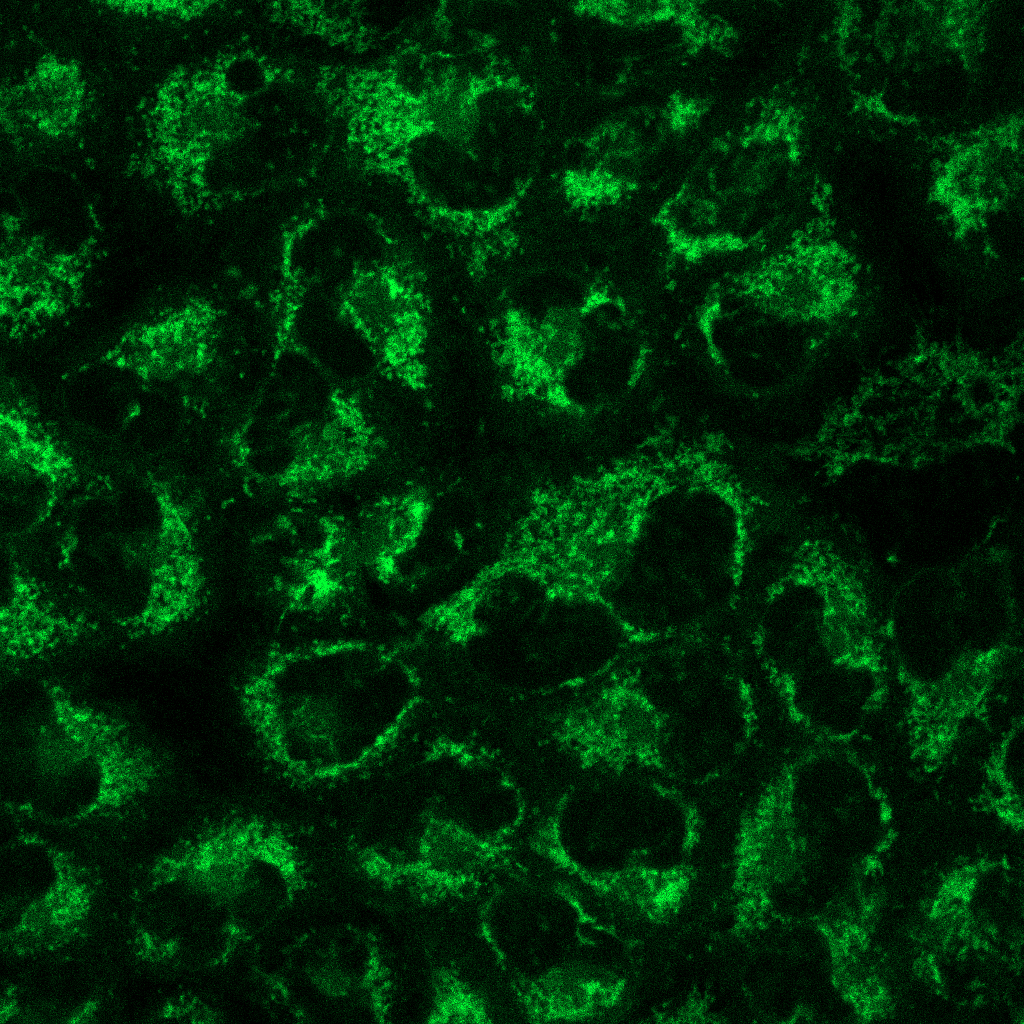

Supplement: Supplementary file 3 — Source data [file 41467_2022_35472_MOESM3_ESM.zip › Fig S25/24h/24h_c2.tif]

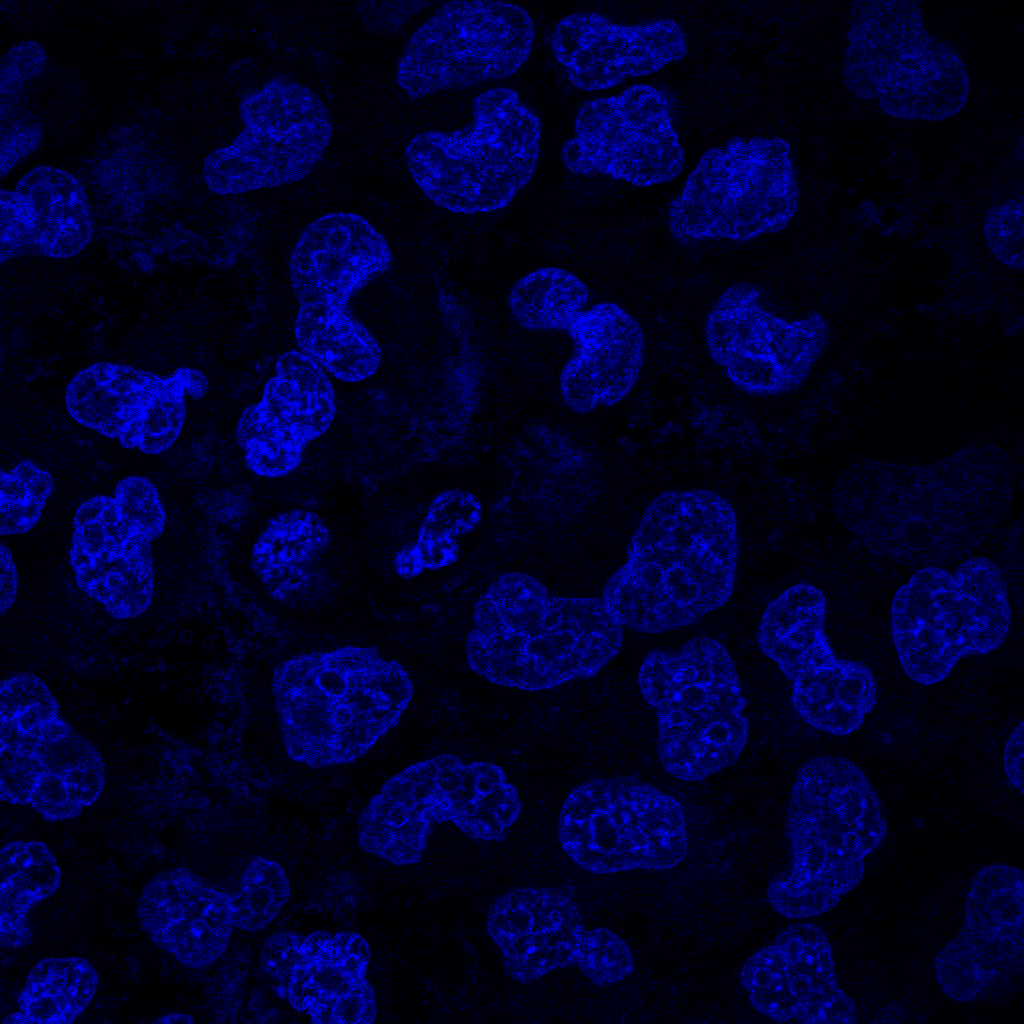

Supplement: Supplementary file 3 — Source data [file 41467_2022_35472_MOESM3_ESM.zip › Fig S25/24h/24h_c3.tif]

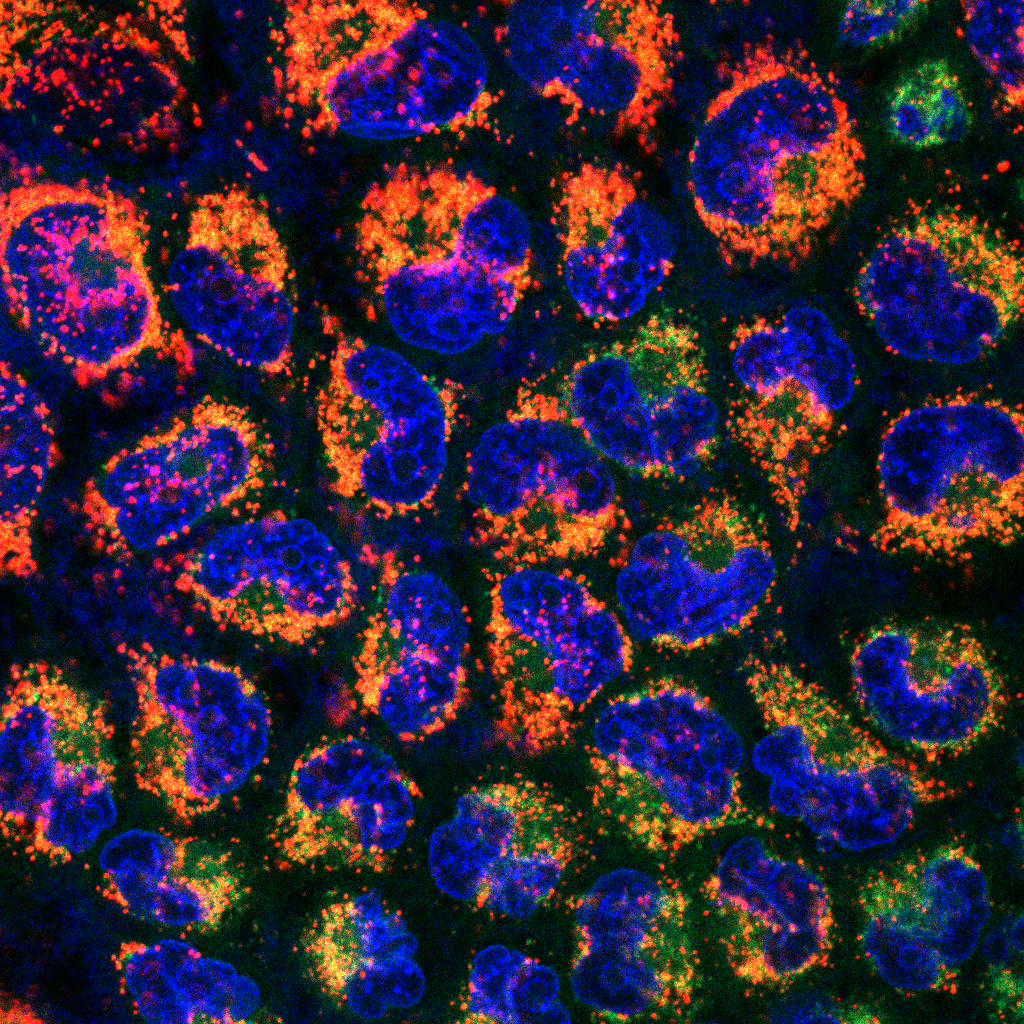

Supplement: Supplementary file 3 — Source data [file 41467_2022_35472_MOESM3_ESM.zip › Fig S25/48h/48h_c1-3.tif]

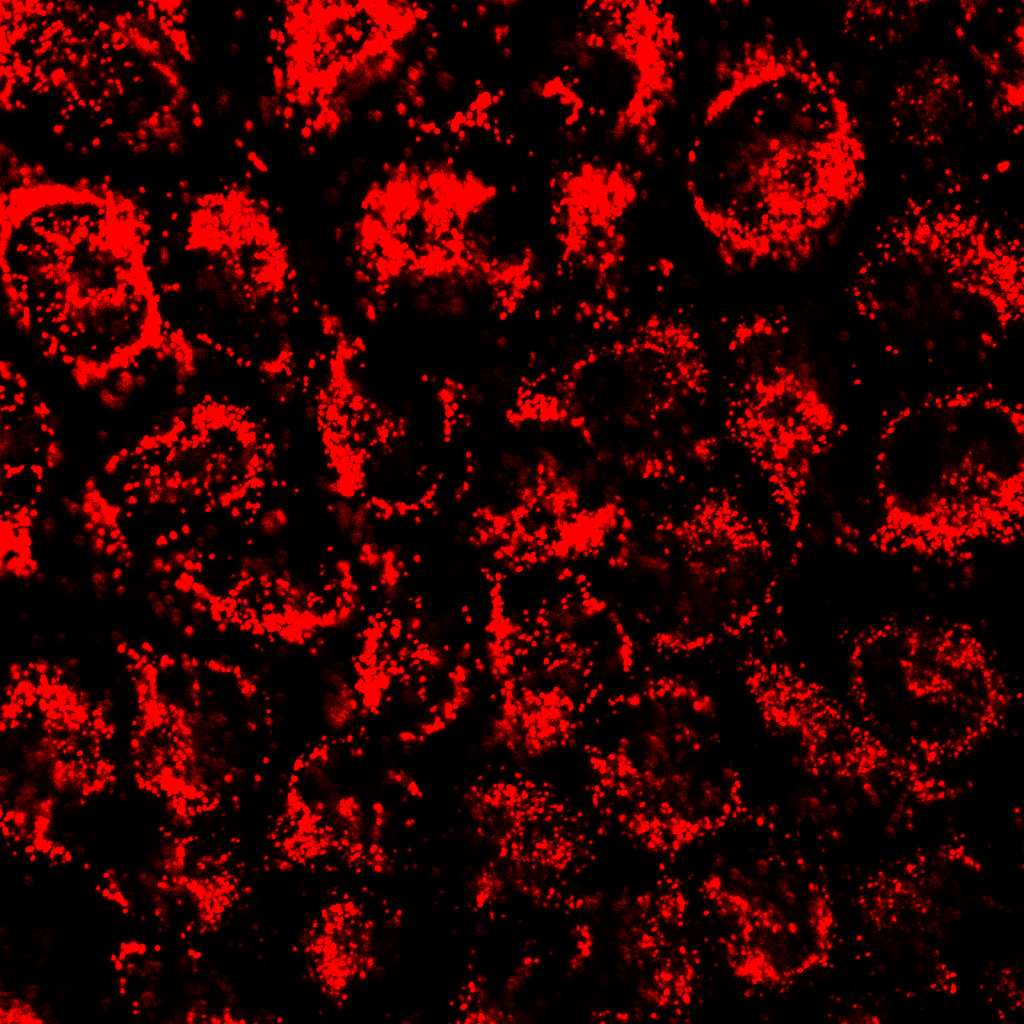

Supplement: Supplementary file 3 — Source data [file 41467_2022_35472_MOESM3_ESM.zip › Fig S25/48h/48h_c1.tif]

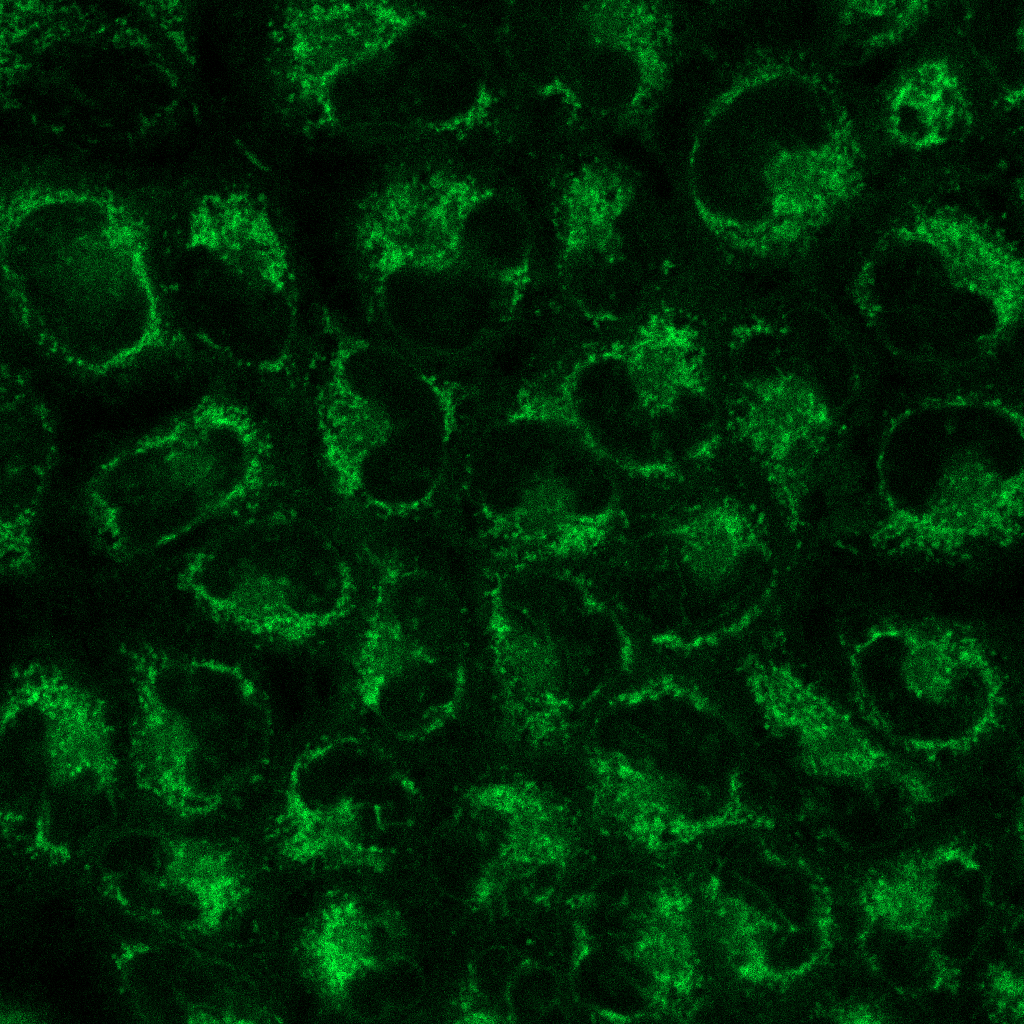

Supplement: Supplementary file 3 — Source data [file 41467_2022_35472_MOESM3_ESM.zip › Fig S25/48h/48h_c2.tif]

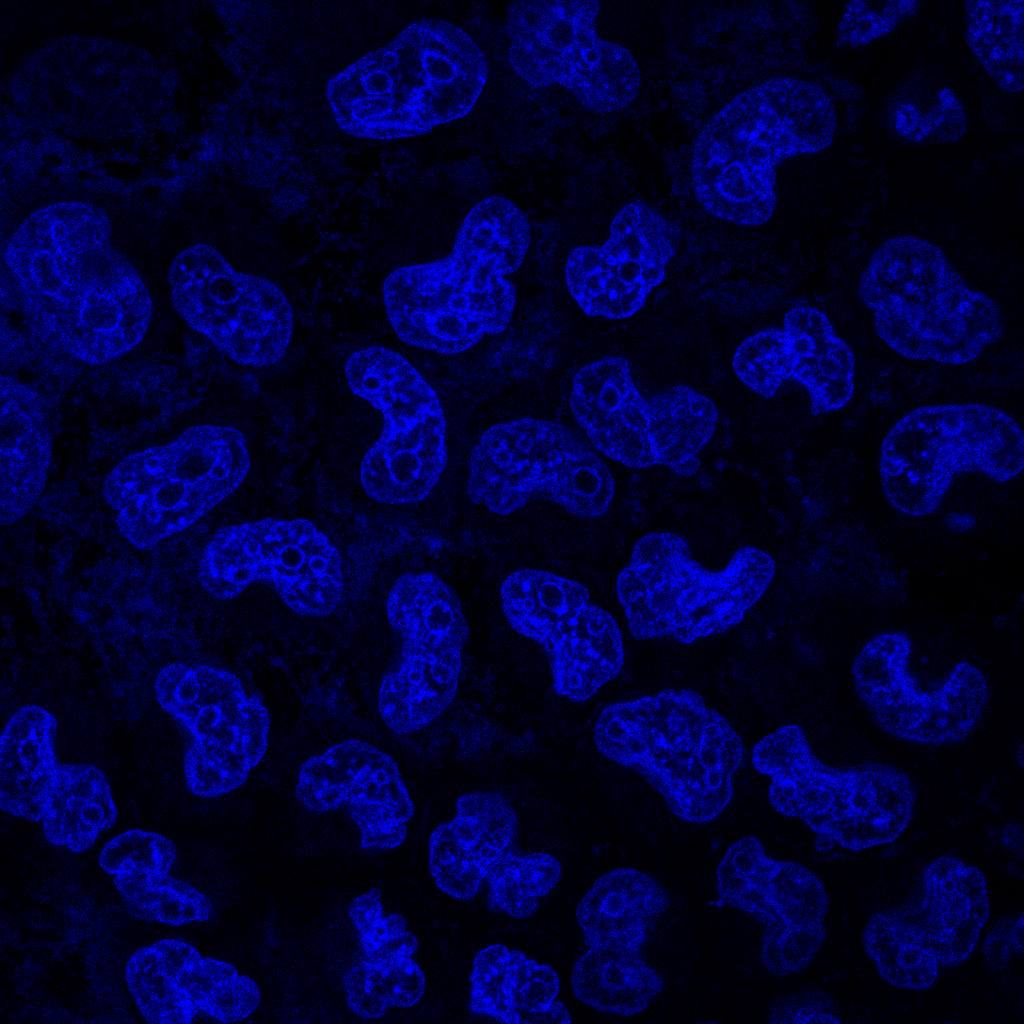

Supplement: Supplementary file 3 — Source data [file 41467_2022_35472_MOESM3_ESM.zip › Fig S25/48h/48h_c3.tif]

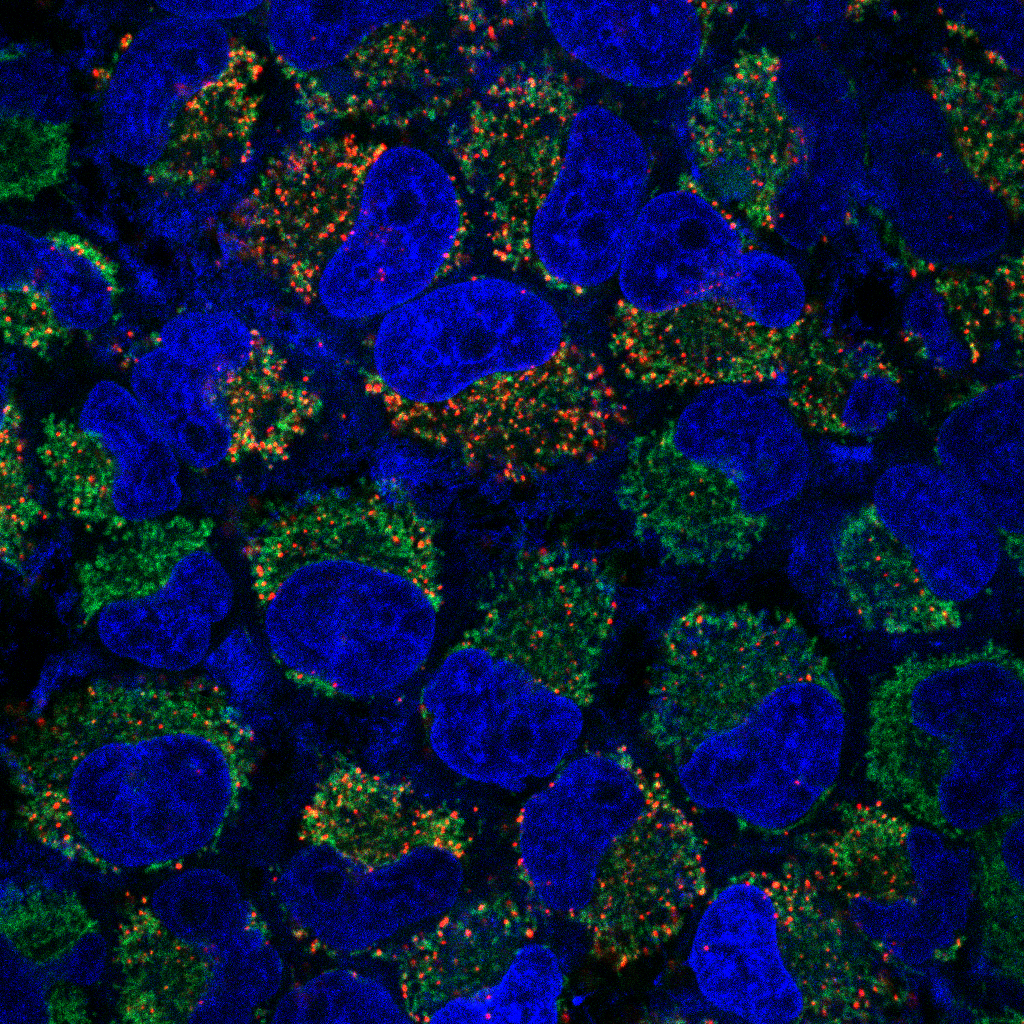

Supplement: Supplementary file 3 — Source data [file 41467_2022_35472_MOESM3_ESM.zip › Fig S25/4h/4h_c1-3.tif]

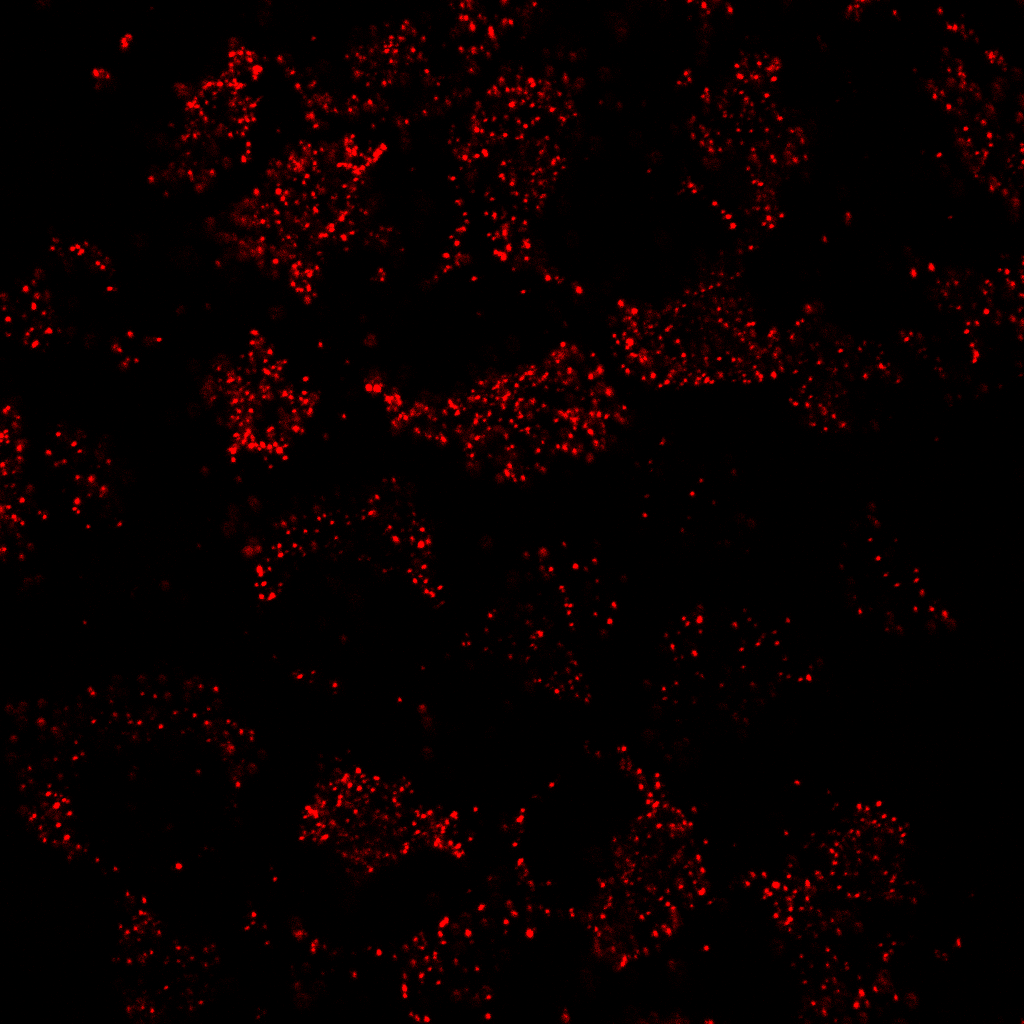

Supplement: Supplementary file 3 — Source data [file 41467_2022_35472_MOESM3_ESM.zip › Fig S25/4h/4h_c1.tif]

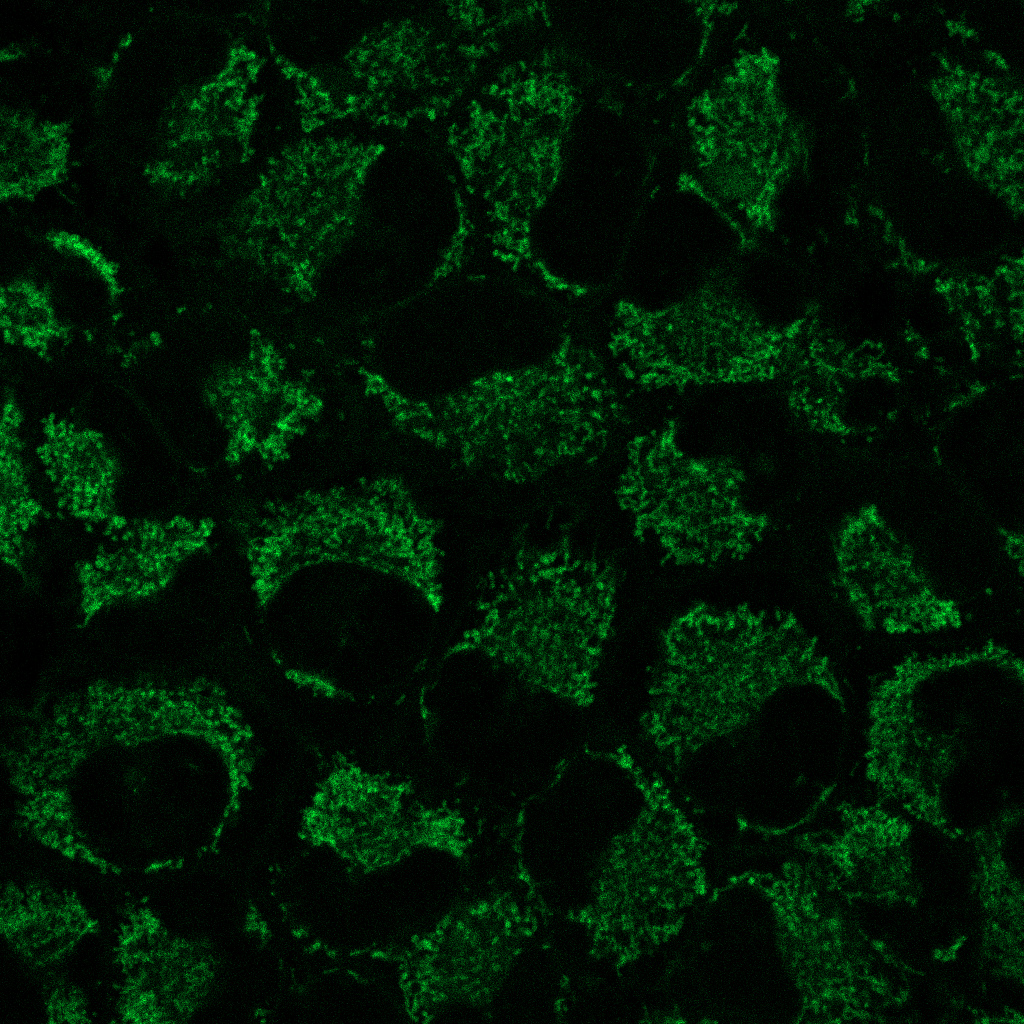

Supplement: Supplementary file 3 — Source data [file 41467_2022_35472_MOESM3_ESM.zip › Fig S25/4h/4h_c2.tif]

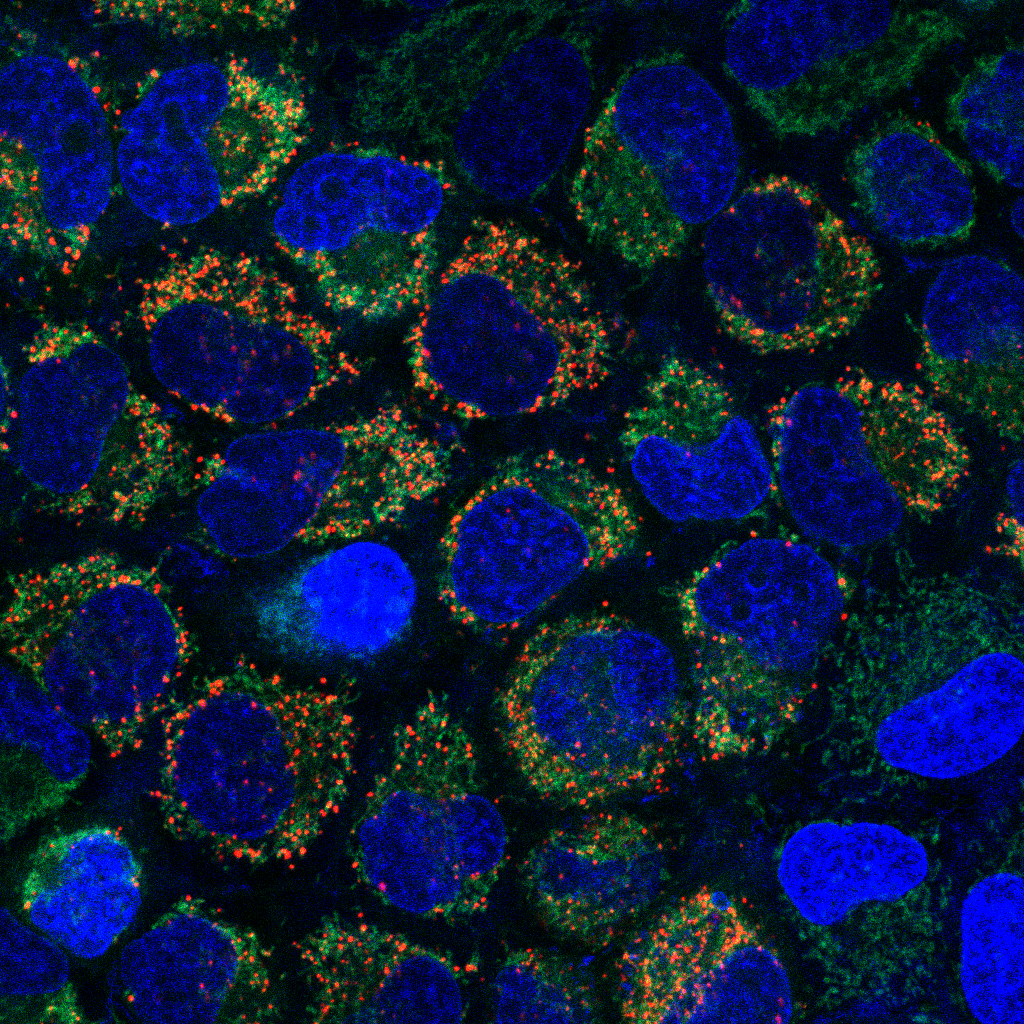

Supplement: Supplementary file 3 — Source data [file 41467_2022_35472_MOESM3_ESM.zip › Fig S25/8h/8h_c1-3.tif]

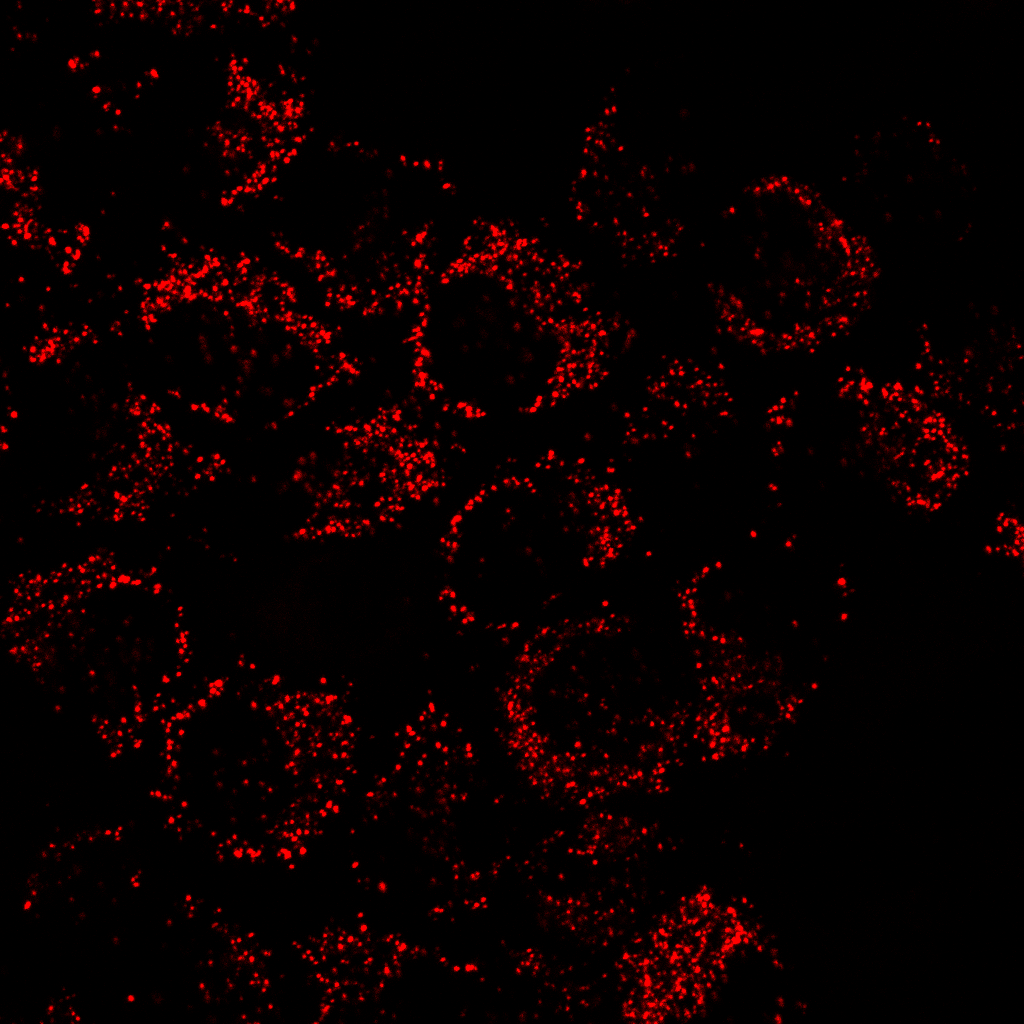

Supplement: Supplementary file 3 — Source data [file 41467_2022_35472_MOESM3_ESM.zip › Fig S25/8h/8h_c1.tif]

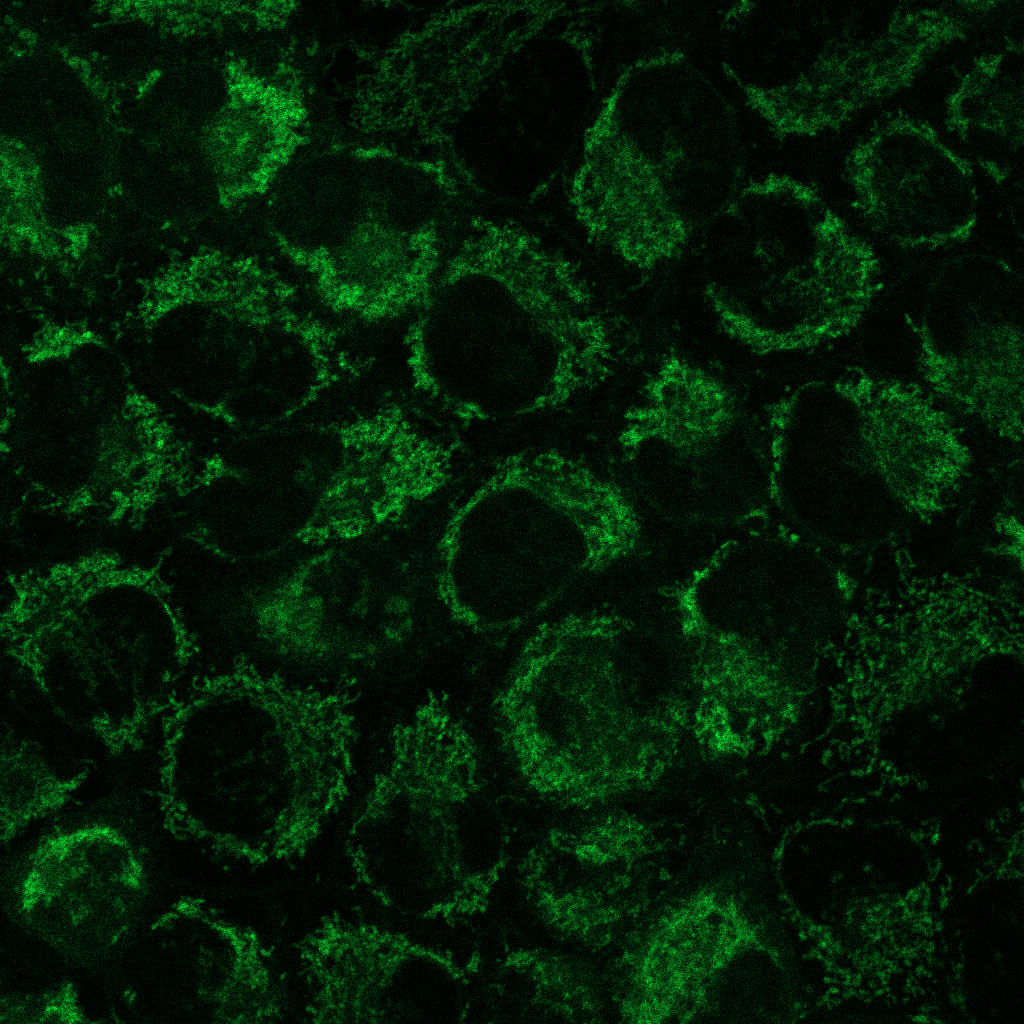

Supplement: Supplementary file 3 — Source data [file 41467_2022_35472_MOESM3_ESM.zip › Fig S25/8h/8h_c2.tif]

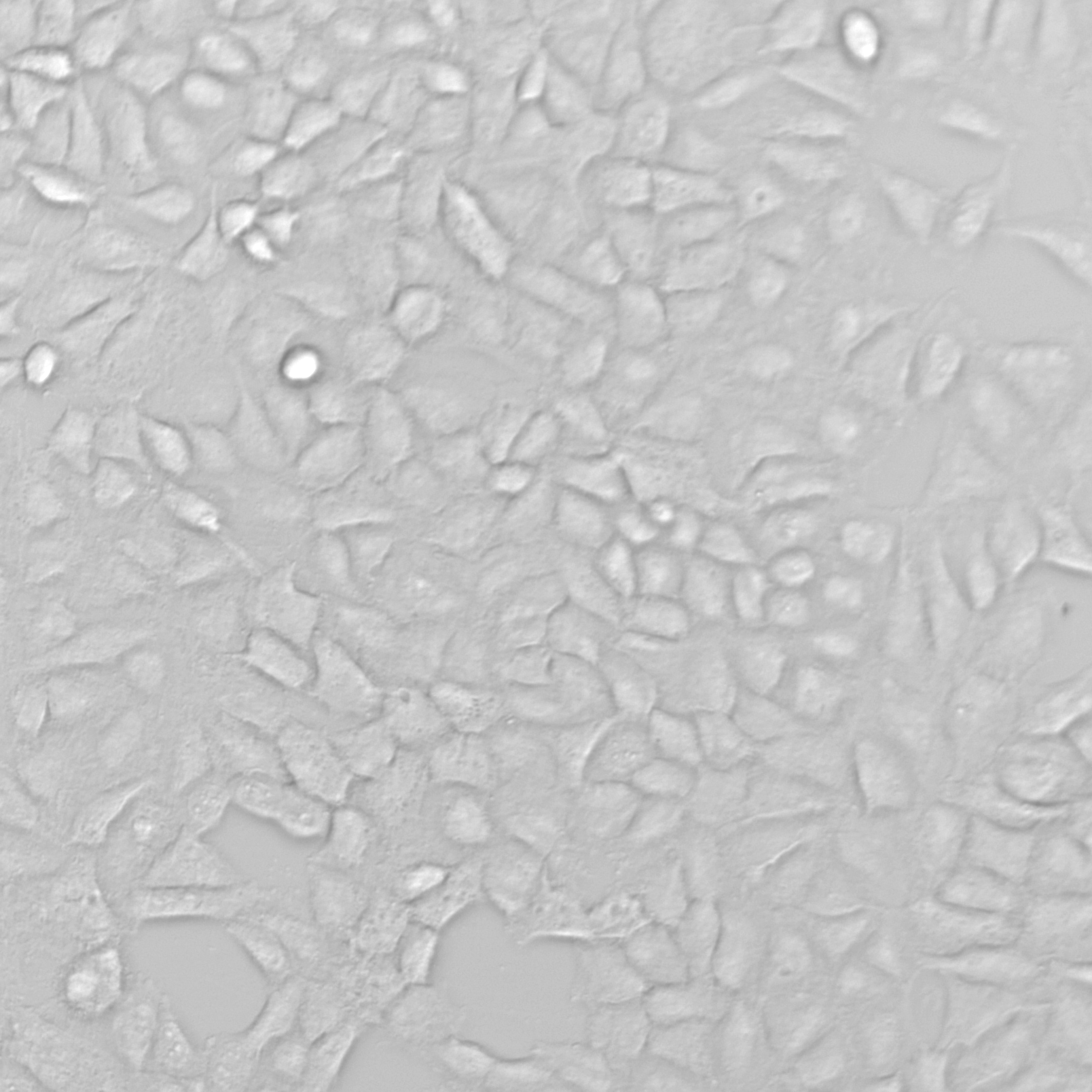

Supplement: Supplementary file 3 — Source data [file 41467_2022_35472_MOESM3_ESM.zip › Fig S26/BF Blank.tif]

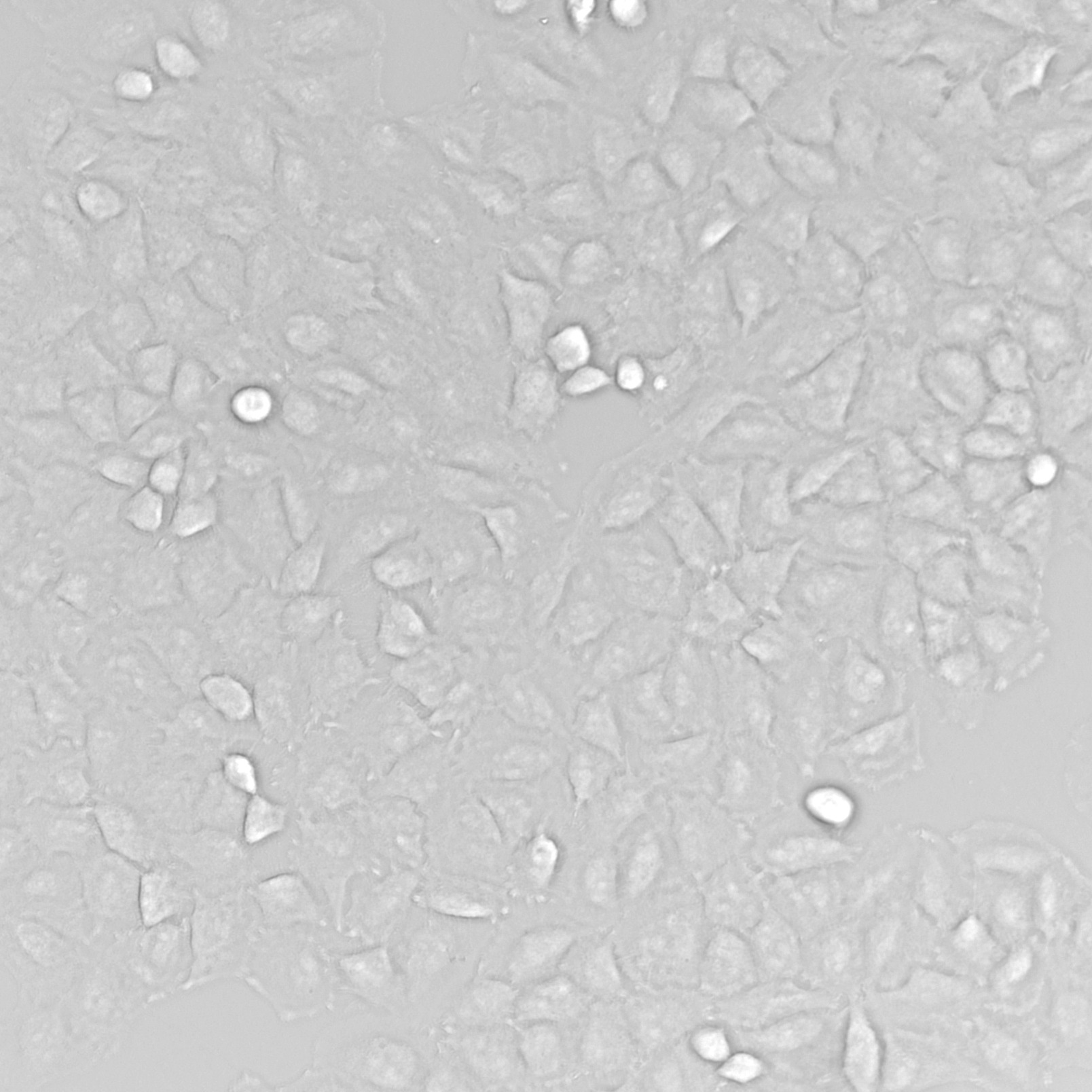

Supplement: Supplementary file 3 — Source data [file 41467_2022_35472_MOESM3_ESM.zip › Fig S26/BF DAS.tif]

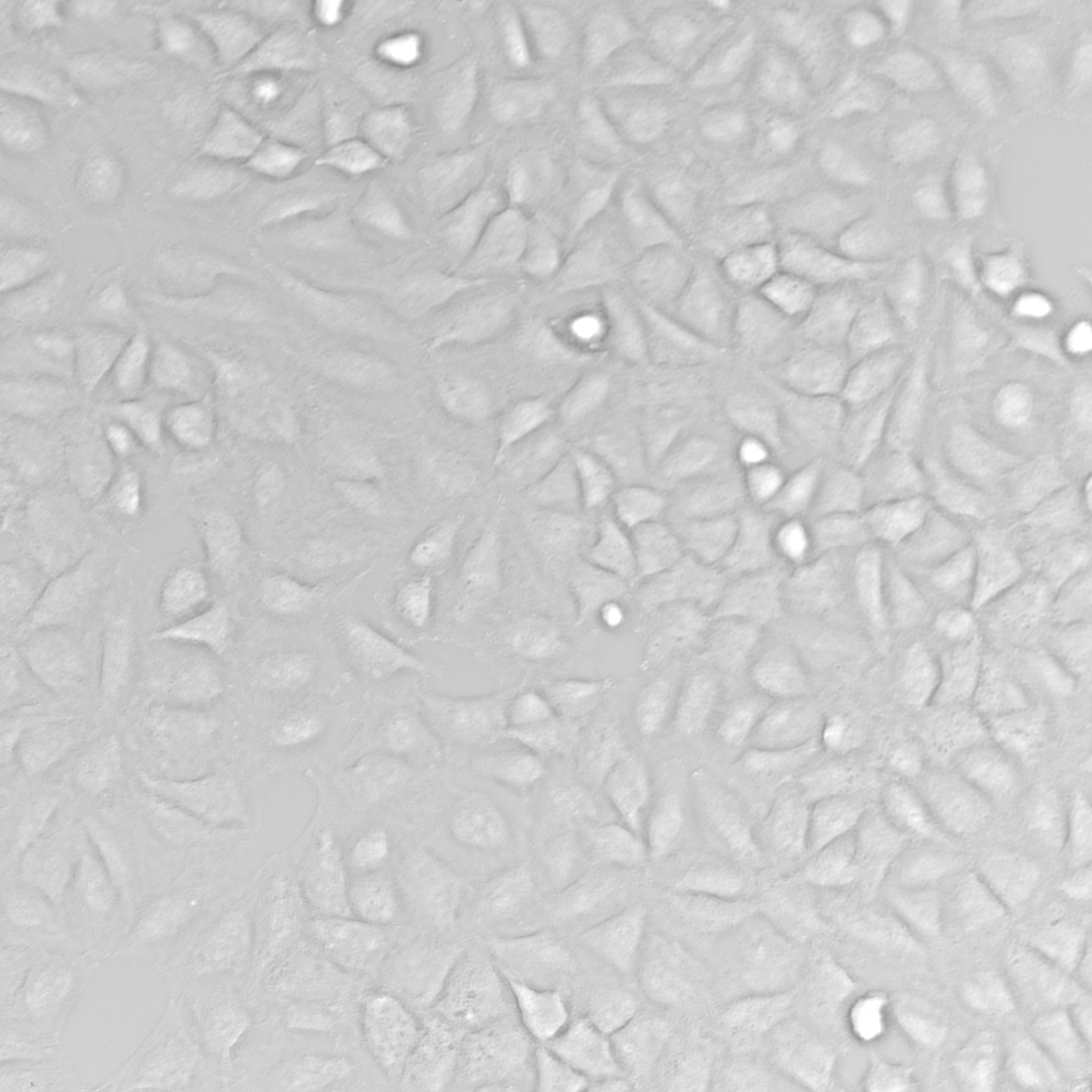

Supplement: Supplementary file 3 — Source data [file 41467_2022_35472_MOESM3_ESM.zip › Fig S26/BF DCNC.tif]

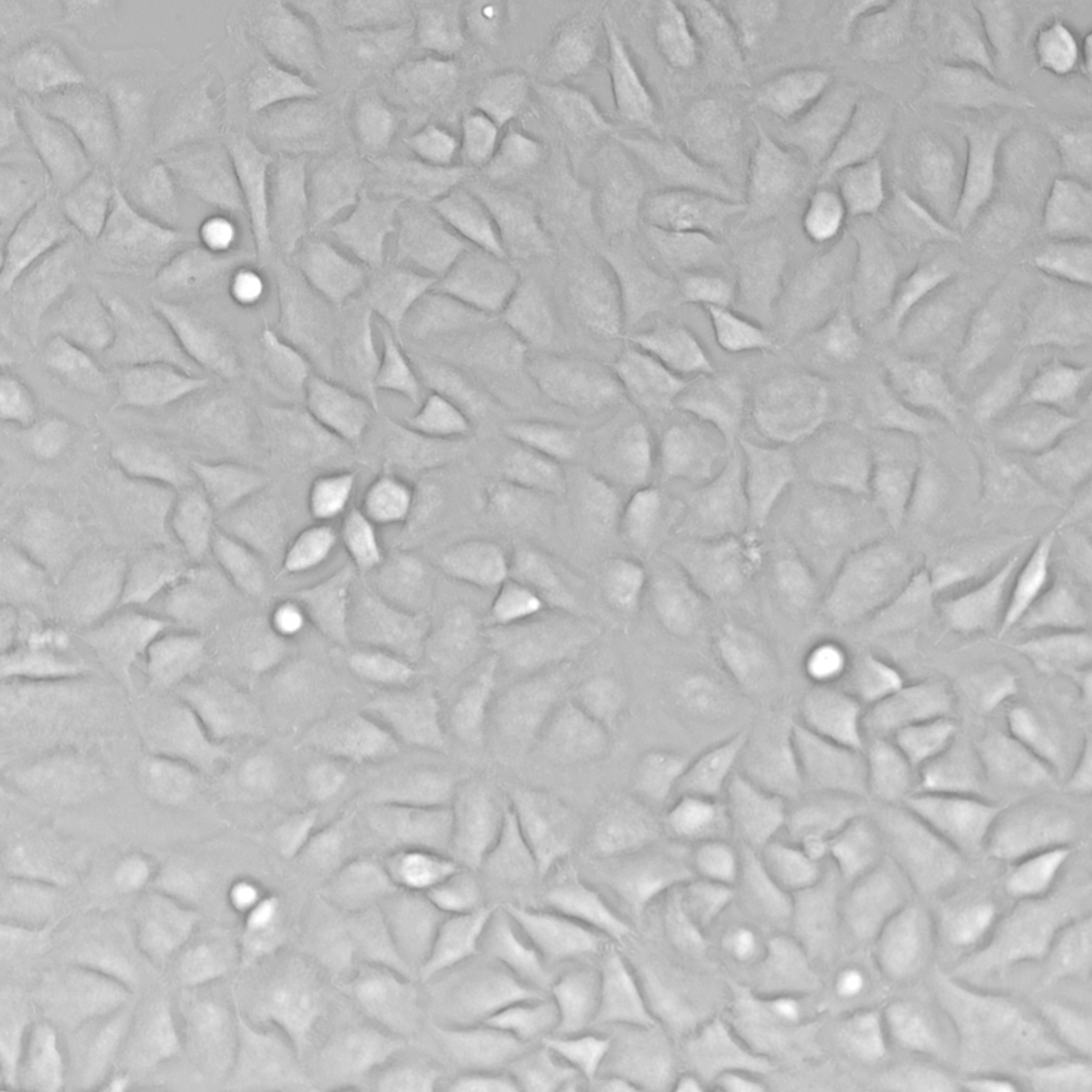

Supplement: Supplementary file 3 — Source data [file 41467_2022_35472_MOESM3_ESM.zip › Fig S26/BF DniAS.tif]

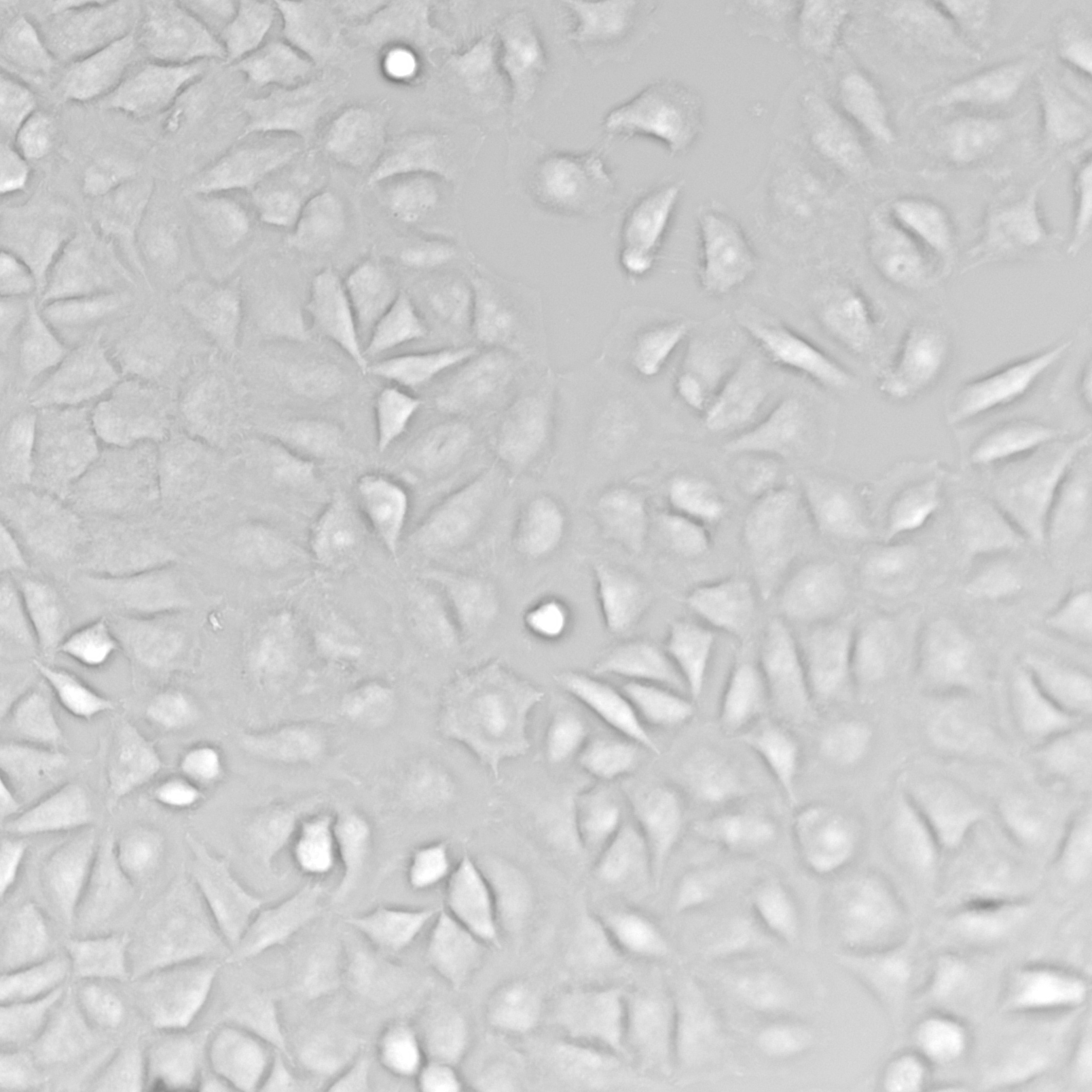

Supplement: Supplementary file 3 — Source data [file 41467_2022_35472_MOESM3_ESM.zip › Fig S26/BF DniCNC.tif]

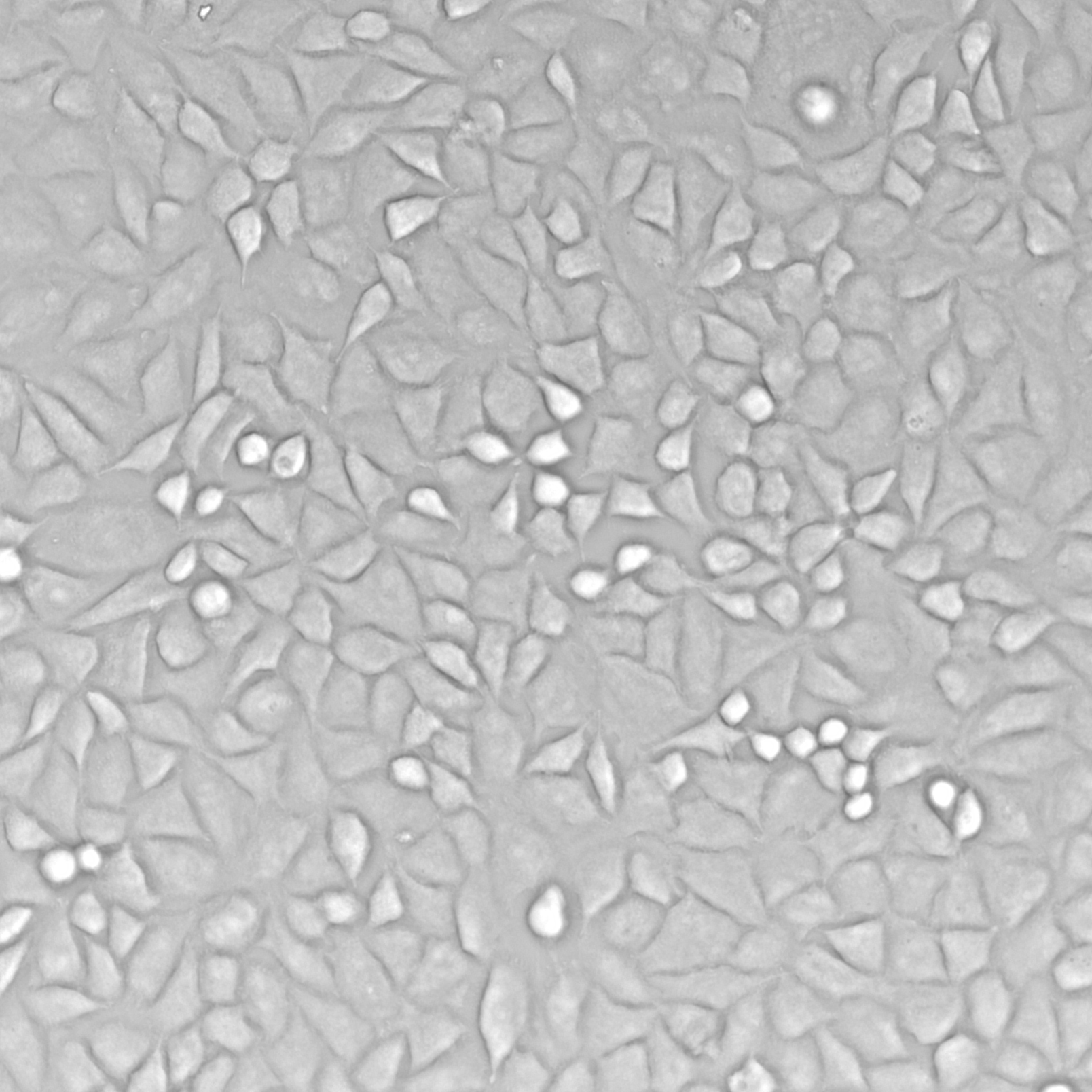

Supplement: Supplementary file 3 — Source data [file 41467_2022_35472_MOESM3_ESM.zip › Fig S26/PBS-B.tif]

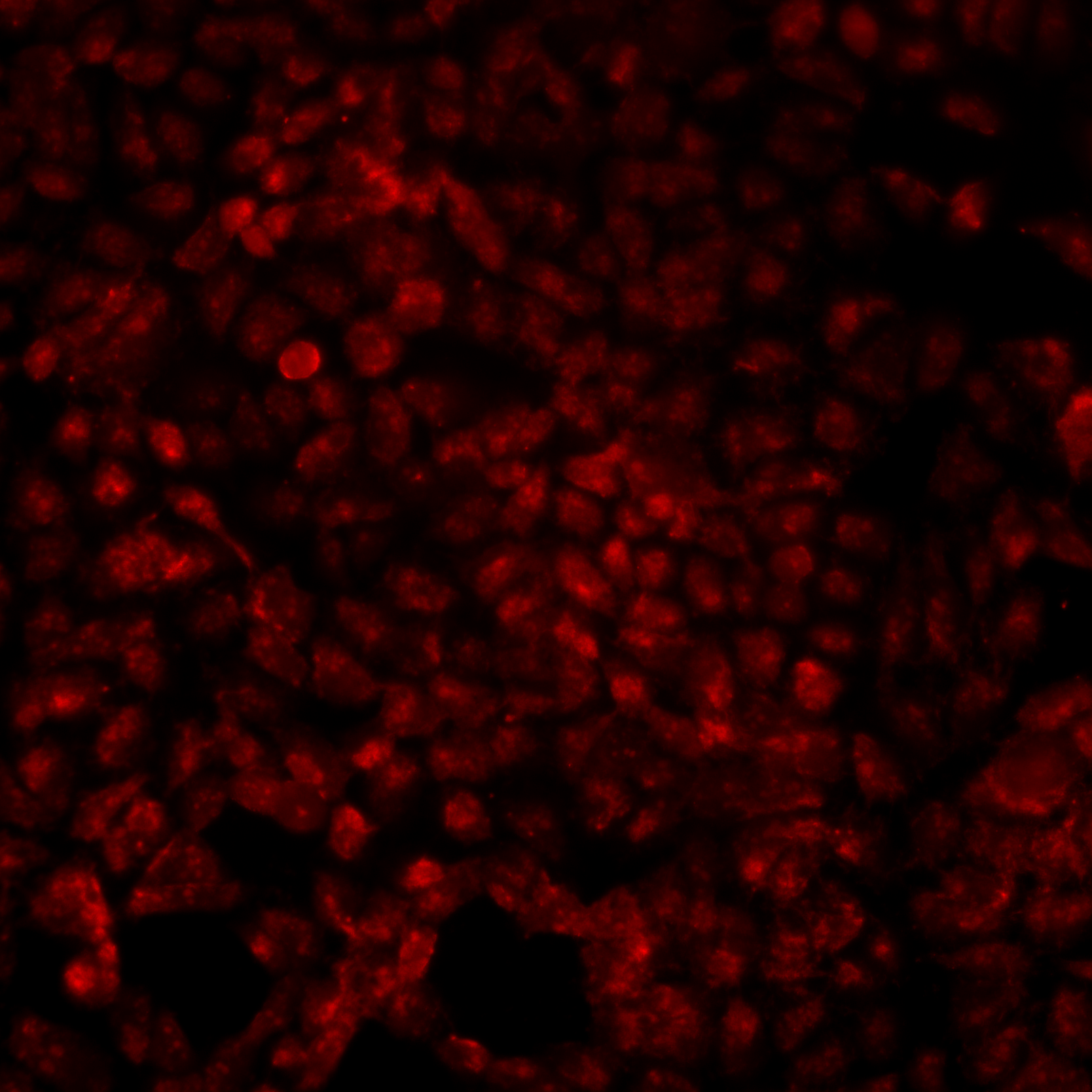

Supplement: Supplementary file 3 — Source data [file 41467_2022_35472_MOESM3_ESM.zip › Fig S26/Rhod-2 Blank.tif]

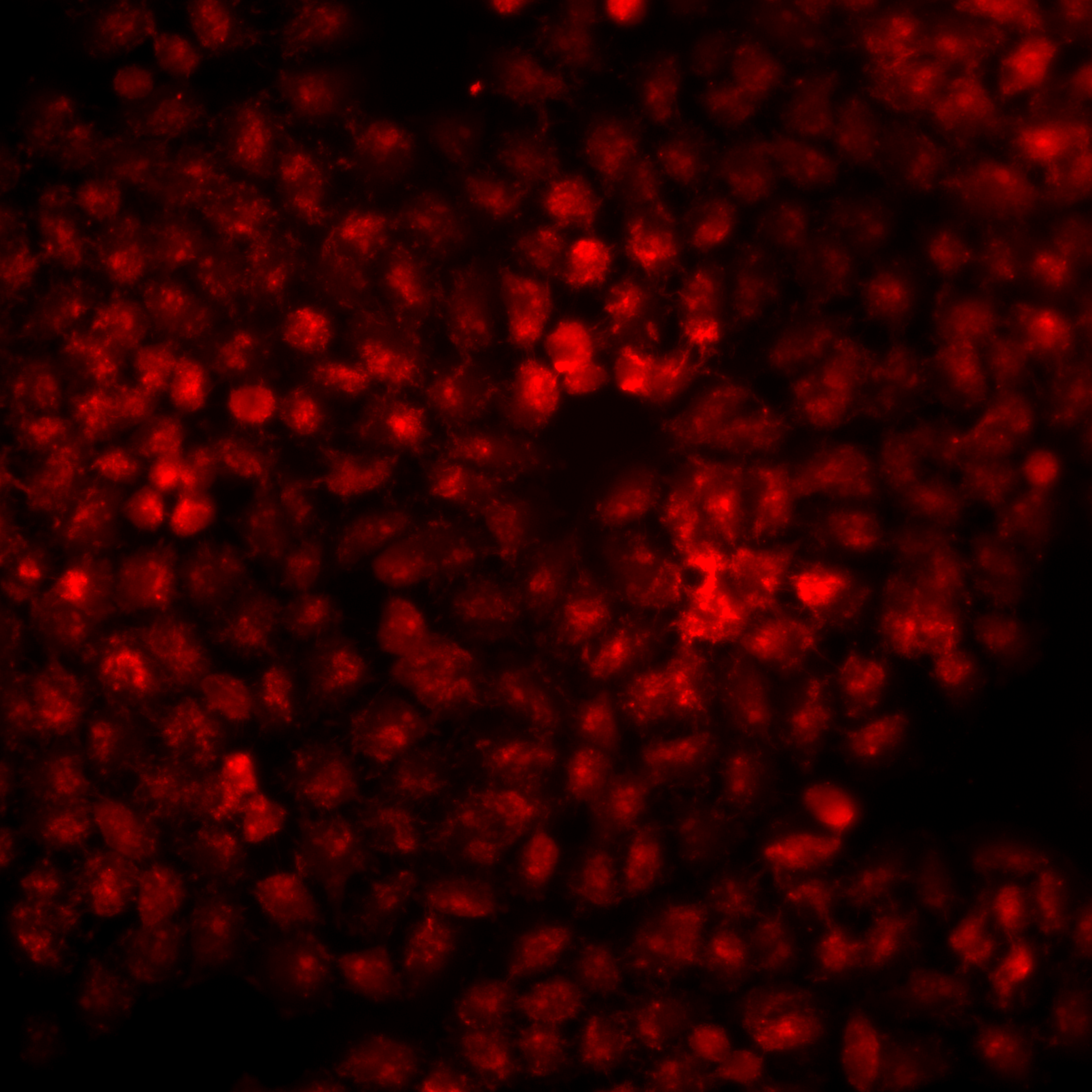

Supplement: Supplementary file 3 — Source data [file 41467_2022_35472_MOESM3_ESM.zip › Fig S26/Rhod-2 DAS.tif]

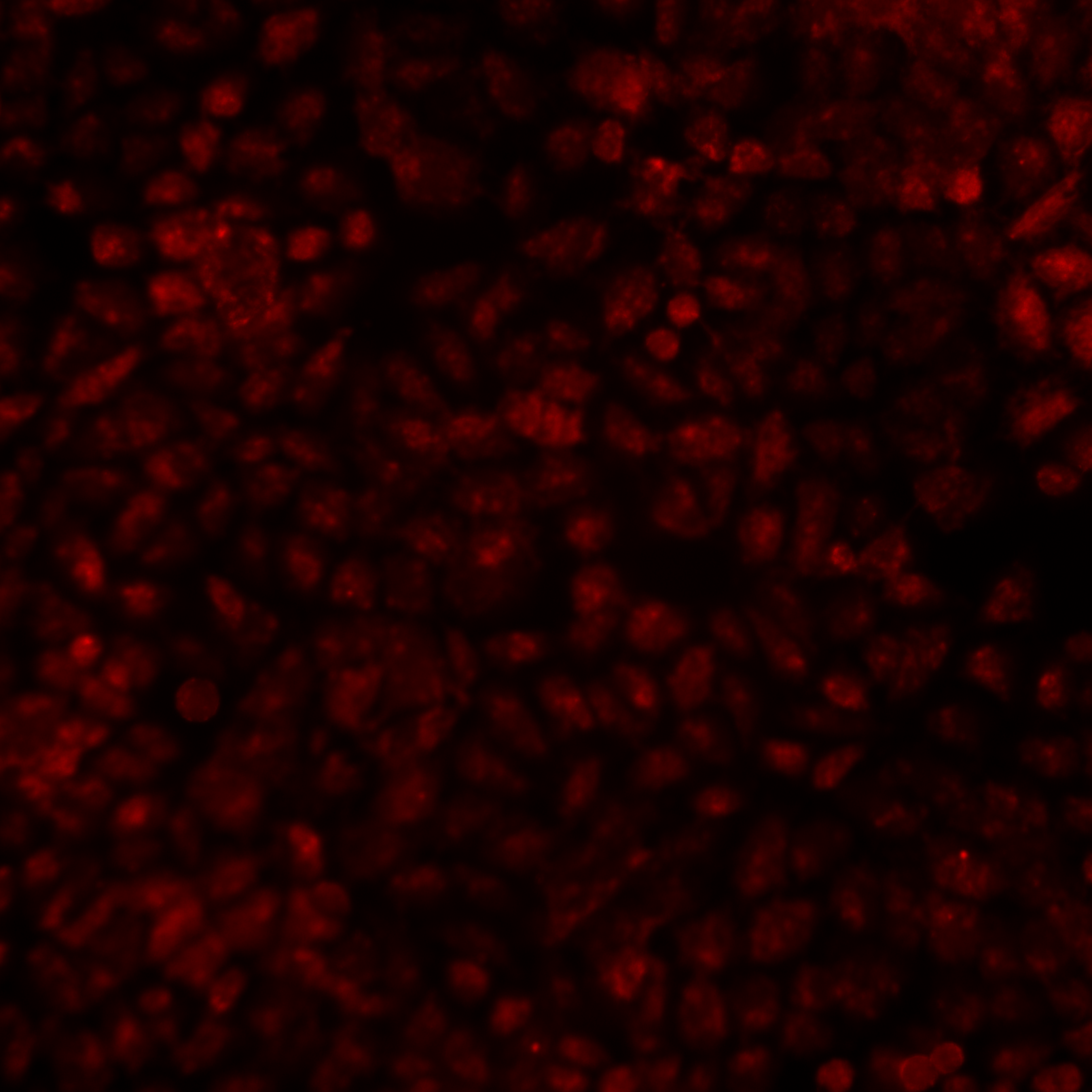

Supplement: Supplementary file 3 — Source data [file 41467_2022_35472_MOESM3_ESM.zip › Fig S26/Rhod-2 DCNC.tif]

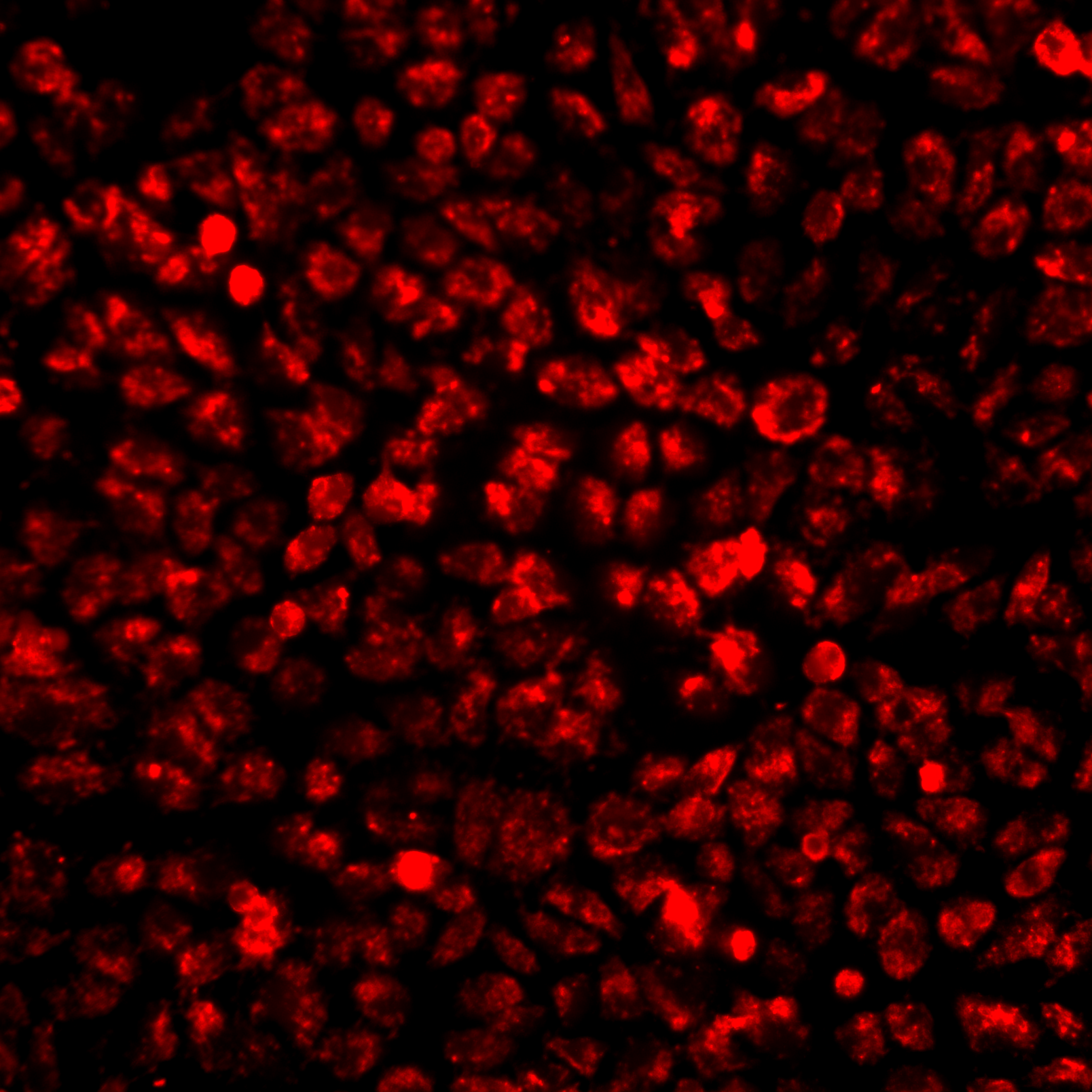

Supplement: Supplementary file 3 — Source data [file 41467_2022_35472_MOESM3_ESM.zip › Fig S26/Rhod-2 DniAS.tif]

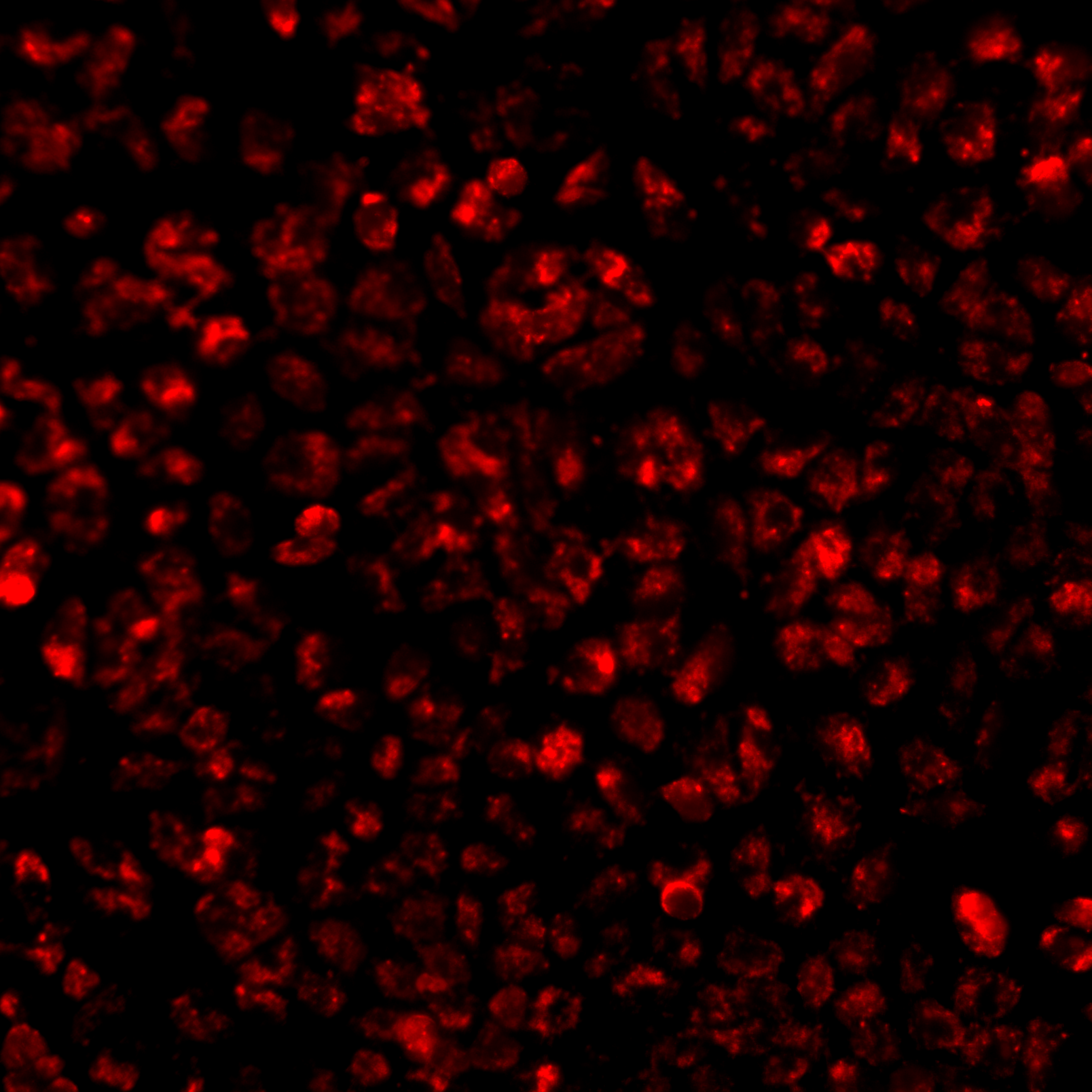

Supplement: Supplementary file 3 — Source data [file 41467_2022_35472_MOESM3_ESM.zip › Fig S26/Rhod-2 DniCNC.tif]
