# Supplementary figures and images for: Dynamic assembly of DNA-ceria nanocomplex in living cells generates artificial peroxisome (part 1 of 2)
Source: Nat Commun. 2022 Dec 14;13:7739. doi: 10.1038/s41467-022-35472-2 (PMC9751304; doi:10.1038/s41467-022-35472-2)

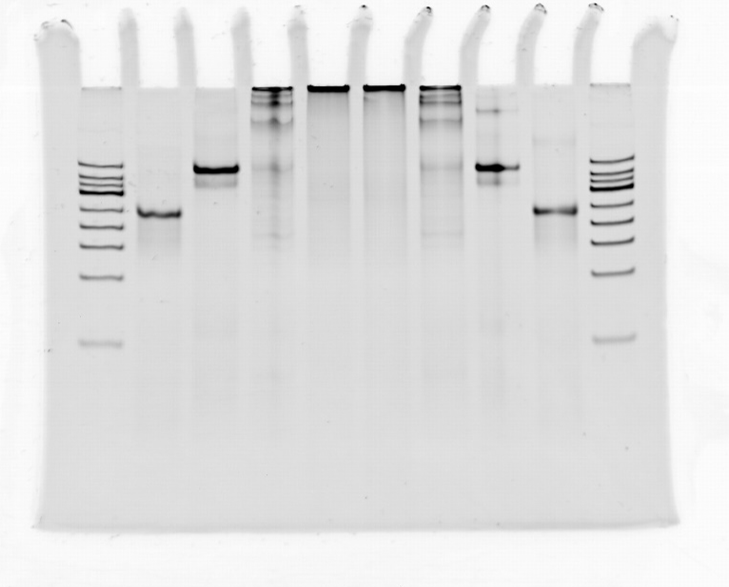

Supplement: Supplementary file 3 — Source data [file 41467_2022_35472_MOESM3_ESM.zip › Fig 2a.tif]

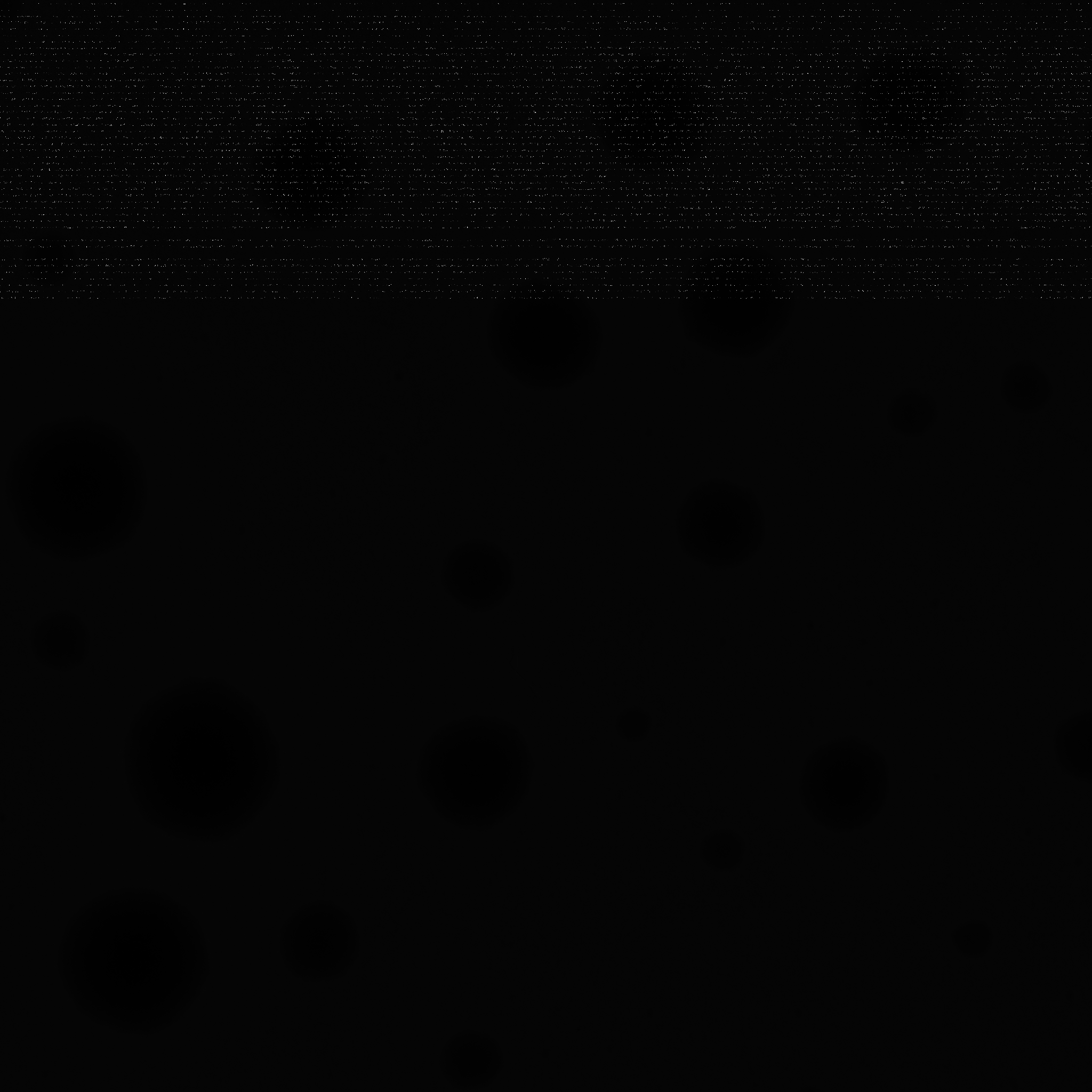

Supplement: Supplementary file 3 — Source data [file 41467_2022_35472_MOESM3_ESM.zip › Fig 2d.tif]

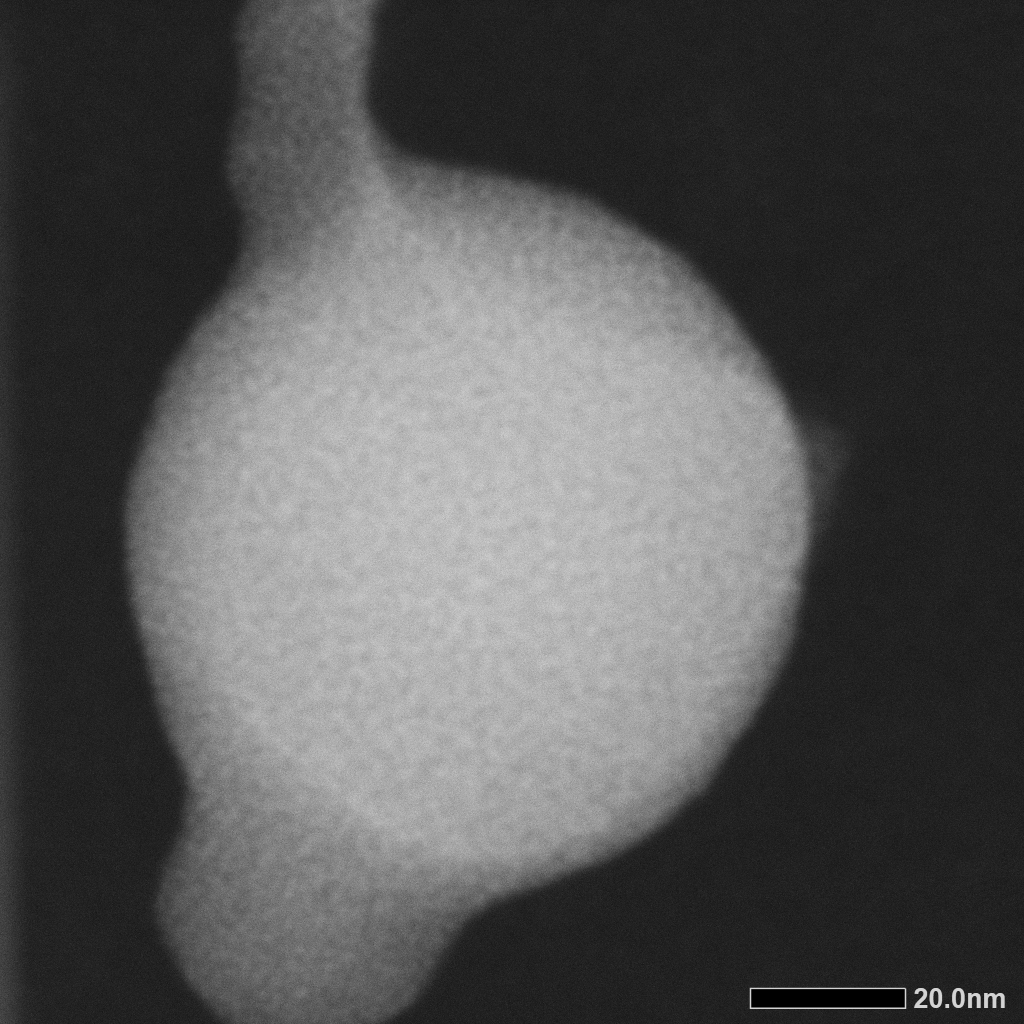

Supplement: Supplementary file 3 — Source data [file 41467_2022_35472_MOESM3_ESM.zip › Fig 2e/JEM-F200_200.00kV_1_x1.5M_150mm_DFUpper_15.bmp]

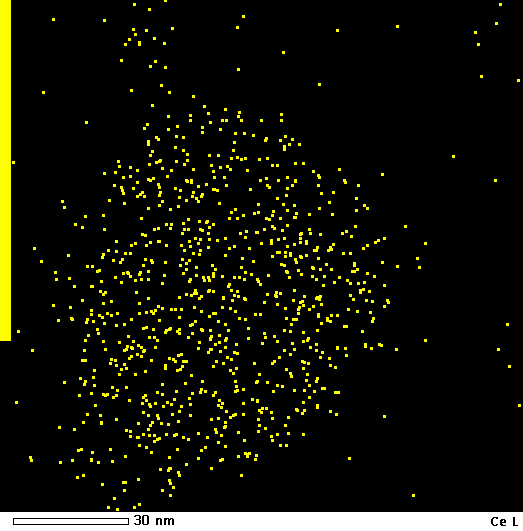

Supplement: Supplementary file 3 — Source data [file 41467_2022_35472_MOESM3_ESM.zip › Fig 2e/View000 Ce L.bmp]

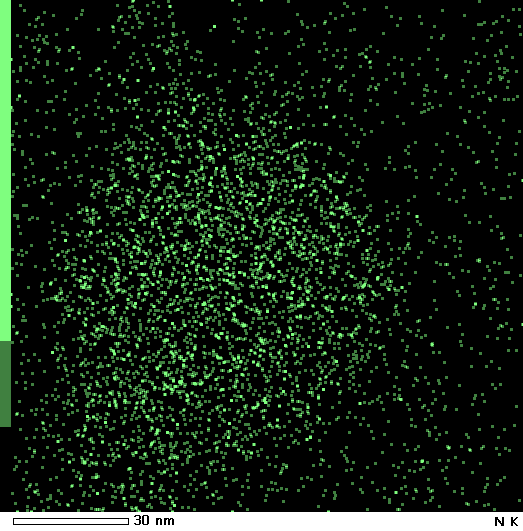

Supplement: Supplementary file 3 — Source data [file 41467_2022_35472_MOESM3_ESM.zip › Fig 2e/View000 N K.bmp]

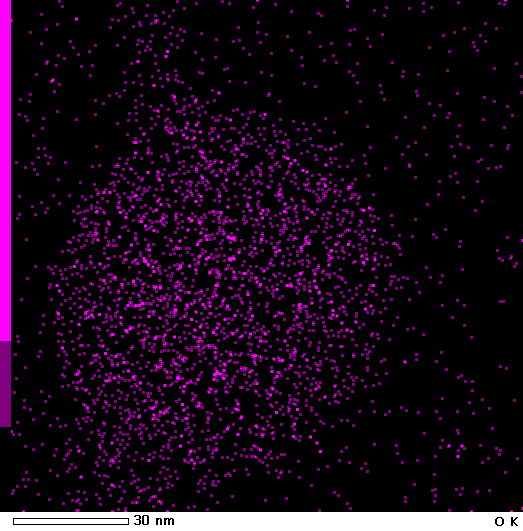

Supplement: Supplementary file 3 — Source data [file 41467_2022_35472_MOESM3_ESM.zip › Fig 2e/View000 O K.bmp]

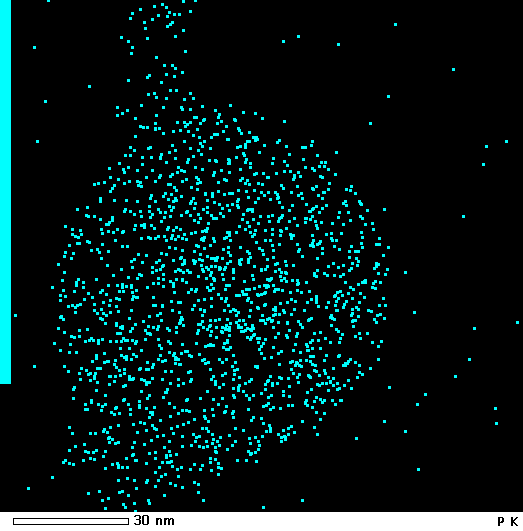

Supplement: Supplementary file 3 — Source data [file 41467_2022_35472_MOESM3_ESM.zip › Fig 2e/View000 P K.bmp]

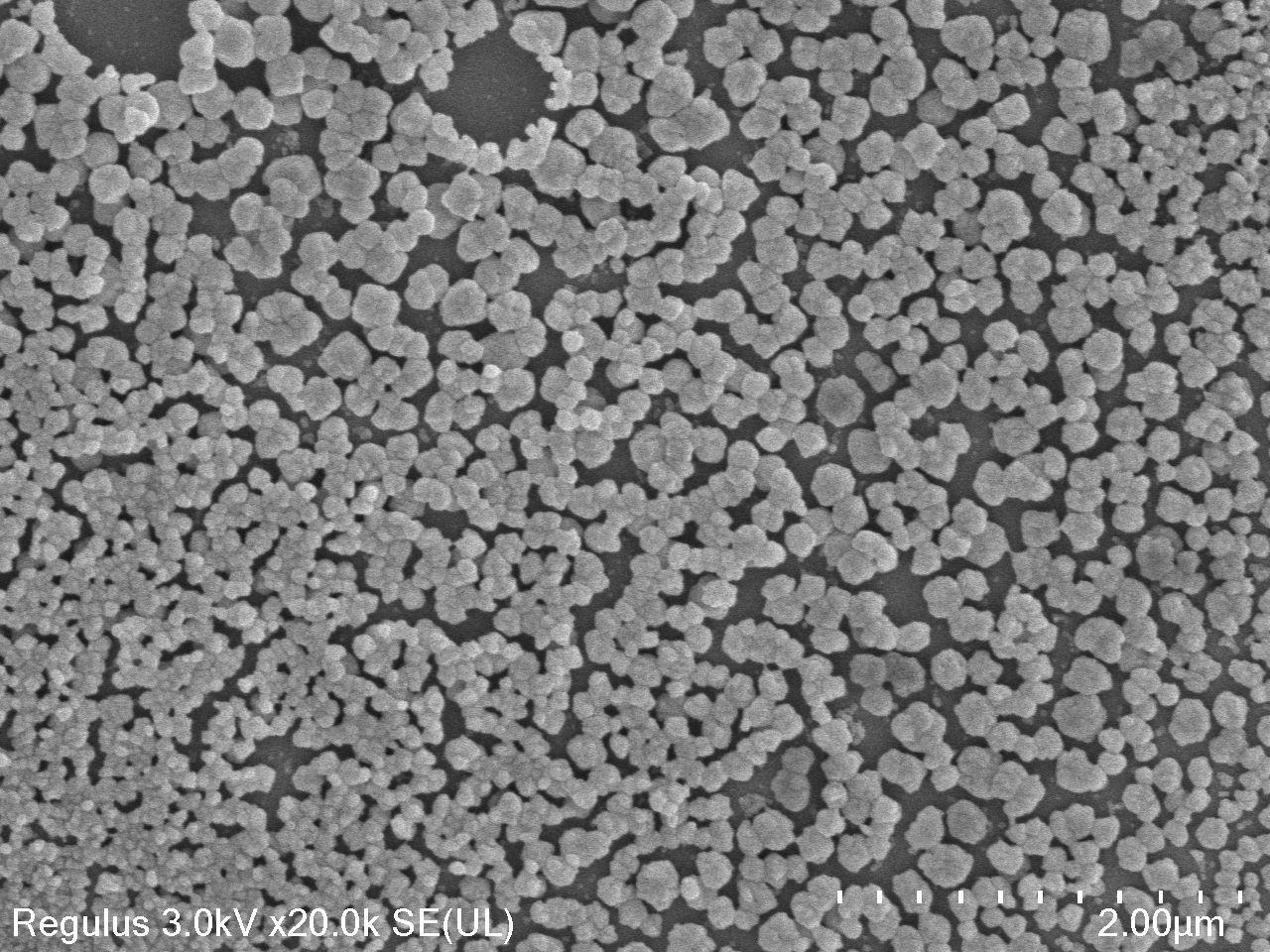

Supplement: Supplementary file 3 — Source data [file 41467_2022_35472_MOESM3_ESM.zip › Fig 2h.tif]

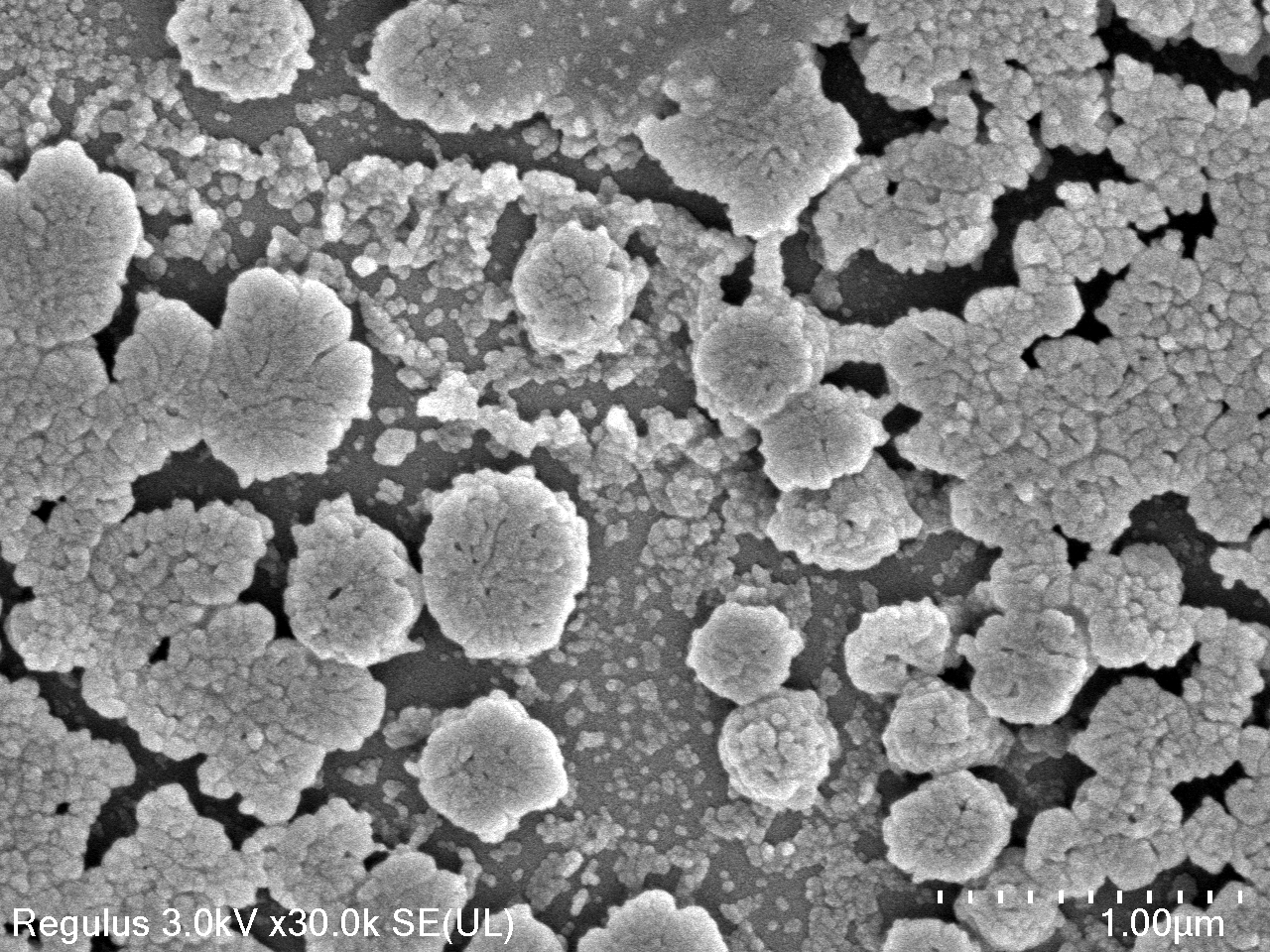

Supplement: Supplementary file 3 — Source data [file 41467_2022_35472_MOESM3_ESM.zip › Fig 2i.tif]

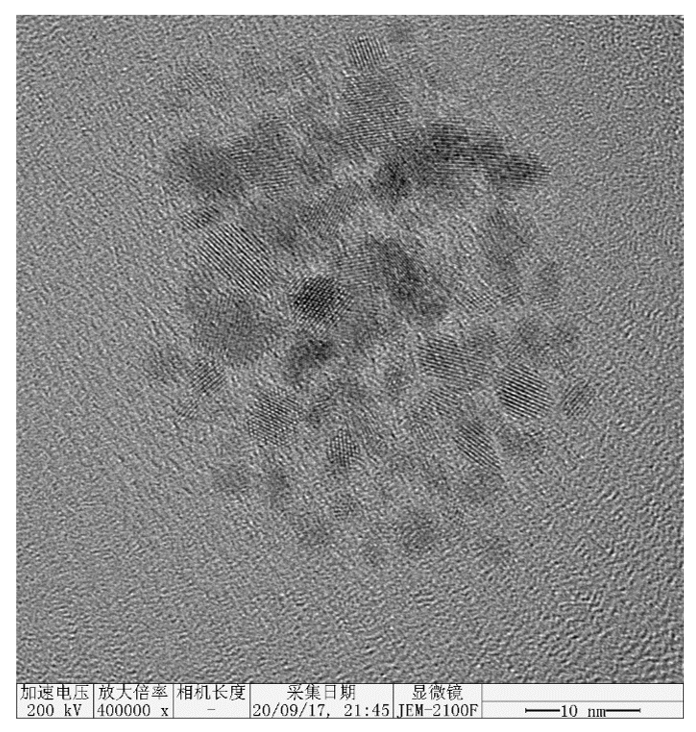

Supplement: Supplementary file 3 — Source data [file 41467_2022_35472_MOESM3_ESM.zip › Fig 3b&S2/TEM.tif]

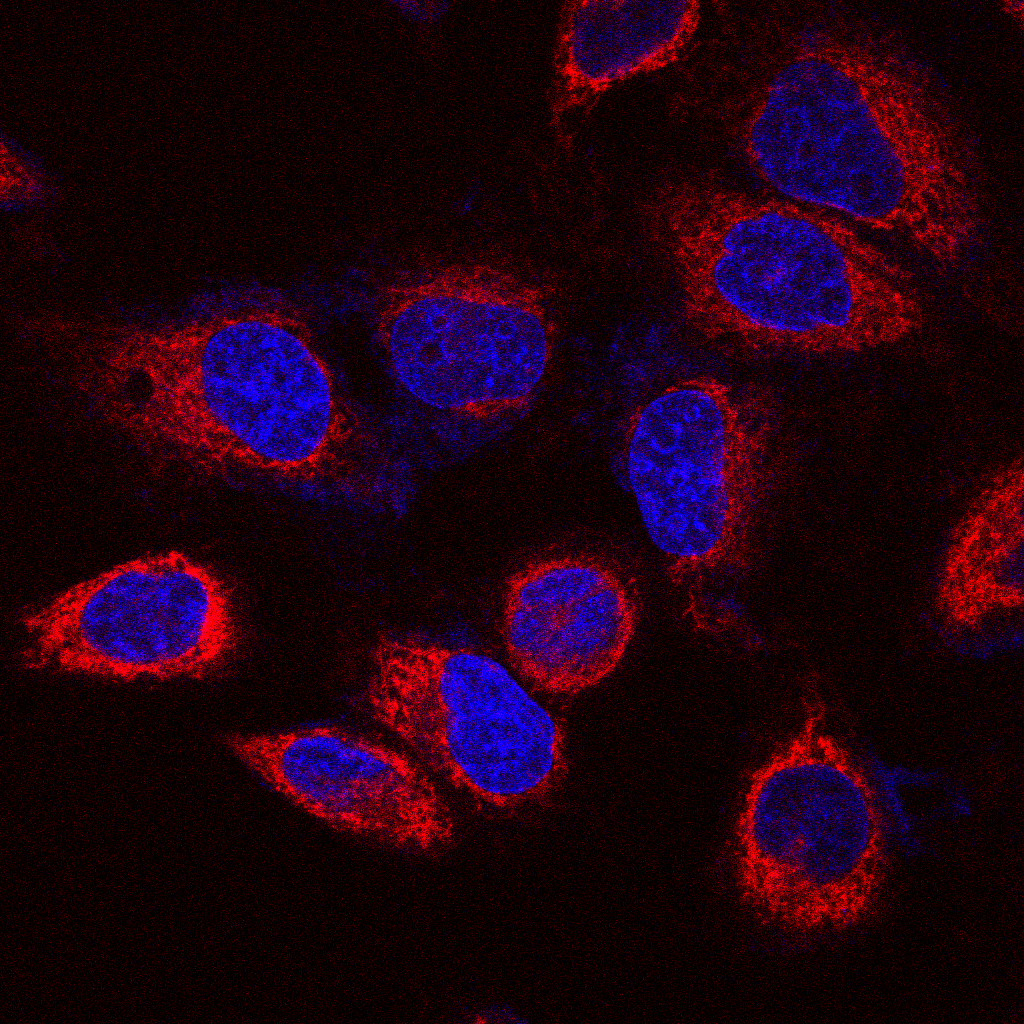

Supplement: Supplementary file 3 — Source data [file 41467_2022_35472_MOESM3_ESM.zip › Fig 4a/37°C.tif]

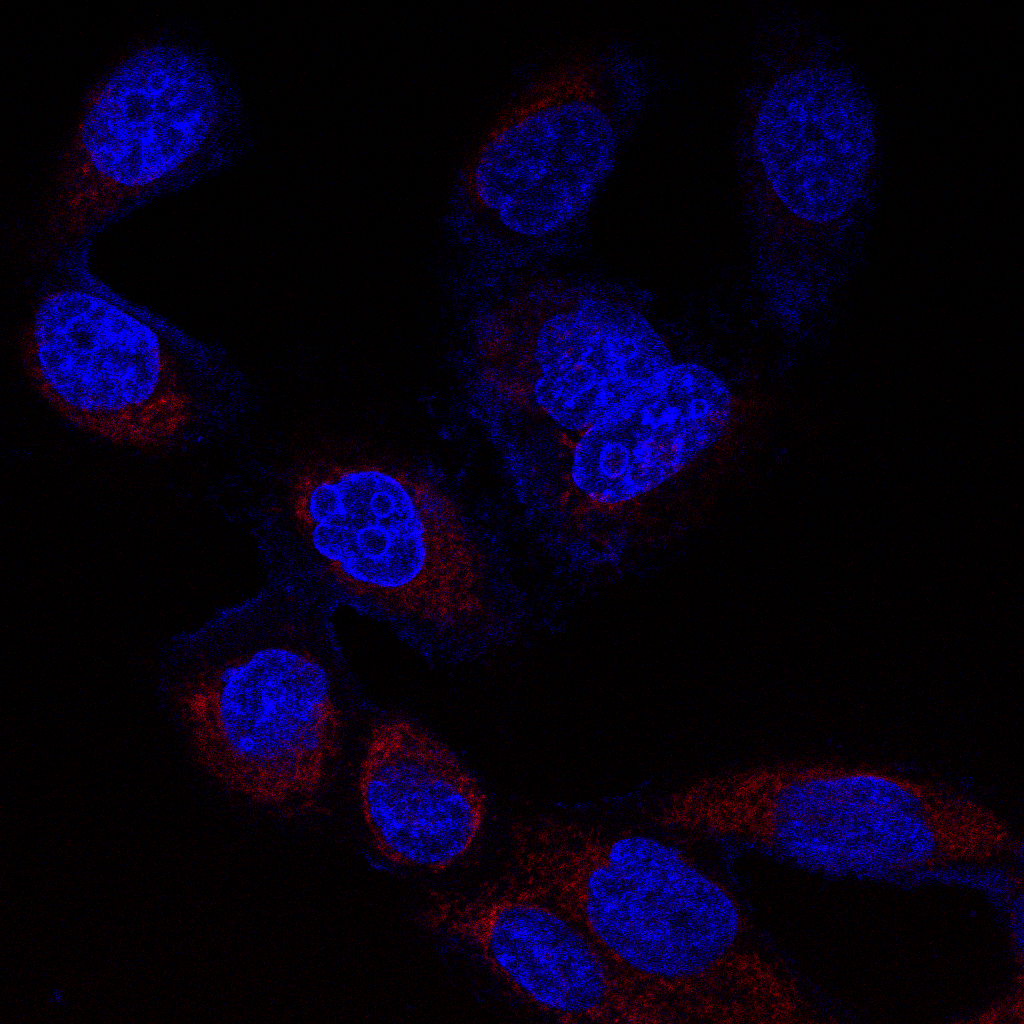

Supplement: Supplementary file 3 — Source data [file 41467_2022_35472_MOESM3_ESM.zip › Fig 4a/4°C.tif]

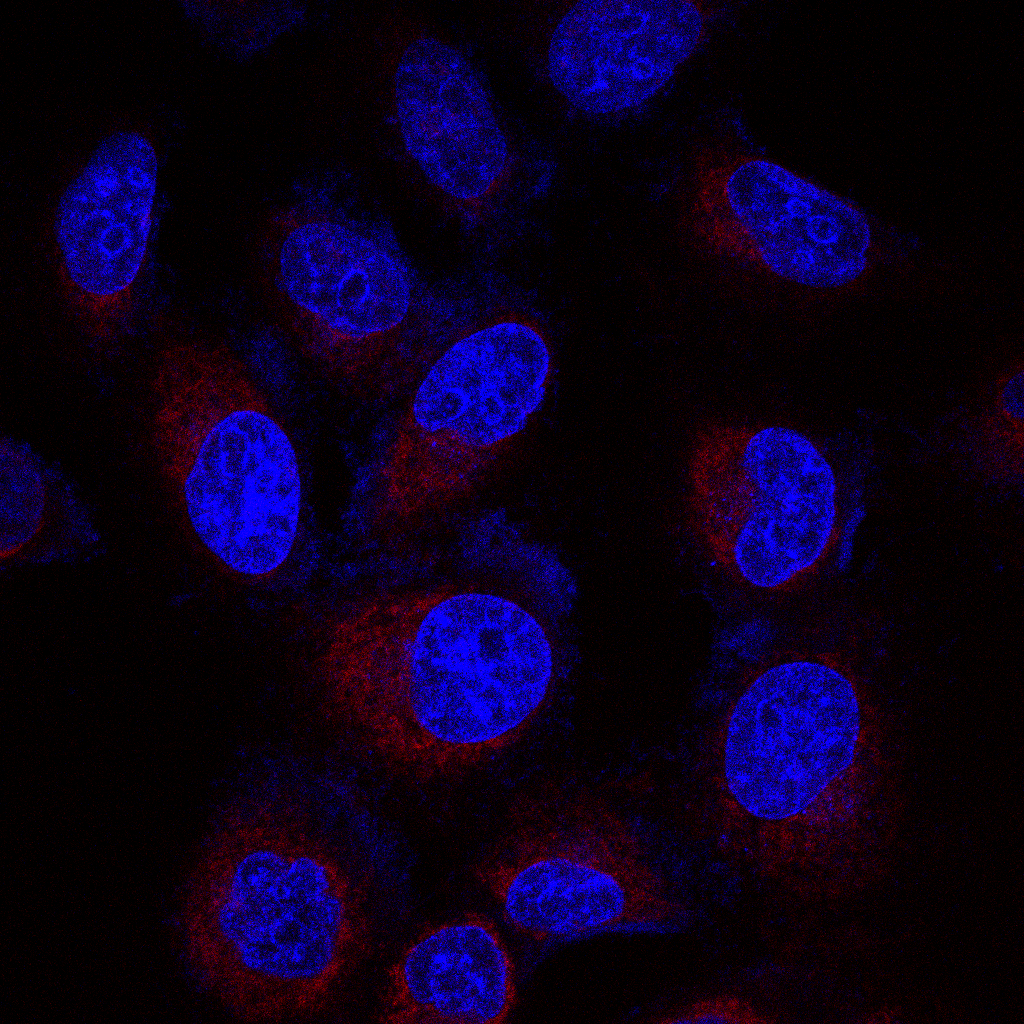

Supplement: Supplementary file 3 — Source data [file 41467_2022_35472_MOESM3_ESM.zip › Fig 4a/Ami.tif]

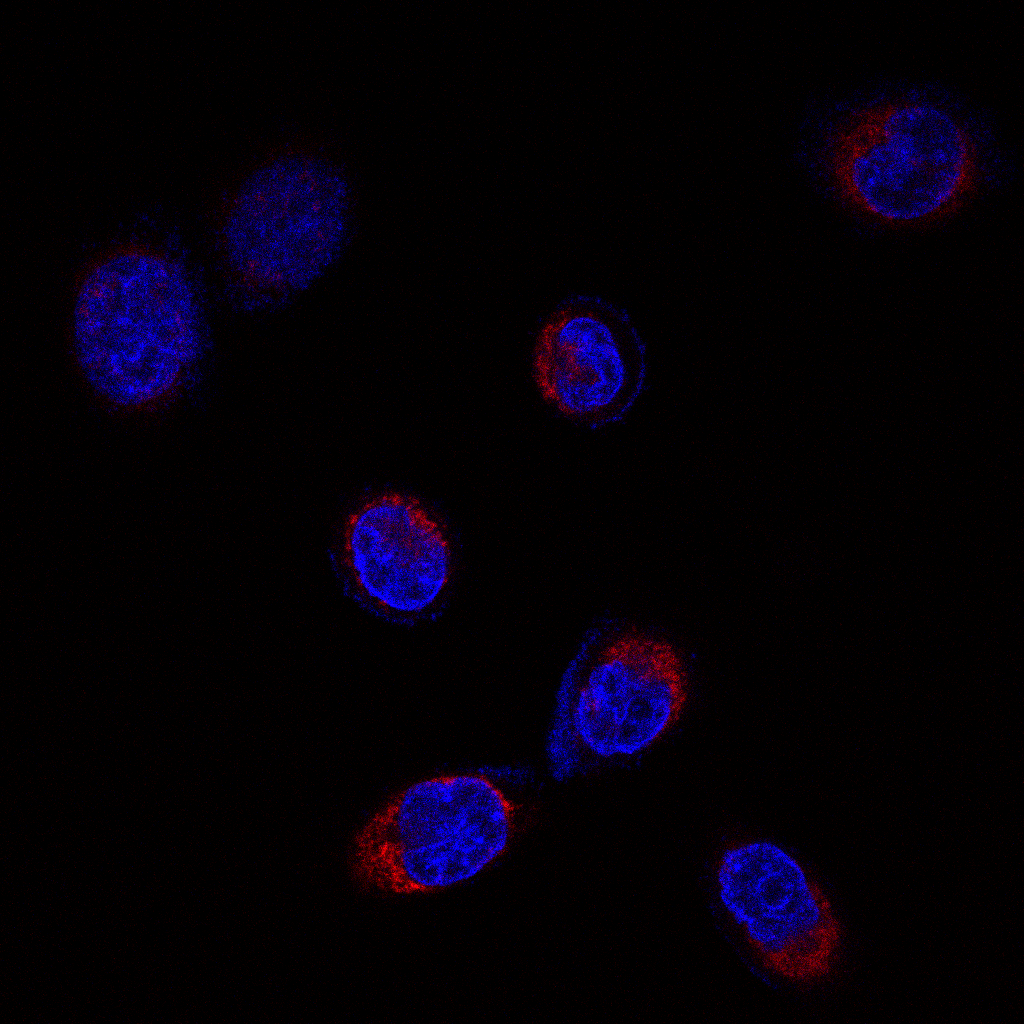

Supplement: Supplementary file 3 — Source data [file 41467_2022_35472_MOESM3_ESM.zip › Fig 4a/Cpz.tif]

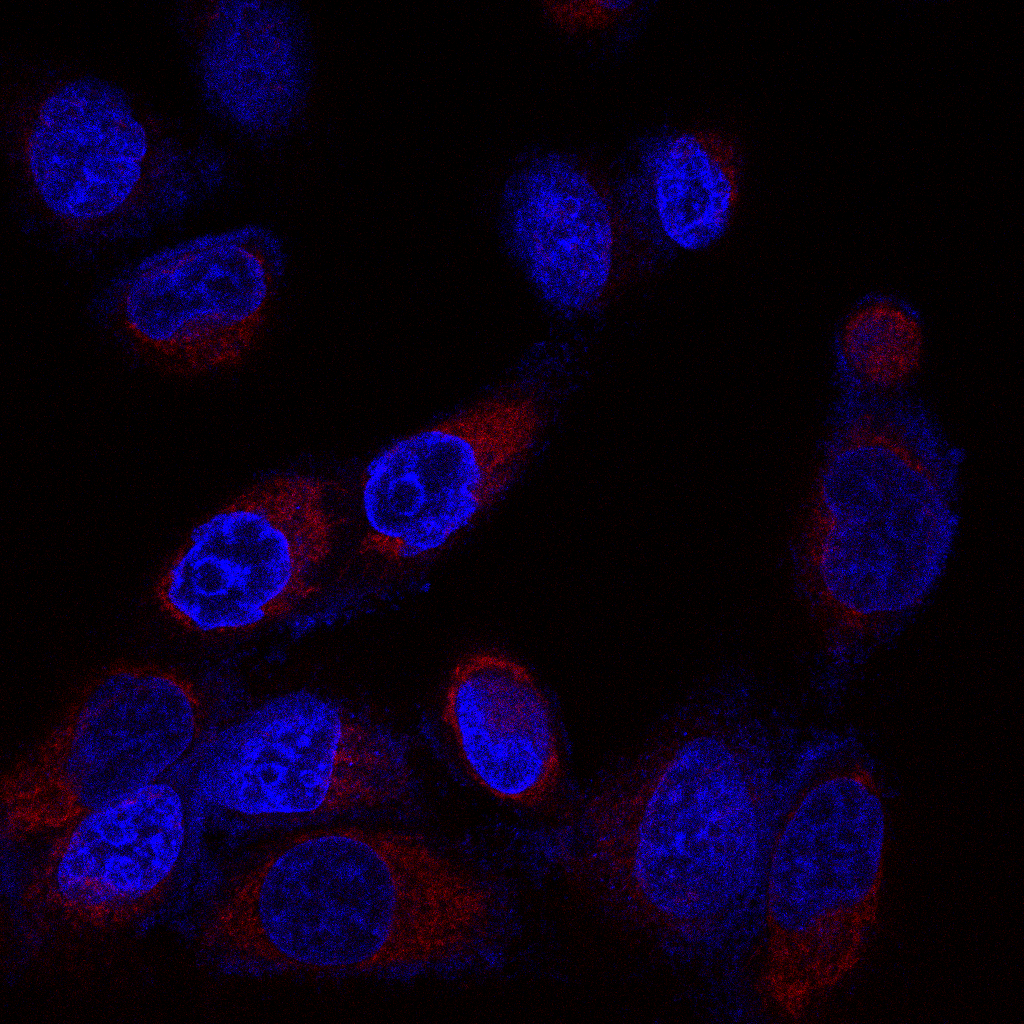

Supplement: Supplementary file 3 — Source data [file 41467_2022_35472_MOESM3_ESM.zip › Fig 4a/Gen.tif]

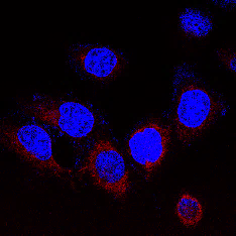

Supplement: Supplementary file 3 — Source data [file 41467_2022_35472_MOESM3_ESM.zip › Fig 4a/Nys.tif]

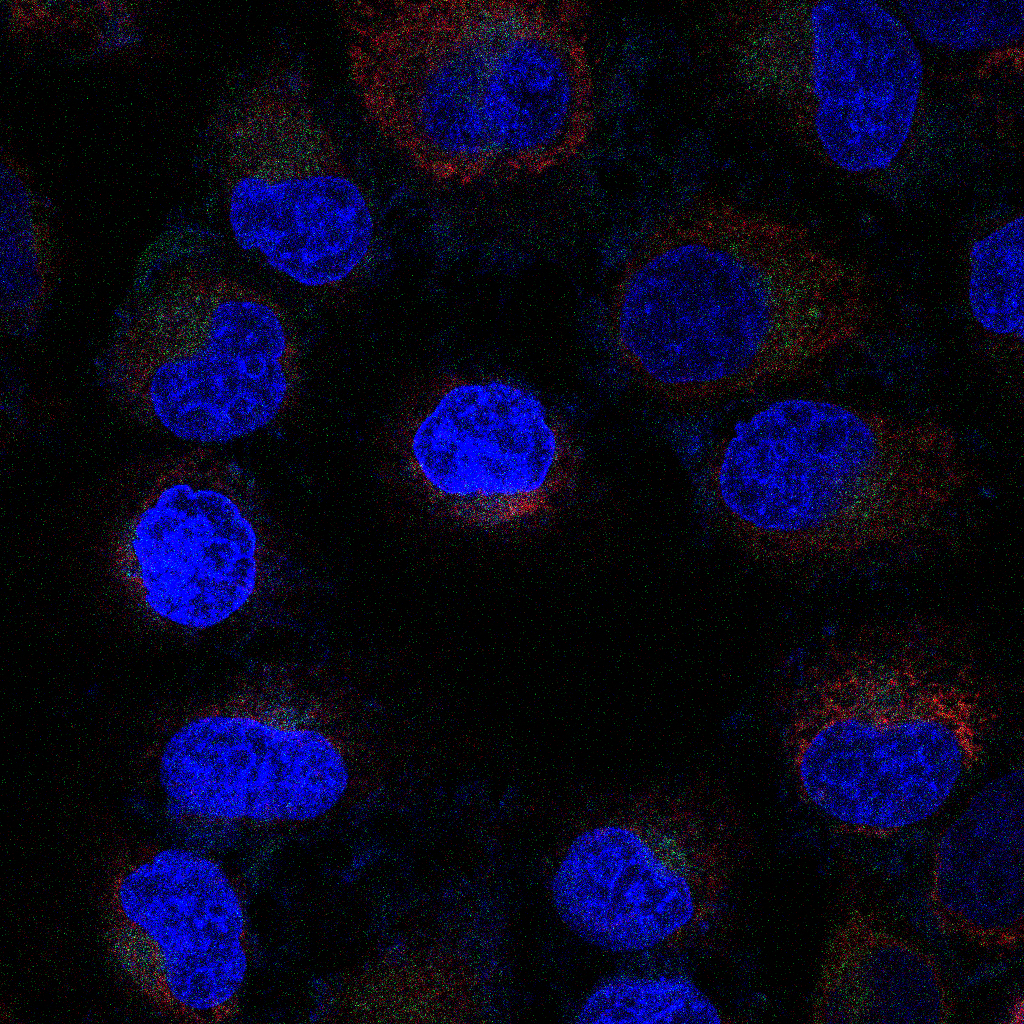

Supplement: Supplementary file 3 — Source data [file 41467_2022_35472_MOESM3_ESM.zip › Fig 4b/2 h/2h_c1-3.tif]

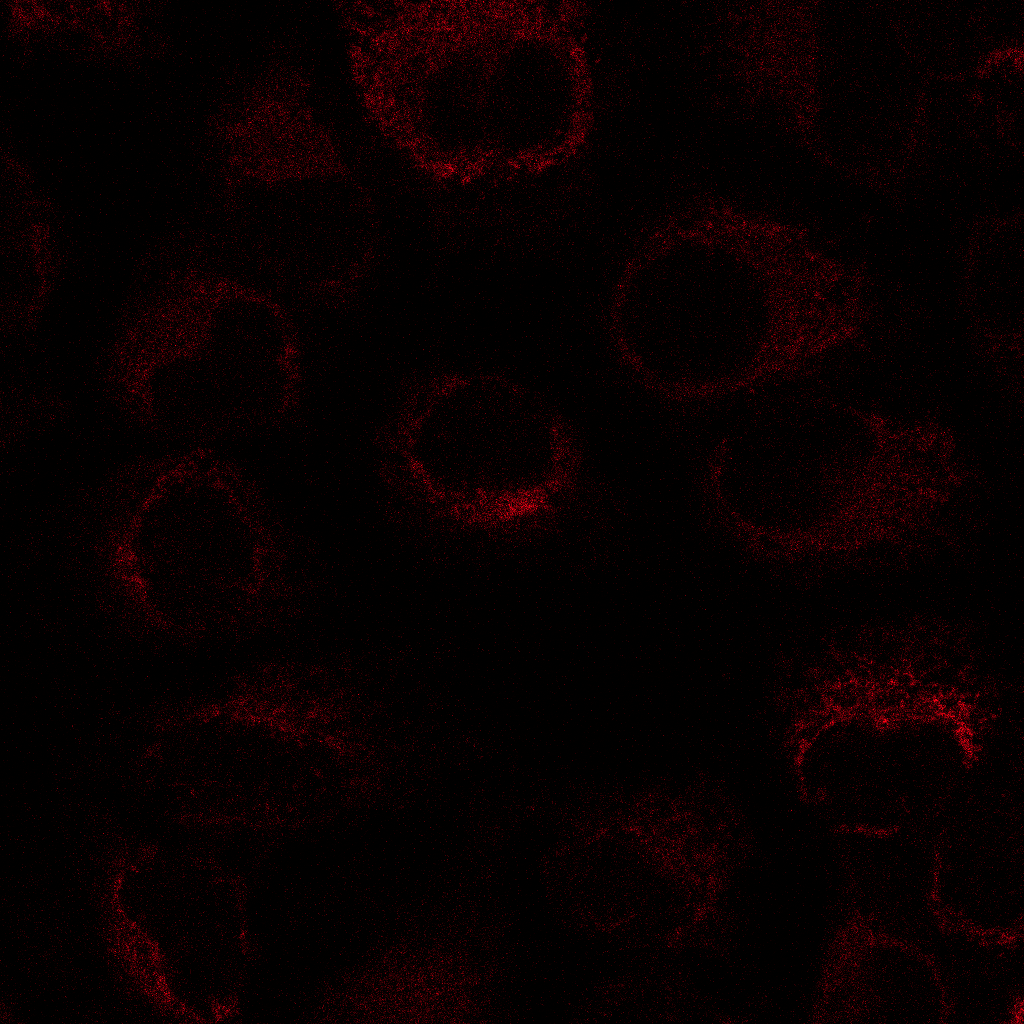

Supplement: Supplementary file 3 — Source data [file 41467_2022_35472_MOESM3_ESM.zip › Fig 4b/2 h/2h_c1.tif]

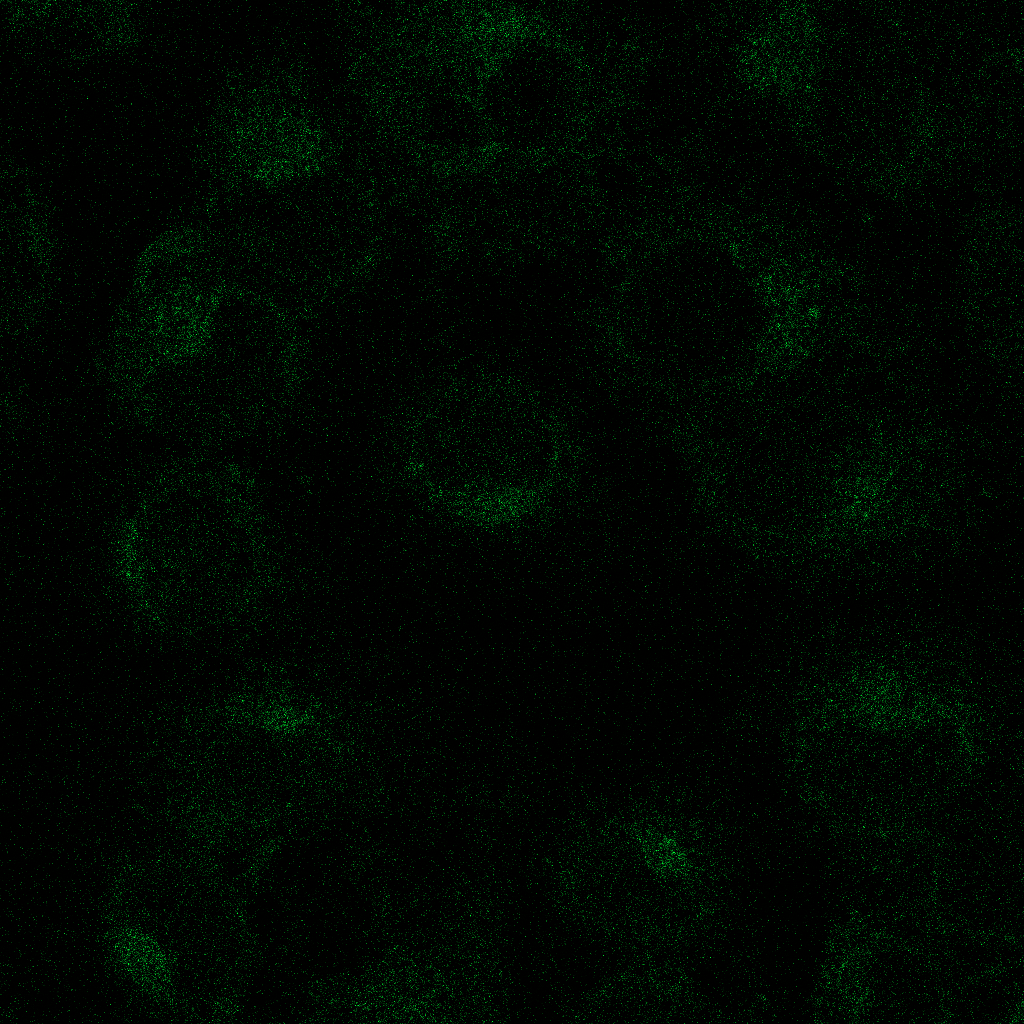

Supplement: Supplementary file 3 — Source data [file 41467_2022_35472_MOESM3_ESM.zip › Fig 4b/2 h/2h_c2.tif]

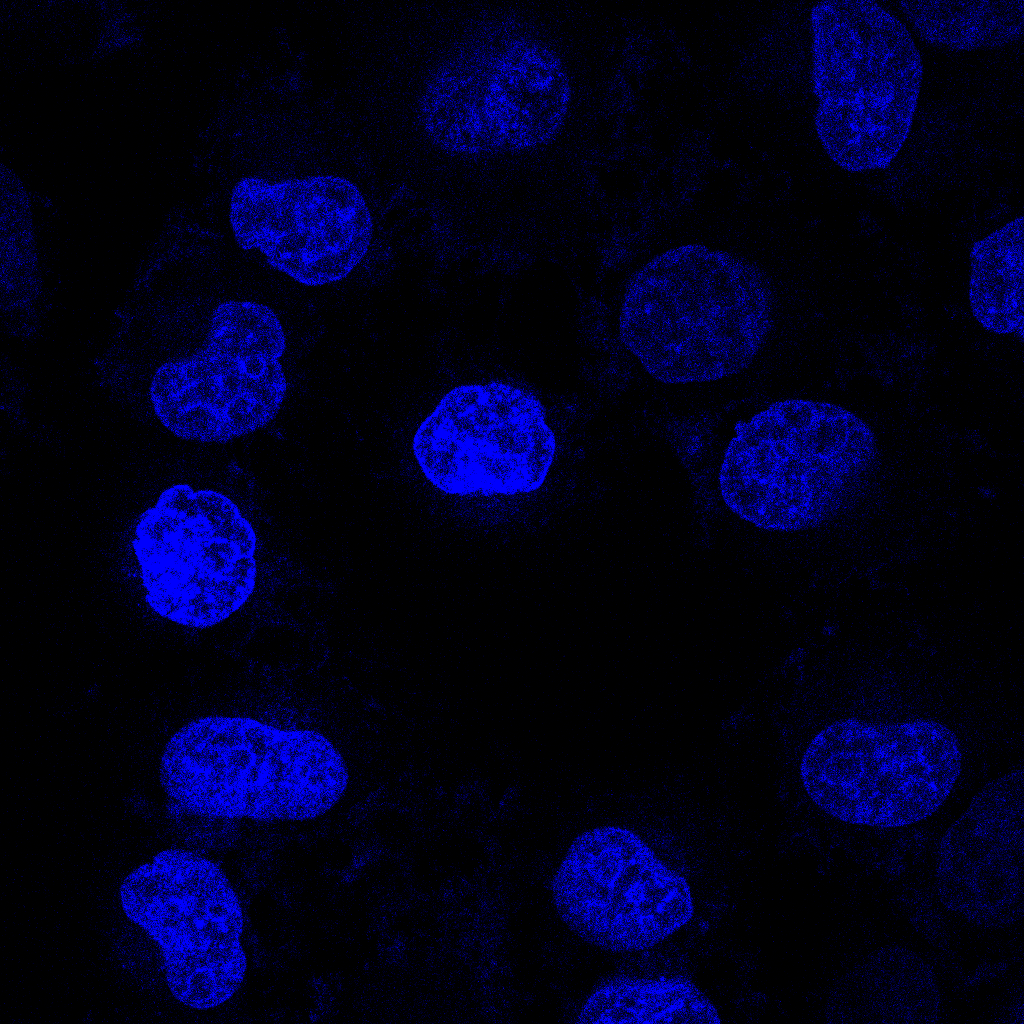

Supplement: Supplementary file 3 — Source data [file 41467_2022_35472_MOESM3_ESM.zip › Fig 4b/2 h/2h_c3.tif]

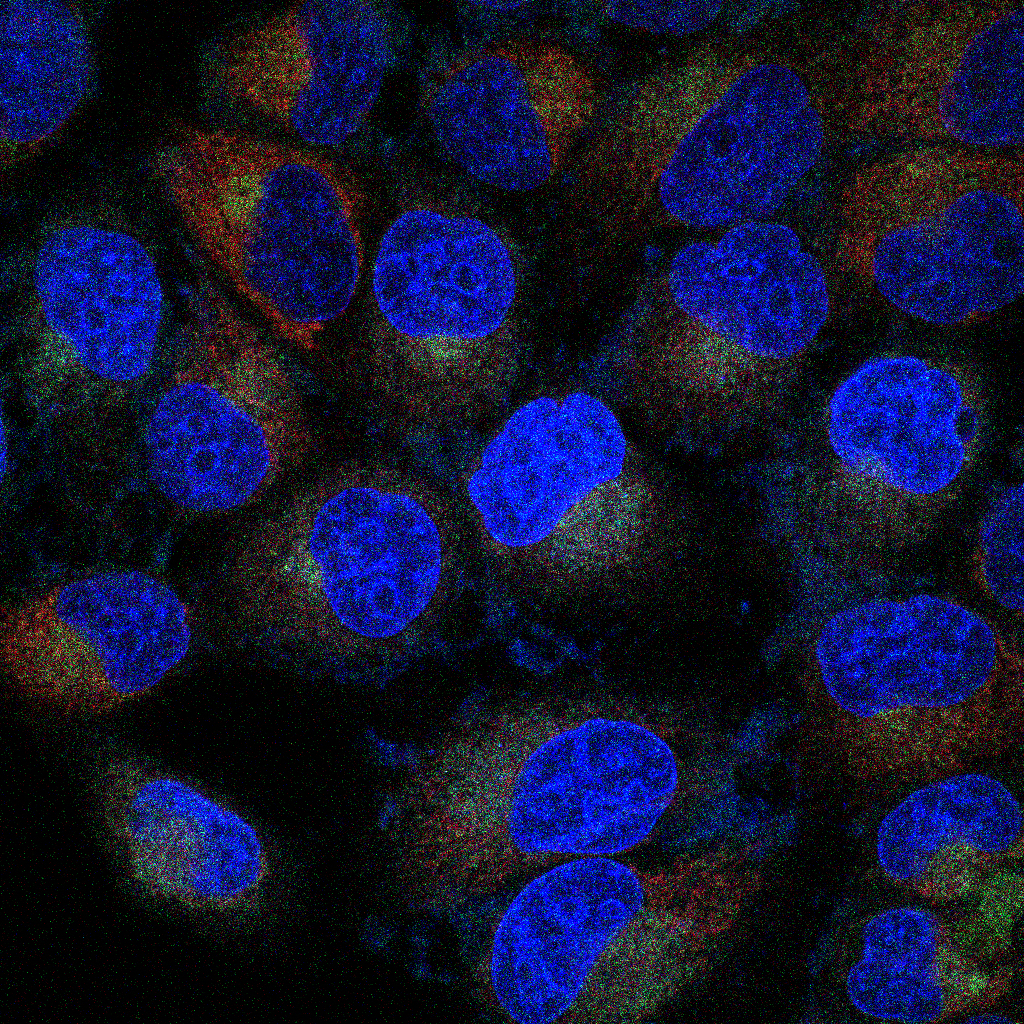

Supplement: Supplementary file 3 — Source data [file 41467_2022_35472_MOESM3_ESM.zip › Fig 4b/4 h/4h_c1-3.tif]

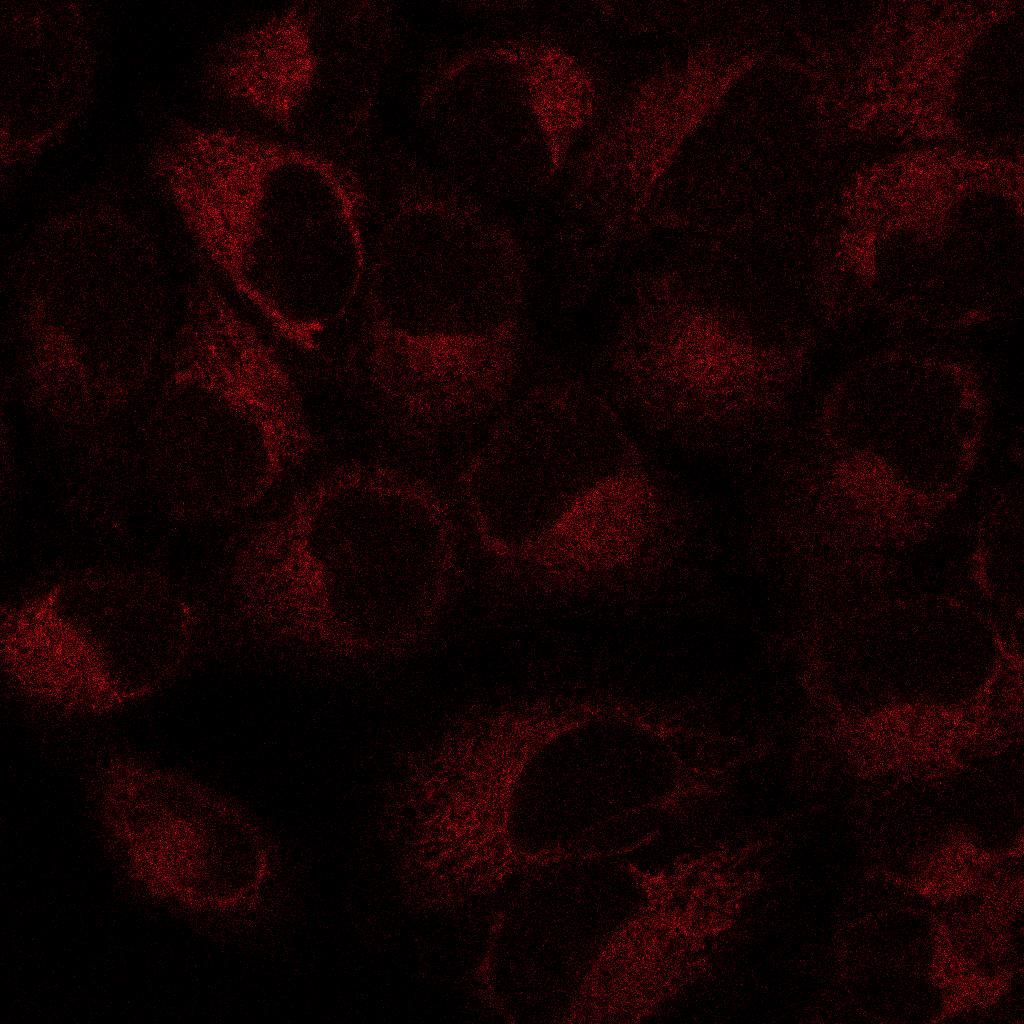

Supplement: Supplementary file 3 — Source data [file 41467_2022_35472_MOESM3_ESM.zip › Fig 4b/4 h/4h_c1.tif]

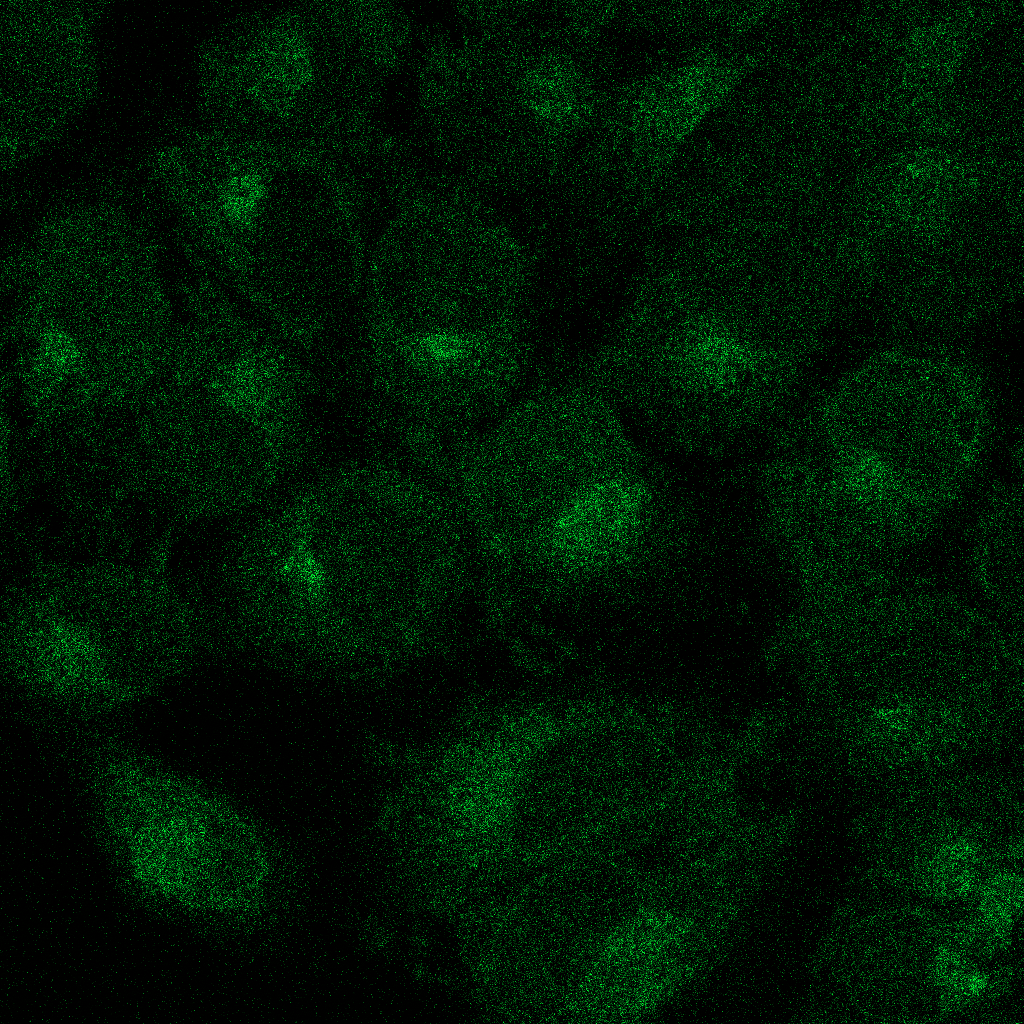

Supplement: Supplementary file 3 — Source data [file 41467_2022_35472_MOESM3_ESM.zip › Fig 4b/4 h/4h_c2.tif]

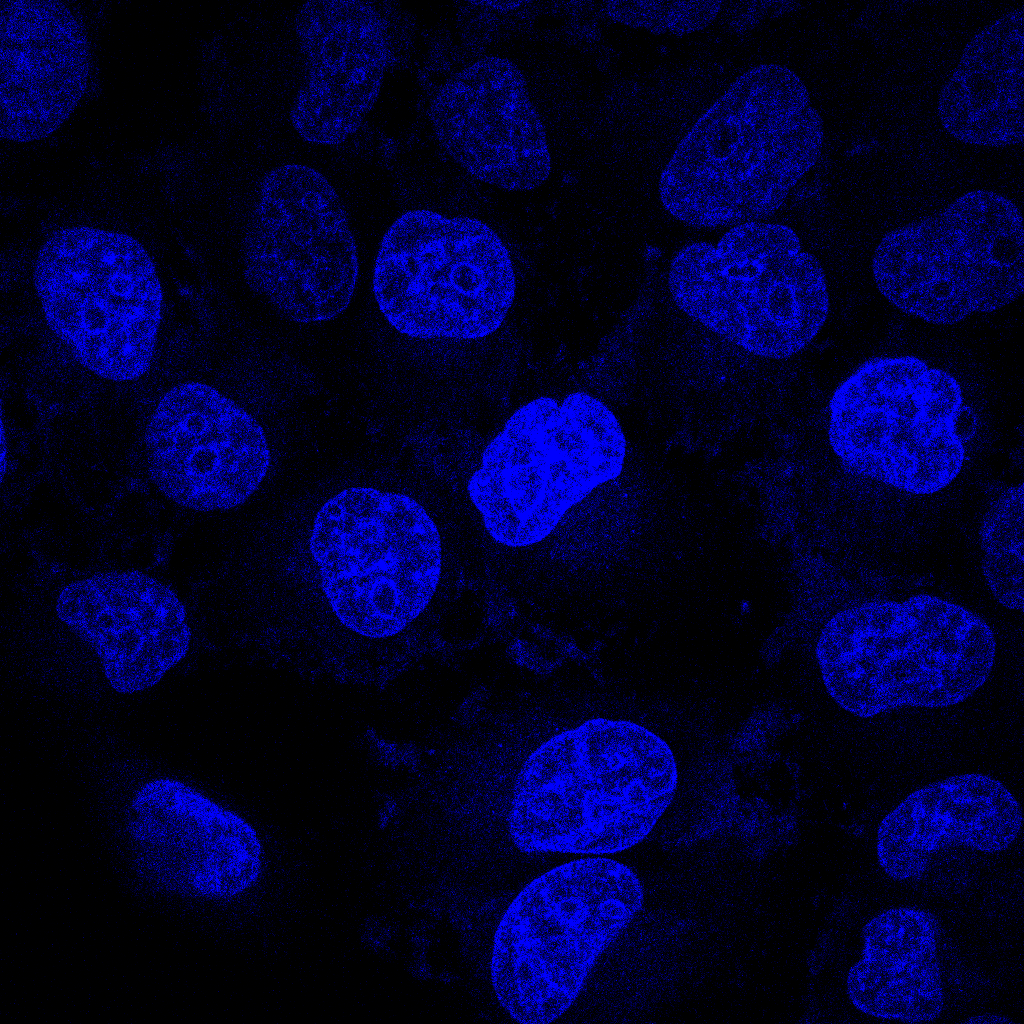

Supplement: Supplementary file 3 — Source data [file 41467_2022_35472_MOESM3_ESM.zip › Fig 4b/4 h/4h_c3.tif]

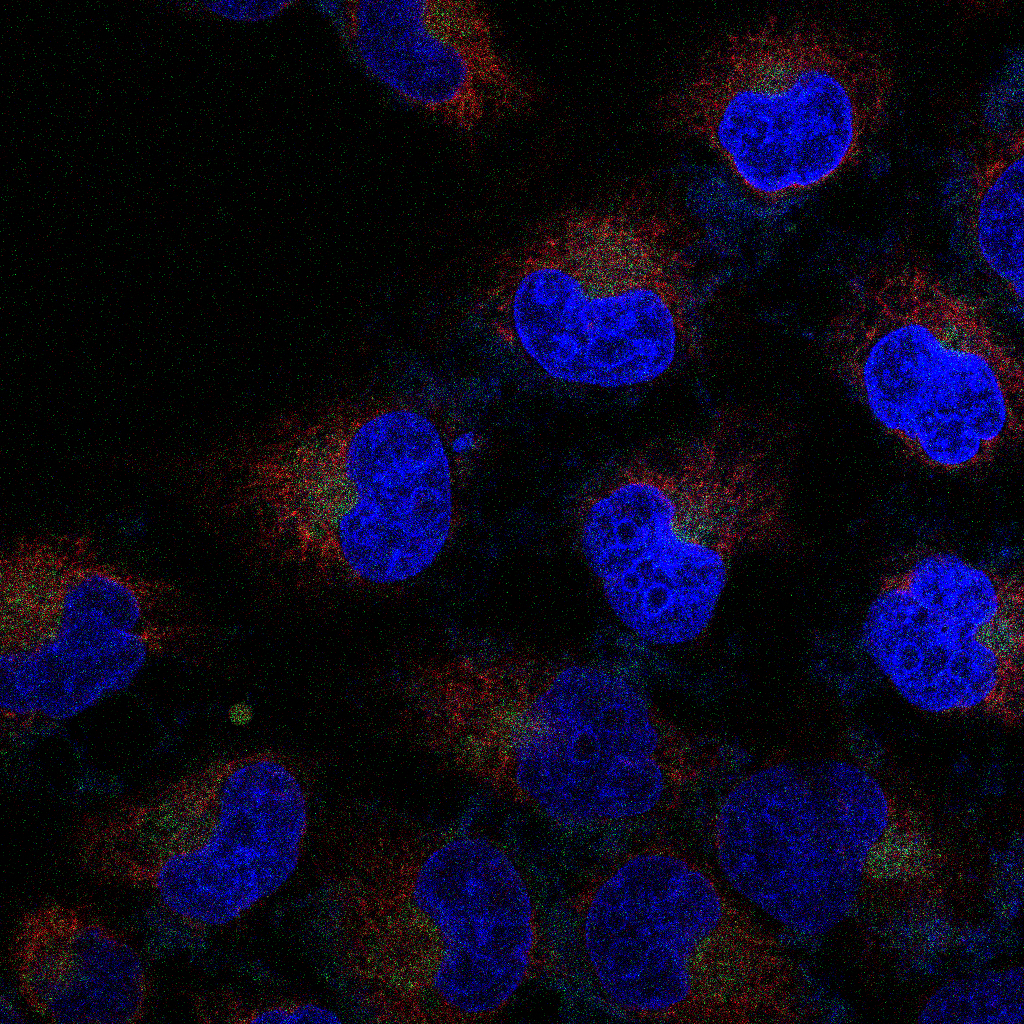

Supplement: Supplementary file 3 — Source data [file 41467_2022_35472_MOESM3_ESM.zip › Fig 4b/6 h/6h_c1-3.tif]

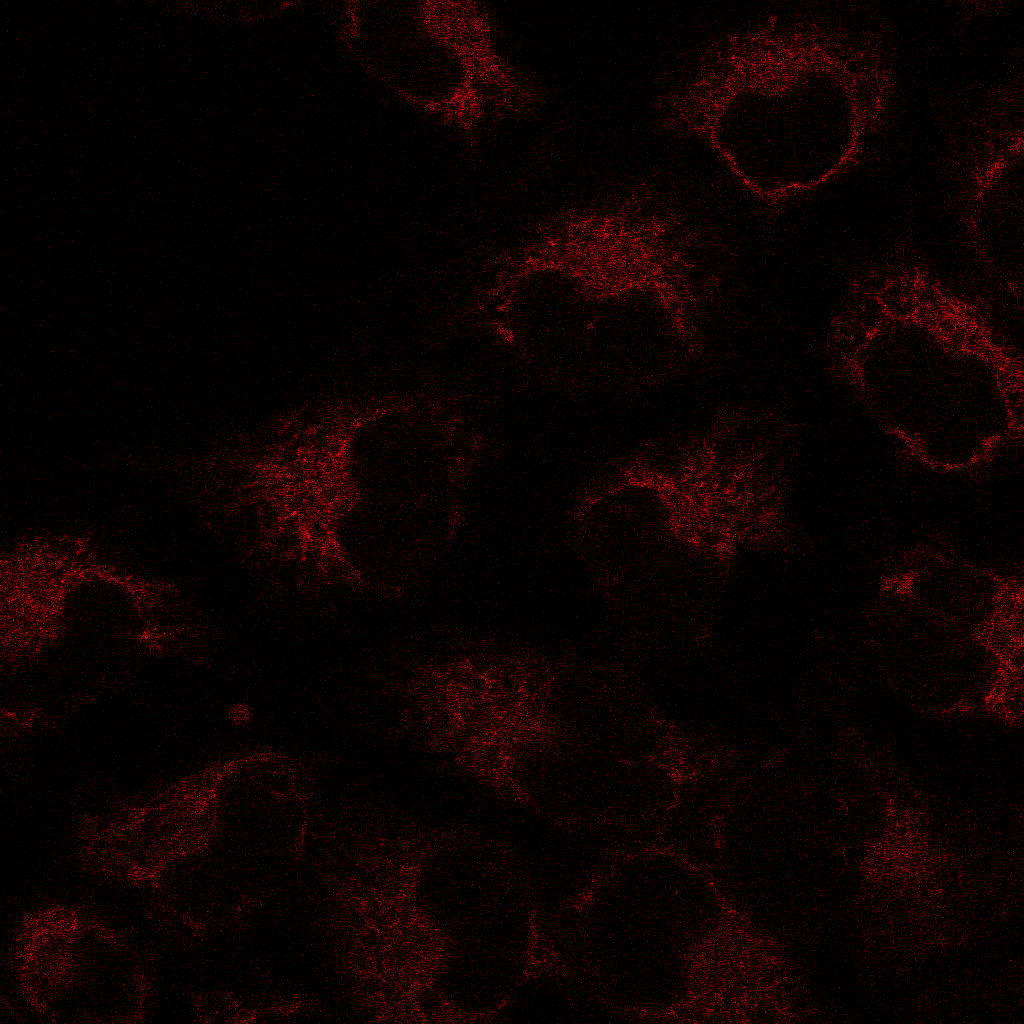

Supplement: Supplementary file 3 — Source data [file 41467_2022_35472_MOESM3_ESM.zip › Fig 4b/6 h/6h_c1.tif]

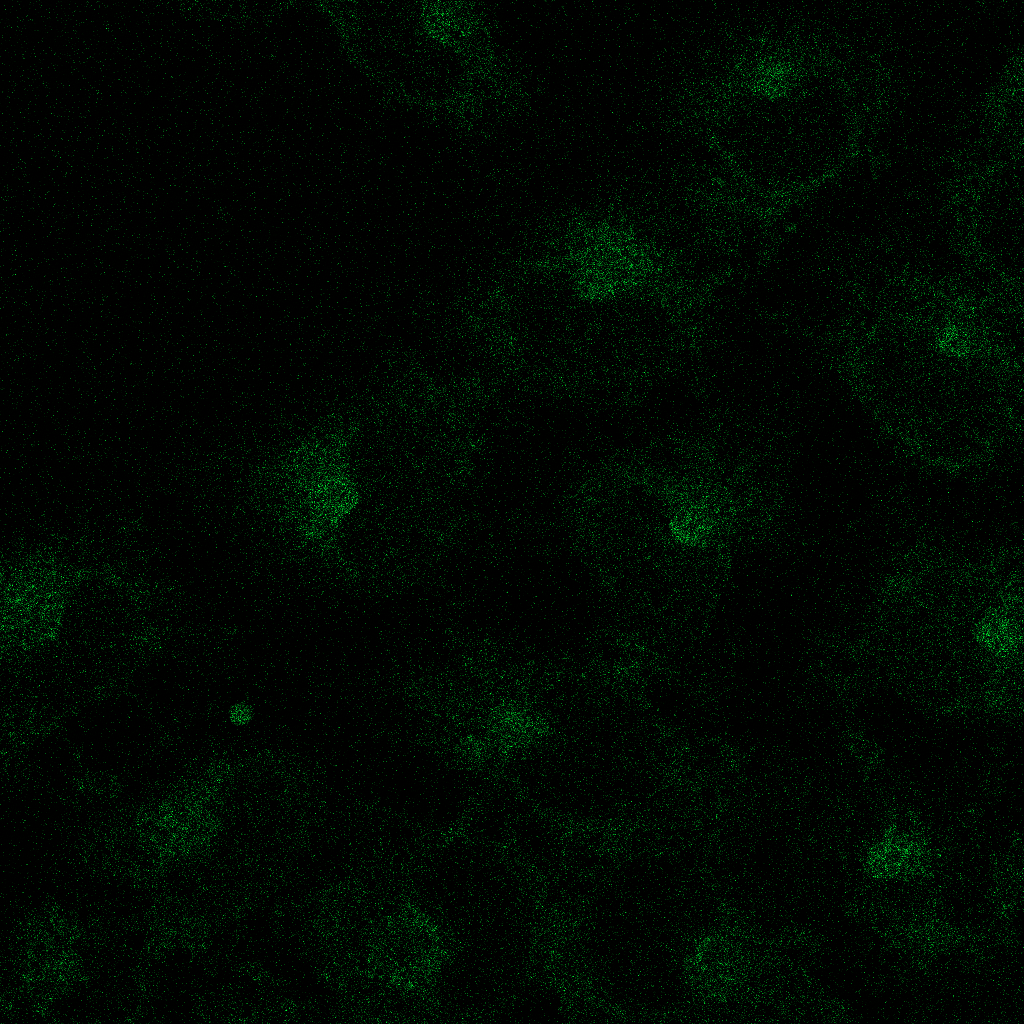

Supplement: Supplementary file 3 — Source data [file 41467_2022_35472_MOESM3_ESM.zip › Fig 4b/6 h/6h_c2.tif]

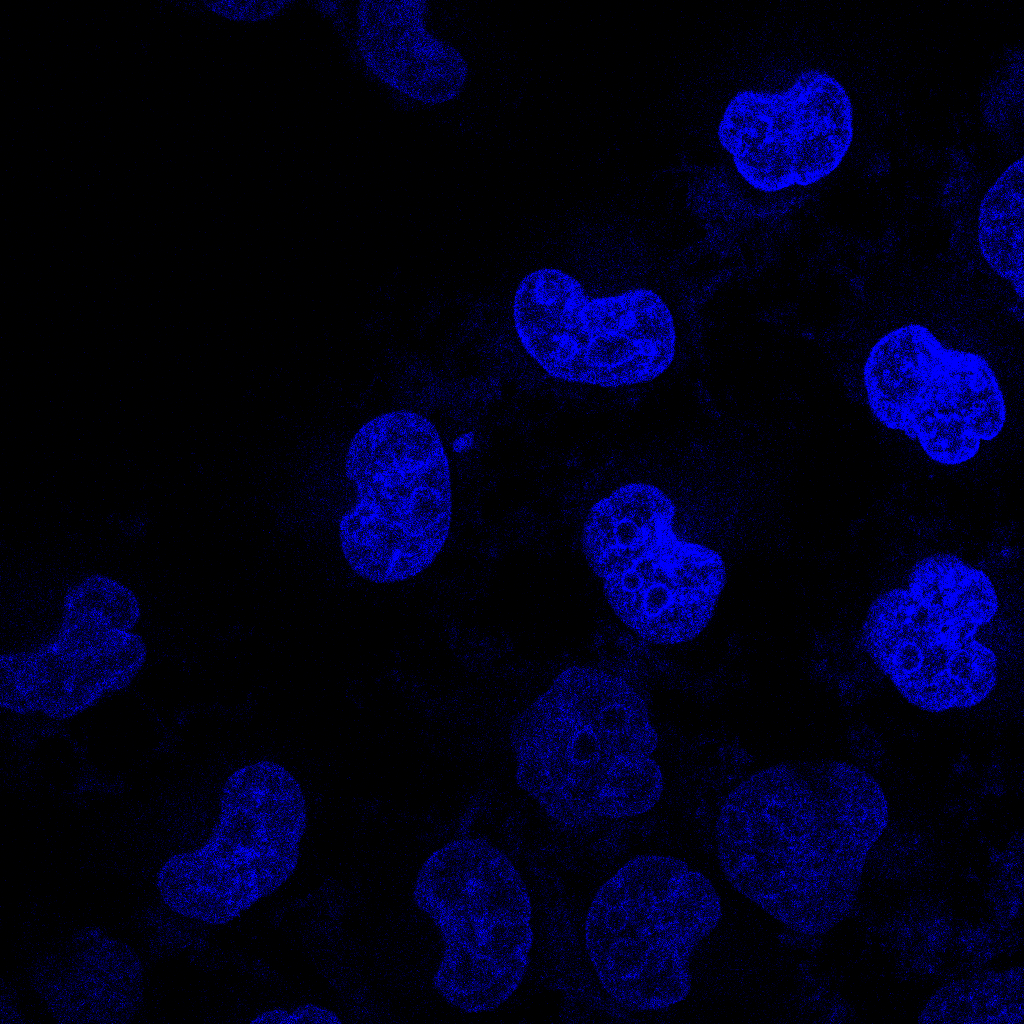

Supplement: Supplementary file 3 — Source data [file 41467_2022_35472_MOESM3_ESM.zip › Fig 4b/6 h/6h_c3.tif]

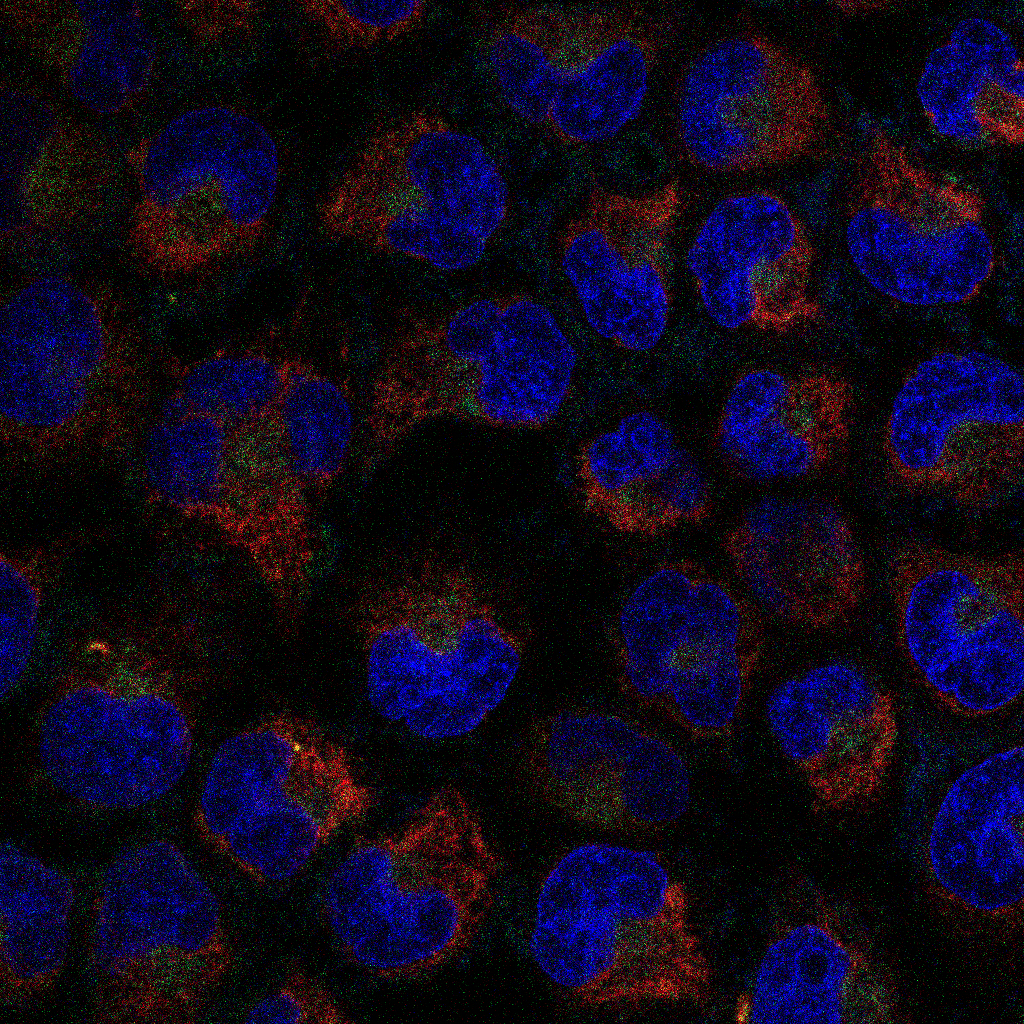

Supplement: Supplementary file 3 — Source data [file 41467_2022_35472_MOESM3_ESM.zip › Fig 4b/8 h/8h_c1-3.tif]

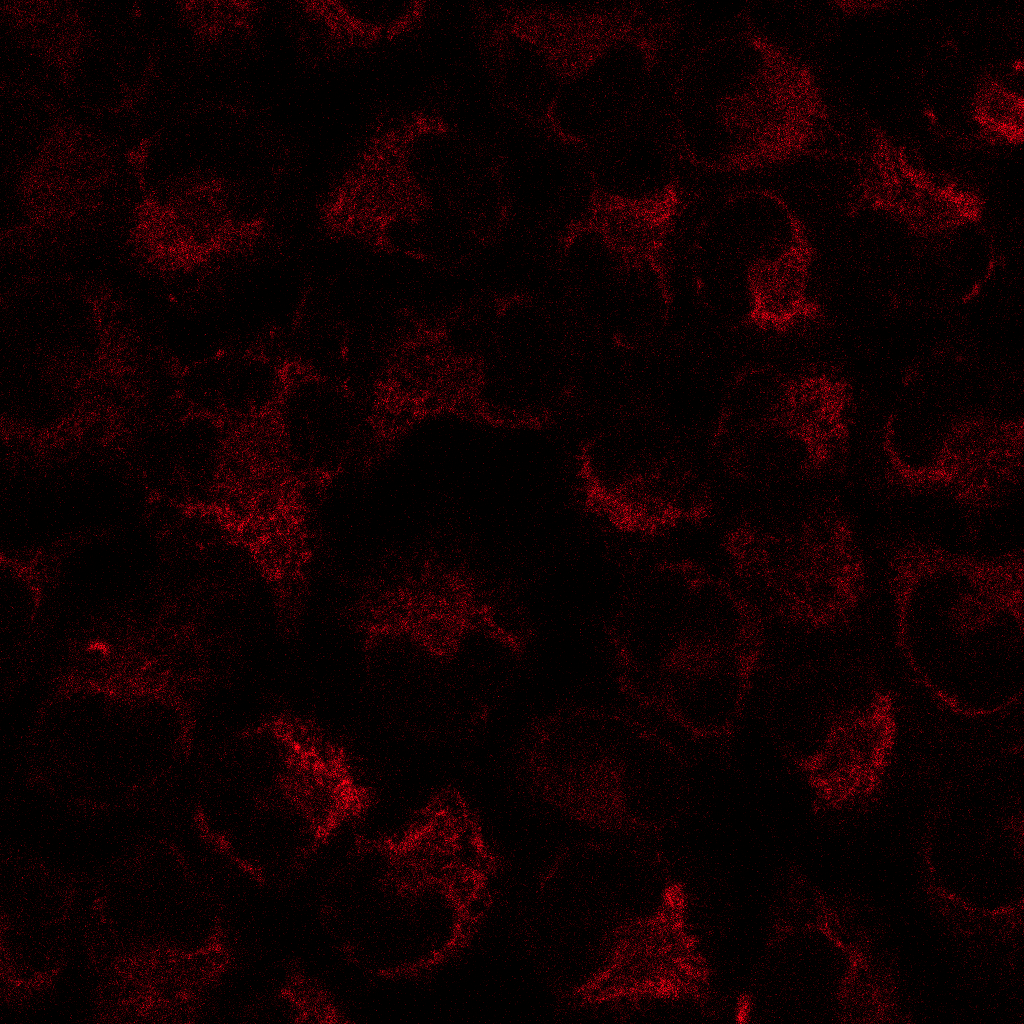

Supplement: Supplementary file 3 — Source data [file 41467_2022_35472_MOESM3_ESM.zip › Fig 4b/8 h/8h_c1.tif]

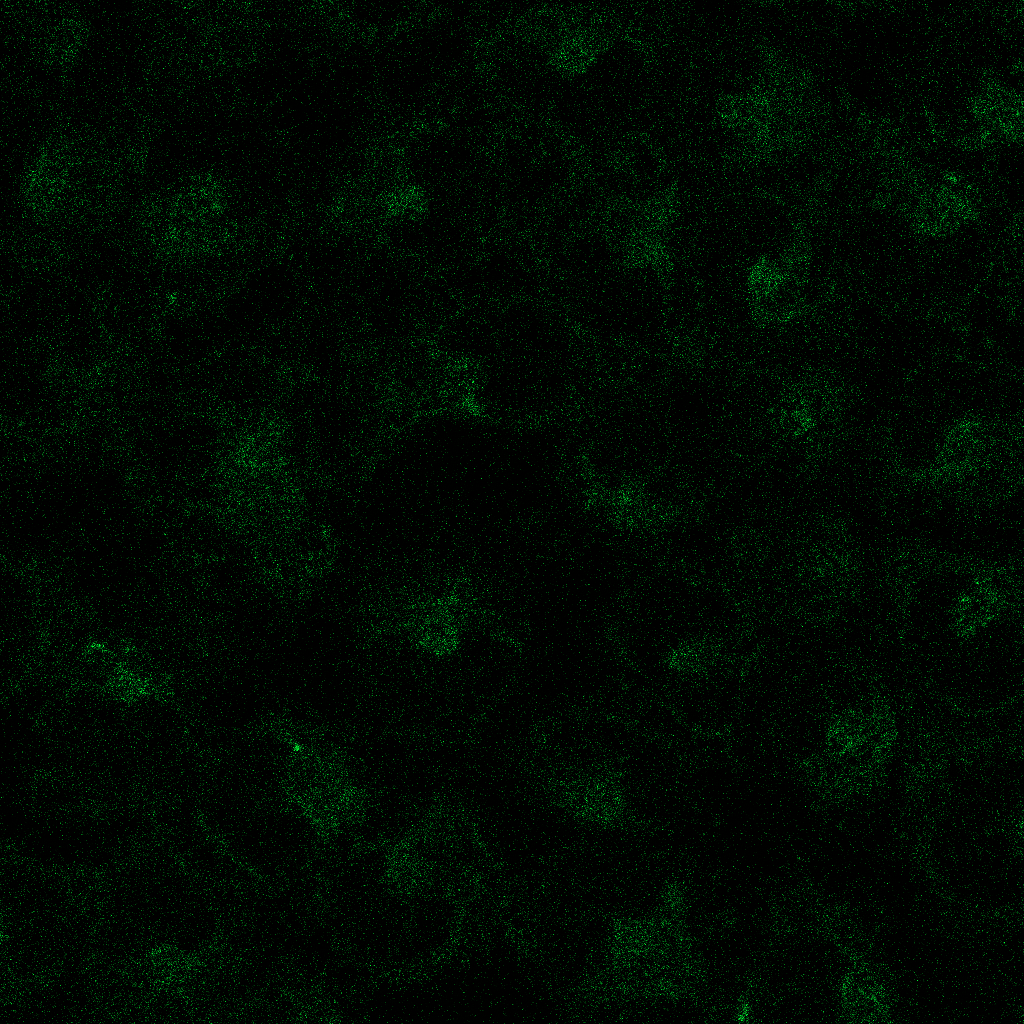

Supplement: Supplementary file 3 — Source data [file 41467_2022_35472_MOESM3_ESM.zip › Fig 4b/8 h/8h_c2.tif]

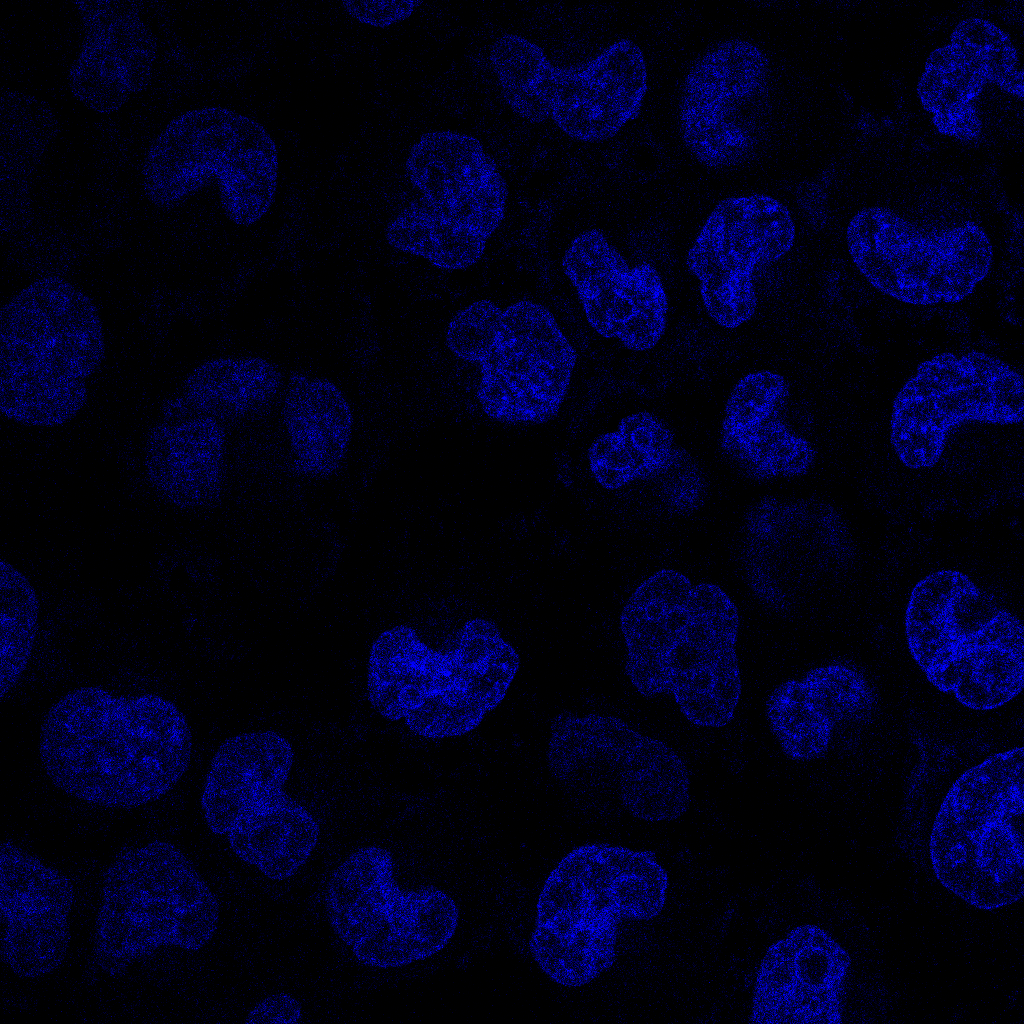

Supplement: Supplementary file 3 — Source data [file 41467_2022_35472_MOESM3_ESM.zip › Fig 4b/8 h/8h_c3.tif]

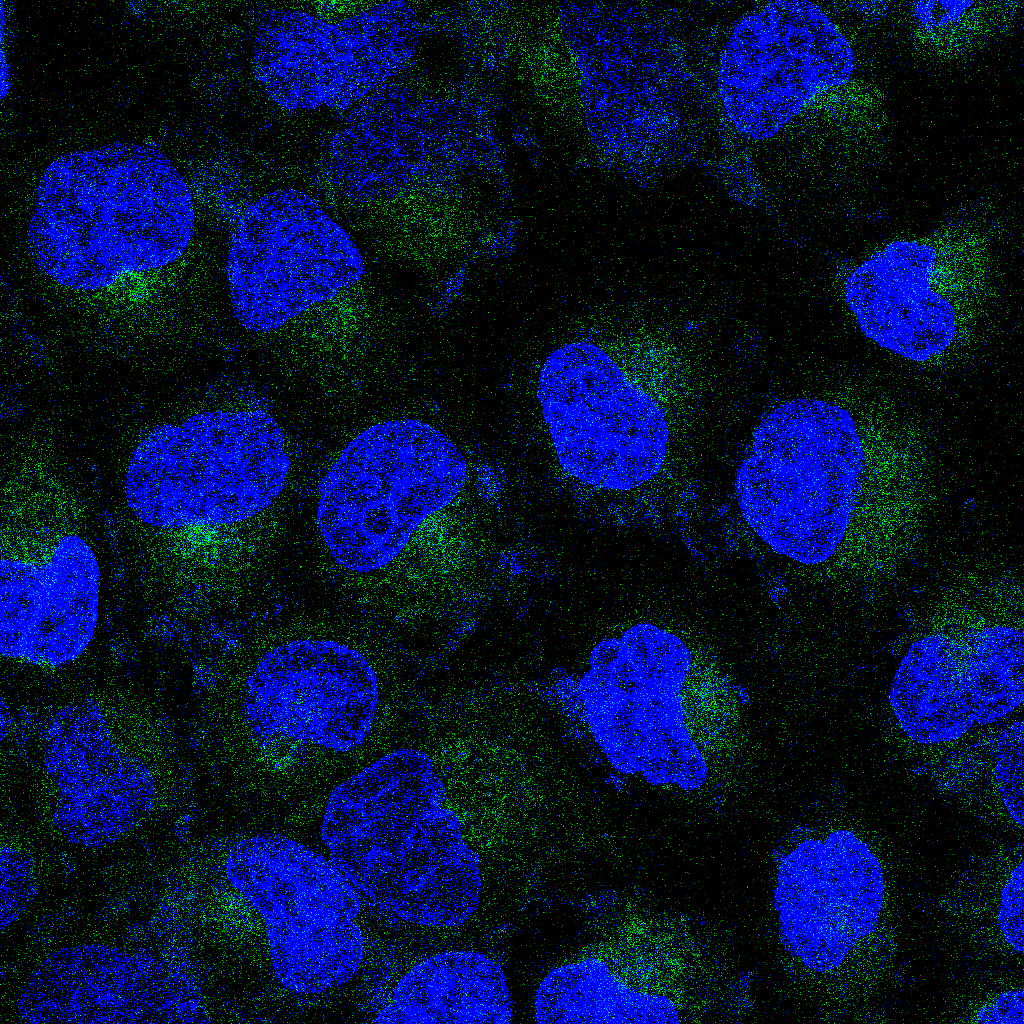

Supplement: Supplementary file 3 — Source data [file 41467_2022_35472_MOESM3_ESM.zip › Fig 4b/PBS/PBS_c1-3.tif]

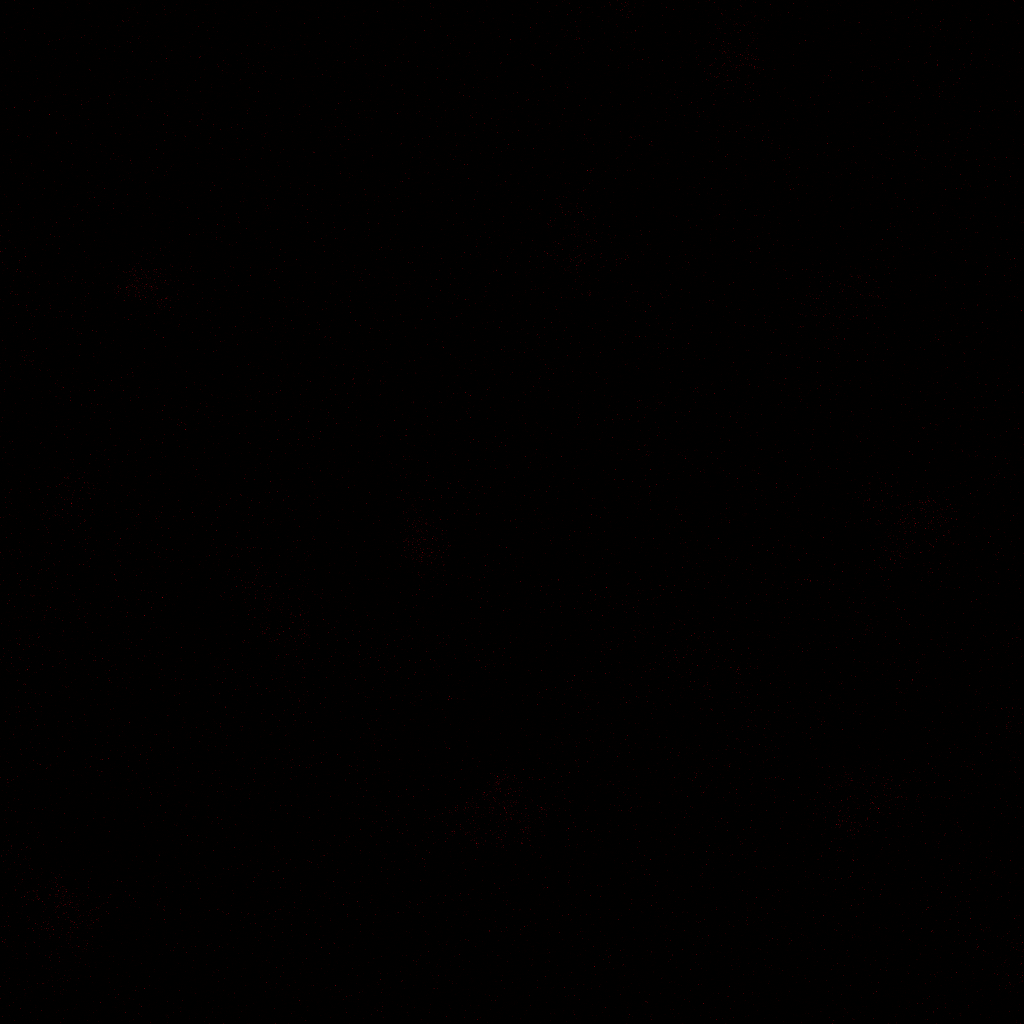

Supplement: Supplementary file 3 — Source data [file 41467_2022_35472_MOESM3_ESM.zip › Fig 4b/PBS/PBS_c1.tif]

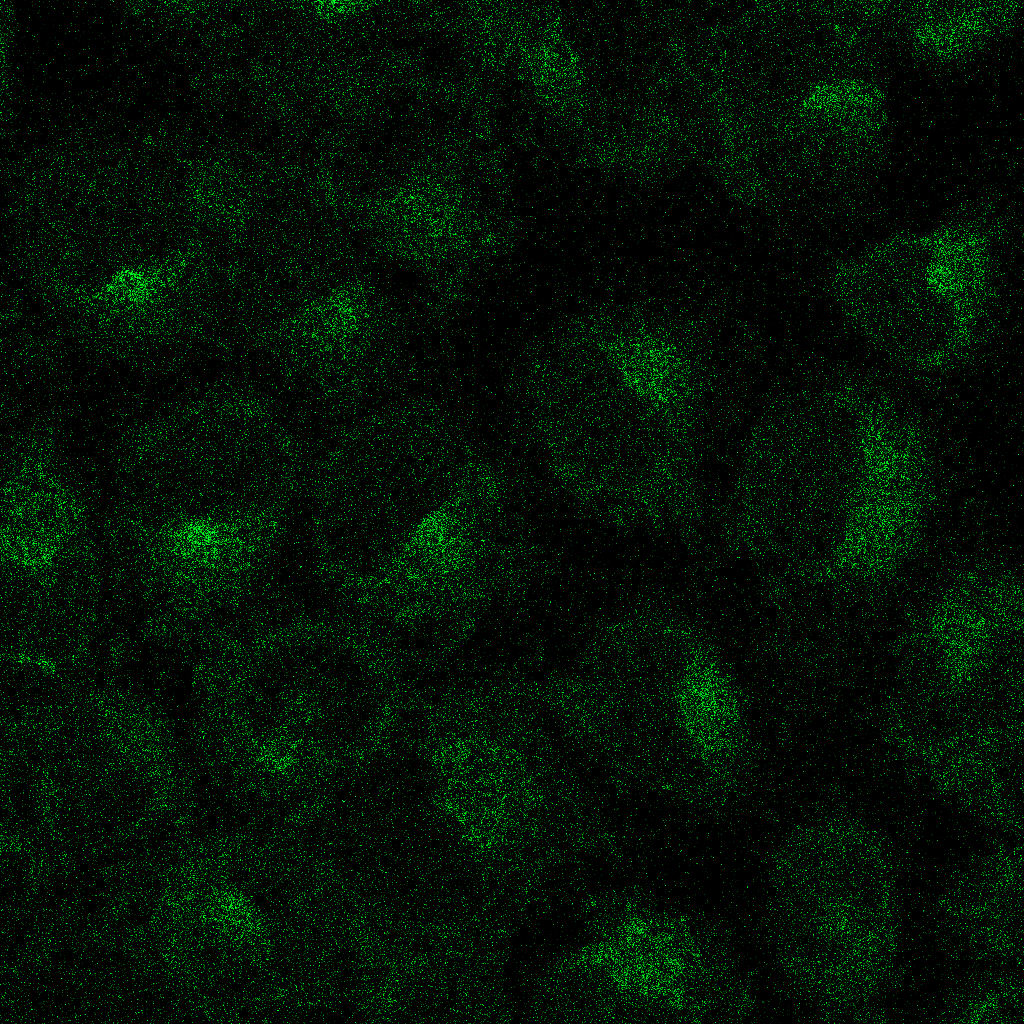

Supplement: Supplementary file 3 — Source data [file 41467_2022_35472_MOESM3_ESM.zip › Fig 4b/PBS/PBS_c2.tif]

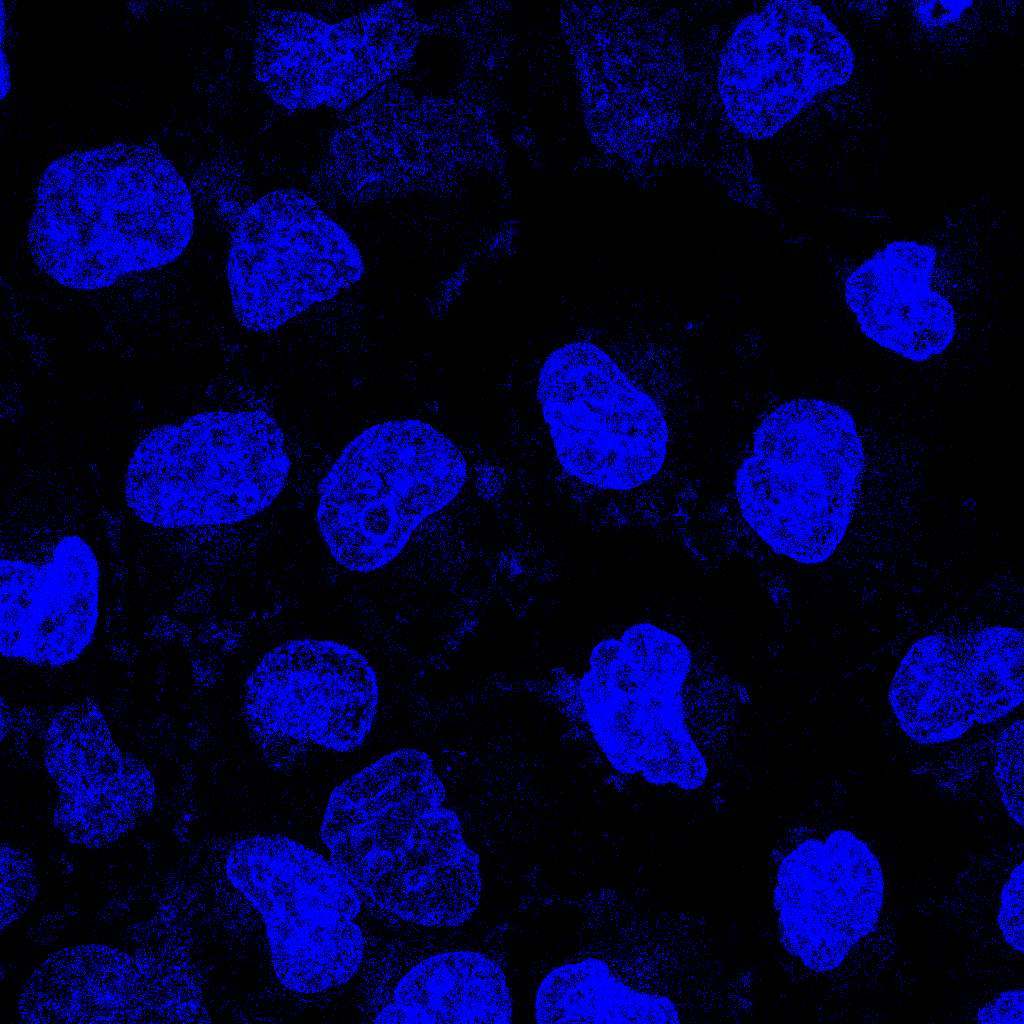

Supplement: Supplementary file 3 — Source data [file 41467_2022_35472_MOESM3_ESM.zip › Fig 4b/PBS/PBS_c3.tif]

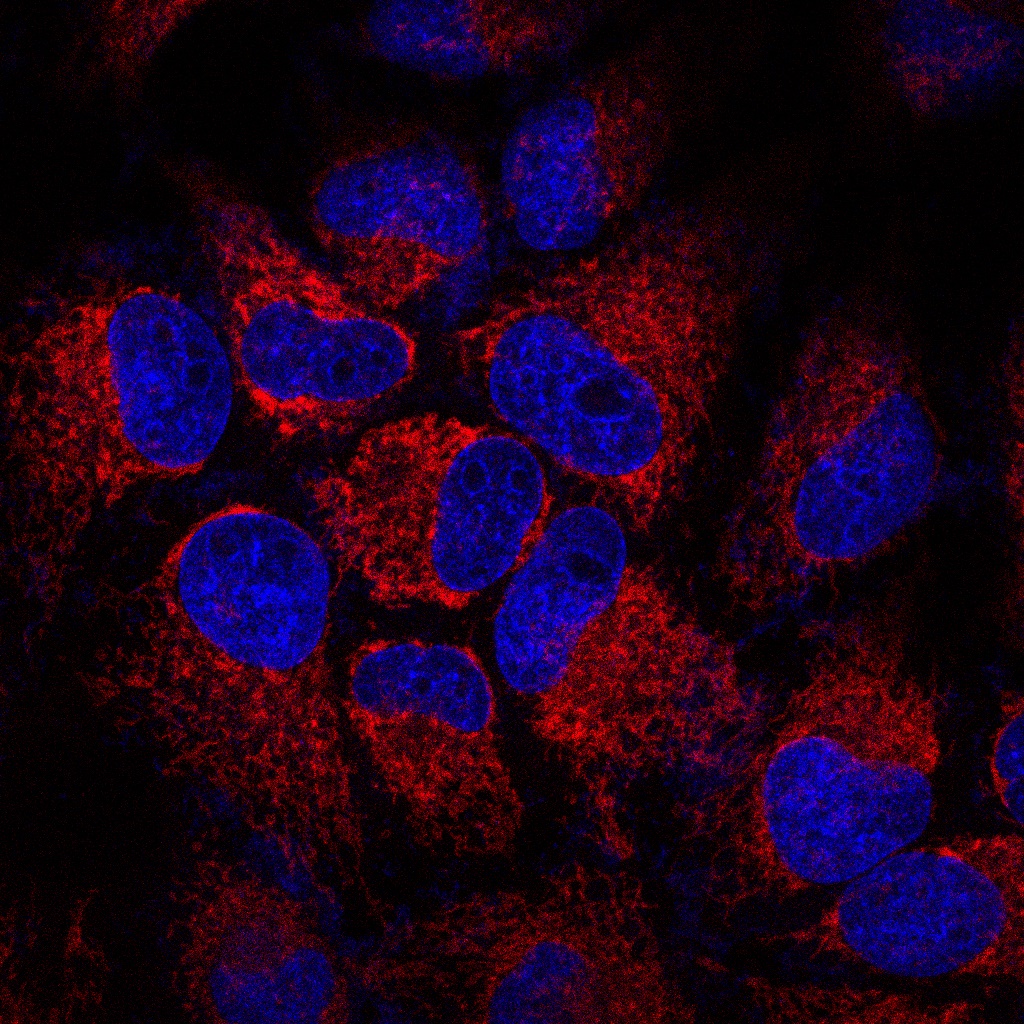

Supplement: Supplementary file 3 — Source data [file 41467_2022_35472_MOESM3_ESM.zip › Fig 4cd/DCNC.jpg]

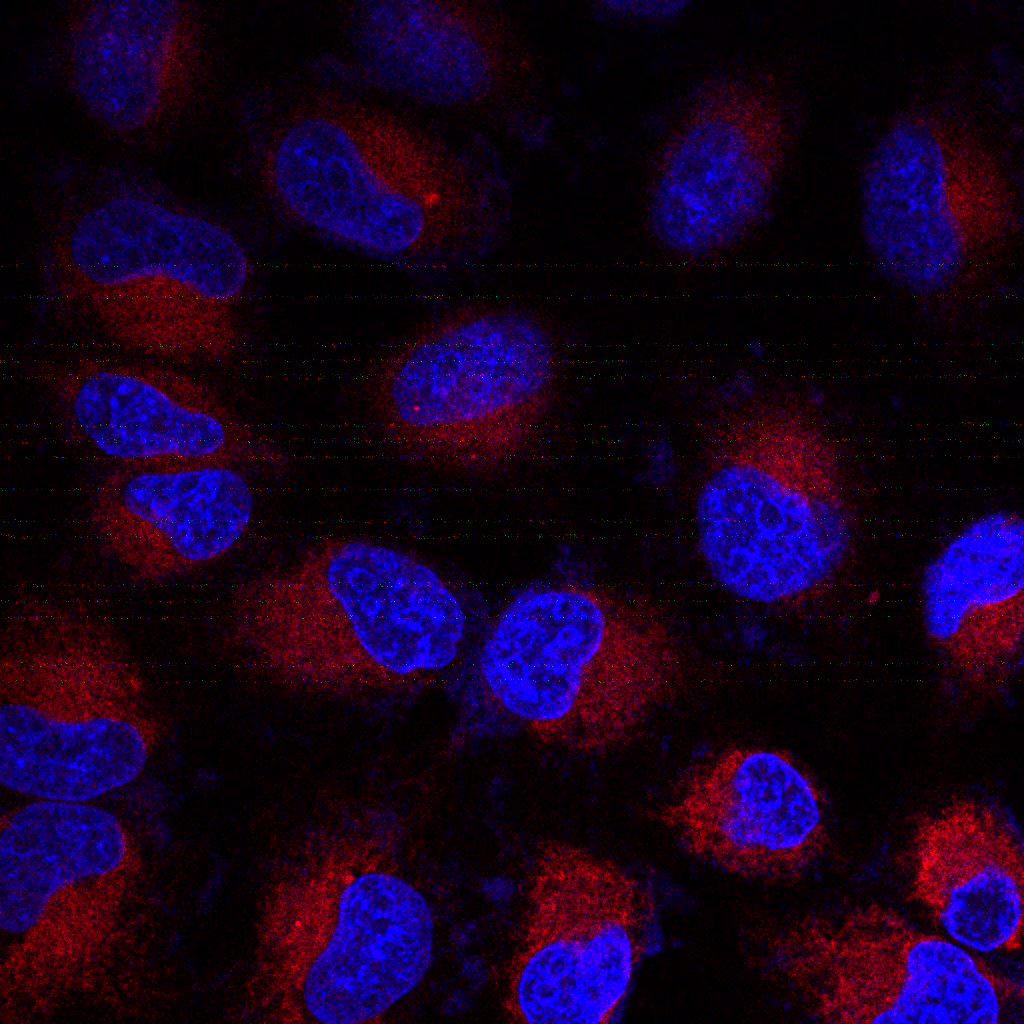

Supplement: Supplementary file 3 — Source data [file 41467_2022_35472_MOESM3_ESM.zip › Fig 4cd/DniCNC.tif]

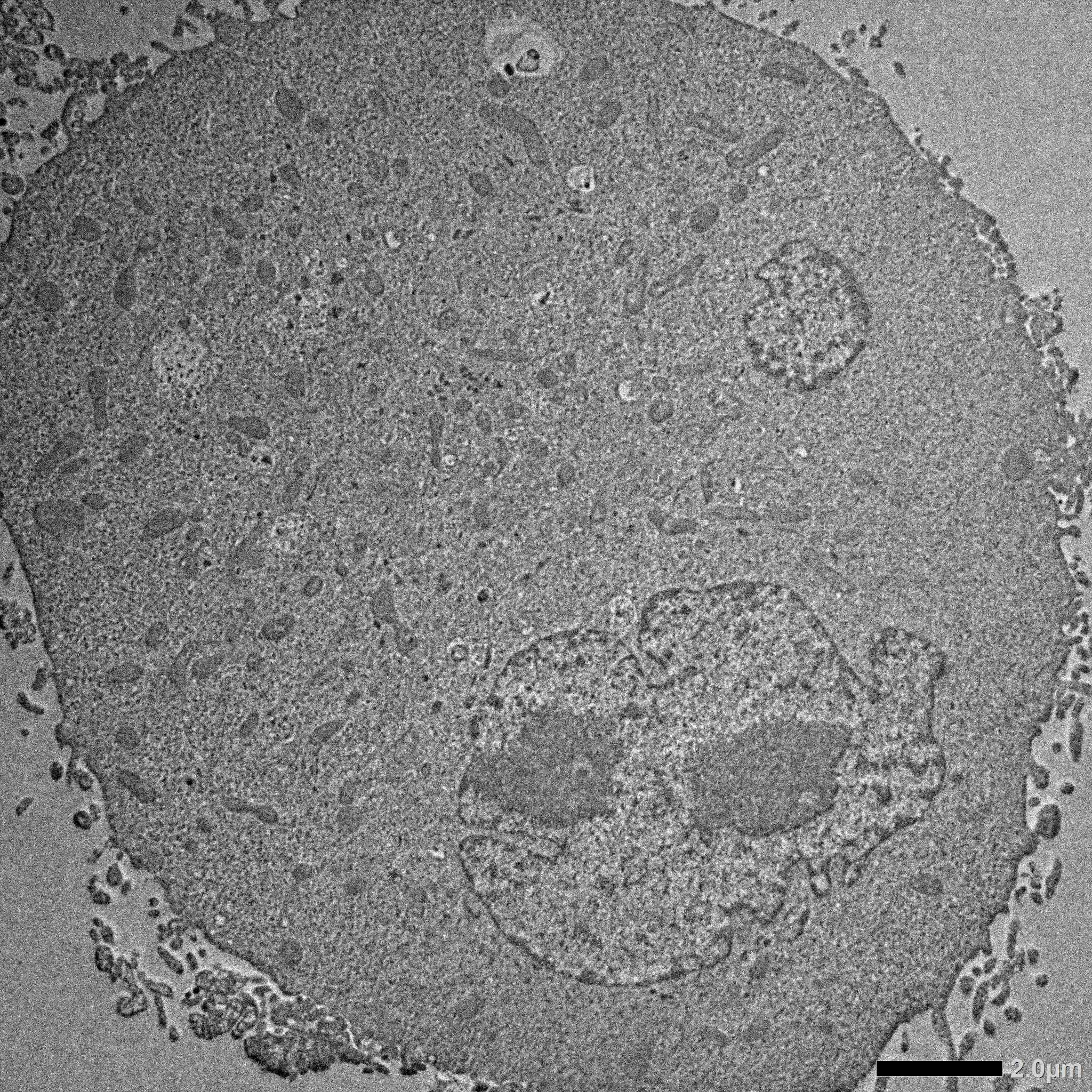

Supplement: Supplementary file 3 — Source data [file 41467_2022_35472_MOESM3_ESM.zip › Fig 4ef/JEM-1400 Flash_2_MAG_X2000_065.jpg]

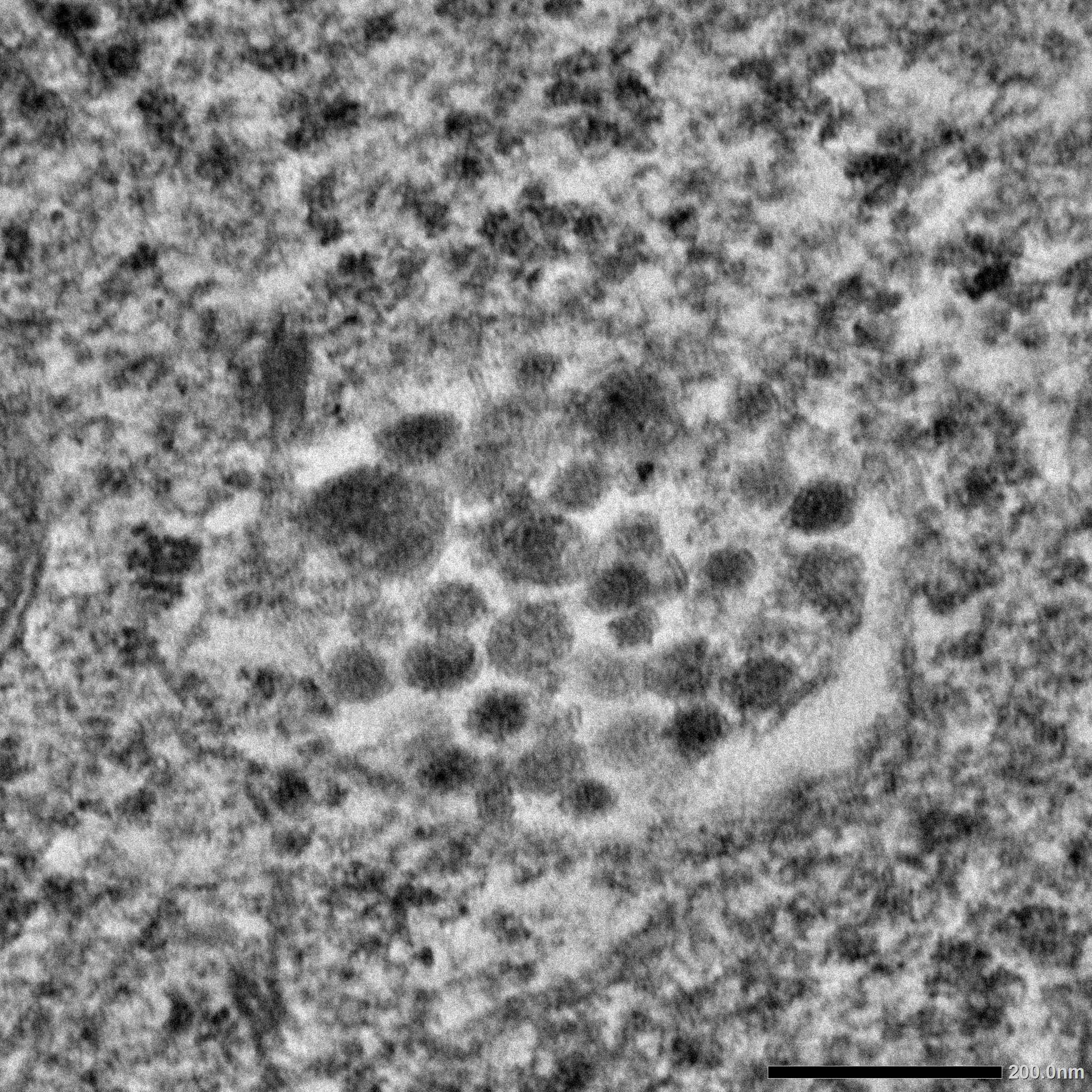

Supplement: Supplementary file 3 — Source data [file 41467_2022_35472_MOESM3_ESM.zip › Fig 4ef/JEM-1400 Flash_2_SA-MAG_X40k_075.jpg]

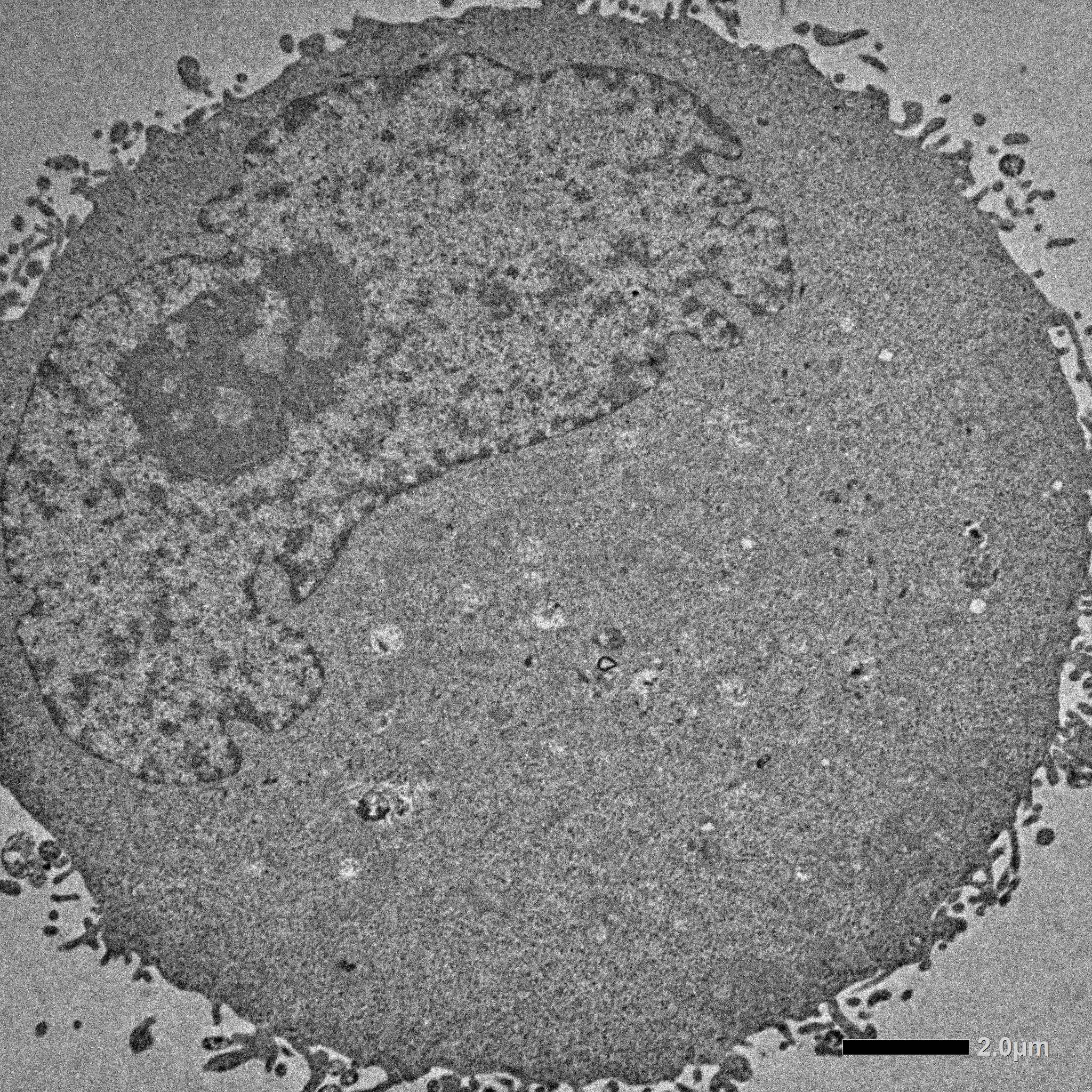

Supplement: Supplementary file 3 — Source data [file 41467_2022_35472_MOESM3_ESM.zip › Fig 4ef/JEM-1400 Flash_3_MAG_X2000_001.jpg]

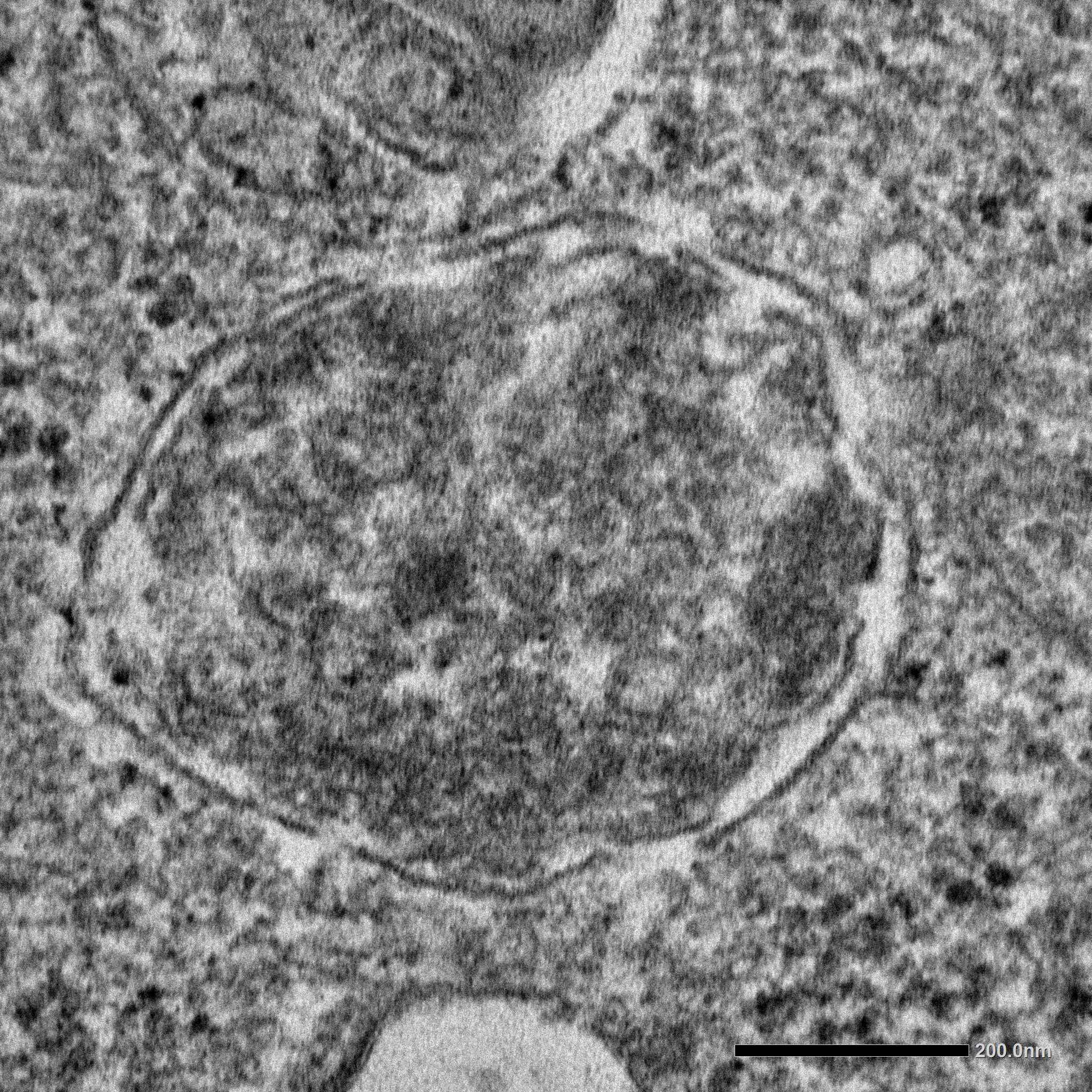

Supplement: Supplementary file 3 — Source data [file 41467_2022_35472_MOESM3_ESM.zip › Fig 4ef/JEM-1400 Flash_3_SA-MAG_X40k_022.jpg]

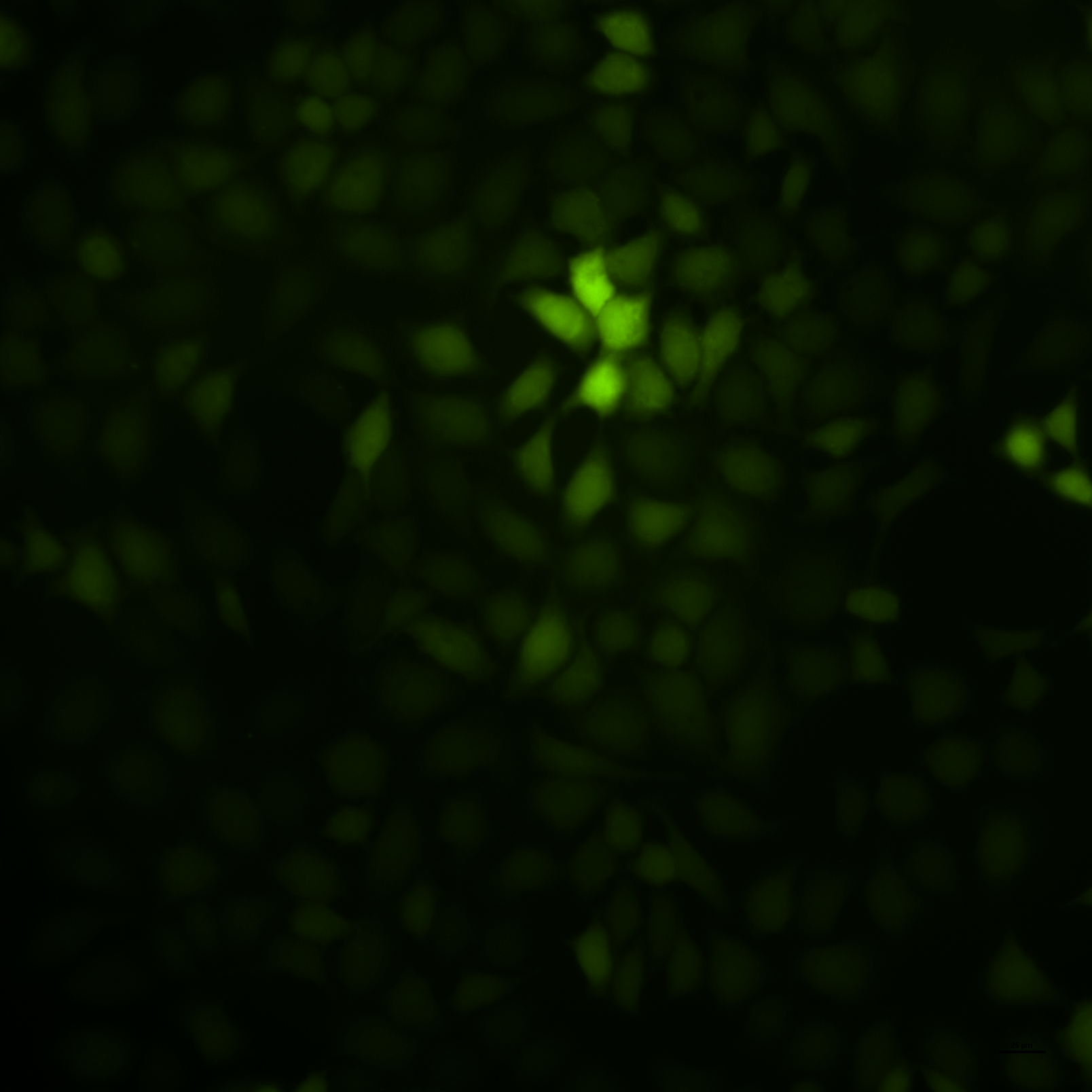

Supplement: Supplementary file 3 — Source data [file 41467_2022_35472_MOESM3_ESM.zip › Fig 5cd/1. Blank.tif]

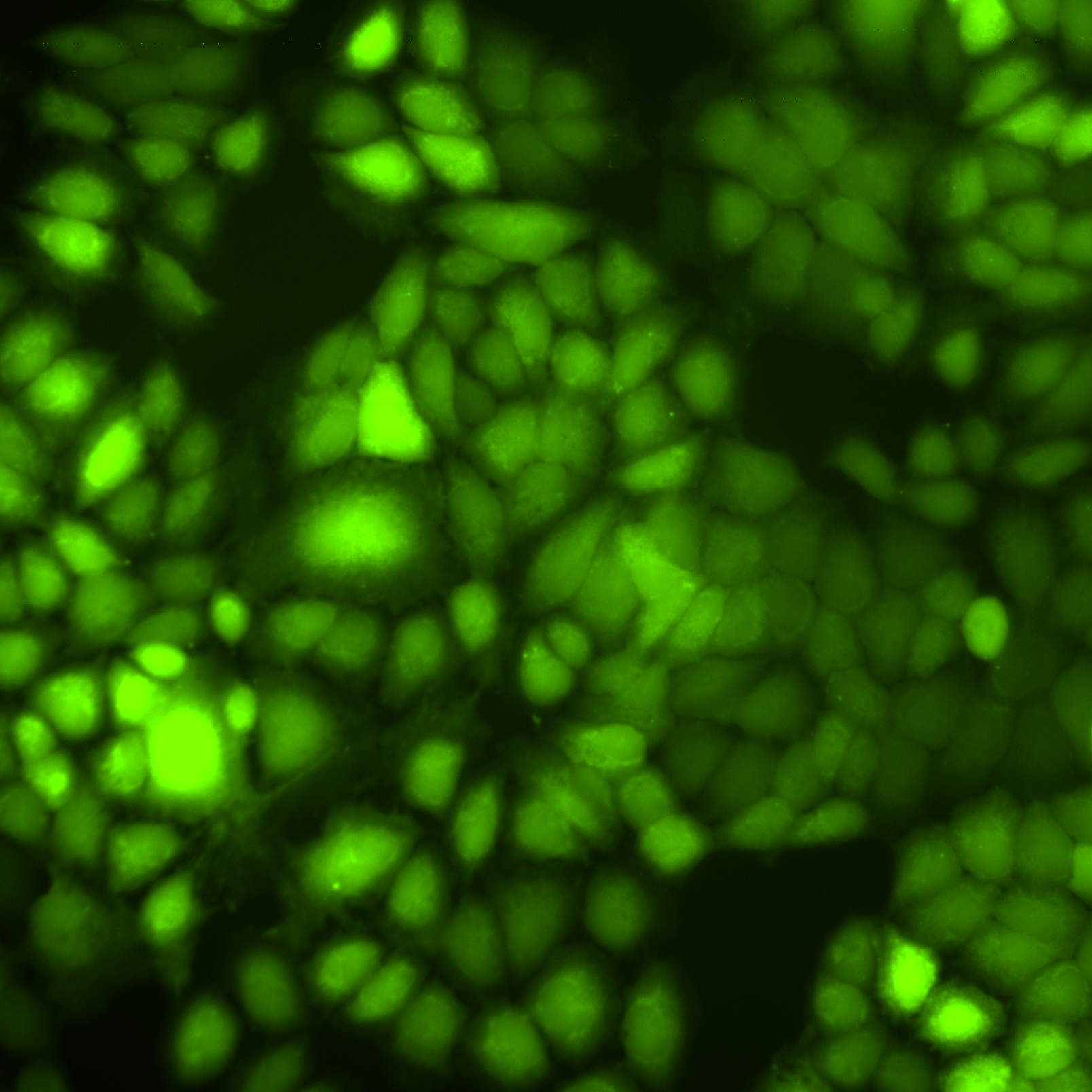

Supplement: Supplementary file 3 — Source data [file 41467_2022_35472_MOESM3_ESM.zip › Fig 5cd/2. PBS.tif]

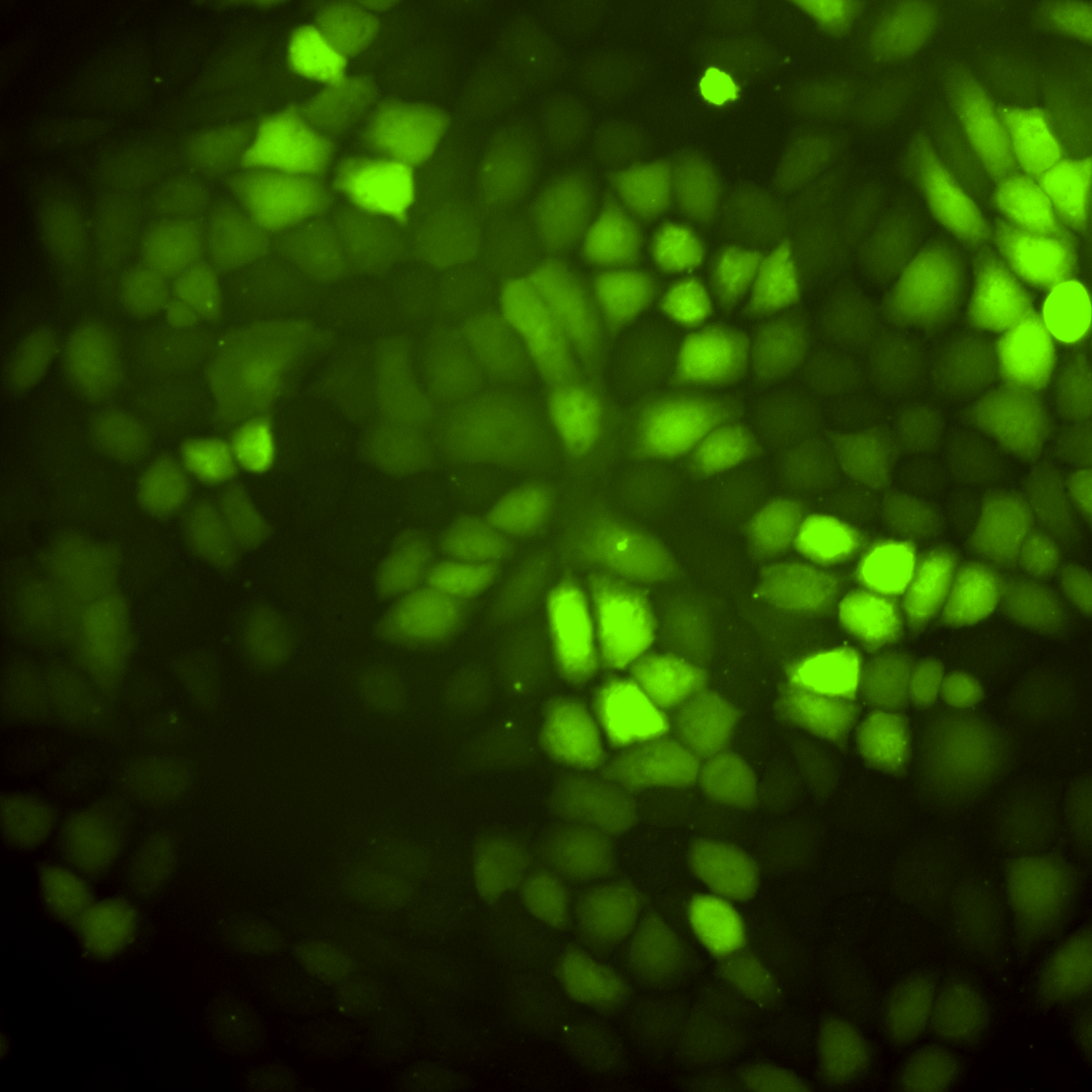

Supplement: Supplementary file 3 — Source data [file 41467_2022_35472_MOESM3_ESM.zip › Fig 5cd/3. DniAS.tif]

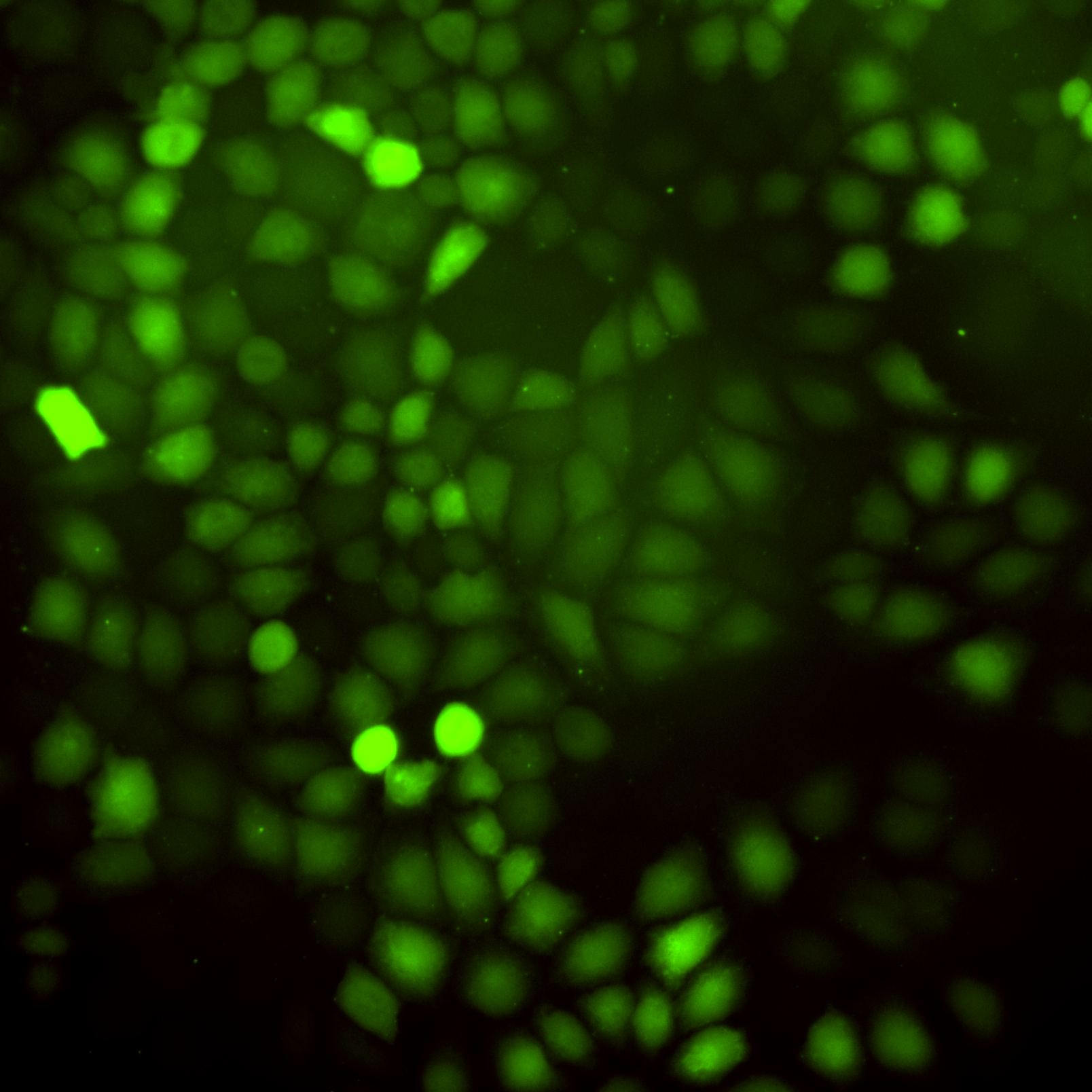

Supplement: Supplementary file 3 — Source data [file 41467_2022_35472_MOESM3_ESM.zip › Fig 5cd/4. DAS.tif]

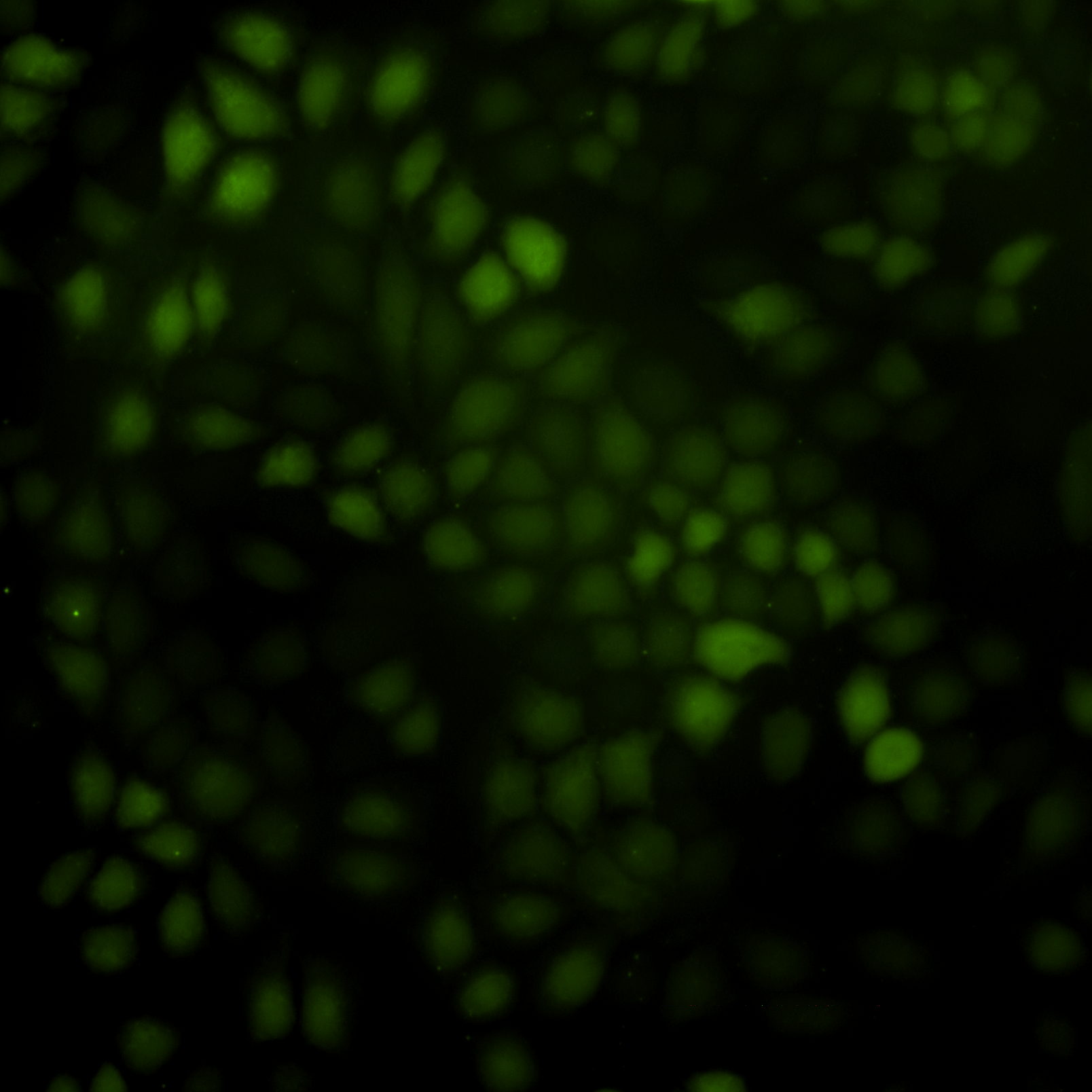

Supplement: Supplementary file 3 — Source data [file 41467_2022_35472_MOESM3_ESM.zip › Fig 5cd/5. DniCNC.tif]

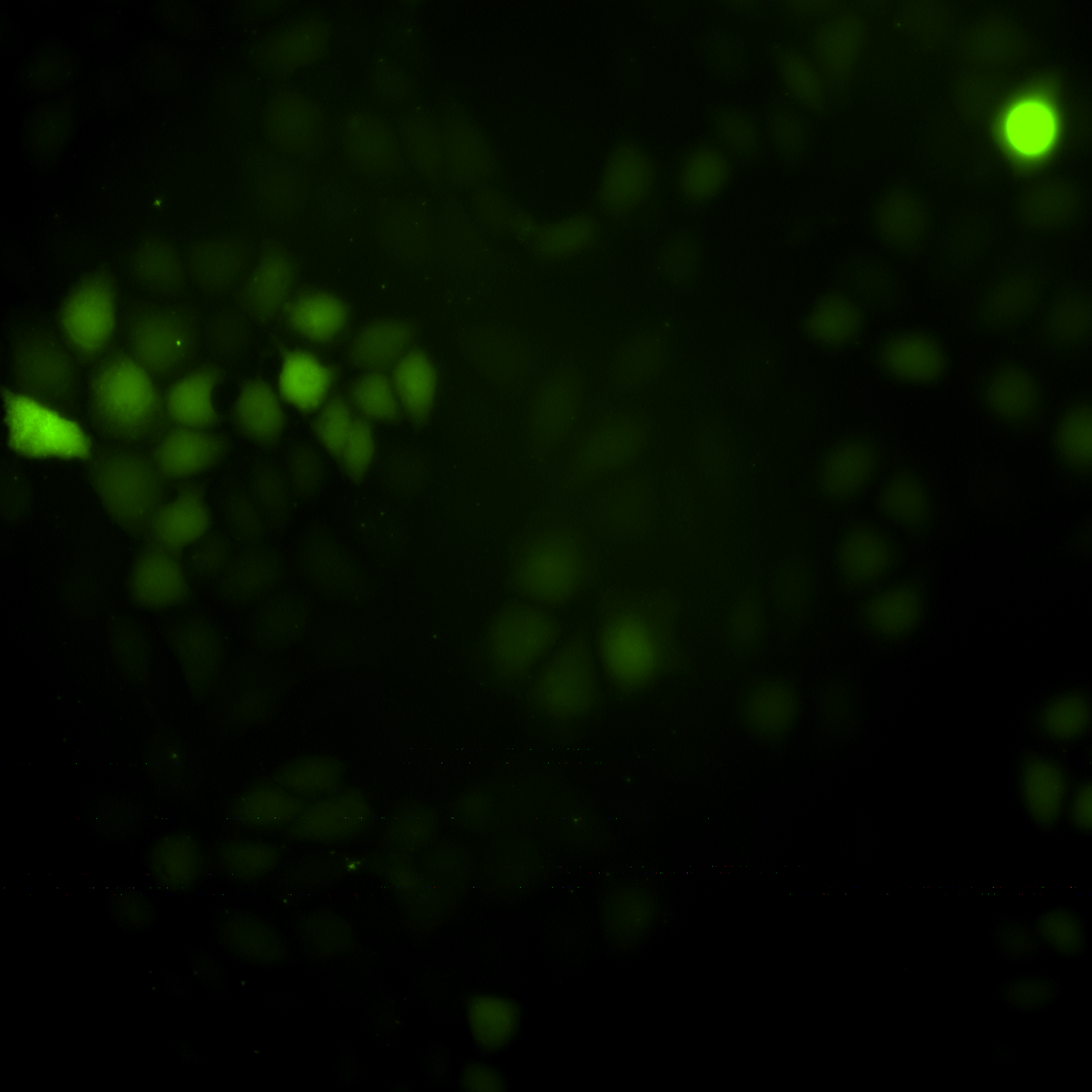

Supplement: Supplementary file 3 — Source data [file 41467_2022_35472_MOESM3_ESM.zip › Fig 5cd/6. DCNC.tif]

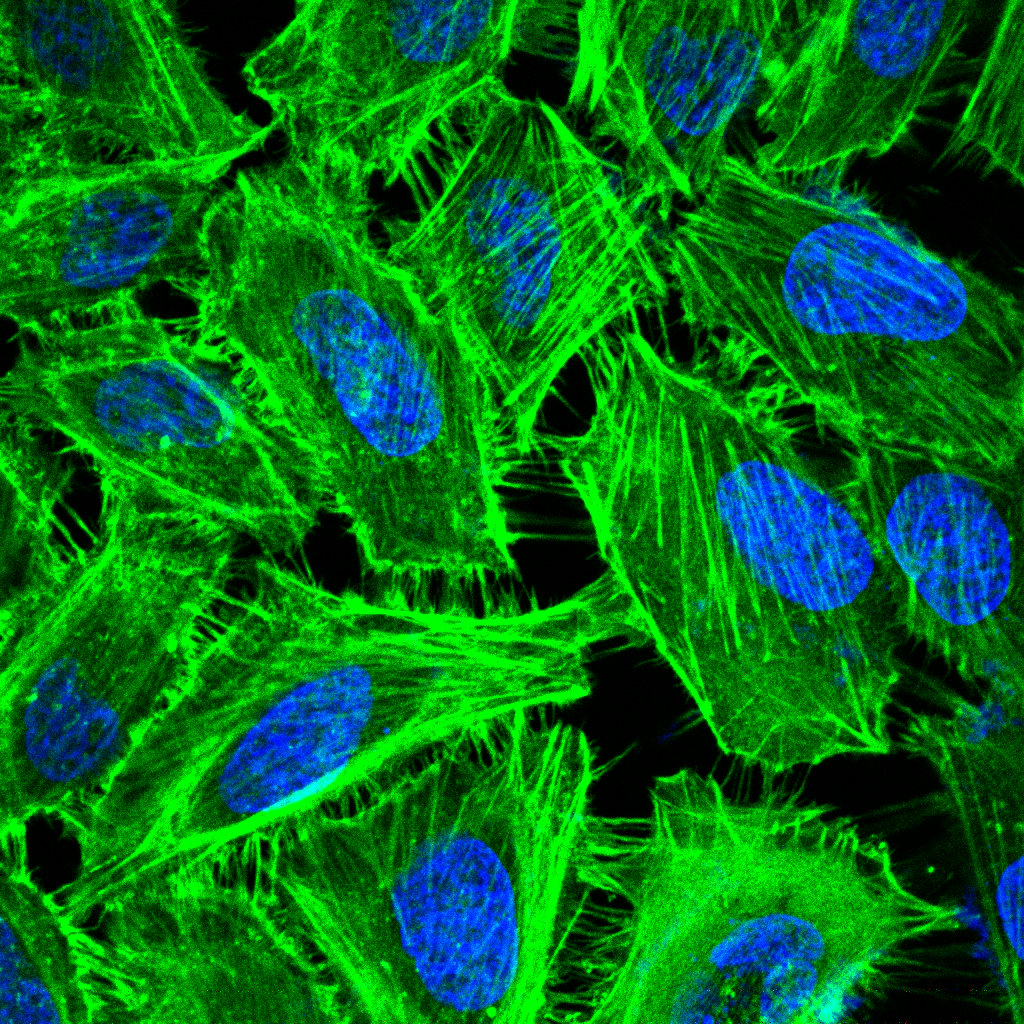

Supplement: Supplementary file 3 — Source data [file 41467_2022_35472_MOESM3_ESM.zip › Fig 5g/1. Blank.tif]

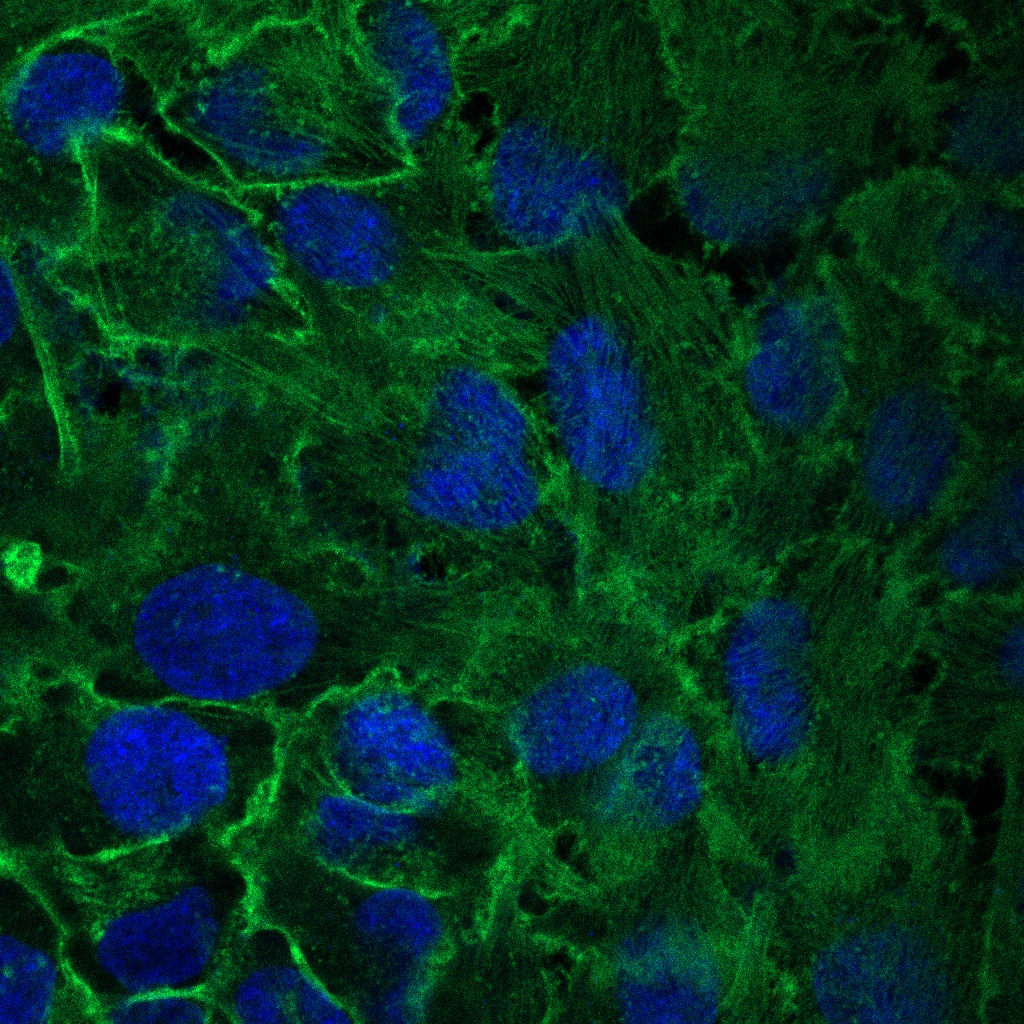

Supplement: Supplementary file 3 — Source data [file 41467_2022_35472_MOESM3_ESM.zip › Fig 5g/2. PBS.jpg]

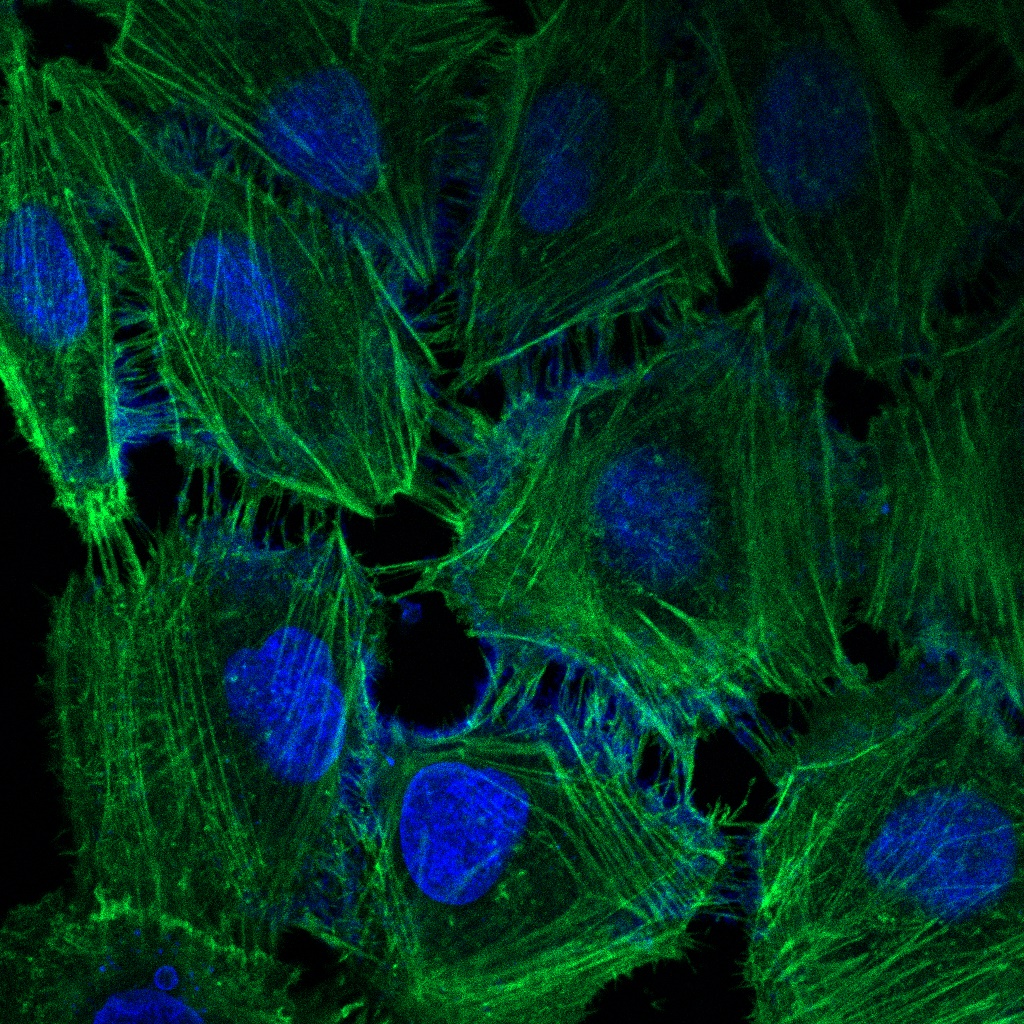

Supplement: Supplementary file 3 — Source data [file 41467_2022_35472_MOESM3_ESM.zip › Fig 5g/3. DniAS.jpg]

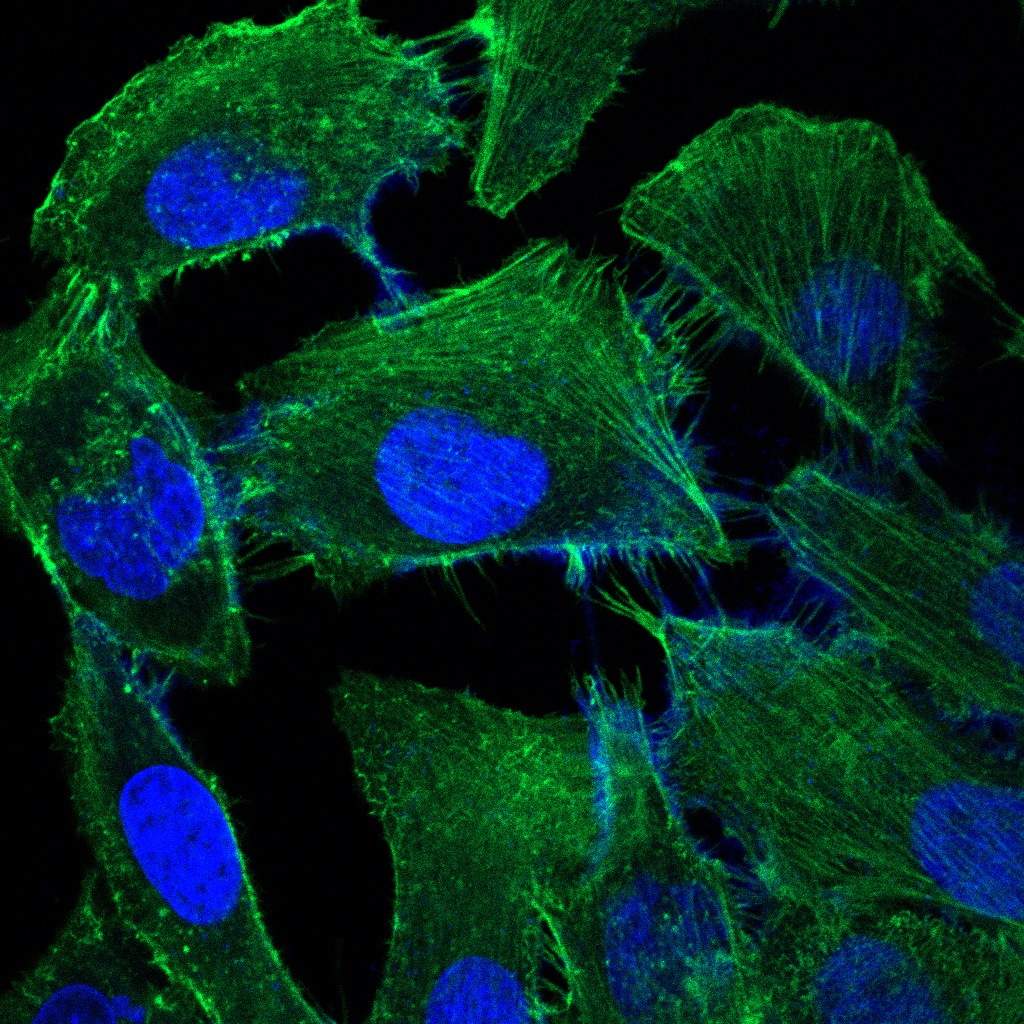

Supplement: Supplementary file 3 — Source data [file 41467_2022_35472_MOESM3_ESM.zip › Fig 5g/4. DAS.jpg]

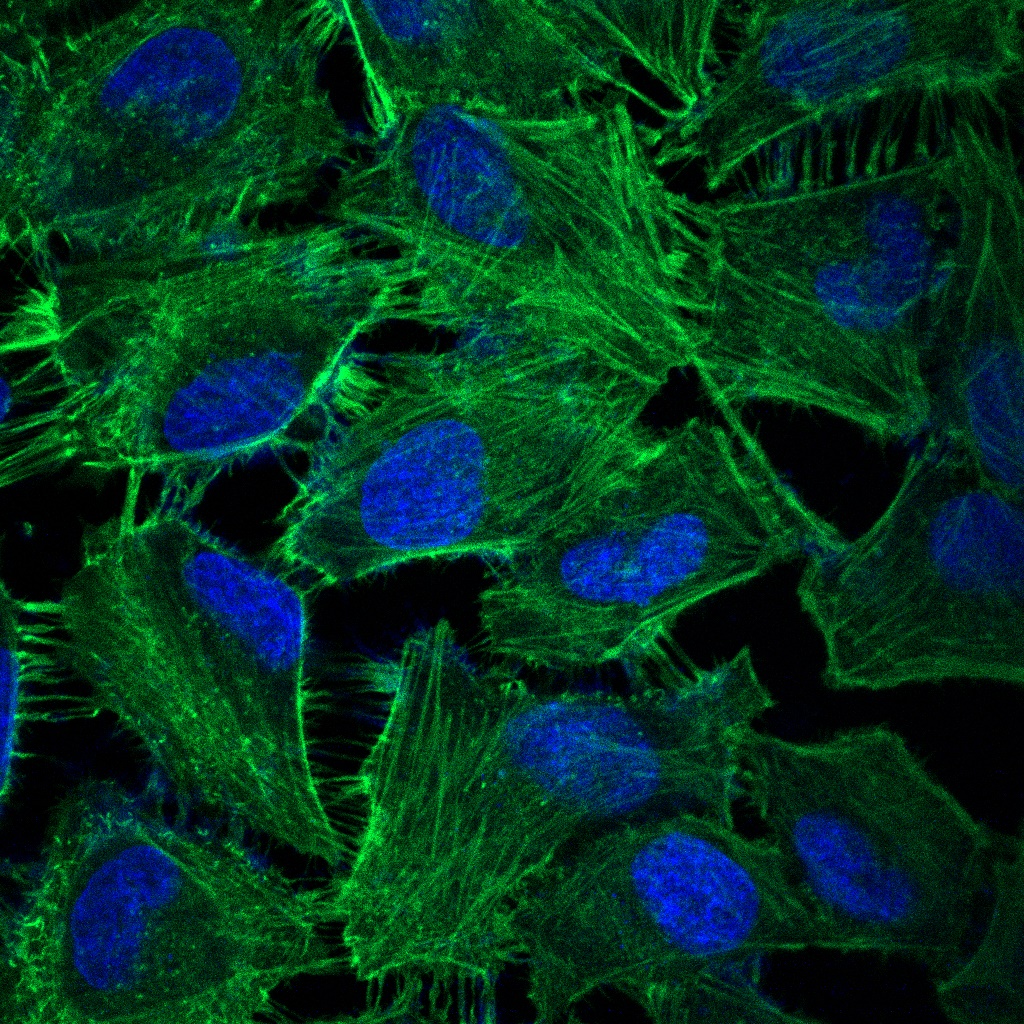

Supplement: Supplementary file 3 — Source data [file 41467_2022_35472_MOESM3_ESM.zip › Fig 5g/5. DniCNC.jpg]

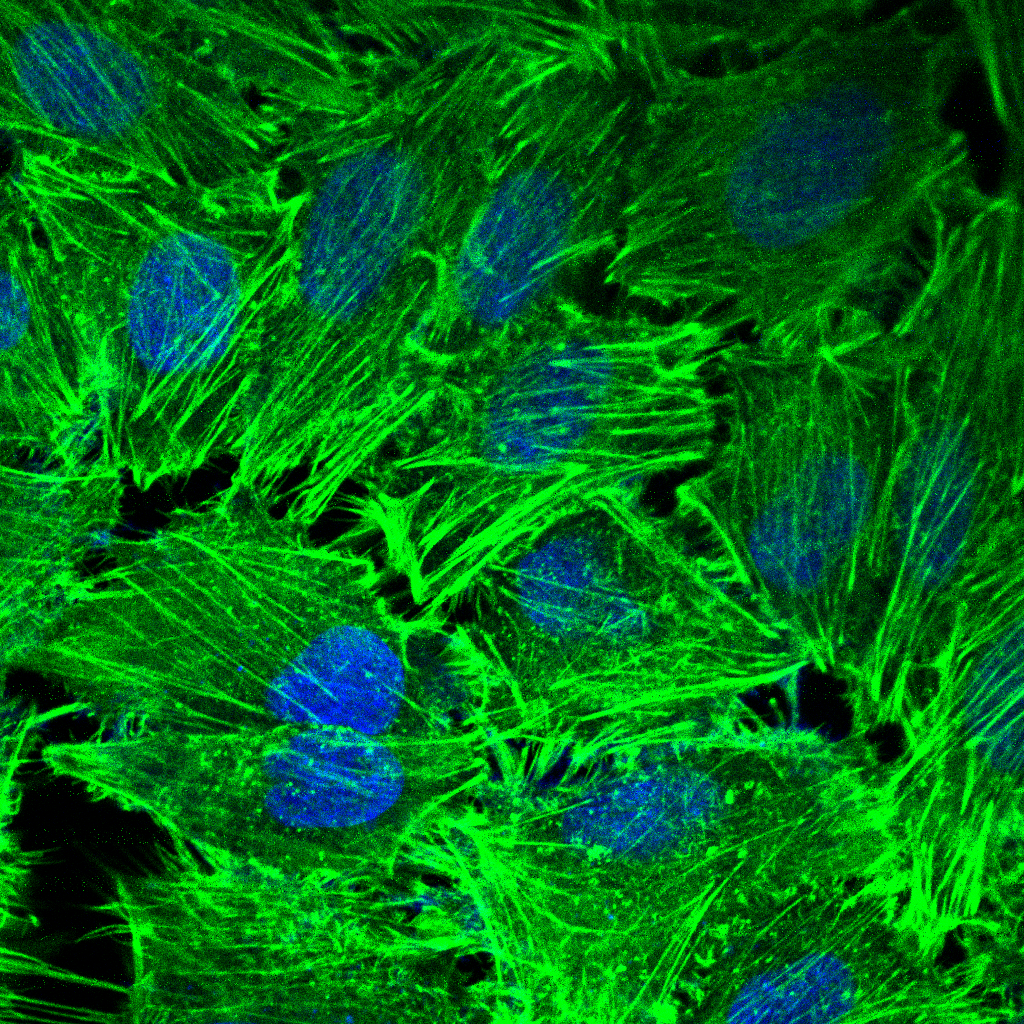

Supplement: Supplementary file 3 — Source data [file 41467_2022_35472_MOESM3_ESM.zip › Fig 5g/6. DCNC.tif]

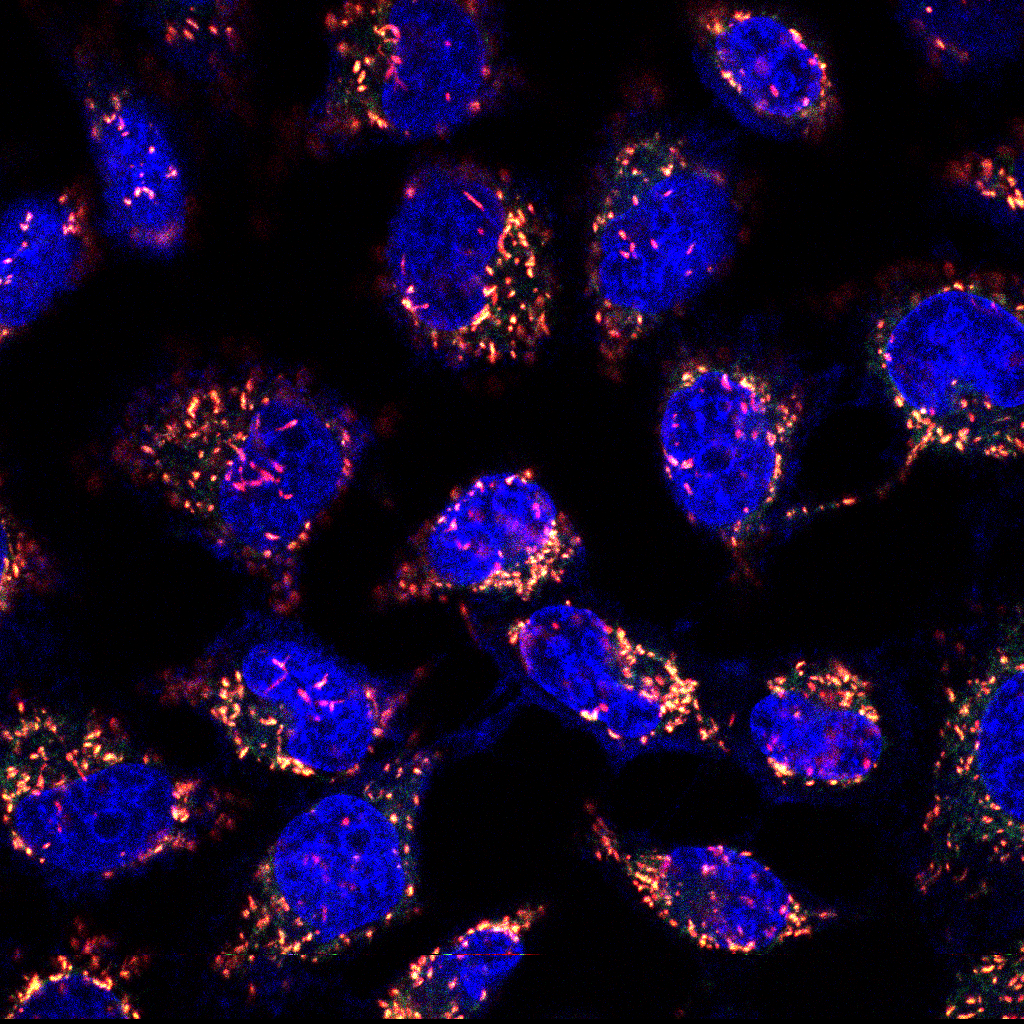

Supplement: Supplementary file 3 — Source data [file 41467_2022_35472_MOESM3_ESM.zip › Fig 6b/Blank/Blank_c1-3.tif]

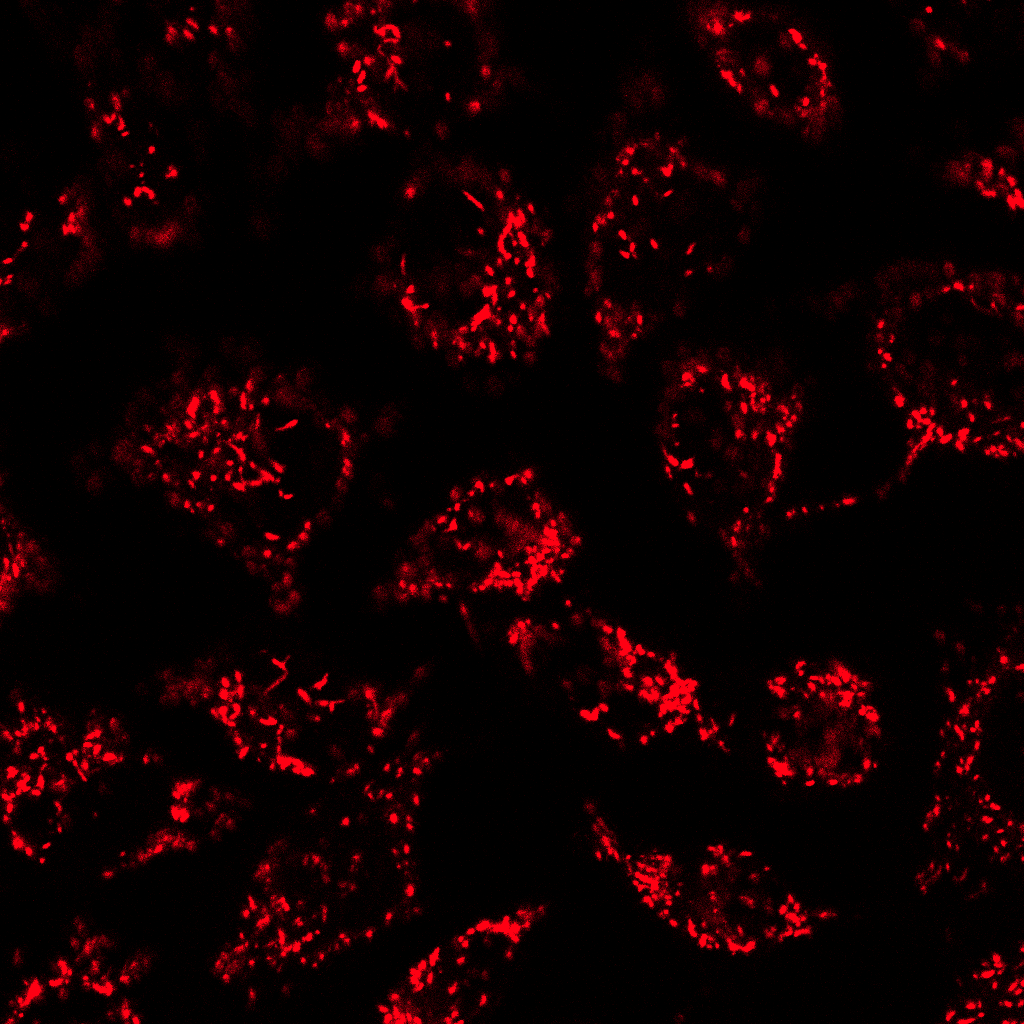

Supplement: Supplementary file 3 — Source data [file 41467_2022_35472_MOESM3_ESM.zip › Fig 6b/Blank/Blank_c1.tif]

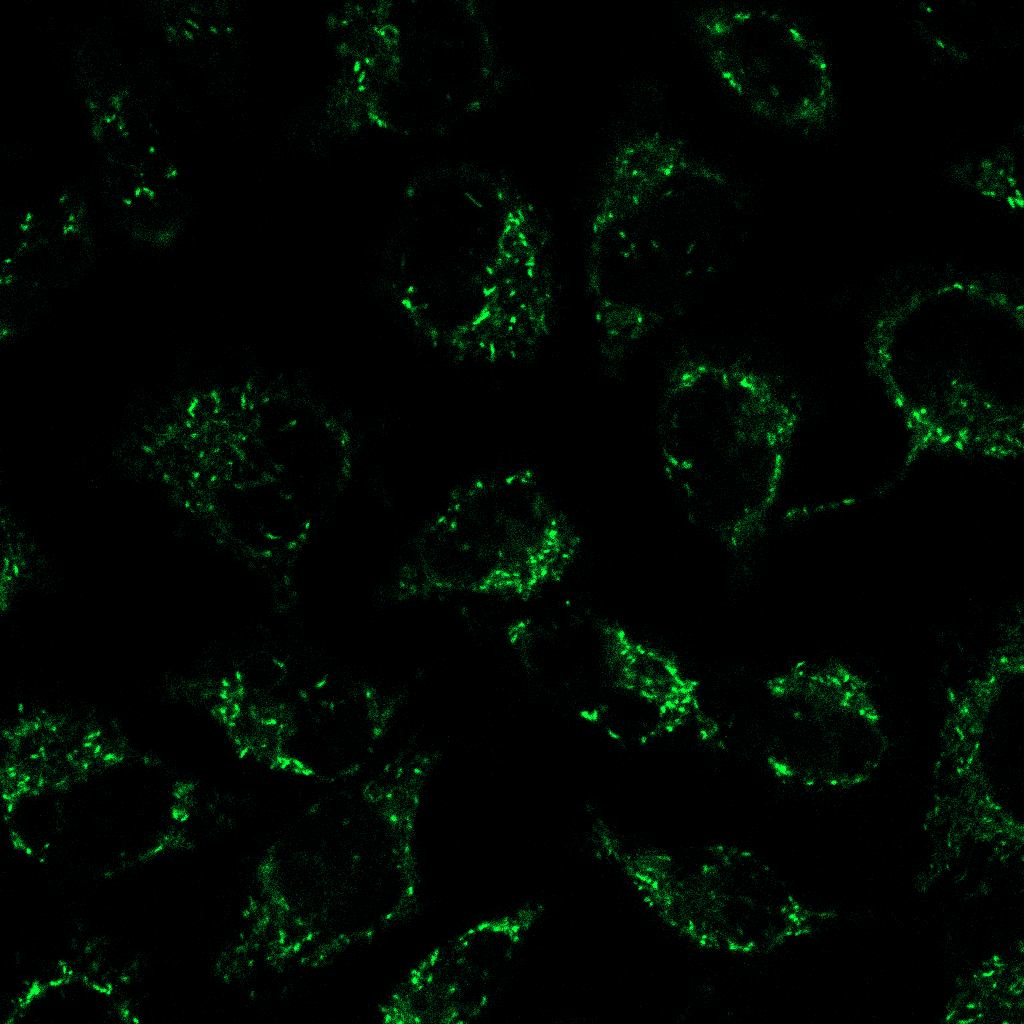

Supplement: Supplementary file 3 — Source data [file 41467_2022_35472_MOESM3_ESM.zip › Fig 6b/Blank/Blank_c2.tif]

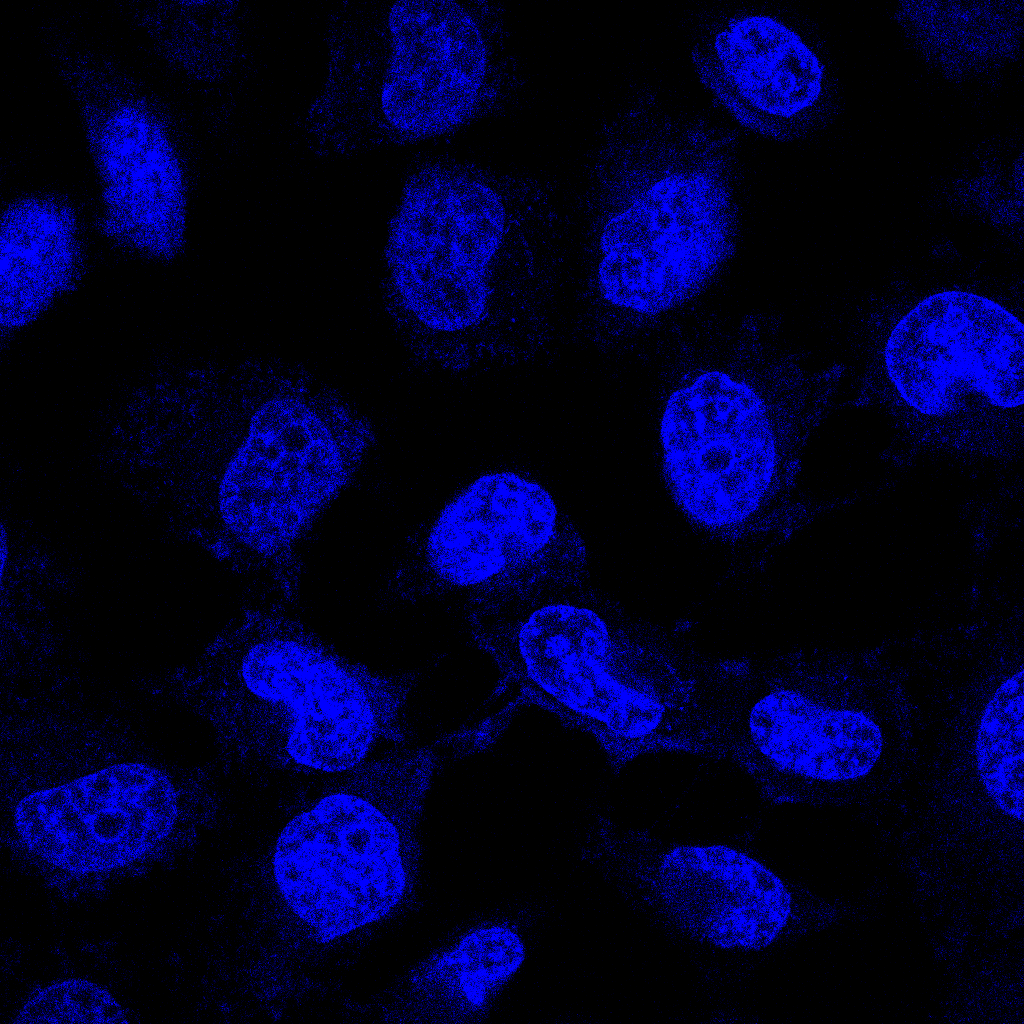

Supplement: Supplementary file 3 — Source data [file 41467_2022_35472_MOESM3_ESM.zip › Fig 6b/Blank/Blank_c3.tif]

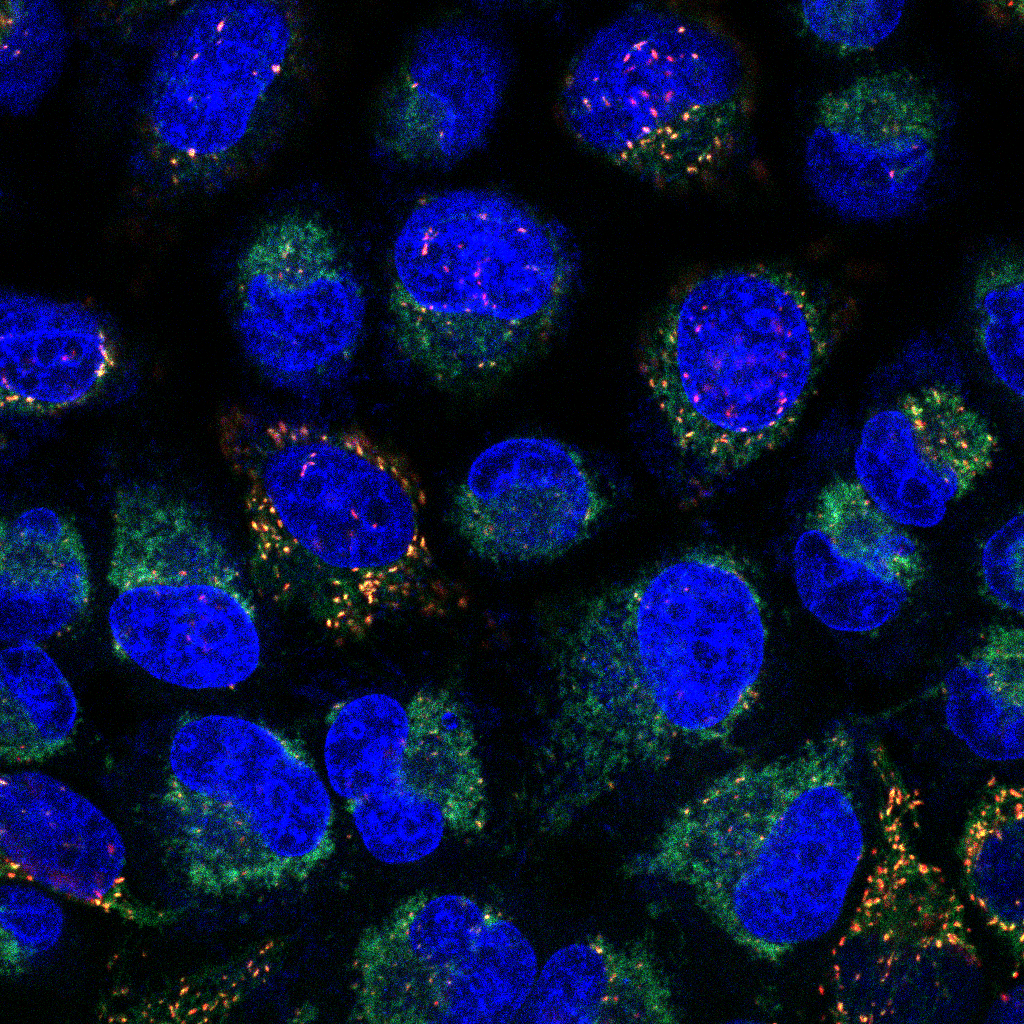

Supplement: Supplementary file 3 — Source data [file 41467_2022_35472_MOESM3_ESM.zip › Fig 6b/DAS/DAS_c1-3.tif]

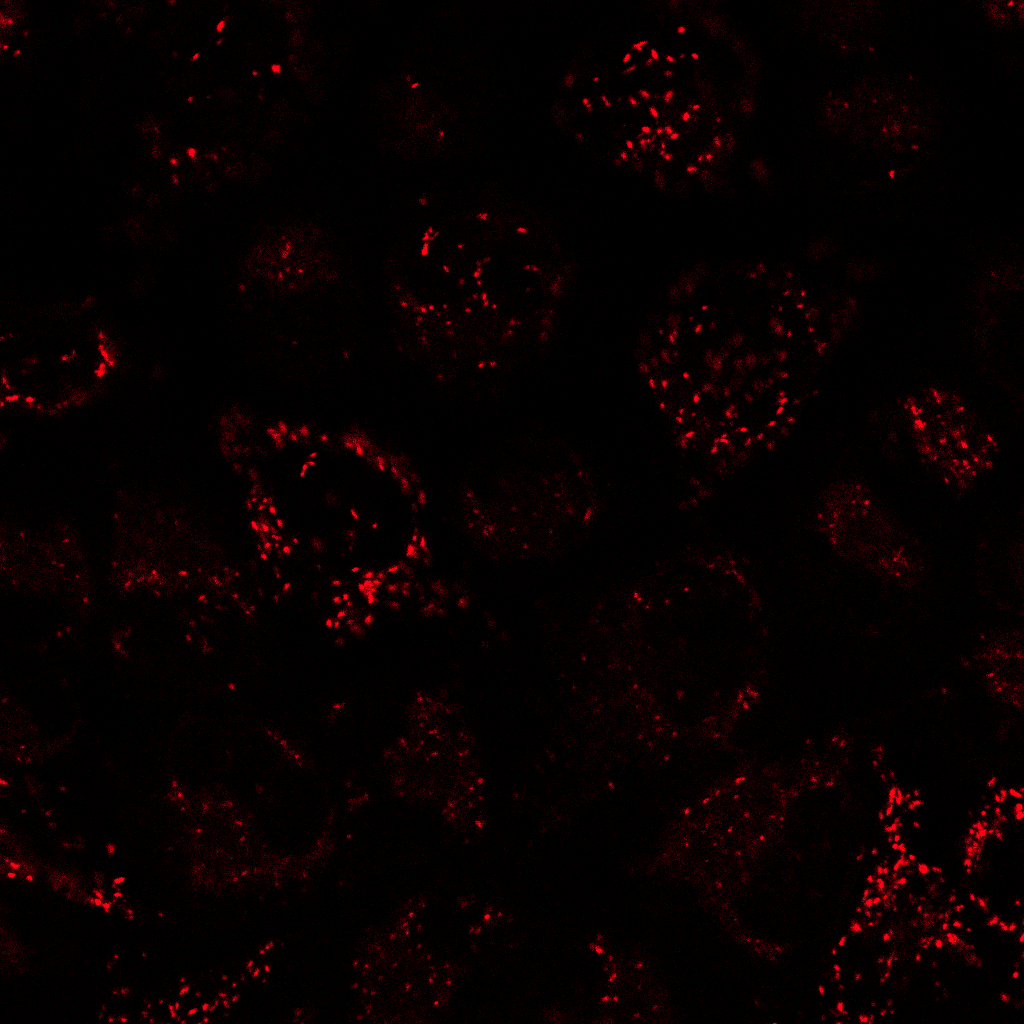

Supplement: Supplementary file 3 — Source data [file 41467_2022_35472_MOESM3_ESM.zip › Fig 6b/DAS/DAS_c1.tif]

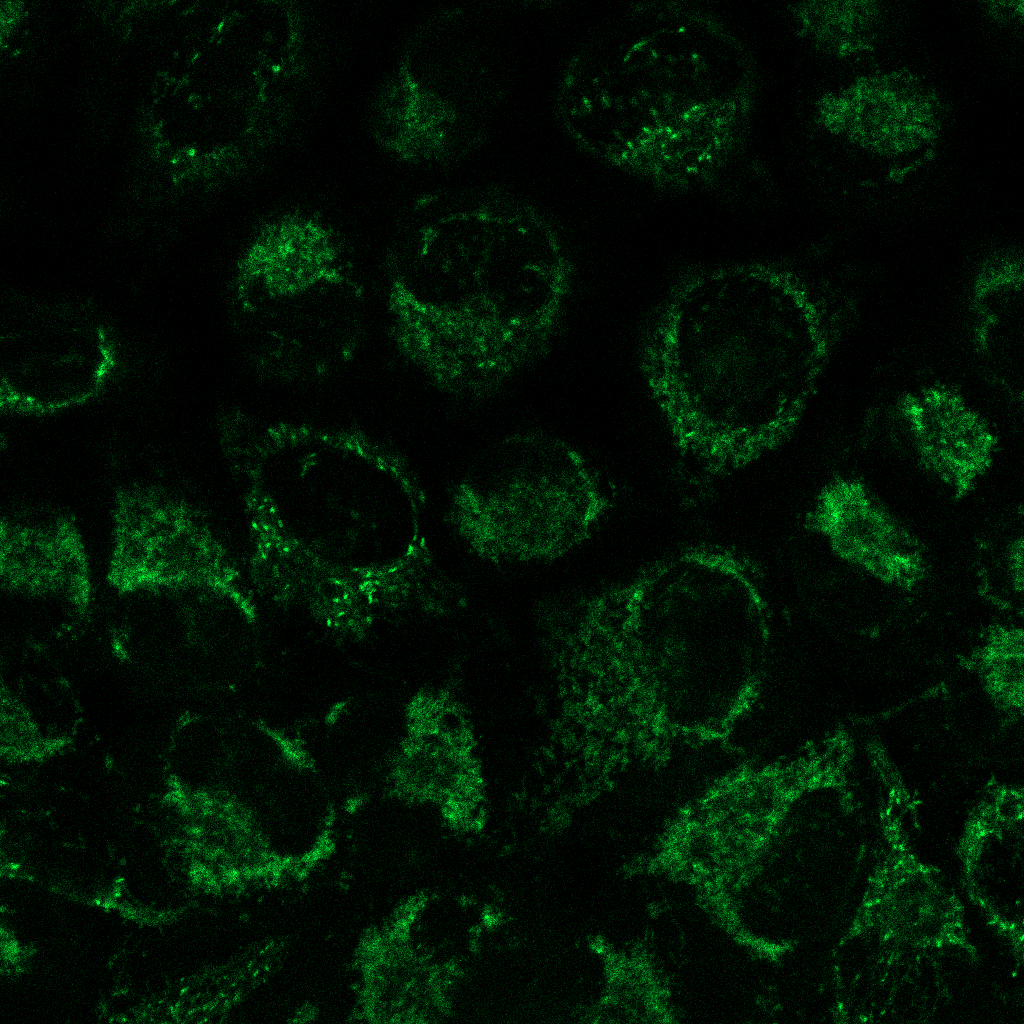

Supplement: Supplementary file 3 — Source data [file 41467_2022_35472_MOESM3_ESM.zip › Fig 6b/DAS/DAS_c2.tif]

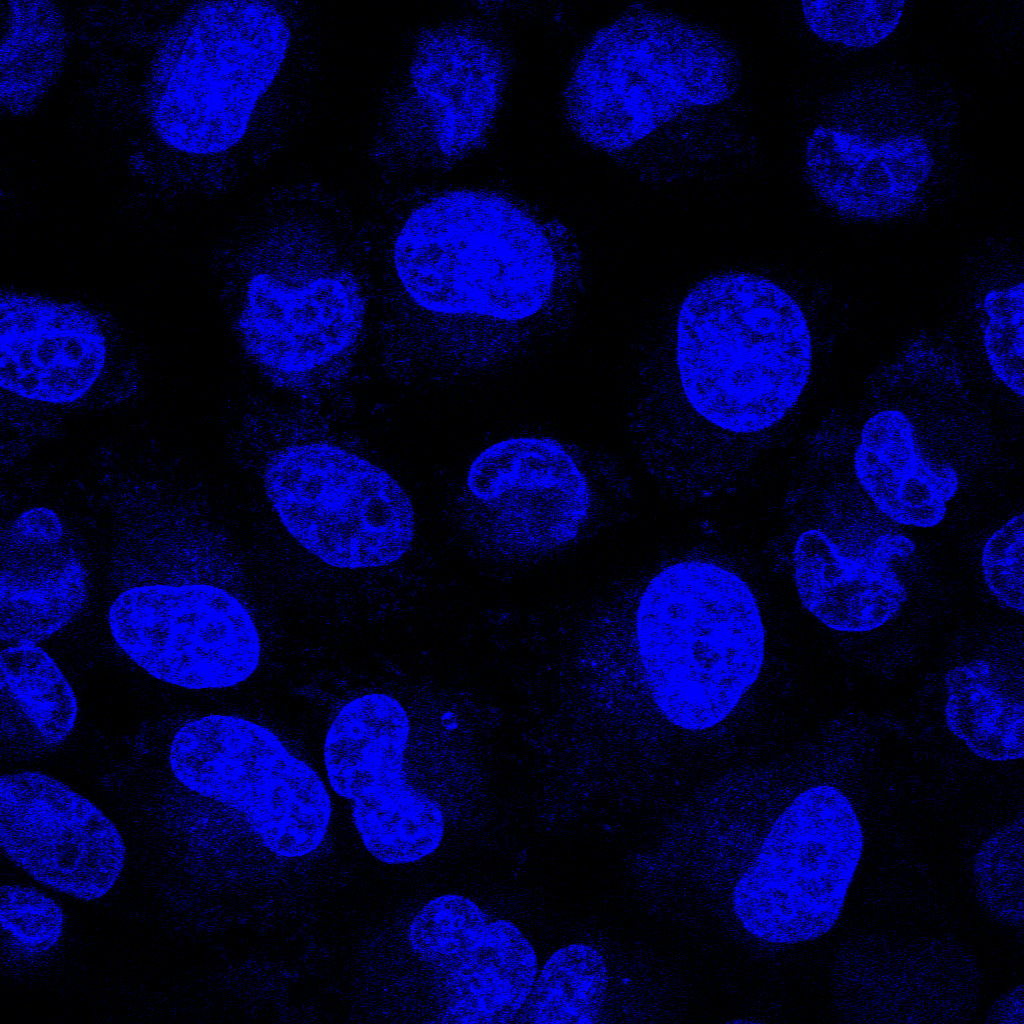

Supplement: Supplementary file 3 — Source data [file 41467_2022_35472_MOESM3_ESM.zip › Fig 6b/DAS/DAS_c3.tif]

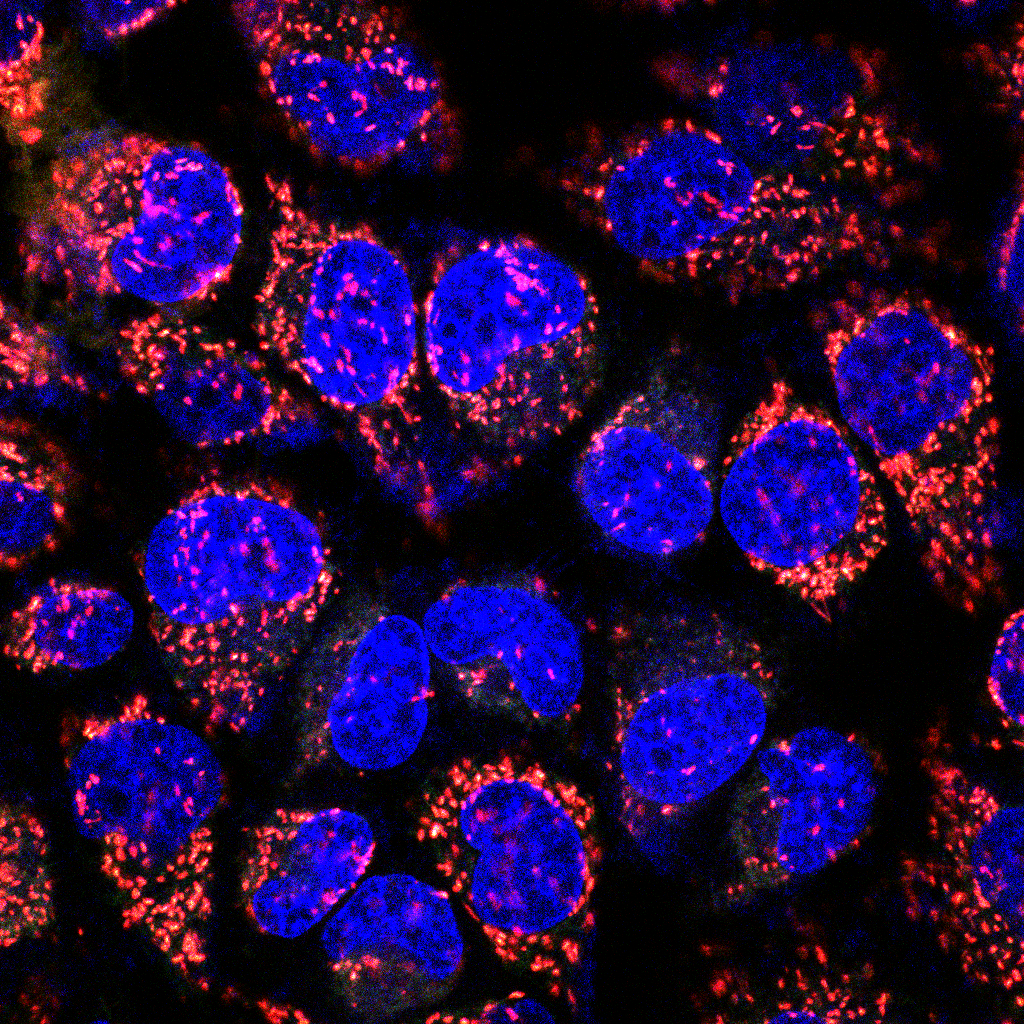

Supplement: Supplementary file 3 — Source data [file 41467_2022_35472_MOESM3_ESM.zip › Fig 6b/DCNC/DCNC_c1-3.tif]

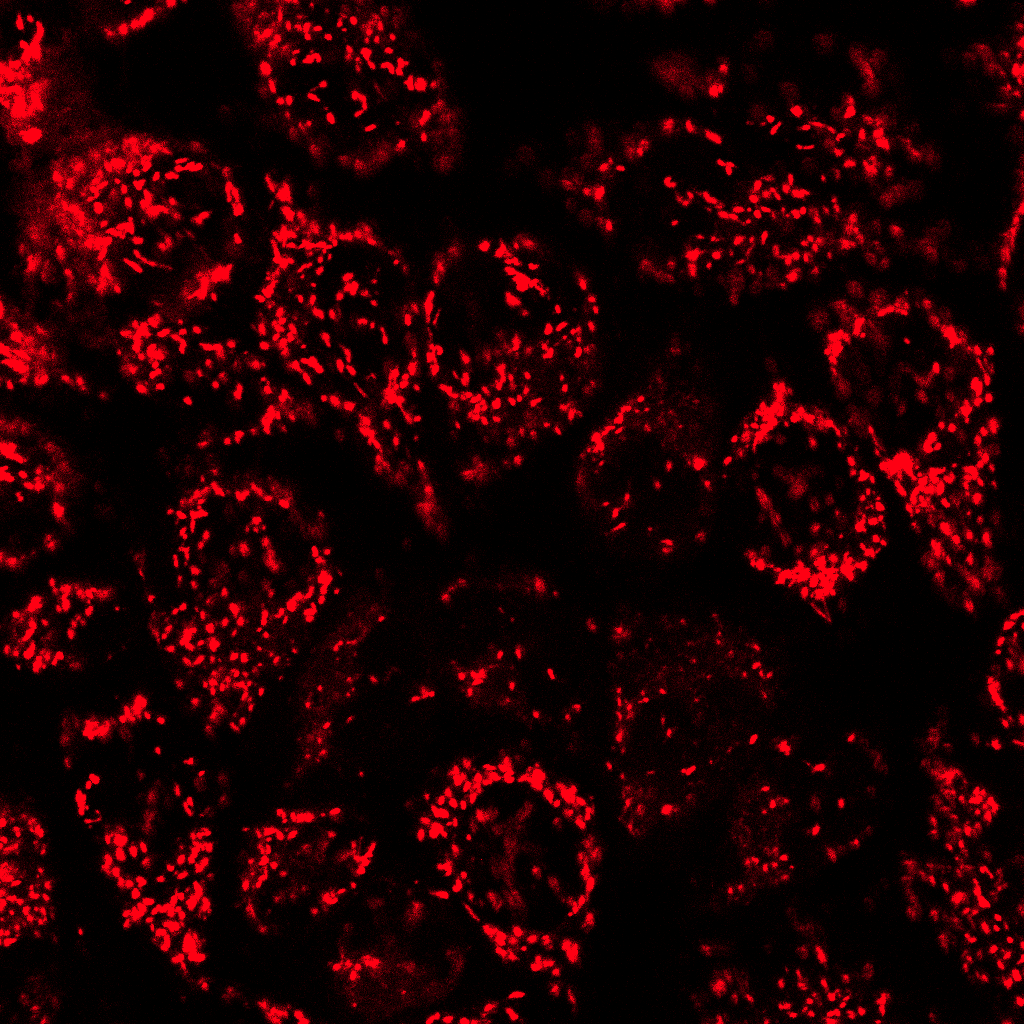

Supplement: Supplementary file 3 — Source data [file 41467_2022_35472_MOESM3_ESM.zip › Fig 6b/DCNC/DCNC_c1.tif]

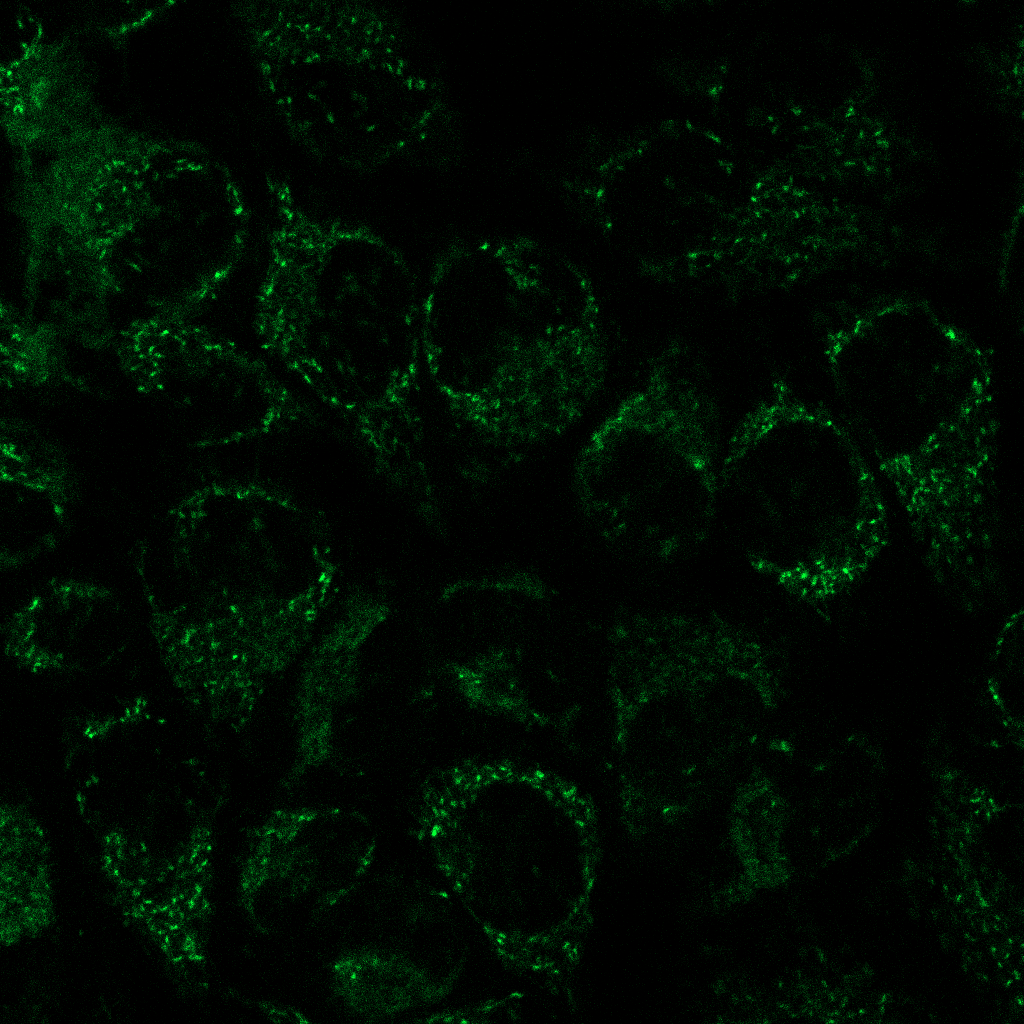

Supplement: Supplementary file 3 — Source data [file 41467_2022_35472_MOESM3_ESM.zip › Fig 6b/DCNC/DCNC_c2.tif]

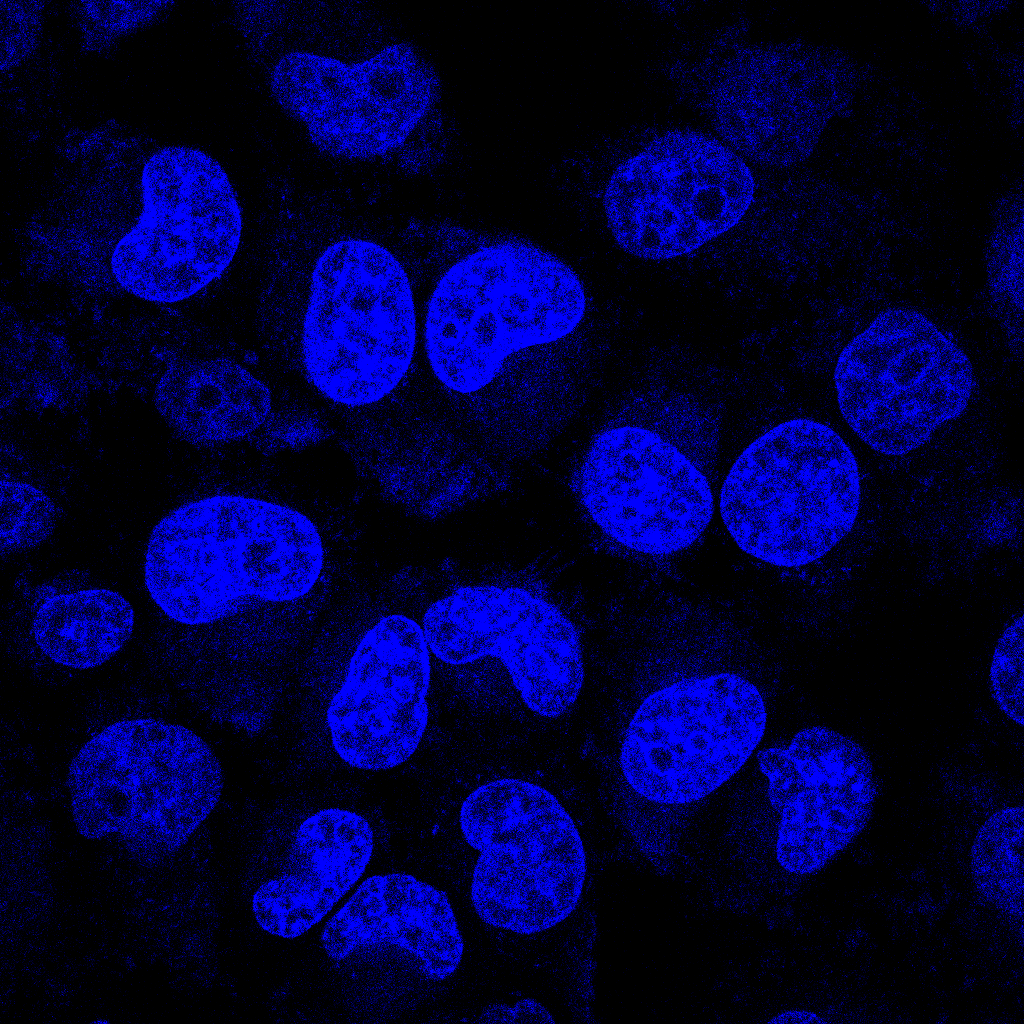

Supplement: Supplementary file 3 — Source data [file 41467_2022_35472_MOESM3_ESM.zip › Fig 6b/DCNC/DCNC_c3.tif]

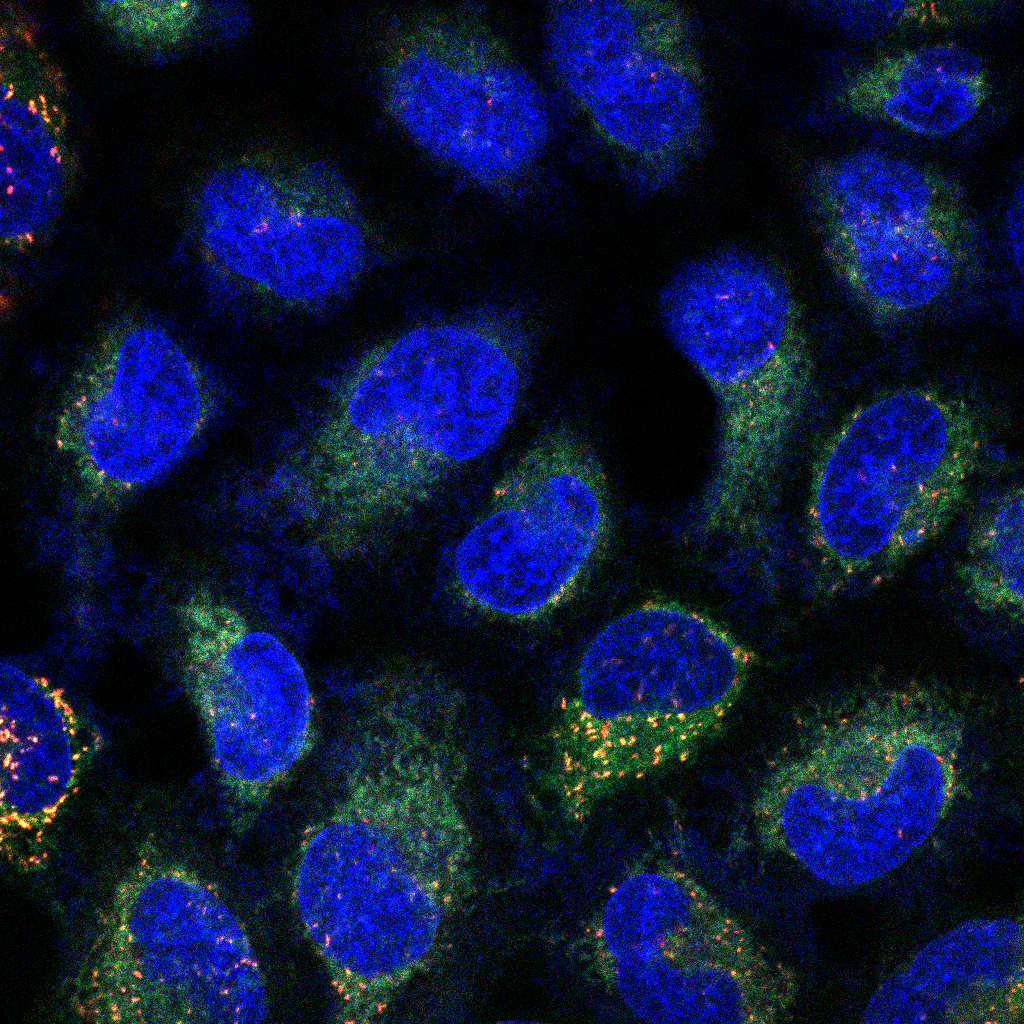

Supplement: Supplementary file 3 — Source data [file 41467_2022_35472_MOESM3_ESM.zip › Fig 6b/DniAS/DniAS_c1-3.tif]

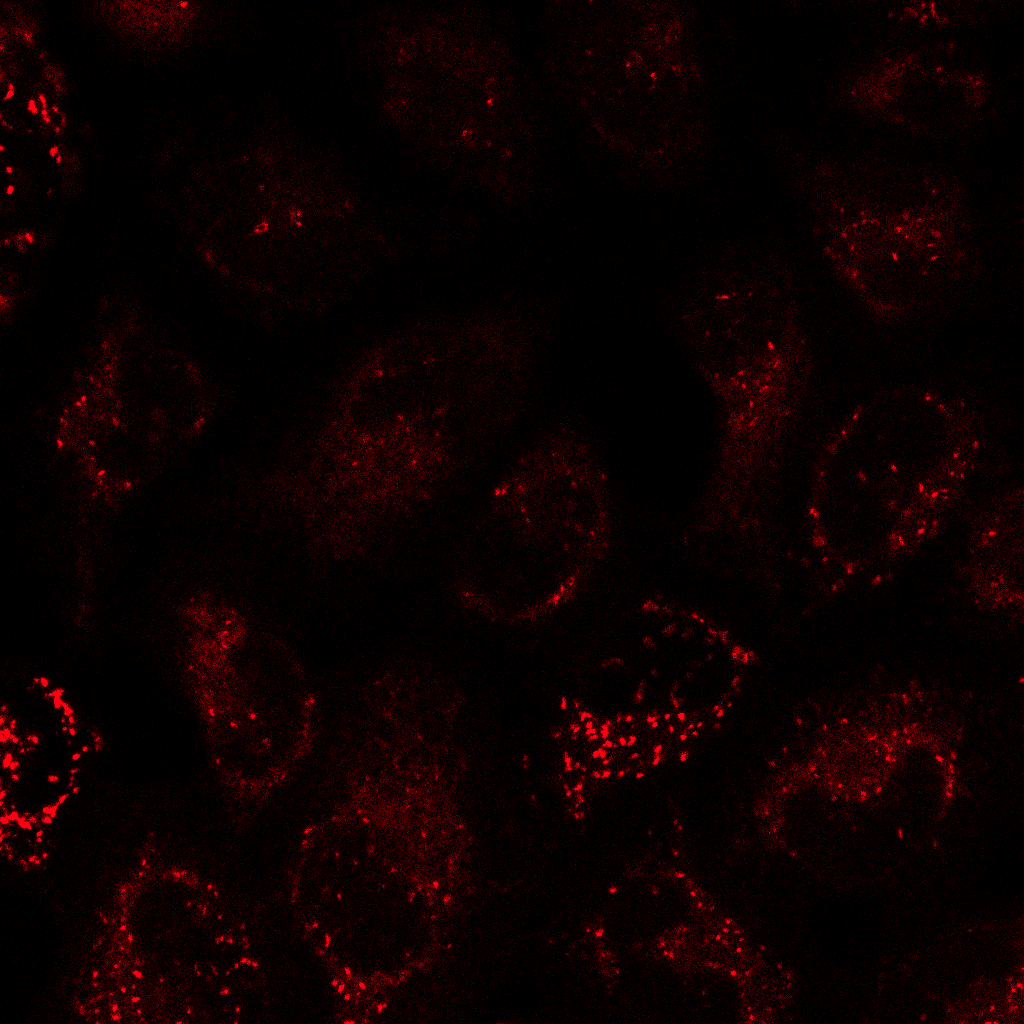

Supplement: Supplementary file 3 — Source data [file 41467_2022_35472_MOESM3_ESM.zip › Fig 6b/DniAS/DniAS_c1.tif]

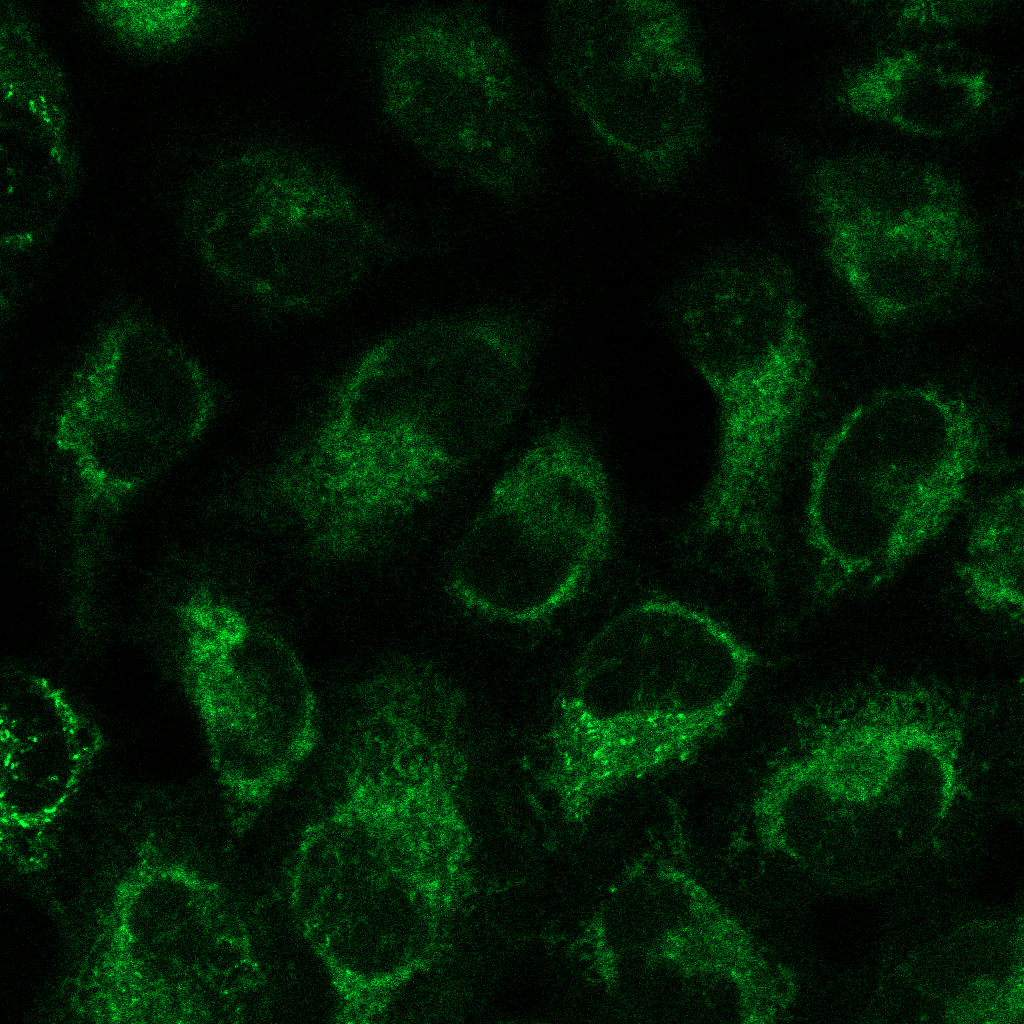

Supplement: Supplementary file 3 — Source data [file 41467_2022_35472_MOESM3_ESM.zip › Fig 6b/DniAS/DniAS_c2.tif]

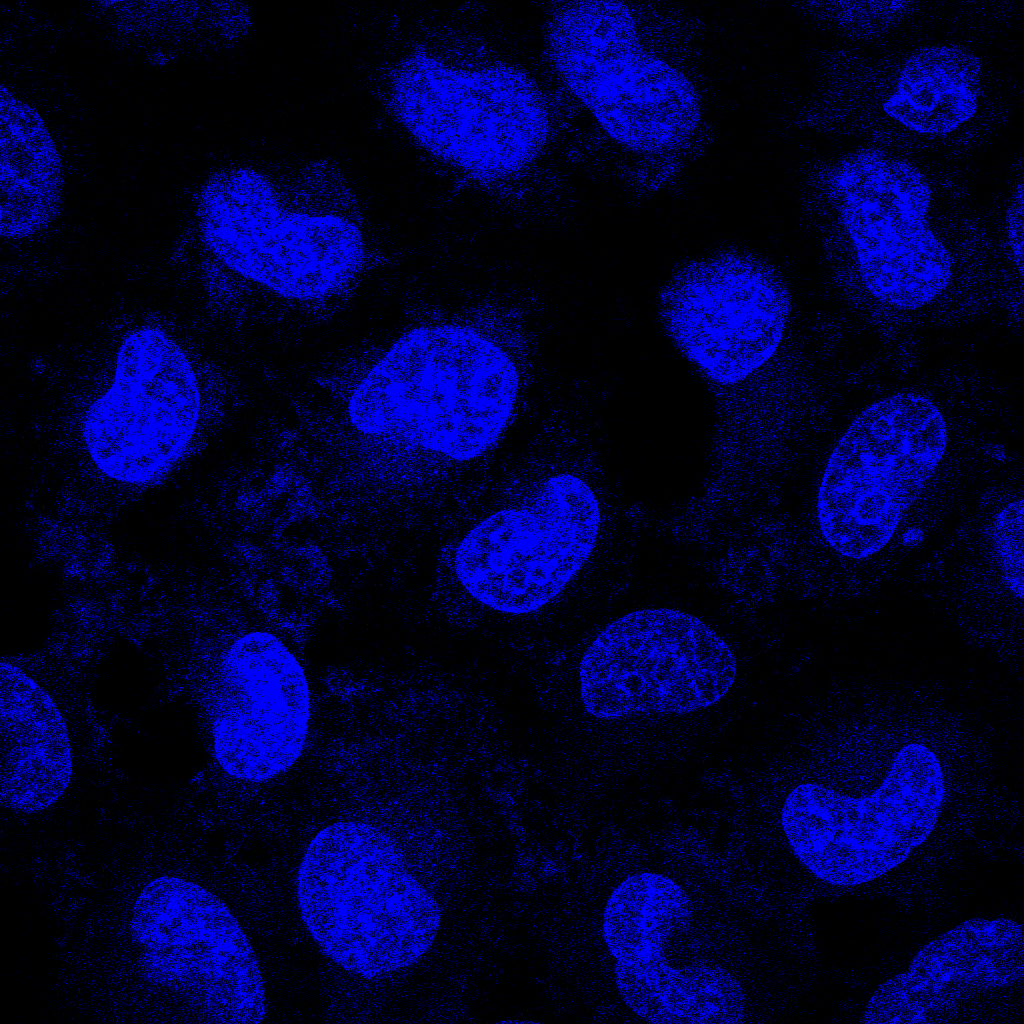

Supplement: Supplementary file 3 — Source data [file 41467_2022_35472_MOESM3_ESM.zip › Fig 6b/DniAS/DniAS_c3.tif]

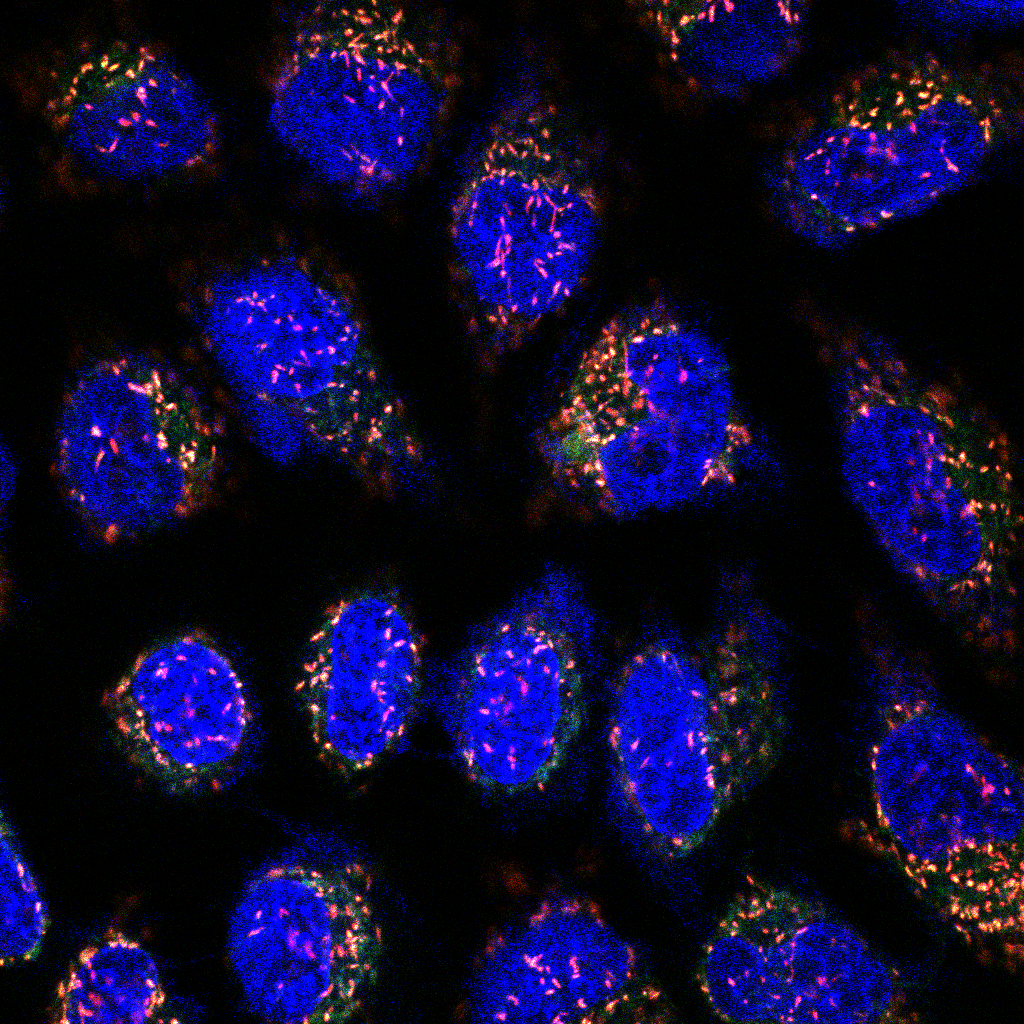

Supplement: Supplementary file 3 — Source data [file 41467_2022_35472_MOESM3_ESM.zip › Fig 6b/DniCNC/DniCNC_c1-3.tif]

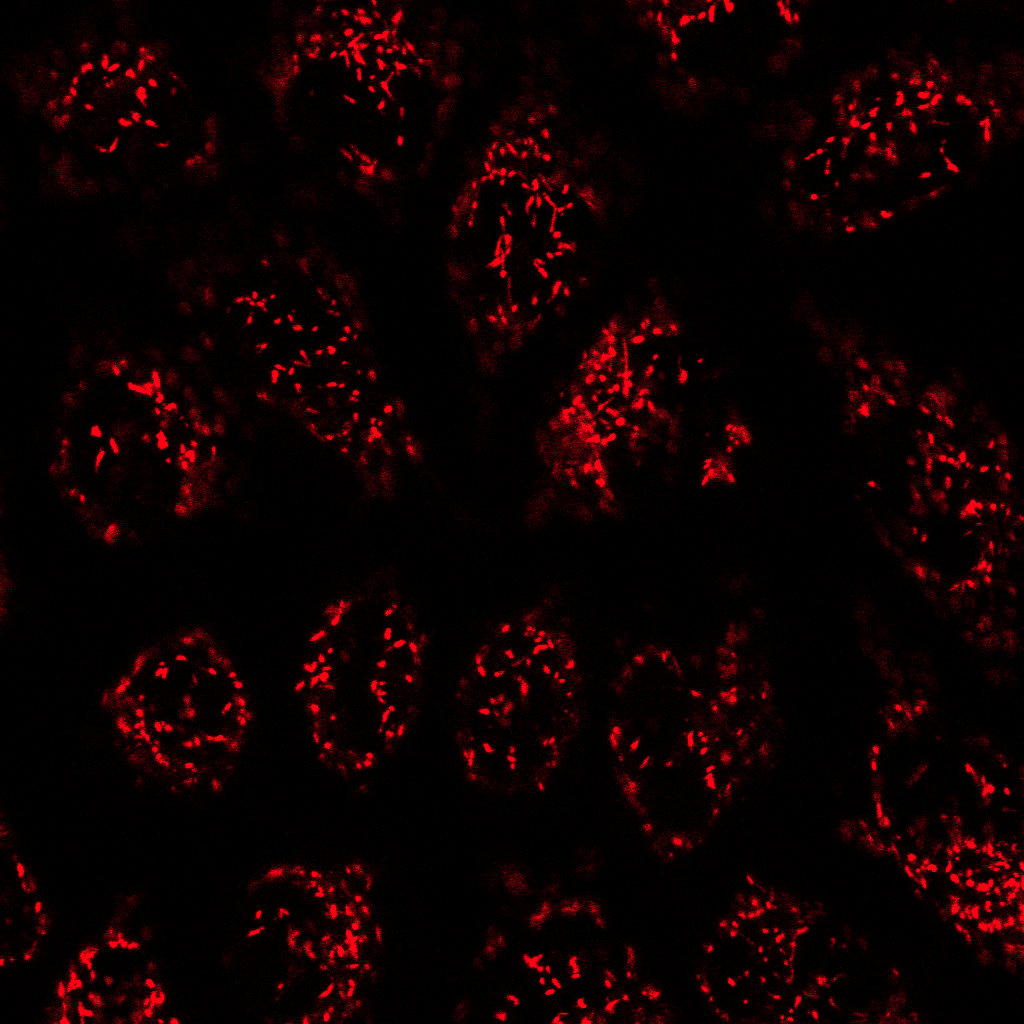

Supplement: Supplementary file 3 — Source data [file 41467_2022_35472_MOESM3_ESM.zip › Fig 6b/DniCNC/DniCNC_c1.tif]

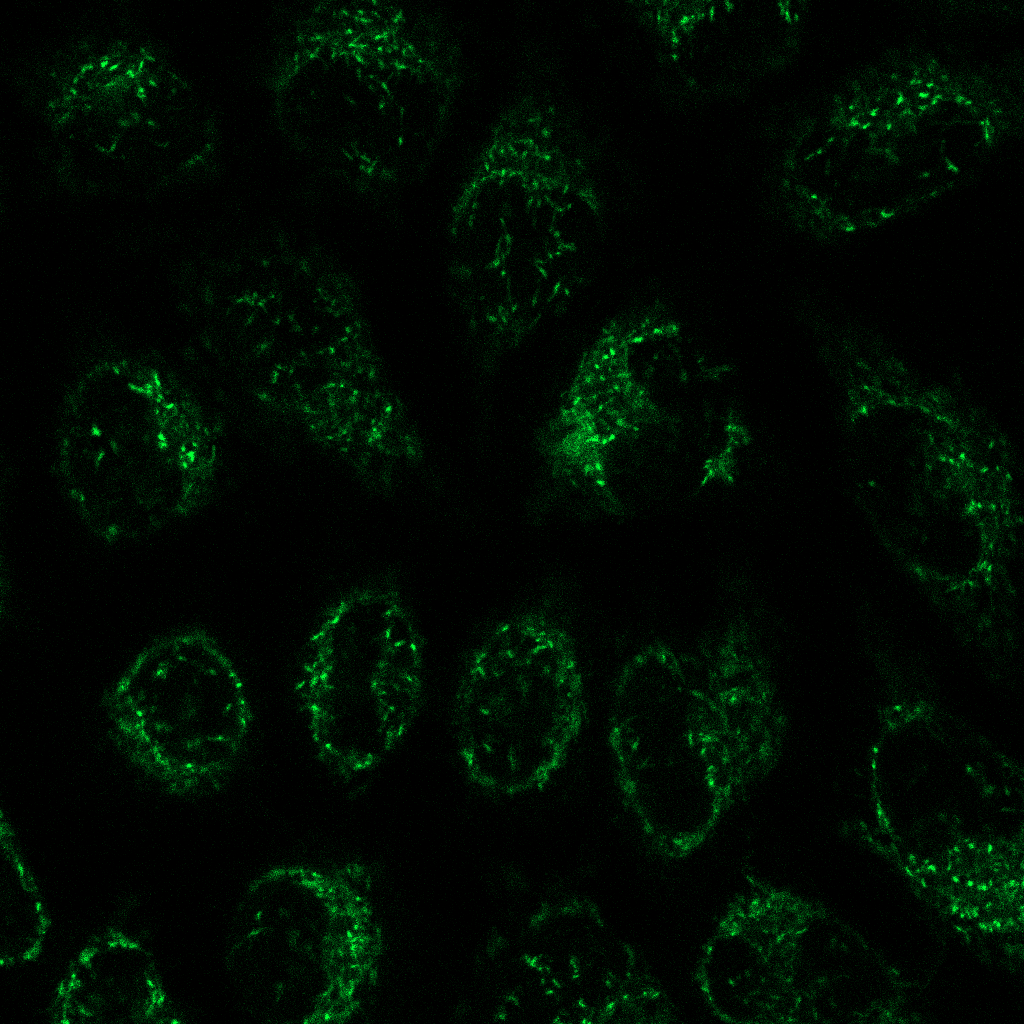

Supplement: Supplementary file 3 — Source data [file 41467_2022_35472_MOESM3_ESM.zip › Fig 6b/DniCNC/DniCNC_c2.tif]

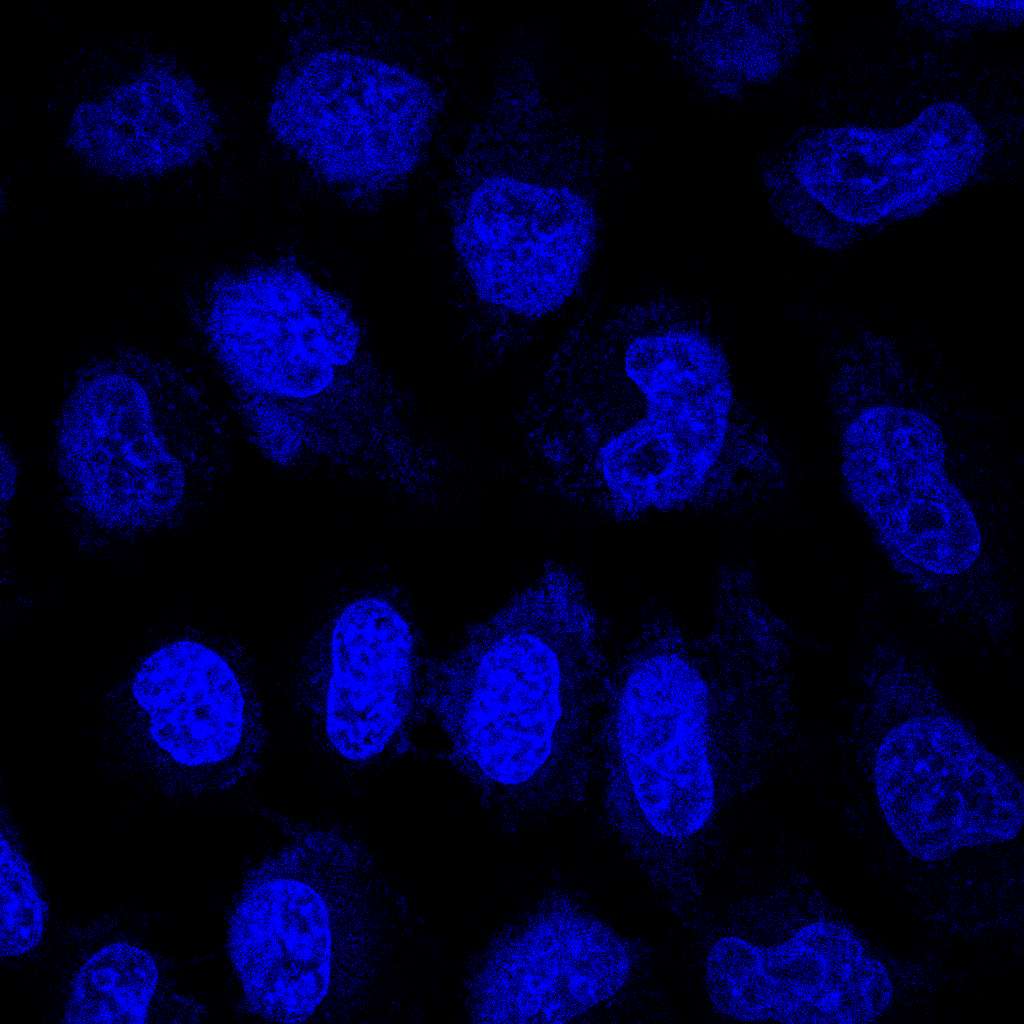

Supplement: Supplementary file 3 — Source data [file 41467_2022_35472_MOESM3_ESM.zip › Fig 6b/DniCNC/DniCNC_c3.tif]

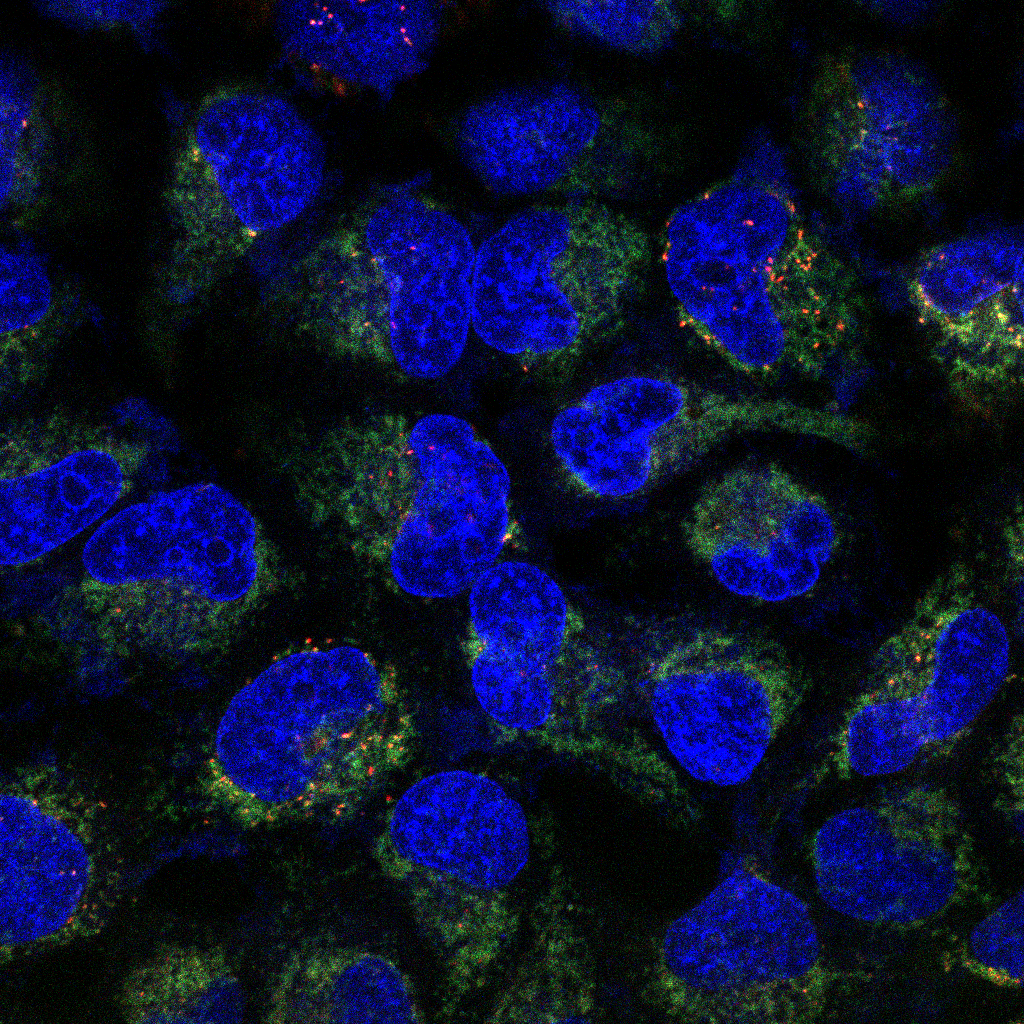

Supplement: Supplementary file 3 — Source data [file 41467_2022_35472_MOESM3_ESM.zip › Fig 6b/PBS/PBS_c1-3.tif]

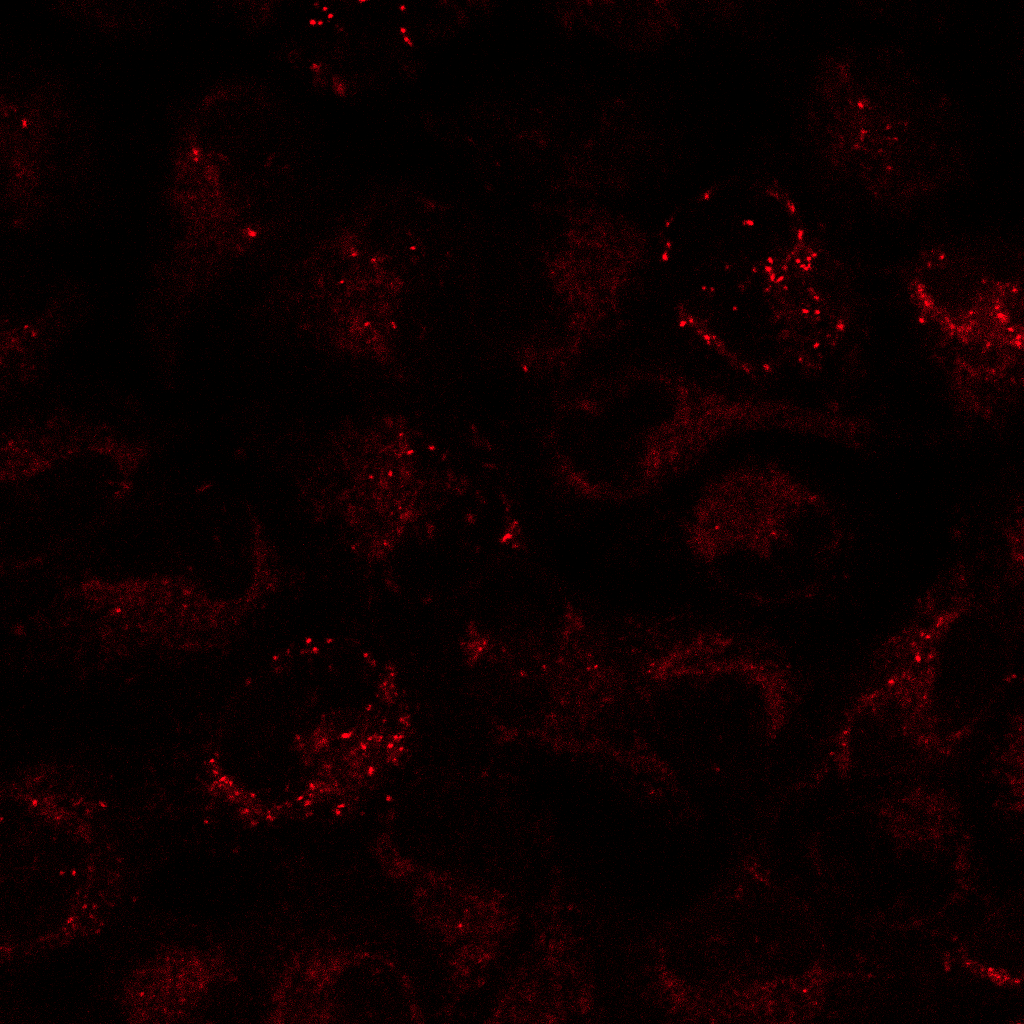

Supplement: Supplementary file 3 — Source data [file 41467_2022_35472_MOESM3_ESM.zip › Fig 6b/PBS/PBS_c1.tif]

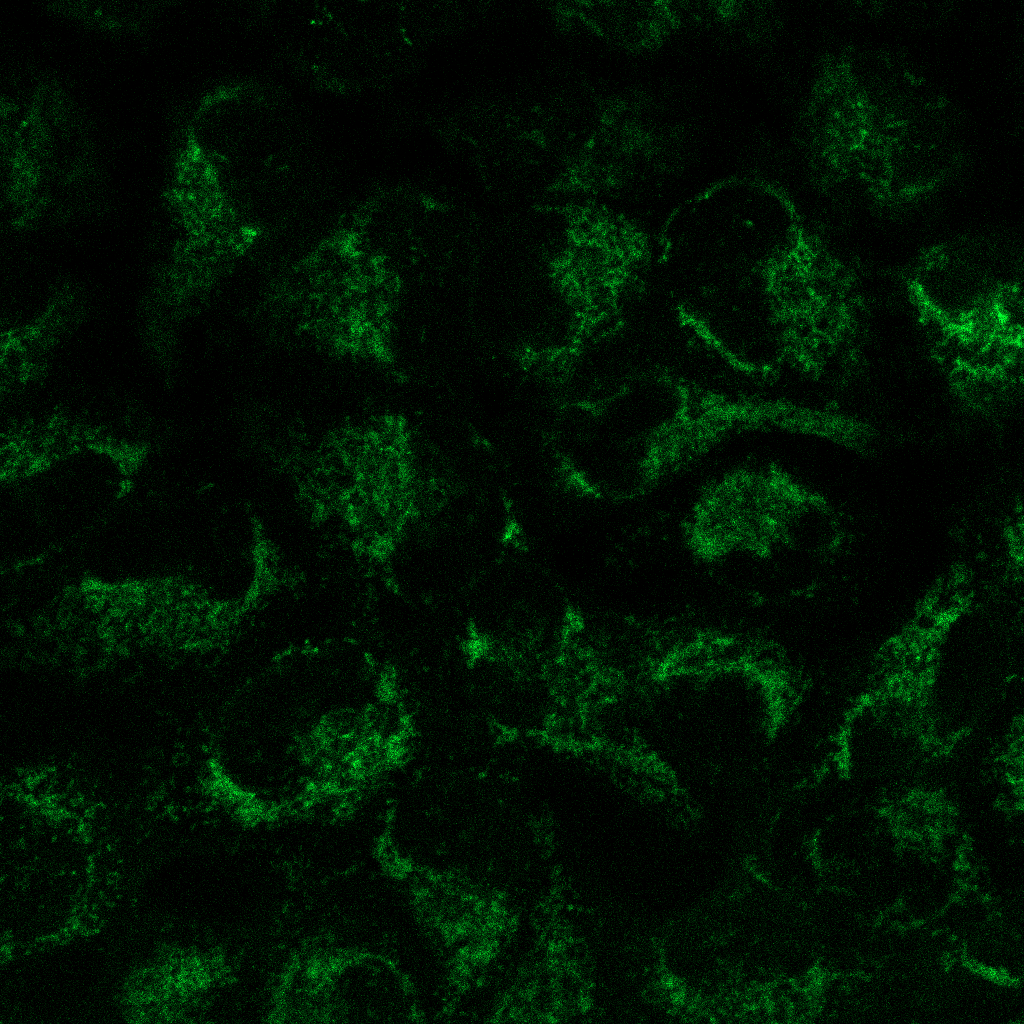

Supplement: Supplementary file 3 — Source data [file 41467_2022_35472_MOESM3_ESM.zip › Fig 6b/PBS/PBS_c2.tif]

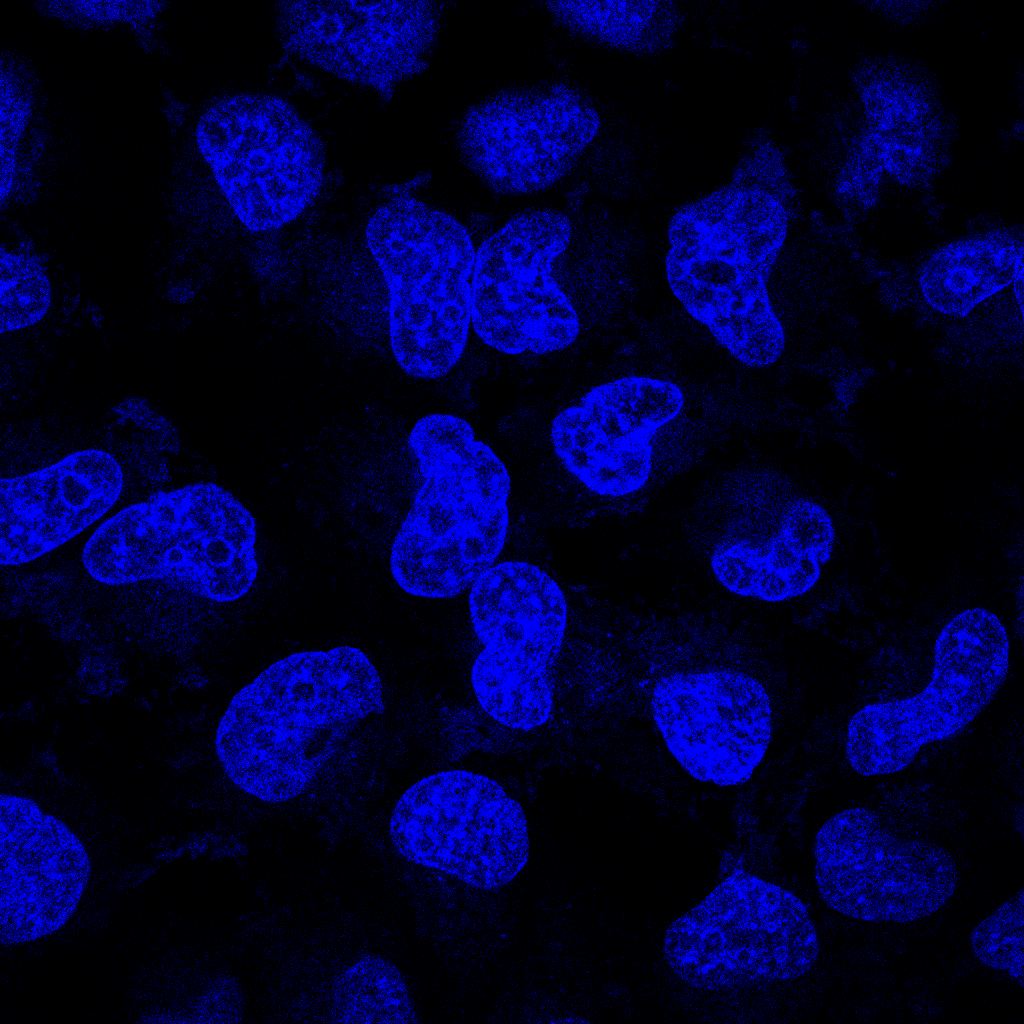

Supplement: Supplementary file 3 — Source data [file 41467_2022_35472_MOESM3_ESM.zip › Fig 6b/PBS/PBS_c3.tif]

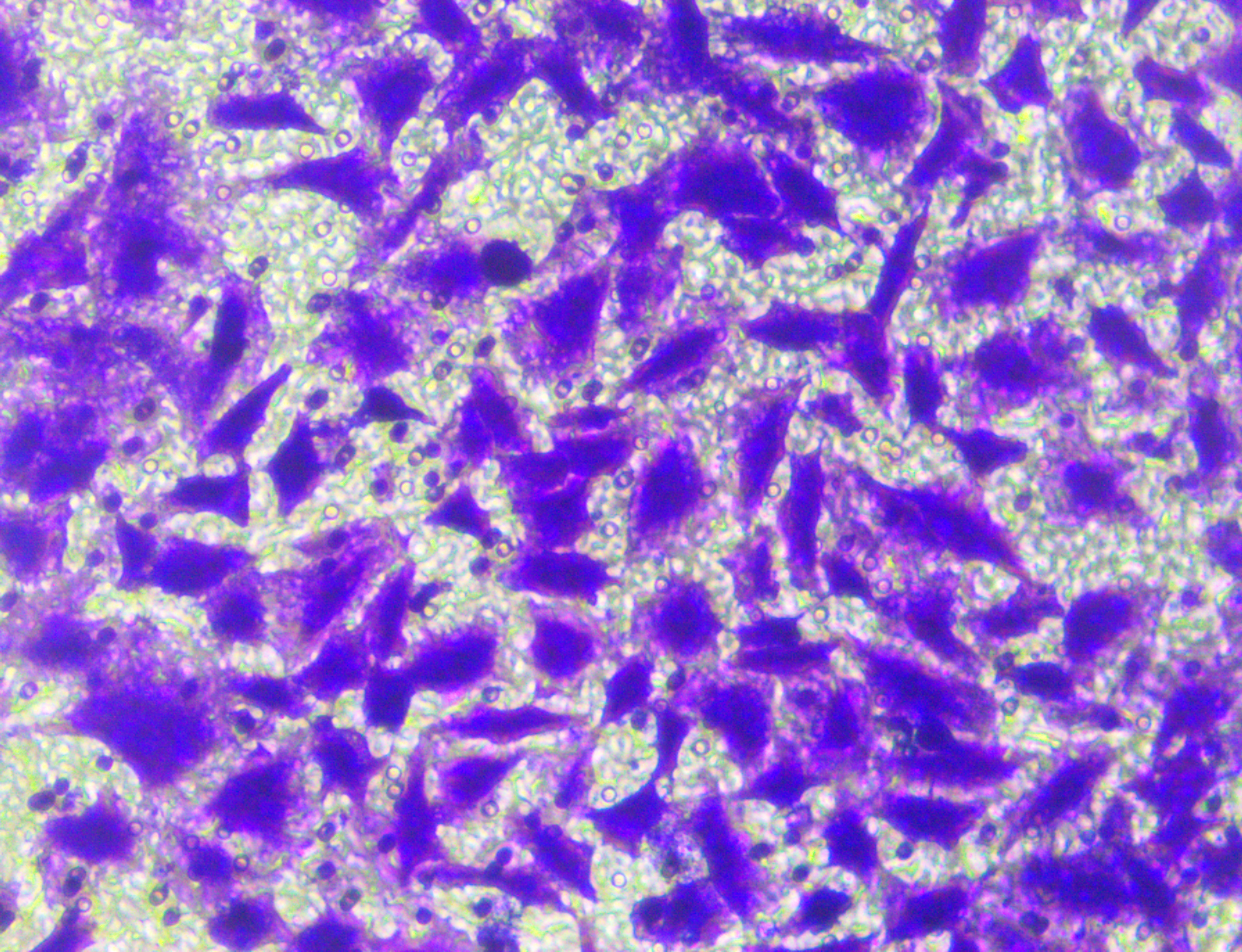

Supplement: Supplementary file 3 — Source data [file 41467_2022_35472_MOESM3_ESM.zip › Fig 6d/1. UT.tif]

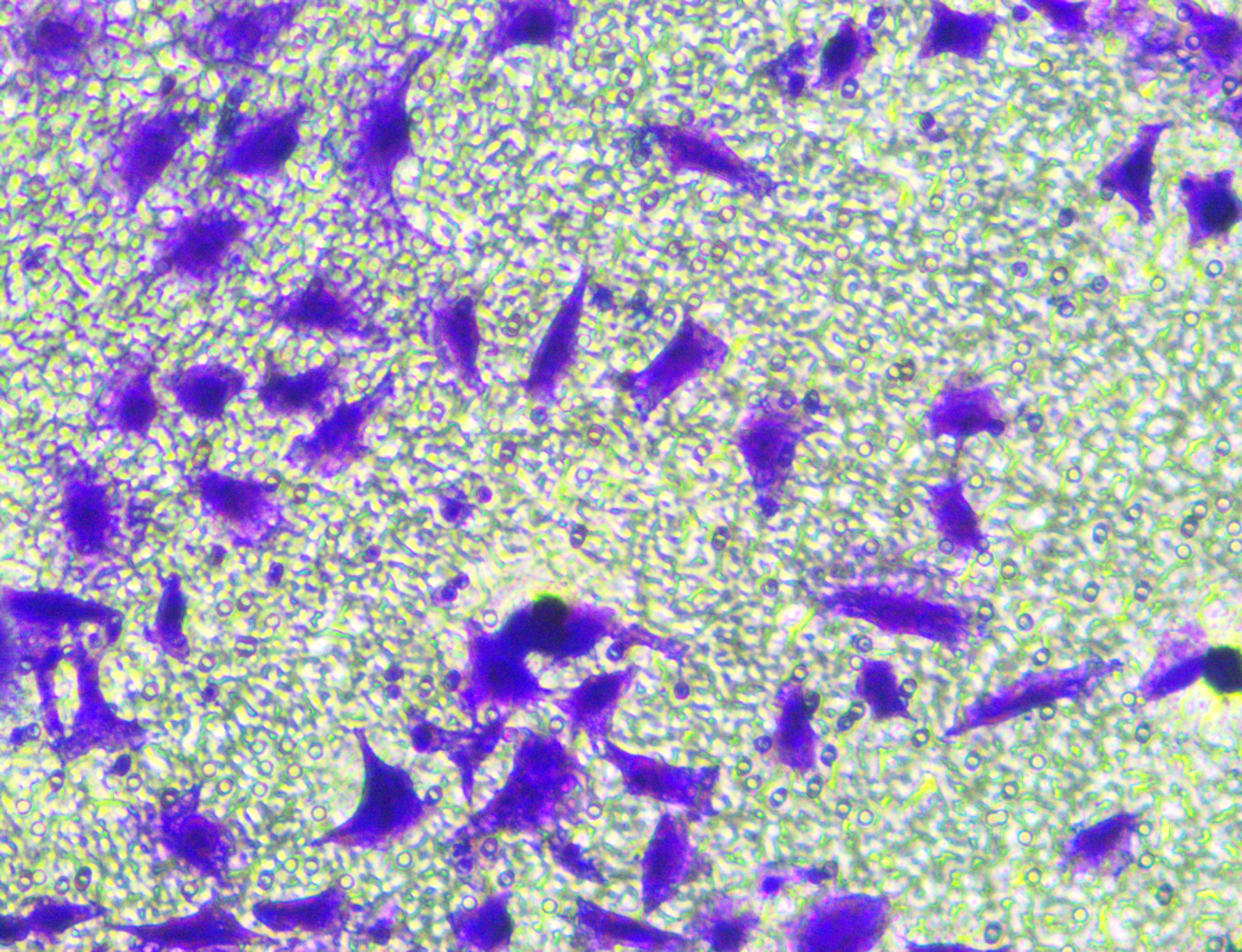

Supplement: Supplementary file 3 — Source data [file 41467_2022_35472_MOESM3_ESM.zip › Fig 6d/2. PBS.tif]

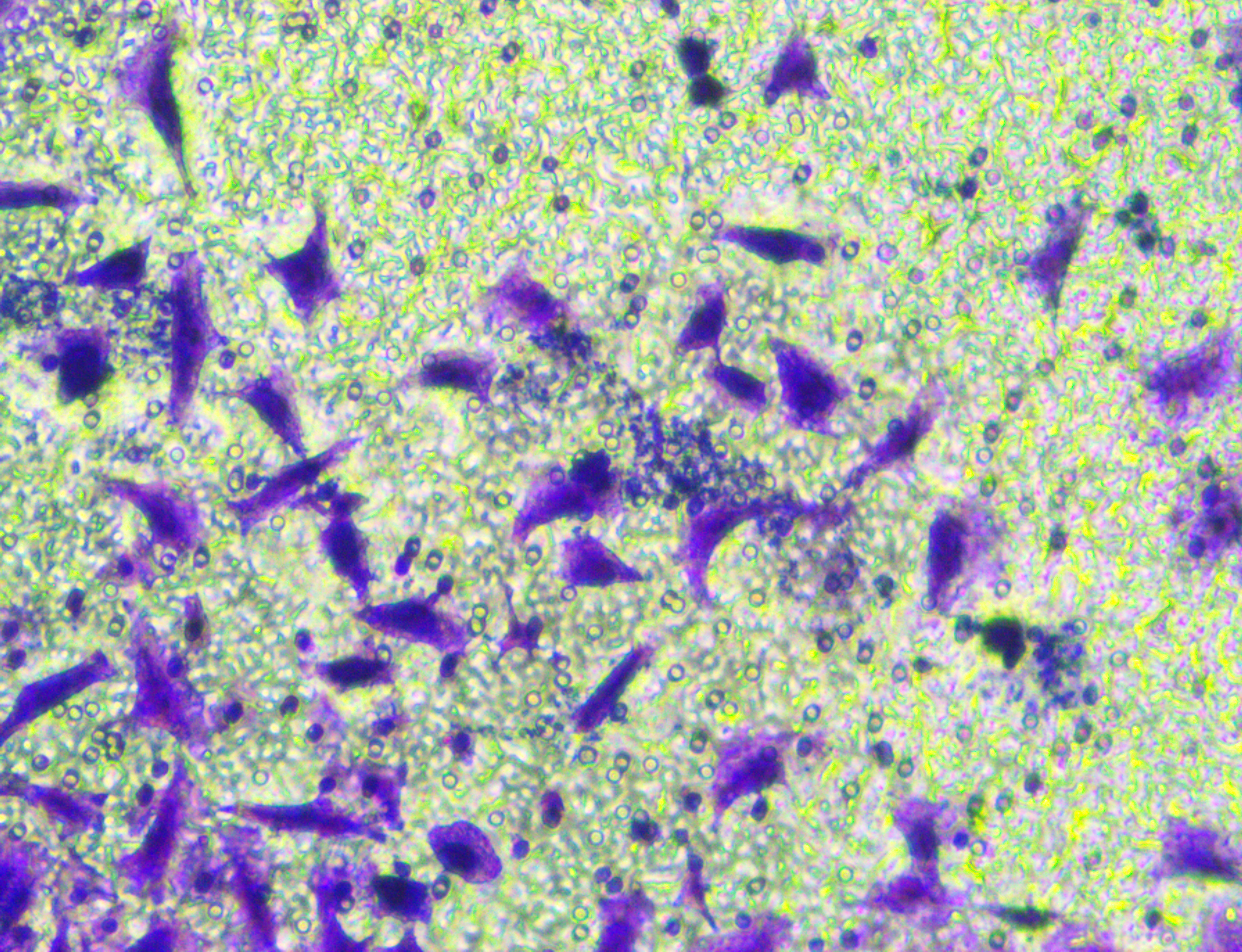

Supplement: Supplementary file 3 — Source data [file 41467_2022_35472_MOESM3_ESM.zip › Fig 6d/3. DniAS.tif]

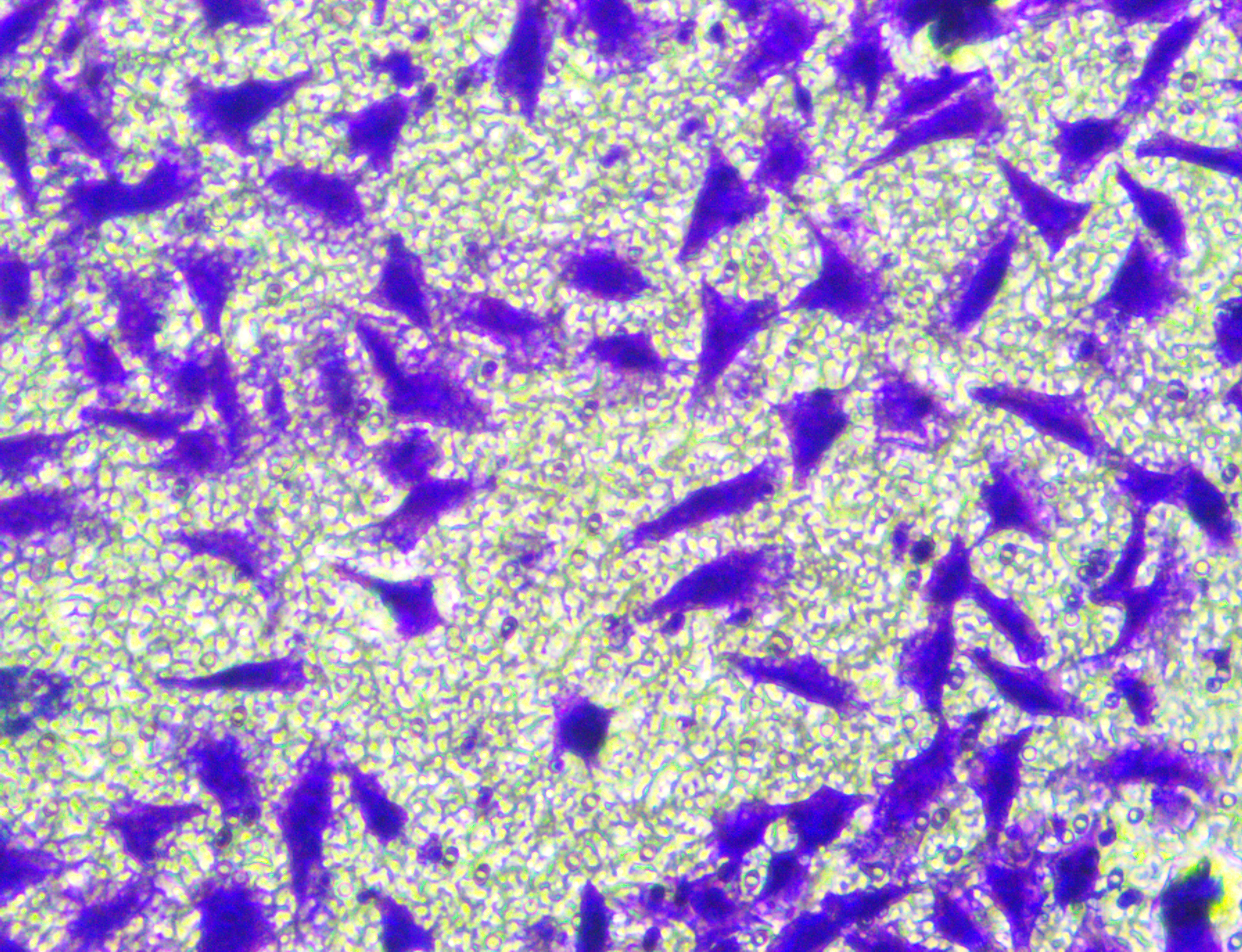

Supplement: Supplementary file 3 — Source data [file 41467_2022_35472_MOESM3_ESM.zip › Fig 6d/4. DAS.tif]

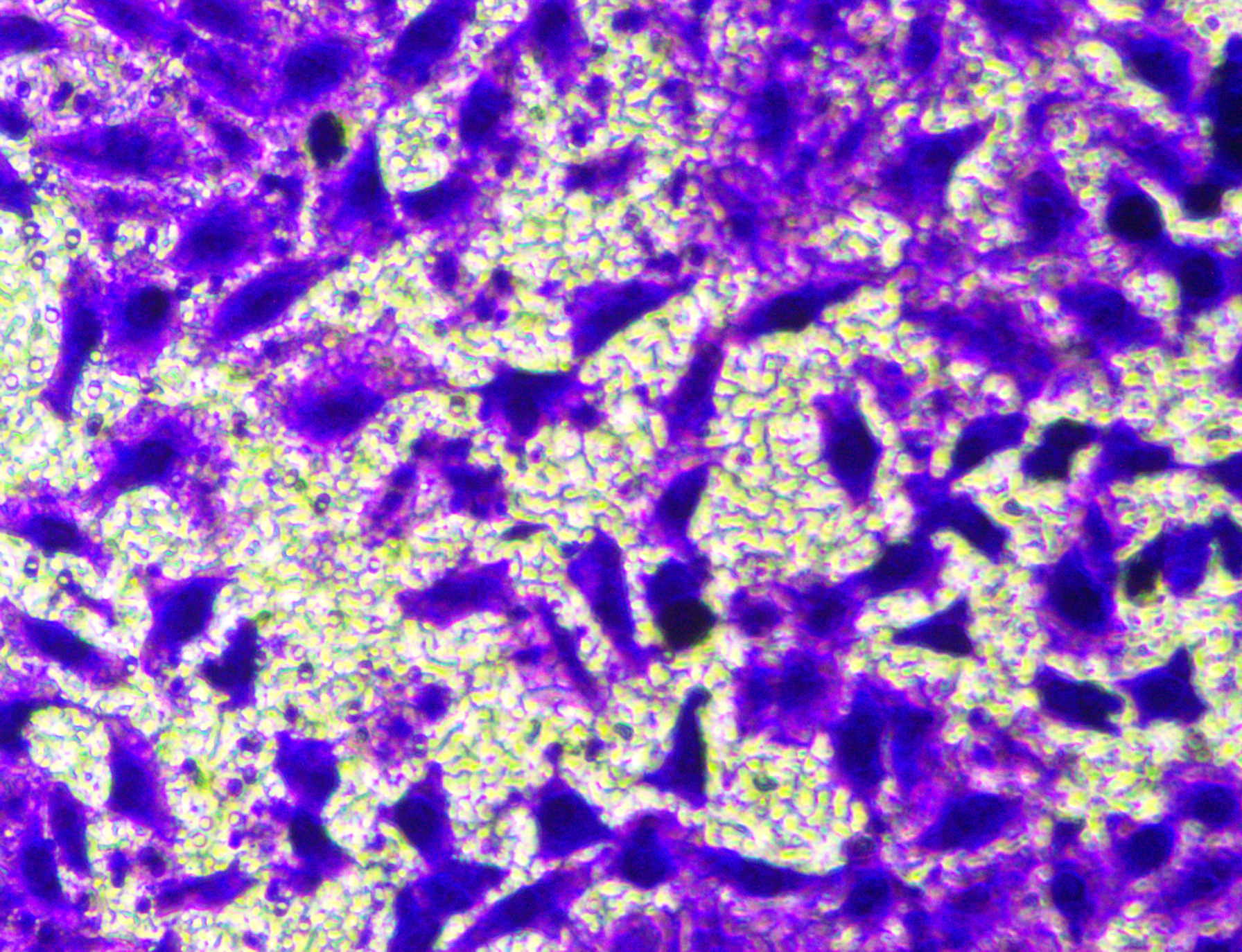

Supplement: Supplementary file 3 — Source data [file 41467_2022_35472_MOESM3_ESM.zip › Fig 6d/5. DniCNC.tif]

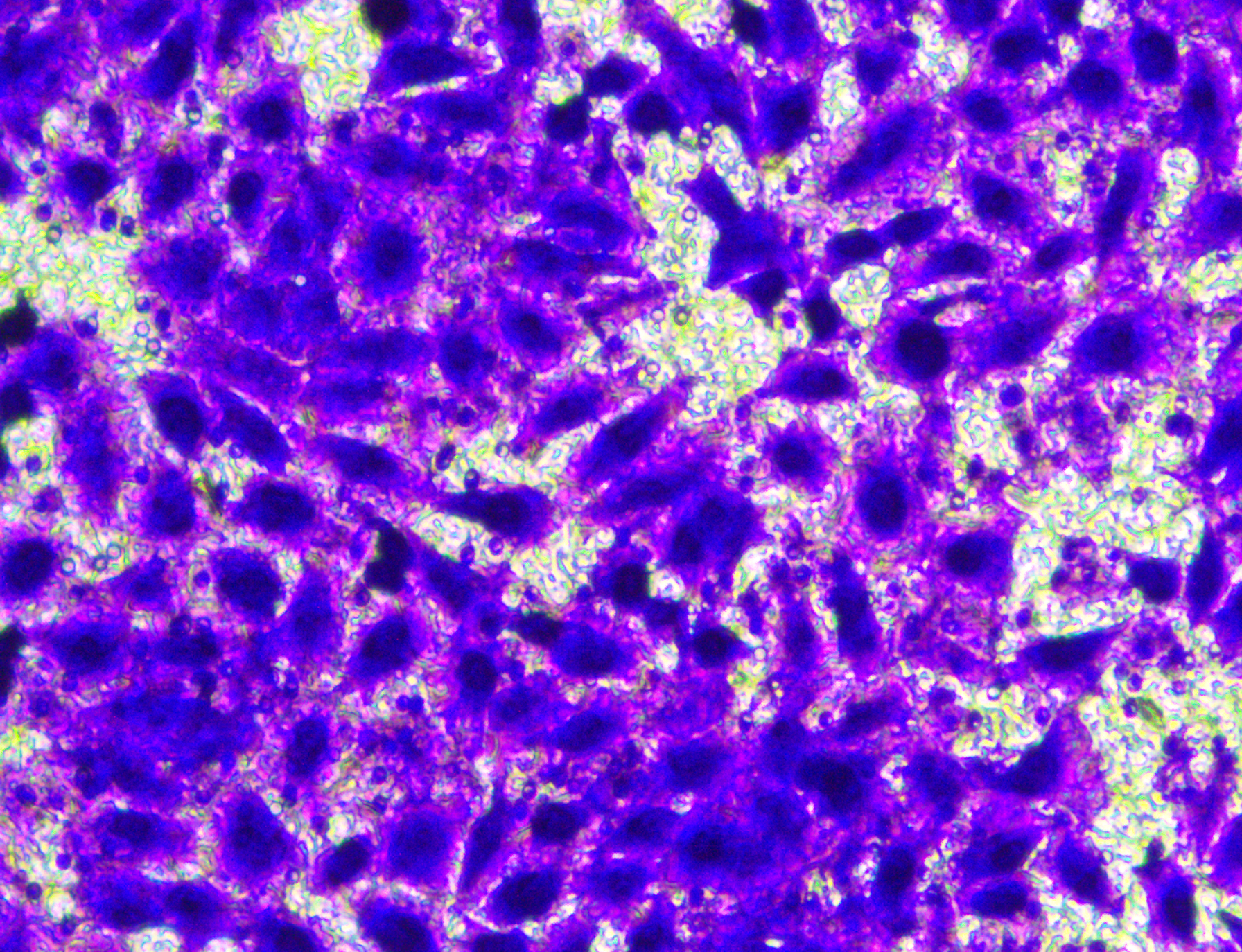

Supplement: Supplementary file 3 — Source data [file 41467_2022_35472_MOESM3_ESM.zip › Fig 6d/6. DCNC.tif]

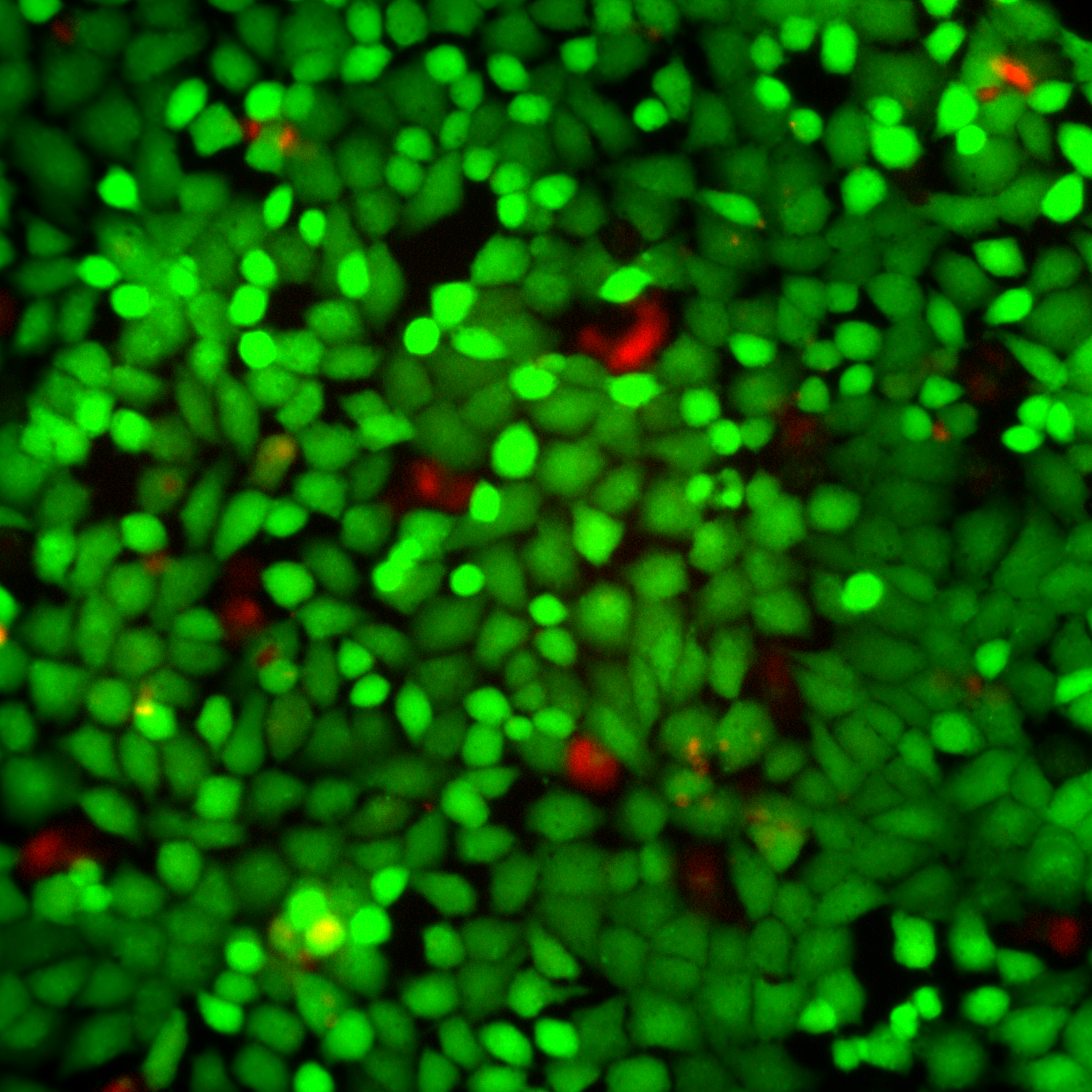

Supplement: Supplementary file 3 — Source data [file 41467_2022_35472_MOESM3_ESM.zip › Fig 6f/1. Blank.tif]

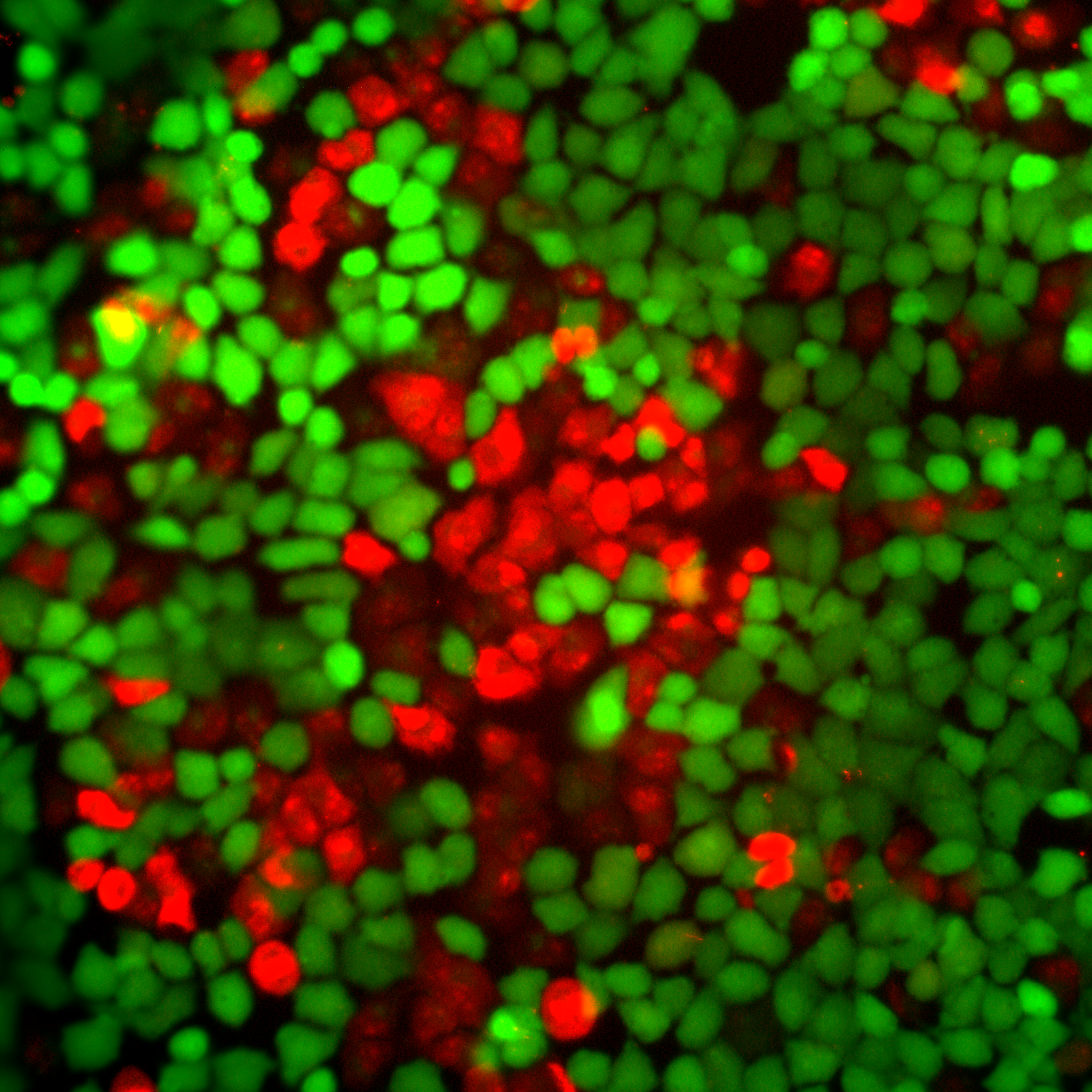

Supplement: Supplementary file 3 — Source data [file 41467_2022_35472_MOESM3_ESM.zip › Fig 6f/2. PBS.tif]

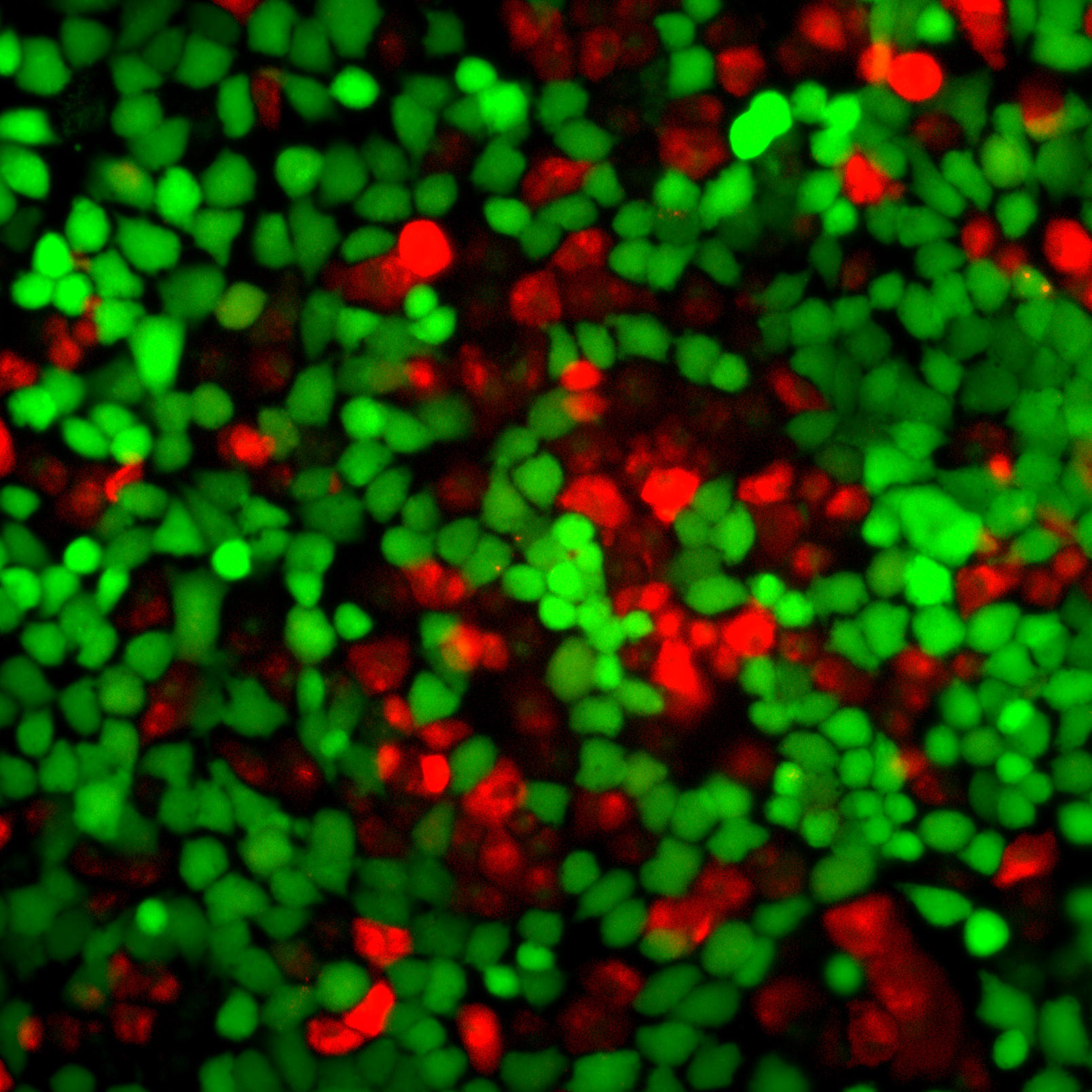

Supplement: Supplementary file 3 — Source data [file 41467_2022_35472_MOESM3_ESM.zip › Fig 6f/3. DniAS.tif]

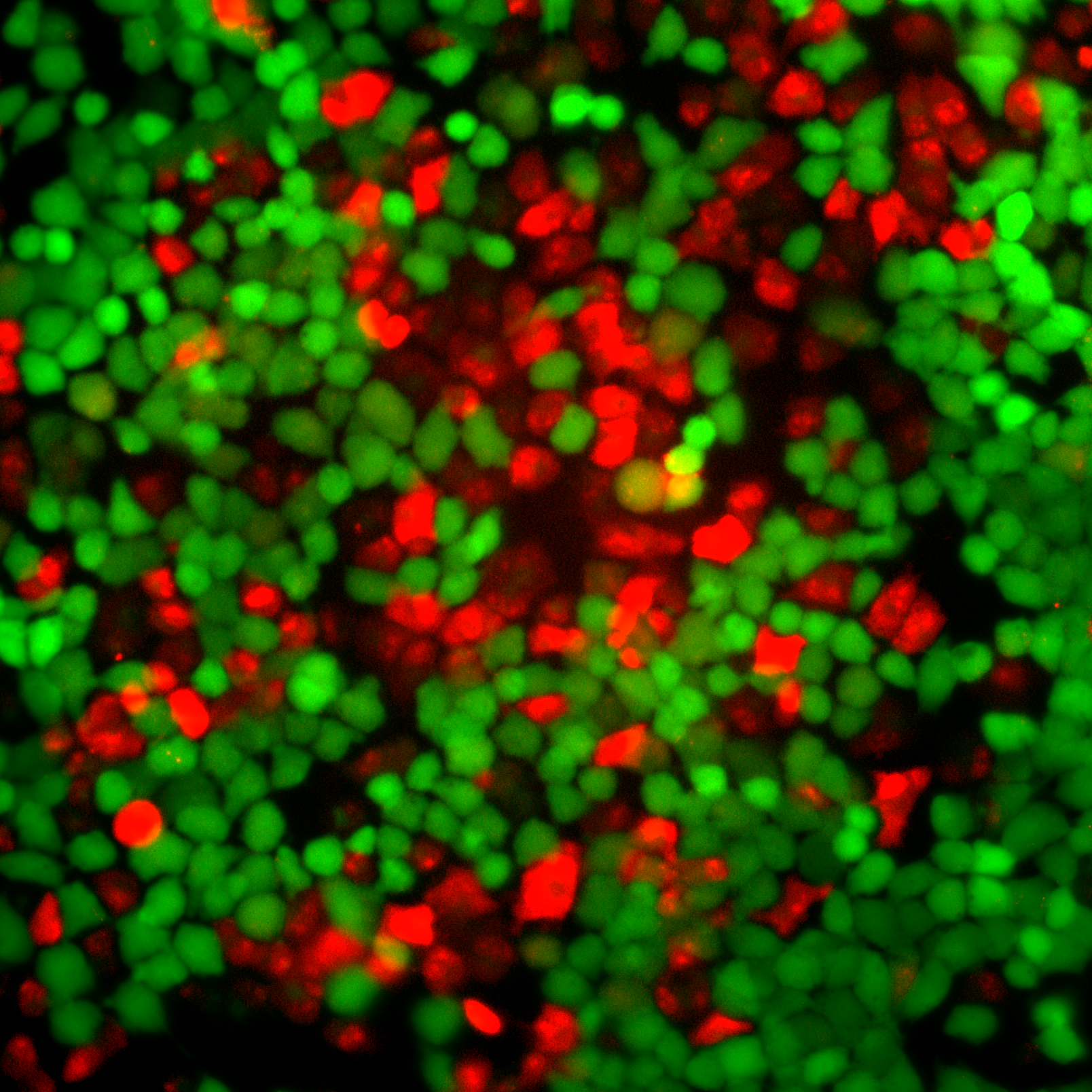

Supplement: Supplementary file 3 — Source data [file 41467_2022_35472_MOESM3_ESM.zip › Fig 6f/4. DAS.tif]

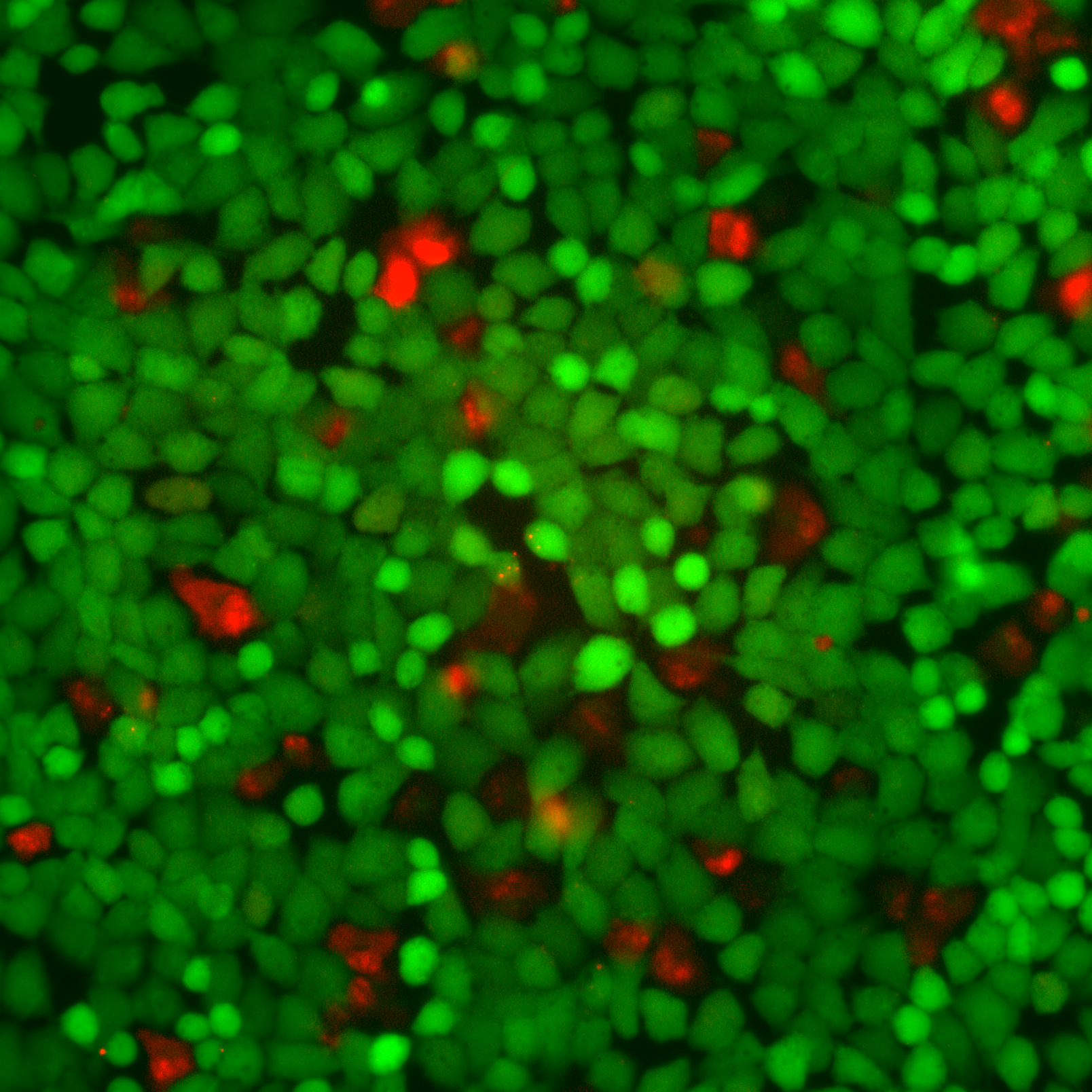

Supplement: Supplementary file 3 — Source data [file 41467_2022_35472_MOESM3_ESM.zip › Fig 6f/5. DniCNC.tif]

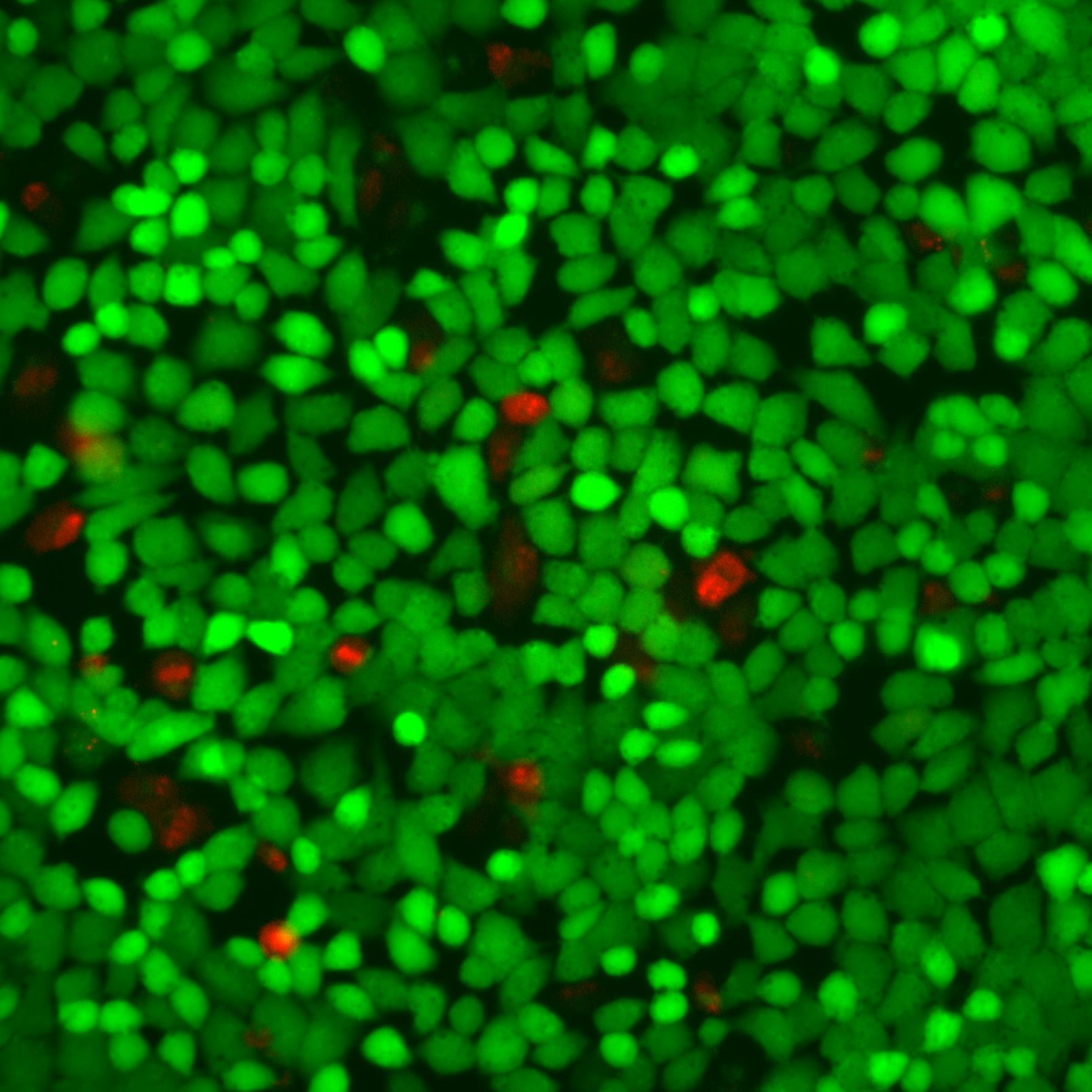

Supplement: Supplementary file 3 — Source data [file 41467_2022_35472_MOESM3_ESM.zip › Fig 6f/6. DCNC.tif]

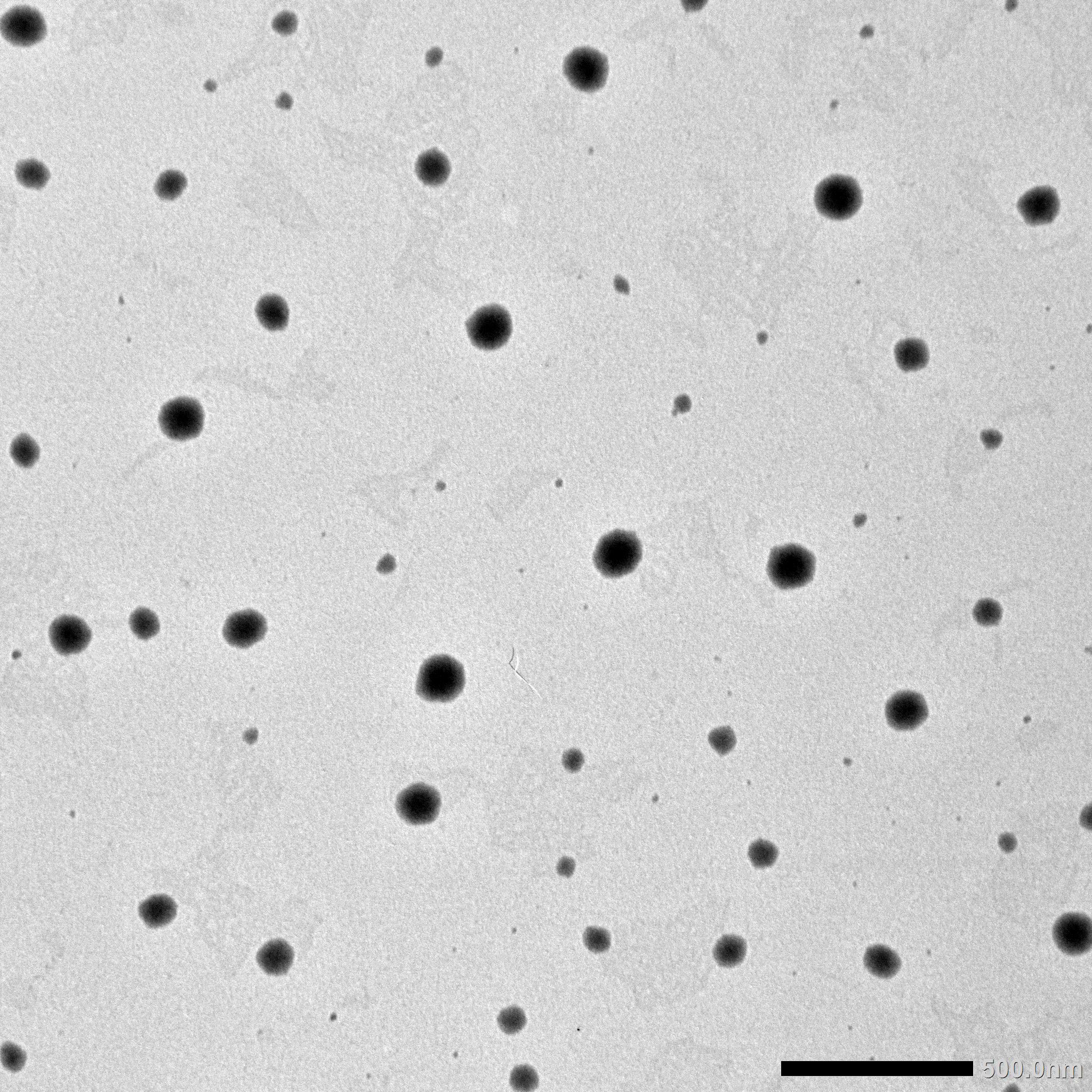

Supplement: Supplementary file 3 — Source data [file 41467_2022_35472_MOESM3_ESM.zip › Fig S10/Fig S10a.jpg]

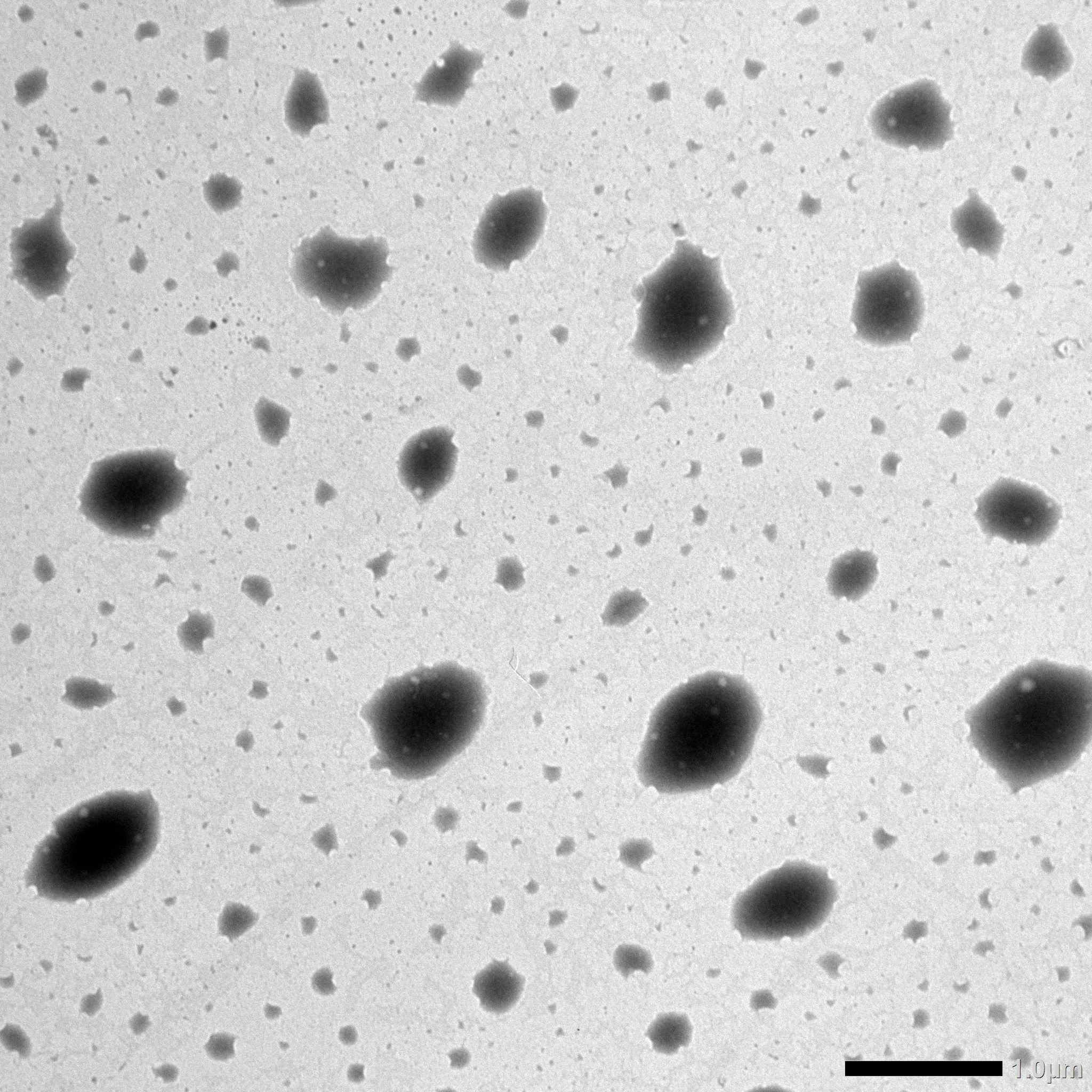

Supplement: Supplementary file 3 — Source data [file 41467_2022_35472_MOESM3_ESM.zip › Fig S10/Fig S10b.jpg]

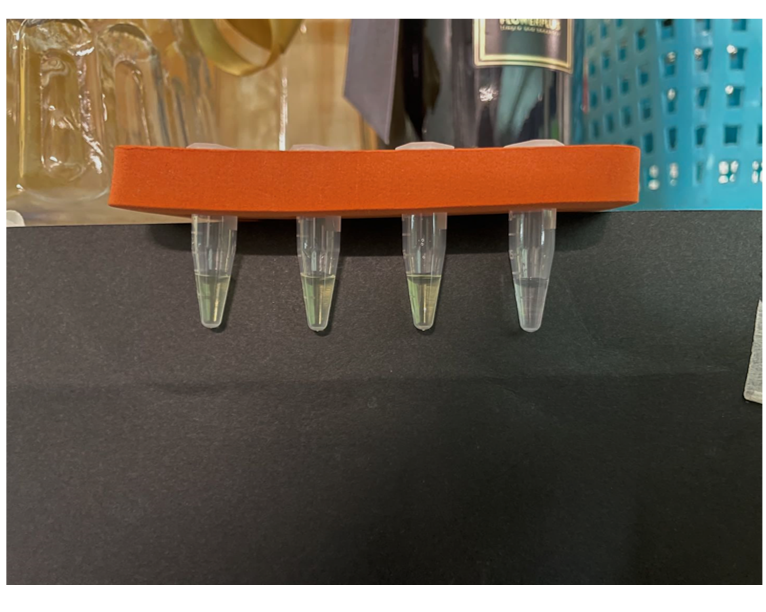

Supplement: Supplementary file 3 — Source data [file 41467_2022_35472_MOESM3_ESM.zip › Fig S12/1.tif]

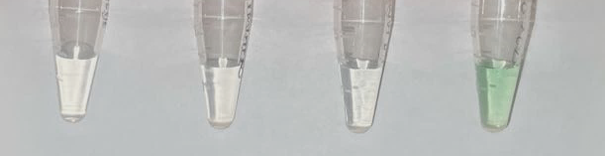

Supplement: Supplementary file 3 — Source data [file 41467_2022_35472_MOESM3_ESM.zip › Fig S13/1.tif]

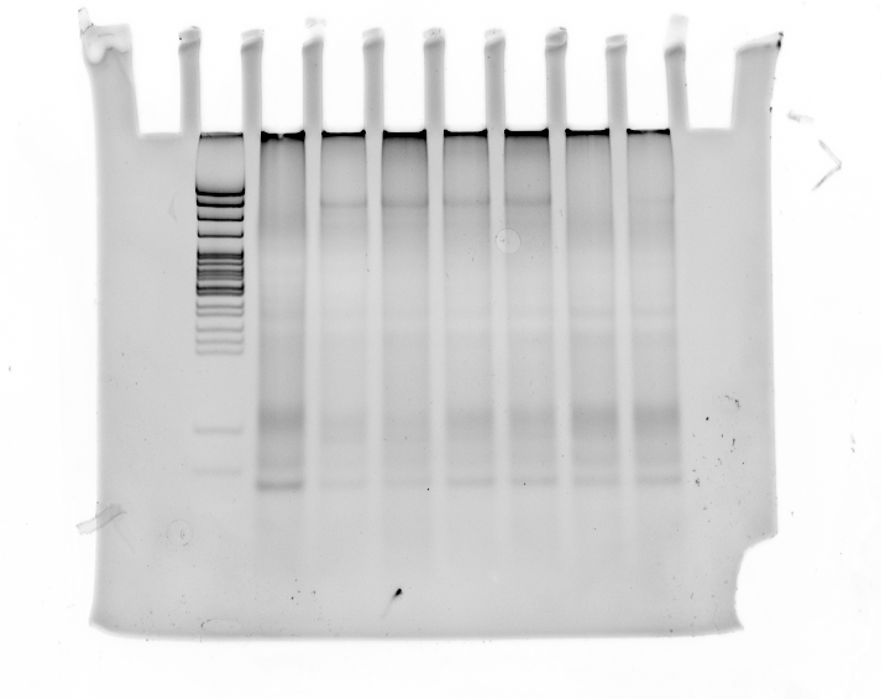

Supplement: Supplementary file 3 — Source data [file 41467_2022_35472_MOESM3_ESM.zip › Fig S15.tif]

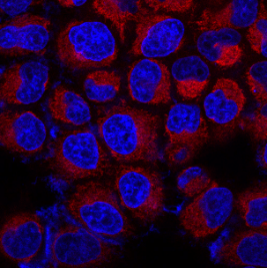

Supplement: Supplementary file 3 — Source data [file 41467_2022_35472_MOESM3_ESM.zip › Fig S19/DCNC-Cy5.png]

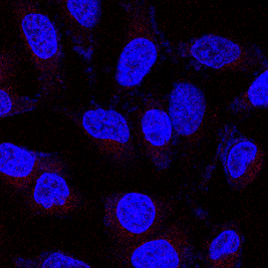

Supplement: Supplementary file 3 — Source data [file 41467_2022_35472_MOESM3_ESM.zip › Fig S19/DCNC-Cy5BHQ.png]

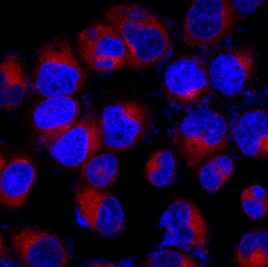

Supplement: Supplementary file 3 — Source data [file 41467_2022_35472_MOESM3_ESM.zip › Fig S19/DniCNC-Cy5.png]

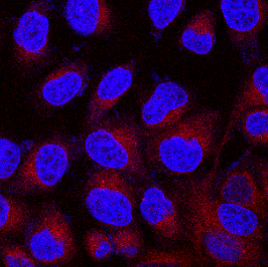

Supplement: Supplementary file 3 — Source data [file 41467_2022_35472_MOESM3_ESM.zip › Fig S19/DniCNC-Cy5BHQ.png]

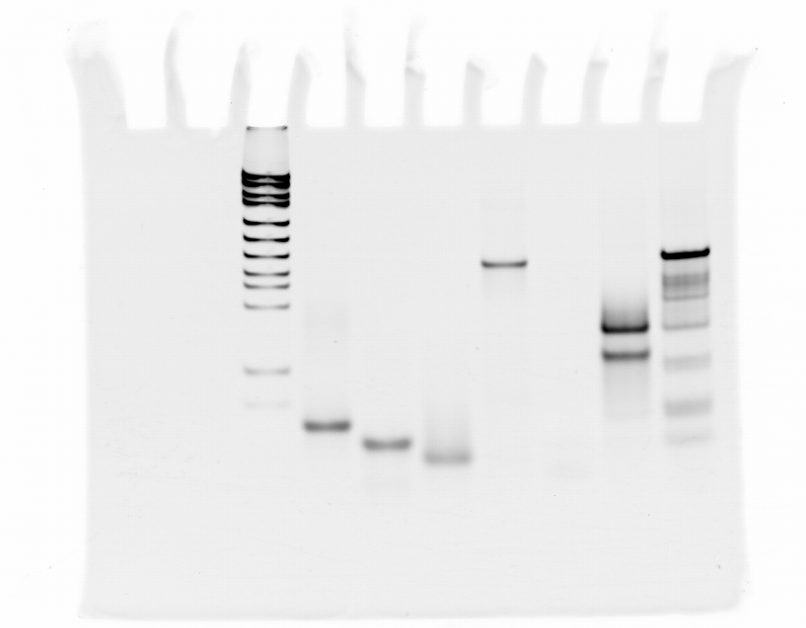

Supplement: Supplementary file 3 — Source data [file 41467_2022_35472_MOESM3_ESM.zip › Fig S1/Fig S1a.tif]
